# Integrative genomics analysis identifies promising SNPs and genes implicated in tuberculosis risk based on multiple omics datasets

**DOI:** 10.18632/aging.103744

**Published:** 2020-10-13

**Authors:** Mengqiu Xu, Jingjing Li, Zhaoying Xiao, Jiongpo Lou, Xinrong Pan, Yunlong Ma

**Affiliations:** 1 Department of Infectious Diseases, Shengzhou People’s Hospital, The First Affiliated Hospital of Zhejiang University Shengzhou Branch, Shengshou 312400, Zhejiang, China; 2 State Key Laboratory for Diagnosis and Treatment of Infectious Diseases, The First Affiliated Hospital, Collaborative Innovation Center for Diagnosis and Treatment of Infectious Diseases, Zhejiang University School of Medicine, Hangzhou 310003, Zhejiang, China; 3 Institute of Biomedical Big Data, Wenzhou Medical University, Wenzhou 325027, Zhejiang, China; 4 School of Biomedical Engineering, School of Ophthalmology and Optometry and Eye Hospital, Wenzhou Medical University, Wenzhou 325027, Zhejiang, China

**Keywords:** genetic variants, GWAS, risk genes, tuberculosis, gene expression

## Abstract

More than 10 GWASs have reported numerous genetic loci associated with tuberculosis (TB). However, the functional effects of genetic variants on TB remains largely unknown. In the present study, by combining a reported GWAS summary dataset (N = 452,264) with 3 independent eQTL datasets (N = 2,242) and other omics datasets downloaded from public databases, we conducted an integrative genomics analysis to highlight SNPs and genes implicated in TB risk. Based on independent biological and technical validations, we prioritized 26 candidate genes with eSNPs significantly associated with gene expression and TB susceptibility simultaneously; such as, *CDC16* (rs7987202, rs9590408, and rs948182) and *RCN3* (rs2946863, rs2878342, and rs3810194). Based on the network-based enrichment analysis, we found these 26 highlighted genes were jointly connected to exert effects on TB susceptibility. The co-expression patterns among these 26 genes were remarkably changed according to *Mycobacterium tuberculosis* (MTB) infection status. Based on 4 independent gene expression datasets, 21 of 26 genes (80.77%) showed significantly differential expressions between TB group and control group in mesenchymal stem cells, mice blood and lung tissues, as well as human alveolar macrophages. Together, we provide robust evidence to support 26 highlighted genes as important candidates for TB.

## INTRODUCTION

Tuberculosis (TB), a communicable respiratory disease, is major threat to human health in the world, especially in low and middle income countries in Asia [1–3]. There are approximately 10.4 million new cases and 1.7 million deaths worldwide in 2016 [4]. Although the advanced developments in diagnosis and treatment, accurate diagnosis of TB is still difficult and the healthcare and economic burdens of TB remain high. Complicated interactions among host, pathogen, and environmental factors contributed to the development of TB, of which the symptoms contain severe persistent coughing, fever, hemoptysis, chest pain and weight loss [5]. Family and twin studies [6–8] have reported that host genetic components play important roles in contributing risk to TB. Thereby, substantial interests in identifying the genetic components implicated in the aetiology of TB are growing.

In previous decades, TB has been a focus of many candidate gene-based and genome-wide association studies (GWAS). For candidate gene-based association studies on TB, which are dependent on a prior hypothesis that we know the knowledge of the functions of candidate genes, numerous genes with pressing single nucleotide polymorphisms (SNPs) have been identified to be associated with TB [9–15]. For example, genetic variations in *TLR* genes have reported to show associations with TB and clinical outcomes in previous studies [9–11]. With the advances of next-generation sequencing or microarray technology, the approach of GWAS based on powerful hypothesis-free methodology has been extensively applied to investigate the genetic architectures of complex diseases including TB and identify thousands of common risk SNPs. Since the first GWAS on TB was reported in the year of 2010 [16], subsequently many GWASs [17–25] have demonstrated associations between numerous common SNPs and TB among European and other ancestry populations. For example, there were 4 common SNPs identified to be significantly associated with TB via GWASs in Russian or African populations [16–18]. Nevertheless, despite intensifying GWAS studies have been conducted, much of the heritability of TB remains missing.

The vast majority of GWAS-identified significant or suggestive SNPs associated with complex diseases were located in non-coding genomic regions [8, 26]. Consistently, most of previously identified susceptibility variants associated with TB were mapped into non-coding regions [27]. Thus, it is plausible to infer that these GWAS-identified variants may have regulatory effects on influencing the expression level of specific gene instead of altering the function of its protein. A recent multi-cohort study [28] demonstrated that aberrant expression signature of a three-gene set (*GBP5*, *DUSP3*, and *KLF2*) is highly diagnostic for active TB. Furthermore, an accruing number of studies have concentrated on exploration of susceptibility genes whose aberrant expression are associated with diseases and traits of medical importance in humans due to pleiotropy [28–32]. For example, by using an integrative analysis of GWAS summary-level, mQTL and eQTL data, our team [33] previously found 34 important genes including *PRKCZ*, *ARHGEF3*, and *CDKN1A* with various critical SNPs contribute risk to the comorbidity of schizophrenia and smoking behaviors. Many novel risk genes identified by numerous integrative genomics studies were hard to be detected by a GWAS alone.

To the best of our knowledge, there was no systematical integrative genomics analysis on TB conducted to reveal the genome-wide regulatory effects of SNPs on gene expression. In the present study, we applied a two-stage designed analysis to identified risk SNPs, genes and pathways for TB. We first used the Sherlock integrative analysis to identify cis- and trans-regulatory effects of SNPs on expression abundance of interested genes via incorporating a large-scale GWAS summary dataset (N = 452,264) with a blood-based eQTL dataset (N = 1,490). Then, using the Sherlock analysis with same parameters, we adopted two independent eQTL datasets based on blood (N = 369) and lung tissue (N = 383) to replicate the results in the discovery stage. Furthermore, we employed a series of bioinformatics analyses including MAGMA analysis, *in silico* permutation analysis, pathways/diseases-based enrichment analysis, network-based enrichment analysis, DGIdb enrichment analysis, and co-expression analysis based on multi-omics data to highlight TB-associated risk genes with strong evidence.

## RESULTS

### Identification of TB-associated genes in the discovery stage

**Figure 1:**
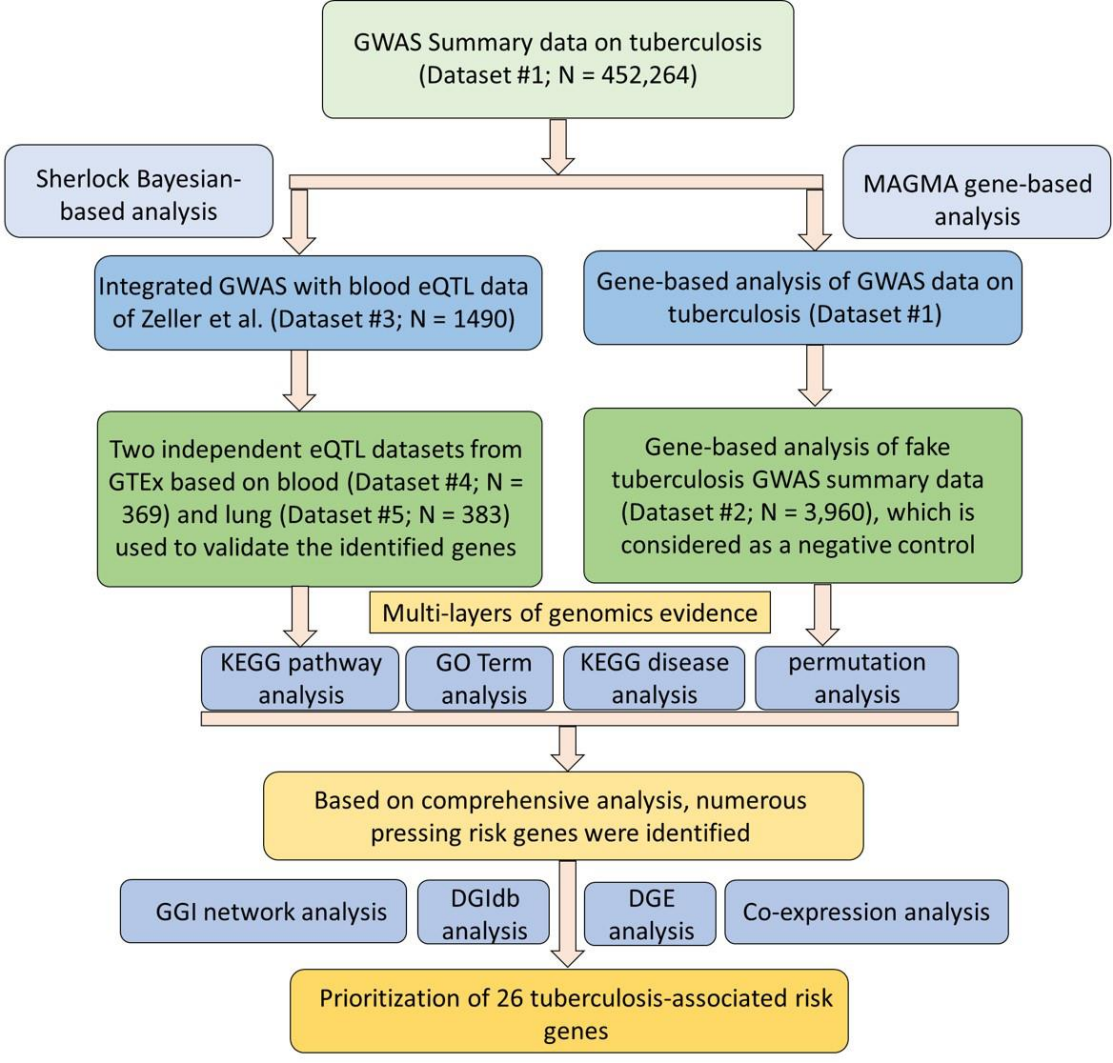
**Workflow of current comprehensive genomics analysis.**

**Figure 2:**
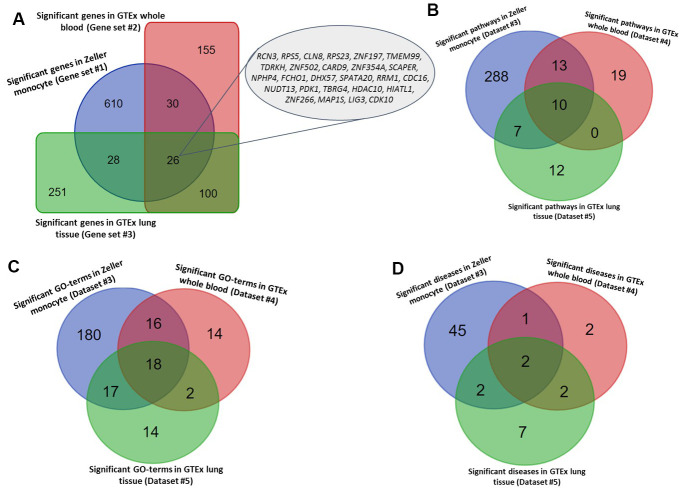
**Identified tuberculosis-related risk genes, pathways, and GO-terms.** (**A**) Common significant genes identified from the Sherlock analysis based on Gene sets #1, #2, and #3. (**B**) Common significant pathways enriched by genes identified from the Sherlock analysis cross 3 gene sets (i.e., Gene sets #1, #2, and #3). (**C**) Common significant GO-terms enriched by genes identified from the Sherlock analysis cross 3 gene sets (i.e., Gene sets #1, #2, and #3). (**D**) Common significant KEGG or NHGRI GWAS Catalog diseases enriched by genes identified from the Sherlock analysis cross 3 gene sets (i.e., Gene sets #1, #2, and #3).

In the discovery stage, we conducted a Sherlock Bayesian integrative analysis by incorporating GWAS summary statistics (Dataset #1; N = 452,264) with eQTL data (Dataset #3; N = 1,490) to identify aberrant expressed genes with eSNPs implicated in TB risk (Figure 1). There were a number of 694 genes identified to be significantly associated with TB risk (Gene set #1, Simulated P ≤ 0.05; Figure 2A and Supplementary Table 1). For example, the top-ranked significant genes were *SIPA1L1* (Simulated P = 1.26 × 10^-5^), *GSTA2* (Simulated P = 1.61 × 10^-4^), *TIGD6* (Simulated P = 3.02 × 10^-4^), *TSPYL4* (Simulated P = 4.22 × 10^-4^), and *POLG2* (Simulated P = 4.68 × 10^-4^). Interestingly, among these identified significant genes, 4 genes of *C2CD2*, *HLA-DRB6*, *HLA-DQB1*, and *LPCAT2* have been reported to be associated with TB in earlier studies (Supplementary Figure 1 and Supplementary Table 1). In addition, there existed 7 genes documented to be associated with respiratory relevant diseases, such as asthma and chronic obstructive pulmonary disease (Supplementary Figure 1 and Supplementary Table 1); and 38 genes identified to be associated with lung function and related diseases, such as lung cancer and adenocarcinoma (Supplementary Figure 1 and Supplementary Table 1).

**Table 1:** Sherlock-based Bayesian genomics analysis identifies 26 candidate genes associated with tuberculosis risk.

**Gene**	**Simulated P values in Gene set #1**	**Simulated P values in Gene set #2**	**Simulated P values in Gene set #3**	**MAGMA-based P values in Gene set #4**	MAGMA-based P values in Gene set #5*	**T-test P values in GSE133803**	**Anova P values in GSE1440943**	**Anova P values in GSE1440944**	**Anova P values in GSE139825**
*CDC16*	6.21E-3	1.20E-2	1.38E-2	5.45E-3	NA	5.63E-4	8.16E-4	8.17E-2	8.34E-02
*HIATL1*	1.51E-2	2.05E-2	1.16E-2	0.12	0.59	1.83E-7	7.33E-4	2.17E-2	0.11
*RCN3*	2.01E-2	1.40E-2	7.14E-3	4.41E-3	0.81	8.73E-3	0.13	1.56E-2	0.78
*FCHO1*	2.93E-2	1.31E-2	1.64E-2	3.53E-2	0.80	0.27	4.01E-2	9.03E-7	7.12E-03
*CDK10*	3.08E-2	3.35E-2	3.70E-2	2.62E-2	NA	0.49	8.70E-3	8.92E-5	7.93E-02
*SCAPER*	3.60E-2	1.95E-2	1.62E-2	1.38E-2	0.20	8.84E-4	0.14	3.34E-2	0.65
*LIG3*	3.98E-2	1.66E-2	3.73E-2	2.91E-2	0.28	1.86E-3	8.88E-2	2.23E-2	8.15E-02
*RRM1*	4.82E-2	2.67E-2	2.04E-2	0.49	0.29	3.24E-3	1.29E-2	5.23E-4	0.11
*PDK1*	3.79E-3	2.54E-2	1.87E-3	5.61E-3	3.51E-3	0.83	1.14E-2	7.84E-4	3.77E-02
*TMEM99*	5.18E-3	1.86E-2	3.02E-3	1.36E-3	0.47	2.00E-3	NA	NA	0.11
*SPATA20*	7.80E-3	4.13E-3	3.51E-3	4.55E-4	0.39	1.98E-3	0.23	0.38	2.23E-02
*TDRKH*	8.18E-3	1.01E-2	8.50E-3	1.17E-2	0.15	0.83	0.14	1.45E-3	7.42E-02
*NPHP4*	1.15E-2	3.69E-2	2.98E-2	2.01E-2	0.35	0.32	0.42	8.73E-2	0.15
*CLN8*	2.10E-2	1.13E-2	1.19E-2	1.46E-2	0.40	8.18E-6	0.10	0.17	1.04E-02
*DHX57*	3.05E-2	1.48E-2	8.48E-3	1.19E-2	0.20	0.48	2.80E-2	0.10	0.18
*RPS5*	3.71E-2	4.65E-2	4.41E-2	0.19	0.93	2.11E-4	3.73E-4	8.54E-2	2.71E-04
*MAP1S*	4.03E-2	8.39E-3	6.70E-3	1.30E-2	6.0E-2	1.01E-2	NA	NA	0.78
*HDAC10*	2.34E-3	2.36E-2	2.53E-2	0.21	NA	0.42	0.15	4.23E-2	0.89
*TBRG4*	1.67E-2	4.53E-2	3.66E-2	0.25	0.80	0.11	0.11	2.99E-3	0.28
*CARD9*	1.73E-2	3.86E-2	1.74E-2	0.13	NA	5.48E-2	NA	NA	0.21
*ZNF354A*	1.74E-2	3.75E-2	4.14E-2	2.74E-2	0.98	0.29	NA	NA	3.80E-02
*ZNF266*	3.66E-2	3.94E-2	3.09E-2	1.09E-2	0.41	0.11	NA	NA	0.18
*ZNF502*	4.23E-2	2.18E-2	1.99E-2	1.23E-2	0.53	0.14	NA	NA	0.14
*ZNF197*	4.32E-2	1.81E-2	2.57E-2	9.53E-4	0.71	5.40E-2	NA	NA	6.69E-03
*NUDT13*	3.27E-2	3.78E-2	3.57E-2	6.0E-2	0.78	0.28	0.41	0.56	0.22
*RPS23*	7.88E-7	2.22E-2	1.66E-2	0.54	2.26E-2	5.45E-5	0.24	0.82	0.49

**Note:** NA means not available, which were largely due to that the expression levels of these genes very lower or the qualities were not feasible.

*Gene set #5 is generated from MAGMA analysis on fake tuberculosis as a negative control.

To annotate the molecular functions and biological pathways of these 694 identified genes, we performed a functional enrichment analysis by using the KOBAS tool. As for pathway enrichment analysis, 305 pathways were significantly enriched by these TB-associated genes (FDR ≤ 0.05; Figure 2B and Supplementary Table 2). For example, the pathways of metabolism (FDR = 1.78 × 10^-28^), immune system (FDR = 2.12 × 10^-21^), metabolic pathways (FDR = 7.75 × 10^-19^), and tuberculosis (FDR = 4.44 × 10^-5^). Furthermore, 231 GO-terms (FDR ≤ 0.05; Figure 2C and Supplementary Table 3) and 50 diseases-terms (FDR ≤ 0.05; Figure 2D and Supplementary Table 4) were significantly overrepresented by these TB-relevant genes.

**Table 2:** 10 common pathways enriched by tuberculosis-associated genes across 3 identified gene sets.

**Pathway ID**	**Common pathways**	**Gene set #1**		**Gene set #2**		**Gene set #3**	
		**Proportion of risk genes**	**FDR**	**Proportion of risk genes**	**FDR**	**Proportion of risk genes**	**FDR**
R-HSA-1430728	Metabolism	4.96%	1.78E-28	1.35%	1.19E-4	1.69%	3.16E-5
R-HSA-74160	Gene expression (Transcription)	4.97%	1.06E-19	1.17%	1.90E-2	1.80%	1.24E-4
R-HSA-392499	Metabolism of proteins	3.93%	3.94E-16	1.29%	3.72E-4	1.64%	5.19E-5
R-HSA-73857	RNA Polymerase II Transcription	4.71%	8.33E-16	1.22%	1.90E-2	1.67%	1.37E-3
R-HSA-212436	Generic Transcription Pathway	4.78%	7.63E-15	1.17%	3.79E-2	1.84%	4.24E-4
R-HSA-597592	Post-translational protein modification	4.32%	4.60E-14	1.42%	1.09E-3	1.63%	1.34E-3
R-HSA-5653656	Vesicle-mediated transport	5.38%	1.36E-10	1.64%	1.68E-2	1.79%	3.09E-2
R-HSA-1643685	Disease	4.29%	3.11E-10	1.81%	1.33E-4	1.43%	4.22E-2
R-HSA-199991	Membrane Trafficking	5.23%	2.17E-9	1.74%	1.10E-2	1.74%	4.22E-2
R-HSA-382551	Transport of small molecules	3.33%	7.01E-4	1.53%	2.19E-2	1.67%	3.98E-2

**Note:** Proportion of risk genes: these identified risk genes accounted for the proportion of all genes in each pathway enriched by these genes. FDR values were calculated by using the method of Benjamini-Hochberg false discovery rate (FDR) correction.

### Validation of TB-associated genes in the replication stage

Furthermore, we utilized two independent eQTL datasets (Datasets #4 and #5) to carry out the Sherlock Bayesian analysis with same parameters for validation. Based on these two independent datasets, we identified 311 significant genes for Dataset #4 based on whole blood samples (Gene set #2, Simulated P ≤ 0.05; Supplementary Table 5) and 405 significant genes for Dataset #5 based on lung tissues (Gene set #3, Simulated P ≤ 0.05; Supplementary Table 6). Among these genes, 3 genes of *ESPPRB*, *GLRX5*, and *LRPAP1* have been reported to be linked with TB in earlier studies (Supplementary Figure 2). 30 and 18 genes have been documented to be associated with lung-related diseases (Supplementary Table 5) and respiratory-related diseases (Supplementary Table 6), separately. Interestingly, there existed 7 genes showing associations with both lung-related diseases and respiratory disease (Supplementary Figure 2). Compared with genes identified in the discovery stage (Gene set #1), we found 26 genes were significantly replicated by the Sherlock analysis of both datasets in the replication stage (Gene sets #2 and #3) (Figure 2A and Table 1). Most of these 26 highlighted genes were highly expressed in human lung tissue (Supplementary Figures 3–15).

For the functional enrichment analyses of these two gene sets, 40 pathways, 50 GO-terms, and 7 diseases-terms (FDR ≤ 0.05; Supplementary Tables 7–9) were significantly overrepresented by Gene set #2, as well as 29 pathways, 51 GO-terms, and 13 diseases-terms (FDR ≤ 0.05; and Supplementary Tables 10–12) were significantly enriched by Gene set #3. Furthermore, we found 10 common pathways, 18 common GO-terms, and 2 common enriched diseases (FDR ≤ 0.05; Figure 2B–2D, Tables 2, 3, and Supplementary Table 13) were significantly enriched by all the 3 independent gene sets.

**Table 3:** 18 common GO-terms enriched by tuberculosis-associated genes across 3 identified gene sets.

**GO-terms ID**	**GO-terms**	**Gene set #1**		**Gene set #2**		**Gene set #3**	
		**Proportion of risk genes**	**FDR**	**Proportion of risk genes**	**FDR**	**Proportion of risk genes**	**FDR**
GO:0005622	Intracellular	4.44%	4.09E-24	1.26%	2.89E-4	1.53%	1.07E-4
GO:0110165	Cellular Anatomical Entity	3.77%	2.12E-21	0.91%	2.42E-2	1.47%	3.36E-5
GO:0044237	Cellular Metabolic Process	3.80%	4.45E-15	1.04%	1.97E-2	1.18%	3.51E-2
GO:0043227	Membrane-Bounded Organelle	3.79%	4.45E-15	1.33%	1.74E-4	1.63%	5.19E-5
GO:0043229	Intracellular Organelle	3.76%	1.97E-13	1.77%	8.74E-8	1.61%	1.36E-4
GO:0005488	Binding	3.47%	2.19E-13	1.38%	4.34E-5	1.56%	5.19E-5
GO:0005737	Cytoplasm	3.90%	5.71E-13	1.46%	1.63E-4	1.40%	8.80E-3
GO:0005515	Protein Binding	3.85%	5.71E-13	1.24%	3.39E-3	1.90%	1.41E-5
GO:1901363	Heterocyclic Compound Binding	5.17%	6.56E-12	1.60%	6.97E-3	2.09%	9.91E-4
GO:0019222	Regulation Of Metabolic Process	3.83%	5.78E-8	1.68%	4.24E-4	2.15%	5.19E-5
GO:0016787	Hydrolase Activity	4.72%	4.28E-7	1.52%	4.74E-2	1.85%	3.47E-2
GO:0031982	Vesicle	4.20%	3.60E-6	1.95%	1.57E-3	1.95%	1.14E-2
GO:0008152	Metabolic Process	2.45%	2.16E-5	1.03%	6.97E-3	1.19%	1.14E-2
GO:0005654	Nucleoplasm	4.43%	5.31E-5	2.11%	6.08E-3	2.11%	2.70E-2
GO:0000166	Nucleotide Binding	4.51%	3.62E-4	1.86%	4.95E-2	3.45%	1.24E-4
GO:0003723	Rna Binding	5.16%	1.42E-2	3.87%	6.80E-3	4.52%	3.95E-3
GO:1901265	Nucleoside Phosphate Binding	3.35%	2.00E-2	2.32%	6.61E-3	2.32%	2.70E-2
GO:1990904	Ribonucleoprotein Complex	6.33%	3.85E-2	5.06%	2.19E-2	6.33%	9.07E-3

**Note:** Proportion of risk genes: these identified risk genes accounted for the proportion of all genes in each pathway enriched by these genes. FDR values were calculated by using the method of Benjamini-Hochberg false discovery rate (FDR) correction.

### MAGMA-based gene analysis for technical replication

**Figure 3:**
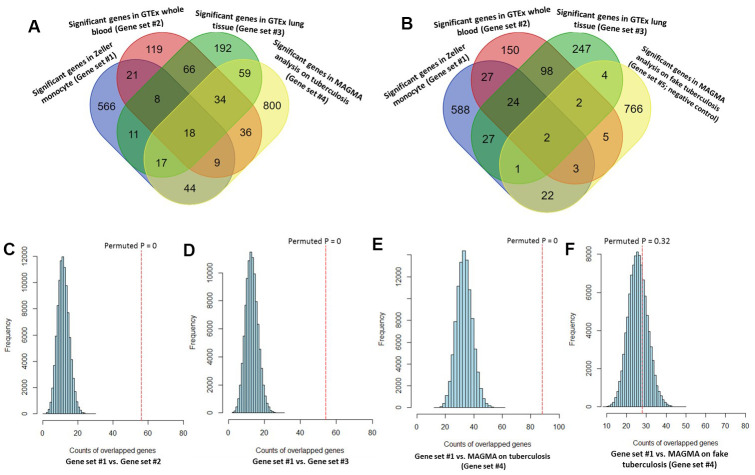
**Consistent evidence support Sherlock-identified genes implicated in tuberculosis (TB).** (**A**) Venn diagram shows that common genes between Sherlock-identified genes of Gene sets #1, #2, and #3 and MAGMA-identified genes on TB (Gene set #4). (**B**) Venn diagram shows that common genes between Sherlock-identified genes of Gene sets #1, #2, and #3 and MAGMA-identified genes on fake TB (Gene set #5). (**C**–**F**) Computer-based permutation analysis; (**C**) for the overlap between Gene set #1 and Gene set #2; (**D**) for the overlap between Gene set #1 and Gene set #3; (**E**) for the overlap between Gene set #1 and Gene set #4; (**F**) for the overlap between Gene set #1 and Gene set #5.

By performing MAGMA gene-level analysis of TB-based GWAS, we identified 1,017 genes were significantly or suggestively associated with TB (Gene set #4, MAGMA-based P ≤ 0.05; Supplementary Table 14). Among them, 128 genes have been documented to be associated with TB or at least one of other respiratory related traits or diseases in the database of GWAS Catalog (Supplementary Figure 16 and Supplementary Table 14). Compared with 3 independent Sherlock-identified gene sets, 18 of 26 common genes were significantly replicated by using MAGMA analysis (Figure 3A and Table 1). As a negative control, genes identified from MAGMA analysis on fake TB (Gene set #5) have obviously lower overlap with Sherlock-identified common genes than those with genes from MAGMA analysis on TB (Figure 3B and Table 1). In addition, we used the MAGMA tool to perform a pathway enrichment analysis based on the KEGG pathway resource. We found that 19 pathways showed significant or suggestive enrichment (P < 0.05). Of them, 15 pathways were enriched by genes identified from Sherlock analysis in the discovery stage (P < 0.05, Supplementary Table 15).

**Figure 4:**
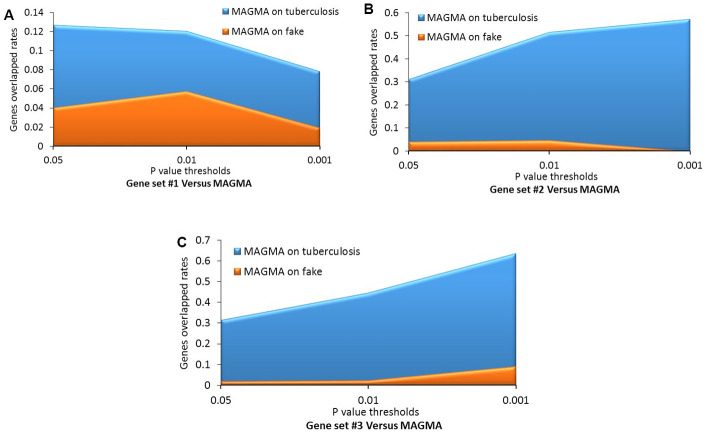
**Comparative analysis of genes identified from the Sherlock analysis with that from the MAGMA analysis of tuberculosis (TB) and fake TB.** (**A**) Gene set #1 versus MAGMA; (**B**) Gene set #2 versus MAGMA; (**C**) Gene set #3 versus MAGMA.

Consistently, by using permutation analyses, genes identified from the discovery stage (Gene set #1) were significantly higher overlapped with identified genes from Gene sets #2, #3, and #4 in the replication stage than that of 100,000 times of random selections (Permuted P = 0, 0, 0 separately; Figure 3C–3E). Furthermore, there was no difference in overlap between genes from Gene set #1 with genes from Gene set #5 and genes from random selections (Permuted P = 0.32; Figure 3F). Additionally, to further determine whether these identified TB-associated genes were due to genetic determinants rather than false discoveries, we compared the results from MAGMA analysis on TB (Gene set #4) and fake TB (Gene set #5) with significant genes identified from 3 times of independent Sherlock analyses (Gene sets #1, #2, and #3) at 3 distinct P value thresholds (i.e., P = 0.05, 0.01, or 0.001), respectively. Consistently, we found that the overlapped gene rates between Sherlock-identified genes and MAGMA-identified genes were remarkably higher than that with MAGMA analysis on fake TB across 3 different thresholds (Figure 4A–4C). Together, these results further confirm that our identified genes are potentially convincing candidate genes for TB.

### GGI network constructed by 26 highlighted TB-risk genes

**Figure 5:**
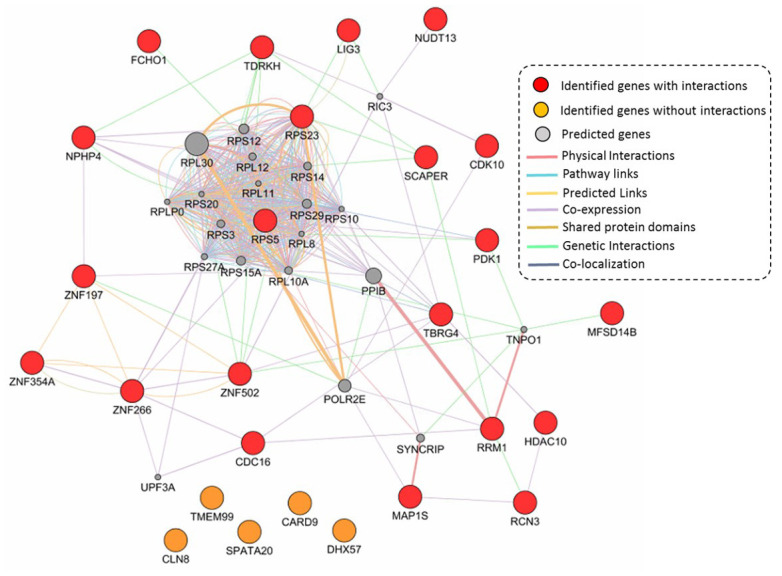
**Constructed GGI network by using identified 26 TB-associated genes.** These 21 identified genes with interactions are marked with red color, 5 identified genes without interactions are marked with orange color, and 20 predicted genes are colored with gray color.

Based on independent biological and technical replications, we highlighted 26 genes as important candidates conferring susceptibility to TB. Based on these 26 genes, we performed a GGI network enrichment analysis. Figure 5 demonstrates that most of these highlighted genes were highly connected with each other. The majority of interactions in the constructed network were depended on co-expression, which accounted for 71.52% of interactions (Supplementary Table 16 and Supplementary Figure 17). For example, the hub gene of *RPS5* had co-expression evidence with *NPHP4* and *PDK1*. Furthermore, the hub gene of *RPS23* showed a genetic interaction with *SCAPER*, as well as the *SCAPER* gene interacted with *TDRKH* based on evidence of genetic interactions. It should be noted that 5 TB-associated genes of *CLN8*, *TMEM99*, *CARD9*, *SPATA20*, and *DHX57* had no interactions with other genes in this constructed network (Figure 5).

### Differential gene expression analysis of these 26 highlighted genes

**Figure 6:**
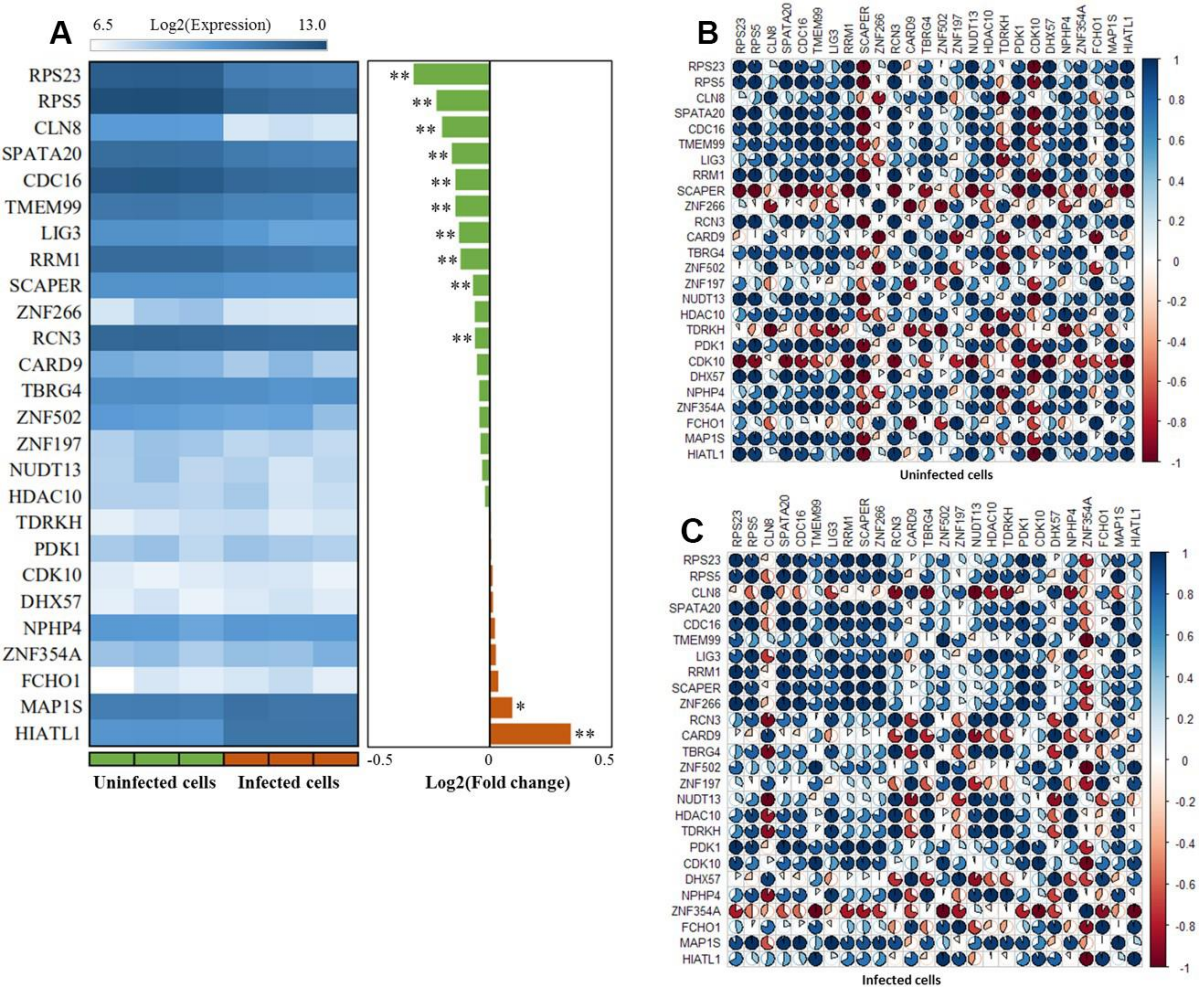
**The expression patterns of these 26 risk genes between infected cells and uninfected cells based on the GSE133803 dataset.** (**A**) Heatmap showing the expression levels of 26 risk genes between infected cells and uninfected cells; * represents the t-test P value < 0.05, ** represents the t-test P value < 0.01; (**B**) The co-expression patterns of 26 risk genes based on the Pearson correlation analysis in uninfected cells; (**C**) The co-expression patterns of 26 risk genes based on the Pearson correlation analysis in infected cells.

By utilizing the expression data of GSE133803, we performed a DGE analysis of these 26 highlighted genes and found 12 genes were significantly expressed between MTB-infected cells and controls (Figure 6A, Table 1, and Supplementary Table 17); for example, *CDC16* (P = 5.63 × 10^-4^), *RPS5* (P = 2.11 × 10^-4^), *HIATL1* (P = 1.83 × 10^-7^), and *RPS23* (P = 5.45 × 10^-5^). 2 genes of *CARD9* (P = 0.055) and *ZNF197* (P = 0.054) were identified to be suggestively significant (Supplementary Table 17). In light of most of interactions among genes were derived from co-expression (71.52%) in our GGI network analysis, we further conducted a Pearson correlation analysis to uncover whether the co-expression patterns of these highlighted genes altered or not between MTB-infected cells and uninfected cells. We detected that there was remarkable differences in co-expression patterns among 26 highlighted genes between MTB-infected cells and uninfected cells (Figure 6B, 6C and Supplementary Tables 18, 19). For example, the positive correlation coefficient of *RPS23* with *NUDT13* was decreased from 0.99 in uninfected cells to 0.34 in MTB-infected cells. Furthermore, the correlation coefficient between *RCN3* and *CLN8* was changed from 0.41 in uninfected cells to -0.92 in MTB-infected cells.

**Figure 7:**
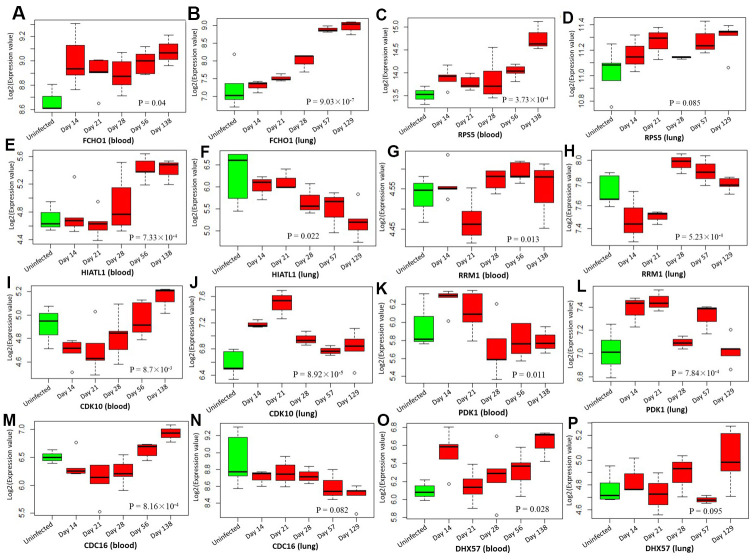
**Boxplots show the differential expression levels of tuberculosis-risk genes between uninfected mice and infected mice with 5 distinct time points based on two GSE1440943 (blood) and GSE1440944 (lung) datasets.** (**A**) *FCHO1* for blood; (**B**) *FCHO1* for lung; (**C**) *RPS5* for blood; (**D**) *RPS5* for lung; (**E**) *HIATL1* for blood; (**F**) *HIATL1* for lung; (**G**) *RRM1* for blood; (**H**) *RRM1* for lung; (**I**) *CDK10* for blood; (**J**) *CDK10* for lung; (**K**) *PDK1* for blood; (**L**) *PDK1* for lung; (**M**) *CDC16* for blood; (**N**) *CDC16* for lung; (**O**) *DHX57* for blood; (**P**) *DHX57* for lung. P values were generated by Anova test.

By analyzing the GSE1440943 dataset based on blood samples, 8 significant genes and 1 suggestive genes showed differential expressions between MTB-infected mice with 5 different time points and uninfected mice (Table 1, Figure 7 and Supplementary Figure 18). Furthermore, we analyzed the GSE1440944 dataset based on lung tissues and identified 11 significant genes and 3 suggestive genes have differential expressions between MTB-infected with 5 different time points and uninfected mice (Table 1, Figure 7 and Supplementary Figure 19). There existed a consistent finding of significant genes between both datasets (Table 1 and Figure 7). For example, 2 genes of *FCHO1* and *RPS5* showed significantly higher expression in MTB-infected mice at 5 time points than in uninfected mice in both blood (Figure 7A: Anova P = 0.04; and Figure 7C: Anova P = 3.73 × 10^-4^) and lung samples (Figure 7B: Anova P = 9.03 × 10^-7^ and Figure 7D: Anova P = 0.085). Consistently, by using the dataset of GSE139825 based on human alveolar macrophages, 7 significant genes (Anova P < 0.05; Supplementary Figure 20) and 4 suggestive genes (Anova P < 0.1; Supplementary Figure 21) showed differential expressions between TB group and control group. For example, *RPS5* (Anova P = 2.74 × 10^-4^) and *FCHO1* (Anova P = 7.12 × 10^-3^).

### Identification of risk eSNPs among these 26 highlighted TB-risk genes

For each highlighted gene, there were multiple eSNPs showing significant association with the expression of this gene and TB risk simultaneously (Supplementary Table 20). To name a few, with respect to the gene of *CDC16*, 2 cis-regulatory eSNPs of rs7987202 (P_eQTL_ = 4.70 × 10^-13^ and P_GWAS_ = 2.53 × 10^-3^) and rs9590408 (P_eQTL_ = 3.79 × 10^-49^ and P_GWAS_ = 2.02 × 10^-3^) and 1 trans-regulatory eSNPs of rs948182 (P_eQTL_ = 4.13 × 10^-6^ and P_GWAS_ = 2.01 × 10^-2^) were identified. 1 eSNP of rs3118766 (P_eQTL_ = 5.45 × 10^-7^ and P_GWAS_ = 7.32 × 10^-4^) has cis-regulatory effect on *HIATL1* gene. 3 eSNPs of rs2946863 (P_eQTL_ = 3.26 × 10^-7^ and P_GWAS_ = 6.42 × 10^-3^), rs2878342 (P_eQTL_ = 2.70 × 10^-12^ and P_GWAS_ = 3.82 × 10^-3^), rs3810194 (P_eQTL_ = 6.65 × 10^-6^ and P_GWAS_ = 1.43 × 10^-2^) have cis-regulatory functions on *RCN3* gene. Furthermore, with regard to *FCHO1* gene, 3 cis-eSNPs (rs4280376: P_eQTL_ = 1.95 × 10^-10^ and P_GWAS_ = 5.86 × 10^-2^, rs4808683: P_eQTL_ = 9.98 × 10^-15^ and P_GWAS_ = 3.39 × 10^-3^, rs8107550: P_eQTL_ = 2.85 × 10^-6^ and P_GWAS_ = 4.40 × 10^-3^) and 1 trans-eSNP (rs1058348: P_eQTL_ = 3.24 × 10^-7^ and P_GWAS_ = 2.78 × 10^-2^) were identified.

## DISCUSSION

TB is an infectious disease and remains a leading public health problem in developing world and an increasing threat in developed countries [1–3]. There were approximately one third of the world populations estimated to be infected with the TB pathogen, *Mycobacterium tuberculosis*, but only about 10% of infected individuals eventually become active TB patients [3], suggesting genetic heterogeneity potentially contribute differential susceptibility to infection. Consistently, host genetic factors having important roles in determining susceptibility to *Mycobacterium tuberculosis* are well-indicated by twin, family linkage, candidate gene analyses, and mouse models [6–8, 34, 35]. Hitherto, more than 10 GWASs on TB have been reported [17–25], and many TB-associated genetic loci have been identified and documented in the NHGRI GWAS Catalog [36]. Nevertheless, some identified genetic variants were hard to be replicated [37, 38], which could be attributed to the genetic heterogeneity of samples used, underpowered GWASs, or small effect sizes of variants. Lack of replications lead to these GWAS-identified SNPs have not translated into clinical practice so far. Thus, there exists a strong interest in improving our understanding of the pathophysiological mechanisms of genetic components on TB with the use of advanced genetics- and genomics-based methods.

For the method of GWAS, it has been widely used to identify genetic loci conveying risk to complex diseases [39]. With the use of GWAS, a growing and large number of SNPs have been documented to be of significant associations with hundreds of phenotypes [36, 40, 41]. However, due to the stringent correction for multiple testing of GWAS, many SNPs with small-to-moderate effects which not reach a genome-wide significance but have important functional roles were largely neglected. In light of many SNP-SNP pairs have highly LD accompanied with similar level of significance when calculate the P-values, thus to pinpoint the exact causative variants of these GWAS-identified associations is still a big challenge. Generally, a large proportion of identified risk SNPs were annotated into noncoding regions of genome in GWASs on complex diseases including TB [8, 26, 28], indicating these SNPs may influence the gene expression levels by cis- and/or trans-regulatory mechanisms to involve in TB risk. Considerable work on exploring the links between genetic variants and RNA expression is interested and warranted. For our current study, we conducted an integrative genomics analysis by combining multi-layers of omics data, including genomics, eQTL, RNA expression, eSNPs, and gene-gene interactions, to identify more susceptible SNPs, genes, and pathways implicated in the etiology of TB risk.

We first performed a Sherlock-based Bayesian analysis through incorporating a large-scale GWAS summary dataset on TB with a discovery eQTL dataset to identify susceptible genes and eSNPs. At this discovery stage, a number of 694 significant genes were identified to be associated with TB. Of note, we noticed that 49 genes of 694 significant genes have been documented to be associated with TB, lung-related or respiratory-related diseases in earlier studies. For example, 4 genes of *C2CD2* [20], *HLA-DRB6* [42], *LPCAT2* [43], and *HLA-DQB1* [42, 44] were associated with TB risk, and *RUNX* [45] showed association with asthma or allergic disease risk. In addition, *RUFY1*, *DEPDC7*, and *IRF4* were reported to be involved in lung cancer [46]. To validate the findings in the discovery stage, we conducted Sherlock analysis based on 2 independent eQTL datasets and found that there were 26 genes significantly replicated. Of note, 1 common gene of *CARD9* was previously identified to be associated with lung function (FVC) [47, 48]. Additionally, based on these significantly identified genes in both discovery and replication stage, we found 10 important biological pathways implicated in TB risk, providing a mechanistic clue for performing molecular studies for TB. Based on multiple layers of protein and genomics evidence deposited in public databases, we found these 26 genes were highly connective with each other in the constructed network, indicating these genes jointly impact on TB susceptibility. Noteworthy, all these 26 genes encompassed at least one eSNPs which are significantly associated with both expression of gene and TB risk. Meanwhile, we also utilized MAGMA analysis of GWAS on TB as an independent technical validation. Interestingly, 18 of 26 (69.23%) common genes were significantly replicated in MAGMA analysis.

Since there existed a high proportion of co-expression links among these 26 genes in our constructed network, we inferred that the co-expression patterns might be changed according to the different disease status of TB. In line with our speculation, the co-expression patterns among 26 genes were prominently altered between MTB-infected and uninfected cells. By performing the DGE analysis based on 4 independent expression datasets, we found that 21 of 26 genes had significantly differential expressions between TB group and control group in mesenchymal stem cells, mice blood and lung tissues, as well as human alveolar macrophages; such as, *CDC16*, *HIATL1*, *RCN3*, *FCHO1*, and *RPS5*. These results are consistent with the primary assumption of the Sherlock-based Bayesian inference algorithm that aberrant expression of genes are more likely to convey risk to complex diseases [29]. For the original GWAS reported by Canela-Xandri and coworkers [49], there was no SNP reaching genome-wide significance to be associated with TB. Due to the strict genome-wide significance threshold applied by the GWAS, numerous susceptible genes and SNPs with small-to-moderate effects on TB being neglected. As the method of reported studies [50–53], based on the two-stage designed integrative genomics analysis, we highlighted 26 genes with multiple eSNPs as important candidates for revealing the pathogenesis of TB risk.

The protein of CDC16, encoded by the highlighted gene of *CDC16*, is a protein ubiquitin ligase and is one of components of the multiprotein APC complex. CDC16 has been reported as a binding partner of chitooligosaccharide deacetylase homolog (YDJC) in breast cancer cells [54]. Overexpression of CDC16 enhanced the ubiquitination of YDJC in an orthotopic mouse model [55]. Kim and coworkers reported that suppression of YDJC or boosting of CDC16 interaction with YDJC might be implicated in the progression of lung cancer [56]. Previous studies have reported that TB is considered as a potential risk factor for the development of lung cancer [57, 58]. In our current analysis, there were 3 eSNPs (rs7987202, rs9590408, and rs948182) with cis- or trans-regulatory effects in *CDC16* gene identified to be associated with TB risk. As for the highlighted gene of *RCN3*, it encodes reticulocalbin 3 (Rcn3), which is an endoplasmic reticulum lumen protein mapped in the secretory pathway. Jin and colleagues [59] showed that Rcn3 protein has an indispensable physiological role in the maturation of perinatal lung and neonatal respiratory adaption by using an Rcn3 knockout mouse model. Furthermore, they demonstrated that upregulated expression of Rcn3 in maturating alveolar epithelial type II cells (AECIIs) seems to have a contribution to the survival and wound healing of AECIIs, indicating Rcn3 has a critical part in mediating pulmonary injury remodeling [60]. Hou and coworkers [61] suggested that there is a potential association between the depletion of Rcn3 protein and development of non-small cell lung cancer. We noticed 3 eSNPs of rs2946863, rs2878342, and rs3810194 in *RCN3* were associated with TB risk in our integrative genomics analysis.

Some limitations of our current analysis need to comment. Although we employed multiple omics datasets, there were other datasets missed. For example, in our current study, gene expression datasets were mainly based on blood samples. Only two datasets of eQTL Dataset #5 and GSE1449044 were derived from mice lung tissue. More molecular studies for exploring the functions of genes identified from our current analysis are warrant to assess tissues that could be more related to the etiology of TB, for example, human lung tissue. Furthermore, due to the heterogeneity of different datasets, we applied different correction methods for multiple testing at each individual dataset; such as, simulated P value < 0.05 for Sherlock Bayesian analysis, false discovery rate (FDR) < 0.05 for pathway enrichment analysis, and empirical P value < 0.05 for 100,000 times of *in silico* permutation analysis. Additionally, association signals of eSNPs from current integrative genomics analysis were obtained in the European population. We did not determine whether the associations exist in other ancestries. Future studies are warrant to evaluate the regulatory effects of eSNPs using genotype and expression data from other ethnic populations. In addition, although a total of 452,264 samples were included for our genomics analysis, it should be noted that our chosen controls might contain persons have latent infection or they are the susceptible host that have never been exposed to TB, which might result in the power loss for genome-wide association analysis of this dataset.

In conclusion, in the present study, we conducted a systematically integrative genomics analysis to identify TB-associated risk SNPs, susceptible genes, and biological pathways. By incorporating GWAS summary statistics with eQTL data, we offered a reasonable explanation of the regulatory functions of intronic SNPs for TB. With the use of detailed topology data on gene-gene and gene-drug information, we highlighted 26 candidate genes for TB susceptibility, which were difficult to be identified by any single GWAS. More molecular experiments are warranted to be performed for identification of the biological mechanisms of these prioritized genes implicated in the aetiology of developing TB.

## MATERIALS AND METHODS

### Sherlock-based integrative genomics analysis

To exploit whether abnormal expression of gene with susceptible SNPs implicated in the etiology of TB risk, we performed a Sherlock-based integrative genomics analysis to integrate GWAS summary-based SNP information with eQTL [29]. The Sherlock integrative analysis based on a Bayesian algorithm is intended to cluster multiple lower-confidence SNPs from GWAS with expression QTL data to reveal authentic susceptible genes involved in complex diseases. In our Sherlock analysis, SNP rs IDs and P values extracted from GWAS summary-level statistics were utilized as an input list. The definition of expression-associated SNPs (i.e., eSNPs) are that SNPs show significant associations with TB risk and meanwhile have cis- or trans-regulatory effects on expression levels of interested genes. There exists 3 potential scenarios: 1) A positive score would be recorded based on a specific eSNP shows a significant association with TB; 2) A negative score would be recorded based on a specific eSNP shows a non-significant association with TB; 3) No score would be recorded based on an SNP was not eSNP but shows a significant association with TB. The summed score of a specific gene was based on the number of eSNPs with integrative evidence from both GWAS and eQTL data. The logarithm of the Bayes Factor (LBF) is generated as a crucial indicator to predict TB-associated functionally-important genes. The significance of Sherlock Bayesian algorithm is assessed by using a simulation analysis, and P < 0.05 is considered to be significant.

### Dataset #1 for GWAS summary statistics on TB

The Dataset #1, the large-scale GWAS summary dataset on TB [49], was downloaded from the UK-Biobank database (Fields: 20002; Field codes: 1440). There were 452,264 subjects with 2,219 patients included in the GWAS. The Affymetrix UK BiLEVE Axiom array and the Affymetrix UK Biobank Axiom array were utilized for obtaining the genotypes of all subjects. There were 62,394 genotyped variants passed quality control. Moreover, based on the UK10K [62], 1,000 Genome [63], and Haplotype Reference Consortium [64] projects as genomics references, all genotyped variants were used for imputation to extend more variants. In the current investigation, we defined two filtering criteria for choosing high quality variants: 1) if variants are genotyped, these variants with minor allele frequency (MAF) > 10^-4^ are included; 2) if variants are imputed, these variants with MAF > 10^-4^ and imputation score > 0.9 are included. After strictly filtering, a number of 13,805,935 SNPs are qualified for subsequent genomics integrative analysis.

### Dataset #2 for GWAS dataset on fake TB

To ensure identified TB-risk genes were due to genetic determinants instead of random events, we constructed a fake TB-based GWAS through using a reported GWAS dataset (N = 3,960) [65]. We used the function of RANDBETWEEN in the Microsoft Excel to randomly generate and assign the phenotype of TB or control to these 3,960 individuals. In view of there is no true genetic effect of fake TB, the sample size of constructed GWAS is not a big issue. Thus, we used this constructed GWAS dataset as a negative control to re-perform genomics analysis by using the software of PLINK v1.07 based on the addictive genetic model.

### Dataset #3 for eQTL dataset reported by Zeller and coworkers

Here we downloaded the monocyte eQTL data reported by Zeller and colleagues [66], which is used as a discovery dataset for the Sherlock Bayesian genomics analysis. For this eQTL dataset, 1,490 subjects with DNA and RNA samples were enrolled from the Gutenberg Heart Study (GHS). The Affymetrix Genome-wide Human SNP Array 6.0 was utilized to obtain the genotypes of subjects, and the Illumina HT-12 v3 BeadChip was utilized to obtain RNA expression abundances. After stringently excluding, a number of 675,350 SNPs and 12,808 genes were qualified for eQTL analysis and subsequent Sherlock analysis. For more detailed characteristics on this dataset, please refers to the original study [66].

### Datasets #4 and #5 for eQTL datasets from the GTEx database

Furthermore, we used two eQTL datasets on whole blood (Dataset #4; N = 369) and lung tissue (Dataset #5; N = 383) from the resource of Genotype-Tissue Expression project (GTEx v7) as an independent replication to conduct Sherlock analysis with same parameters. As for the resource of GTEx [67–69], nearly 1,000 subjects with 54 non-diseased tissues were utilized to collect samples for whole genome sequencing, whole exome sequencing, and RNA sequencing, which can be used for integrative genomics analysis to explore the relationship between genetic variants and expression levels of interested genes across multiple tissues. Multi-layers of omics data including gene expression and QTL data can be obtained through the GTEx Portal (https://www.gtexportal.org/home/).

### Gene-based analysis by using MAGMA tool

To further replicate the findings identified from the Sherlock analysis, we conducted a gene-based analysis of GWAS on TB by applying the Multi-marker Analysis of GenoMic Annotation (MAGMA) [70]. Here, we used GWAS-relevant SNP rs IDs and SNP P values as an input list for MAGMA analysis. To improve the mapping of SNPs across different files and reference data, we used the SNP synonym file encompassing lists of synonymous SNP rs IDs that refer to the same SNP on the basis of the resource of dbSNP database release 151. By using multiple regression method, we attempted to discover multi-variant aggregated genetic effects by incorporating SNP-SNP linkage disequilibrium (LD) information, which is reference to the 1,000 Genomes European Panel Phase 3. The definition of the SNP set of each gene is that the SNP located in the gene body or within extended +/-20 kb downstream or upstream of the gene, and the locations of SNPs are based on the Human Genome Build 37. In addition, based on the KEGG pathway resource, we used the MGMA tool to conduct a pathway-based enrichment analysis.

### *In silico* permutation analysis

By using the Sherlock Bayesian and MAGMA analysis, 5 gene sets were identified to be associated with TB risk; namely Gene set #1 from discovery stage (Dataset #3), Gene set #2 from replication stage (Dataset #4), Gene set #3 from replication stage (Dataset #5), Gene set #4 from MAGMA analysis on TB (Dataset #1), and Gene set #5 from MAGMA analysis on fake TB (Dataset #2). Based on these 5 gene sets, we carried out serial *in silico* permutation analyses with 100,000 times of random trial [71]. In first step of this permutation analysis, the number of overlapped genes between Gene set #1 with other gene sets (*N*_observation_) were counted separately. Second, the background genes of each gene set was treated as a gene pool, which could be used for random selections. The number of background genes (N _total_) were 5,786, 7,452, 18,318, and 17,565 for Gene sets #2, #3, #4, and #5, respectively. By randomly picking the same number as the significant genes in Gene sets #2, #3, #4, and #5 from background genes (N _total_) respectively, via 100,000 times of repeat, we calibrated the count of genes overlapped with these significant genes of Gene set #1(*N*_random_). Finally, we calculated the number of times *N*_random_ ≤ *N*_observation_ and divided by 100,000 to obtain an empirical permuted P value. P value less than 0.05 is considered to be of significance. The density plot of each analysis was generated by using the R platform.

### Functional enrichment analysis by using KOBAS tool

We carried out functional enrichment analyses with the use of the web-access tool of KOBAS version 3.0 [72]. The tool of KOBAS (http://kobas.cbi.pku.edu.cn/kobas3), which is depended on the machine learning-based called Combined Gene set analysis incorporating Prioritization and Sensitivity (CGPS) [73], is designed to analyze protein or gene functional annotation and functional gene set enrichment. With respect to gene set enrichment analysis, the method of KOBAS can accept either gene list or gene expression data as a submitted file. In our current analysis, we used identified TB-associated genes from 3 times of Sherlock analyses (i.e., Gene sets #1, #2, and #3) as 3 lists of submitted genes for the KOBAS tool to calculate significantly enriched gene sets, including gene set related name, enrichment score, raw P values and corrected P values. There were 3 types of databases used in our analyses: 1) Biological pathways: Reactome pathway, KEGG pathway, PANTHER pathway, and BioCyc pathway; 2) Gene Ontology (GO) terms; 3) Diseases: OMIM, NHGRI GWAS Catalog, and KEGG disease. The statistical significance is corrected by using the method of Benjamini-Hochberg false discovery rate (FDR) correction.

### GeneMANIA-based GGI network analysis of risk genes

We used the bioinformatics tool of GeneMANIA [74] to conduct a gene-gene interaction (GGI) network-based analysis for identifying collective interaction patterns of the identified TB-associated genes and predicted genes with similar functions or co-expressions. We used these highlighted risk genes to query the large database of documented genomics and proteomics data. By using a guilt-by-association approach, the GeneMANIA tool based on multi-layers of supportive evidence including co-expression links, shared protein domains, genetic interactions, pathway links, co-localization, physical interactions, and predicted links, is designed to quickly and effectively predict the molecular functions and biological interactions of submitted genes. The GGI network is visualized by using the Cytoscape network visualization and analysis platform [75].

### Differential expression patterns of identified genes

To determine whether abnormal alterations in RNA expression levels of highlighted TB-risk genes, we downloaded 4 independent gene expression datasets from the database of the NCBI’s Gene Expression Omnibus (GEO). The accession numbers of 4 expression datasets were GSE133803, GSE140943, GSE140944, and GSE139825. For GSE133803, the dataset was designed to analyze the mesenchymal stem cell gene expression level upon *Mycobacterium tuberculosis* (MTB) infection. RNA samples were obtained from MTB-infected mesenchymal stem cells (N = 3) and compared with that of uninfected mesenchymal stem cells (N = 3). The Illumina Human HT-12 V4.0 expression BeadChip was used to obtain the genome-wide gene expression profiles for all samples.

As for two datasets of GSE1440943 and GSE1440944, they were designed to characterize global transcriptional responses to MTB infection in different mouse models. The samples of GSE1440943 were based on blood samples obtained from BALB mice infected with low dose of MTB H37Rv, collected at 4 distinct time points Day 14 (N = 5), Day 21 (N = 5), Day 56 (N = 5), and Day 138 (N = 3) after MTB infection and uninfected control mice (N = 3). Similarly, the samples of GSE1449044 were based on lung tissues obtained from BALB mice infected with low dose of MTB H37Rv, collected at 4 distinct time points Day 14 (N = 3), Day 21 (N = 3), Day 56 (N = 3), and Day 129 (N = 5) after MTB infection and uninfected controls (N = 5). The genome-wide gene expression signatures of both GSE1440943 and GSE1440944 were assessed by using the Illumina MouseWG-6 v2.0 expression BeadChip. With regard to the dataset of GSE139825, it was designed to explore the response to infection with MTB by human extrapulmonary macrophages. Total RNA samples (N = 26) were obtained from alveolar macrophages from TB patients infected with clinical isolates of MTB to compared to alveolar macrophages from control samples. The Illumina HumanHT-12 V4.0 expression beadchip was used to evaluate the genome-wide transcriptional abundance.

### Statistical analysis of RNA expression data from GEO database

With regard to GSE133803 dataset, we conducted a differential gene expression (DGE) analysis. The Student’s t-test is used to assess the significant differences between MTB-infected cells and uninfected cells. Based on the Pearson correlation analysis, we used the *Corrplot* package in R platform to analyze and visualize the co-expression patterns among these highlighted TB-associated genes in the dataset of GSE133803. For both GSE1440943 and GSE1440944, the ANOVA test was used to compare the statistically significant differences between MTB-infected mice and uninfected mice at 4 distinct time points. Furthermore, for GSE139825, the ANOVA test was applied to assess the significant difference among different groups. The Rscript for this analysis was uploaded into the public github website (https://github.com/mayunlong89/TB/blob/master/Anova_test.R).

## Supplementary Material

Supplementary Figures

Supplementary Table 1

Supplementary Table 2

Supplementary Table 3

Supplementary Table 4

Supplementary Table 5

Supplementary Table 6

Supplementary Tables 7, 8, 9, 10, 11, 12 and 13

Supplementary Table 14

Supplementary Tables 15, 16 and 17

Supplementary Table 18

Supplementary Table 19

Supplementary Table 20
