# Supplementary material for: Integrative genomics analysis identifies promising SNPs and genes implicated in tuberculosis risk based on multiple omics datasets
Source: Aging (Albany NY). 2020 Oct 13;12(19):19173–220. doi: 10.18632/aging.103744 (PMC7732298; doi:10.18632/aging.103744)
Supplement: Supplementary Figures [file aging-12-103744-s001..pdf]

SUPPLEMENTARY FIGURES

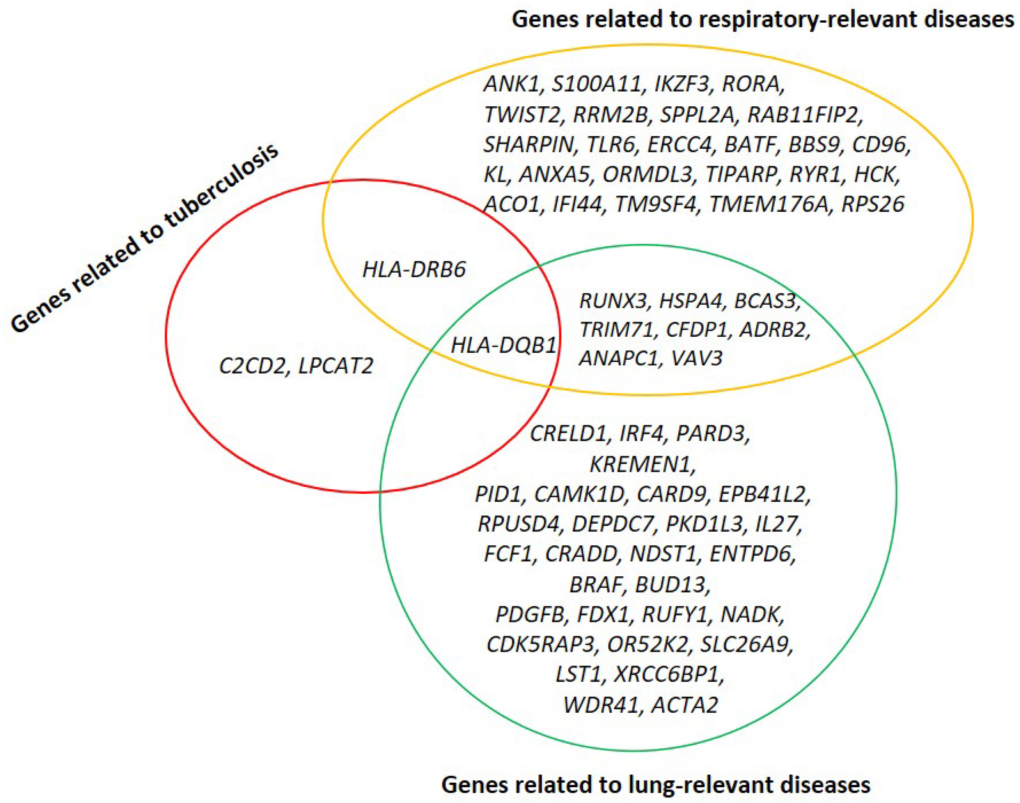

Supplementary Figure 1. Previous studies provide supportive evidence of these Sherlock-identified genes in the discovery stage.

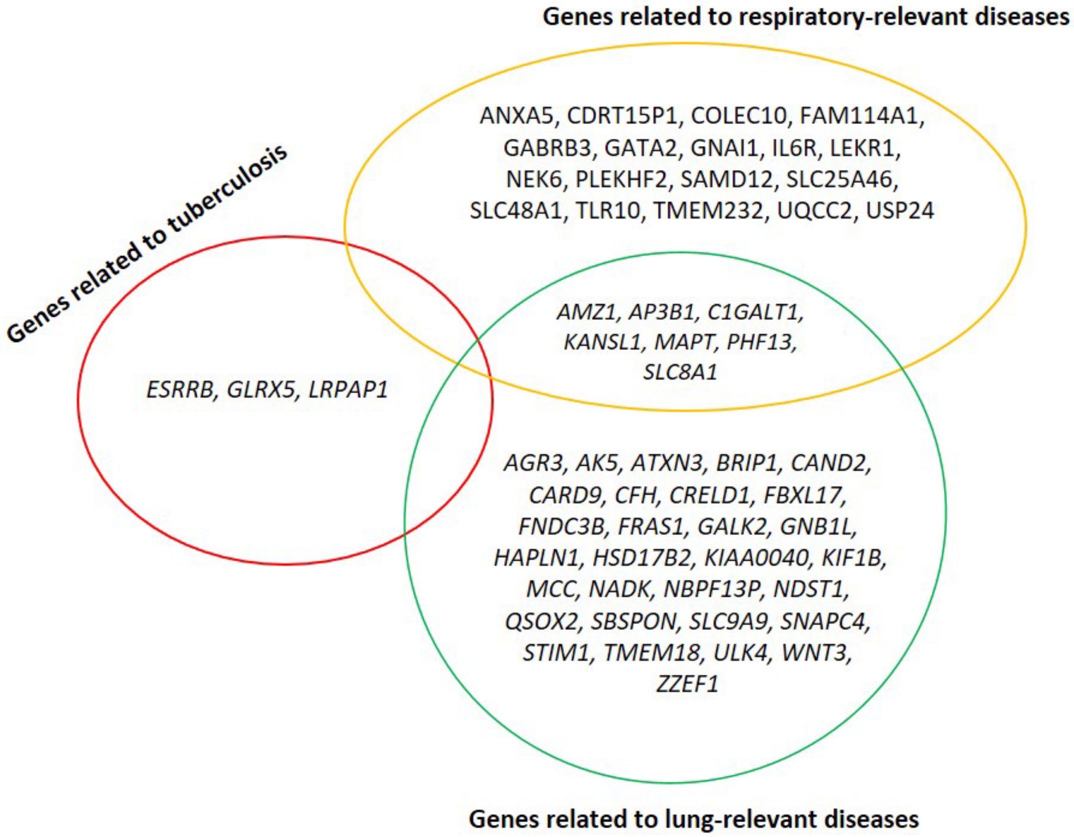

Supplementary Figure 2. Previous studies provide supportive evidence of these Sherlock-identified genes in the replication stage (based on both Dataset #4 and #5).

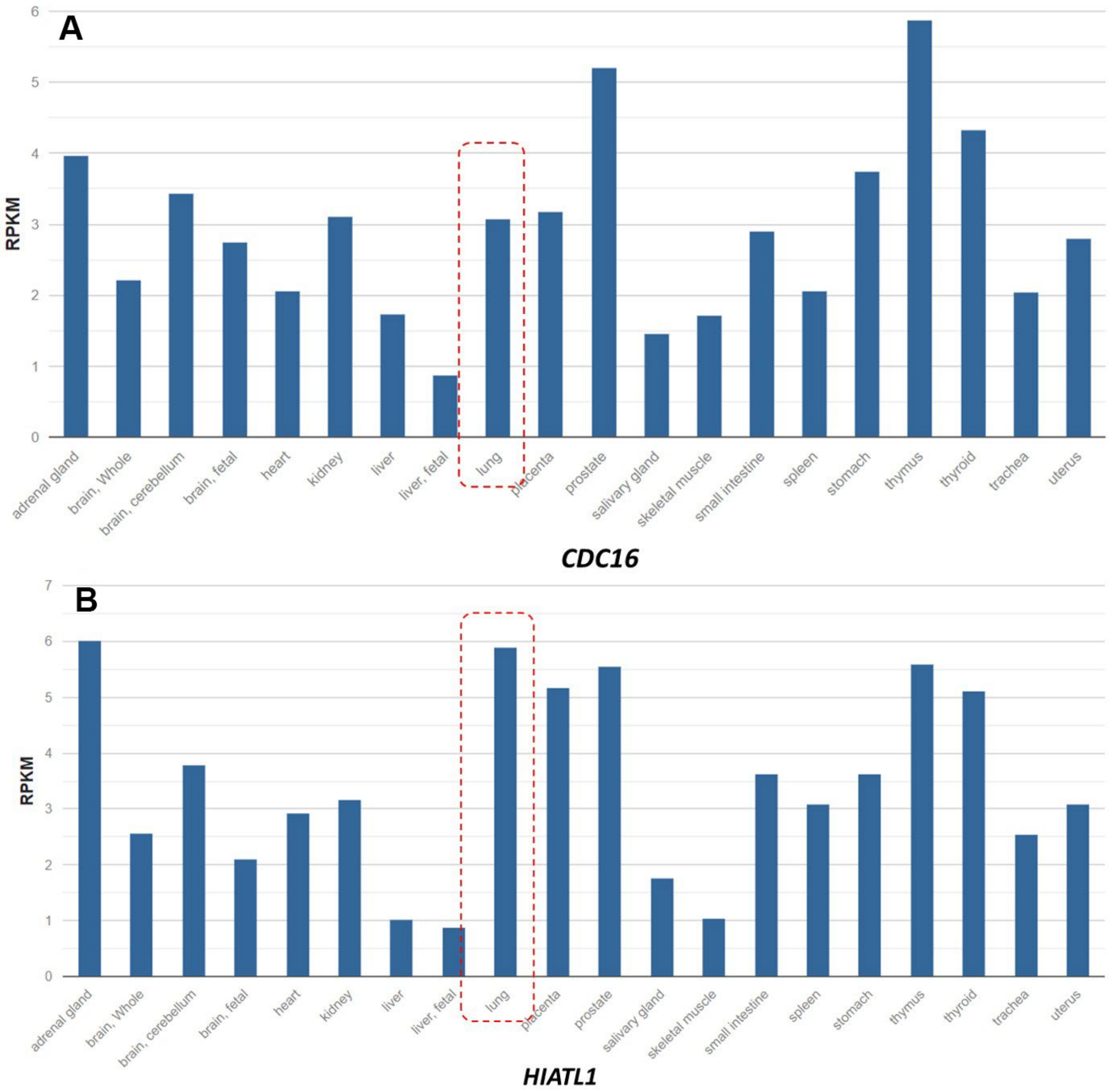

Supplementary Figure 3. Expression abundance of *CDC16* and *HIATL1* based on RNA sequencing from 20 human tissues. The expression data were obtained from BioProject (Accession No. PRJNA280600). Expression values are shown in Reads Per Kilobase per Million mapped reads (RPKM).

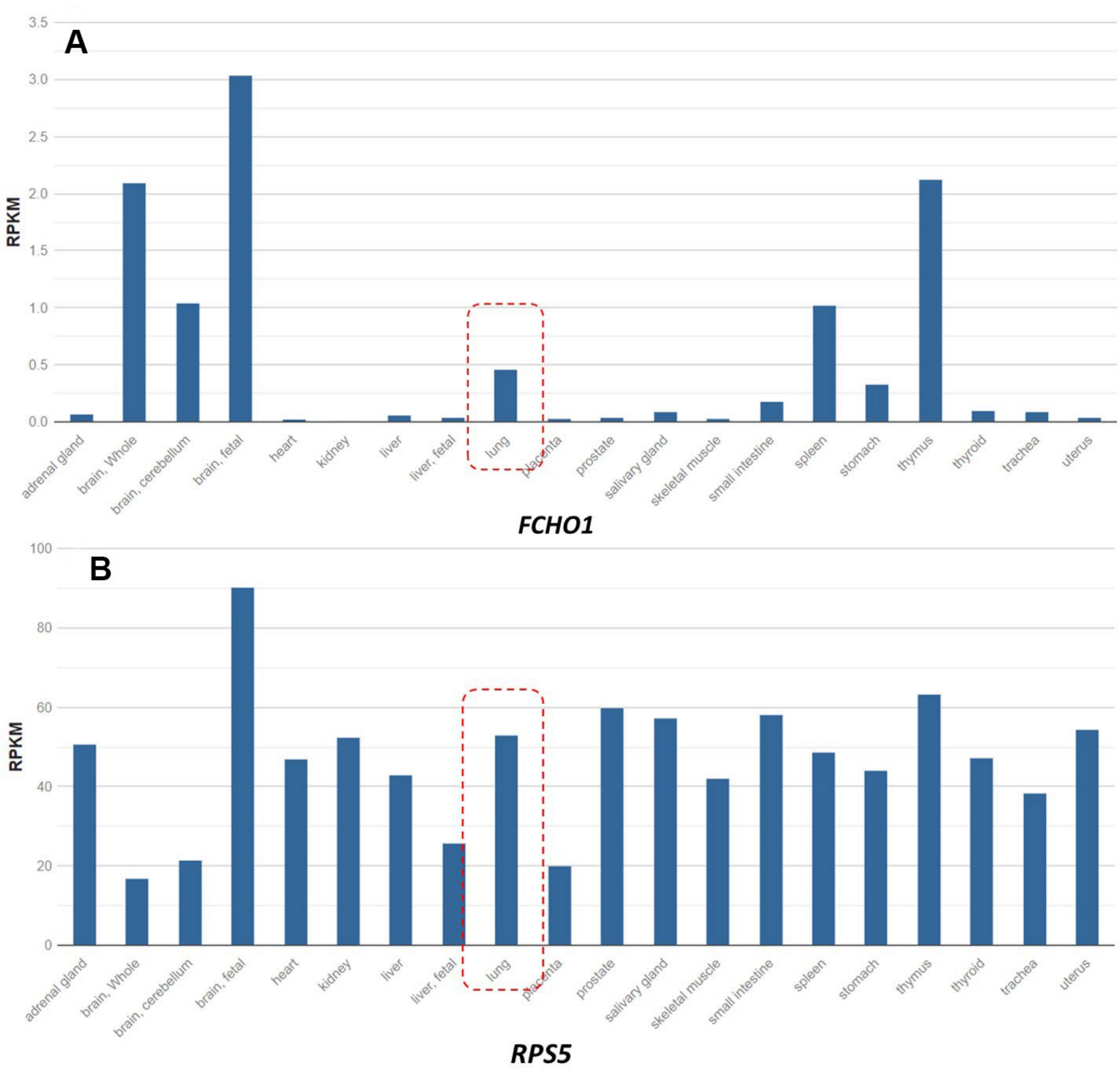

Supplementary Figure 4. Expression abundance of *FCHO1* and *RPS5* based on RNA sequencing from 20 human tissues. The expression data were obtained from BioProject (Accession No. PRJNA280600). Expression values are shown in Reads Per Kilobase per Million mapped reads (RPKM).

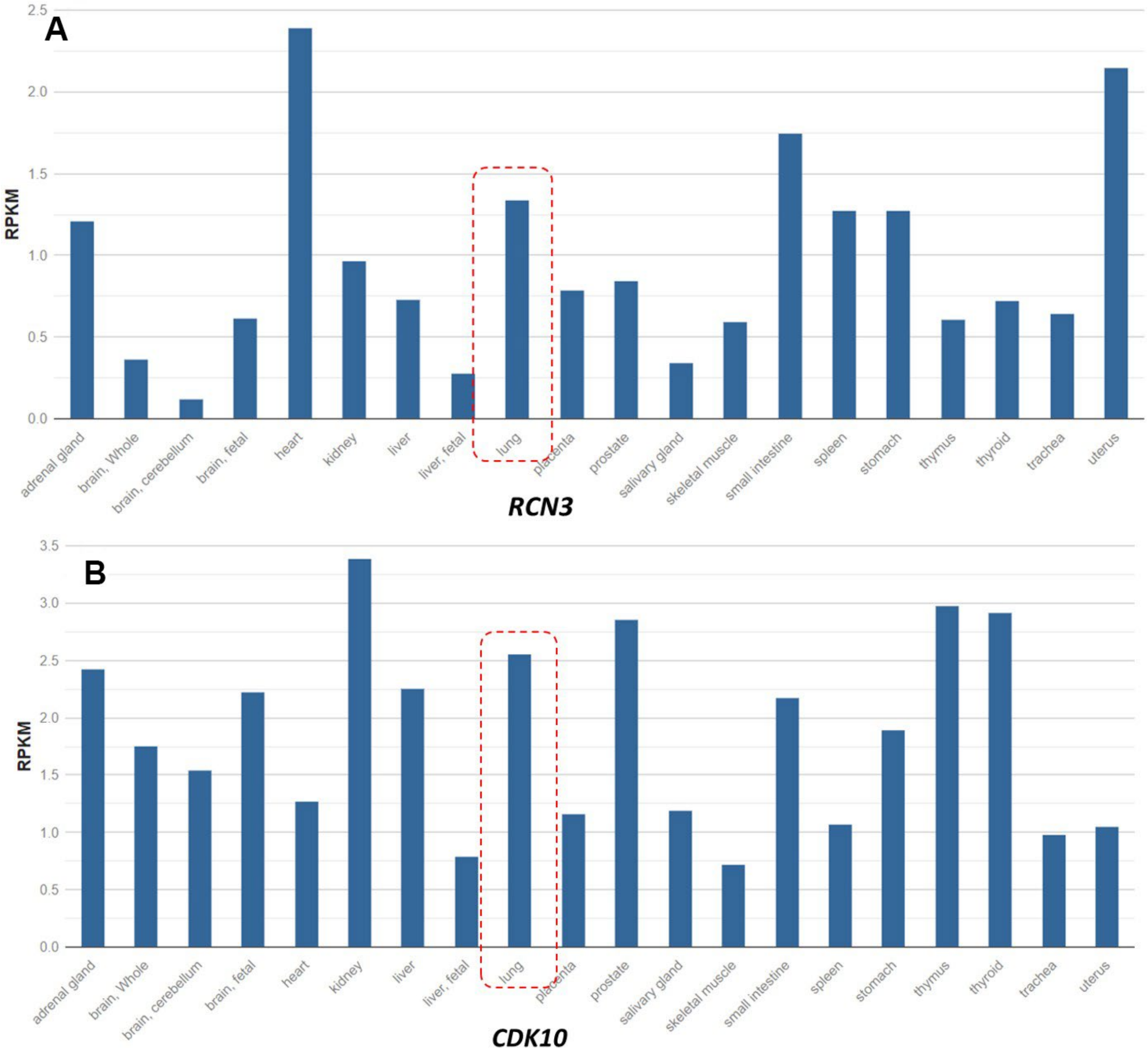

Supplementary Figure 5. Expression abundance of *RCN3* and *CDK10* based on RNA sequencing from 20 human tissues. The expression data were obtained from BioProject (Accession No. PRJNA280600). Expression values are shown in Reads Per Kilobase per Million mapped reads (RPKM).

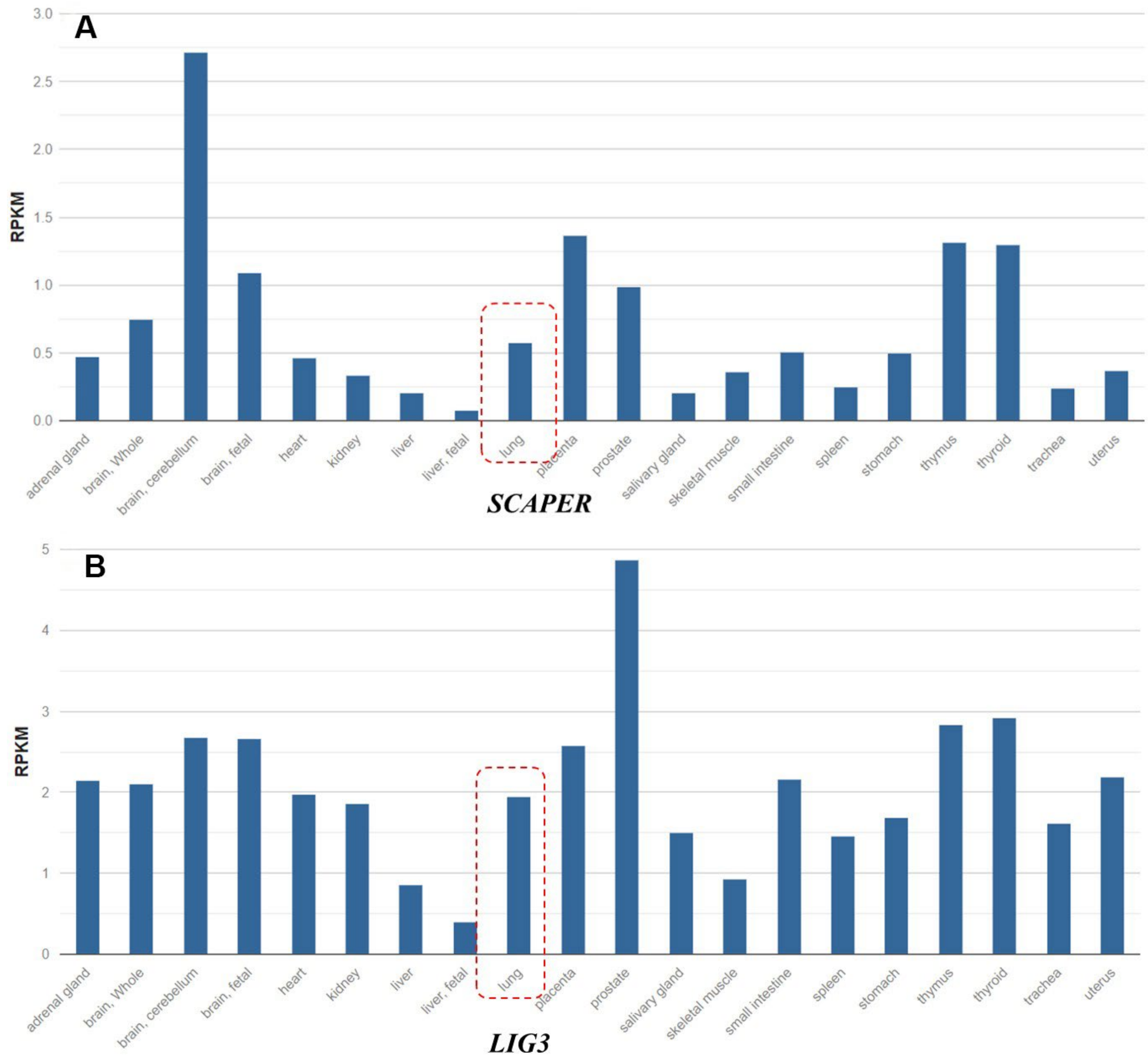

Supplementary Figure 6. Expression abundance of *SCAPER* and *LIG3* based on RNA sequencing from 20 human tissues. The expression data were obtained from BioProject (Accession No. PRJNA280600). Expression values are shown in Reads Per Kilobase per Million mapped reads (RPKM).

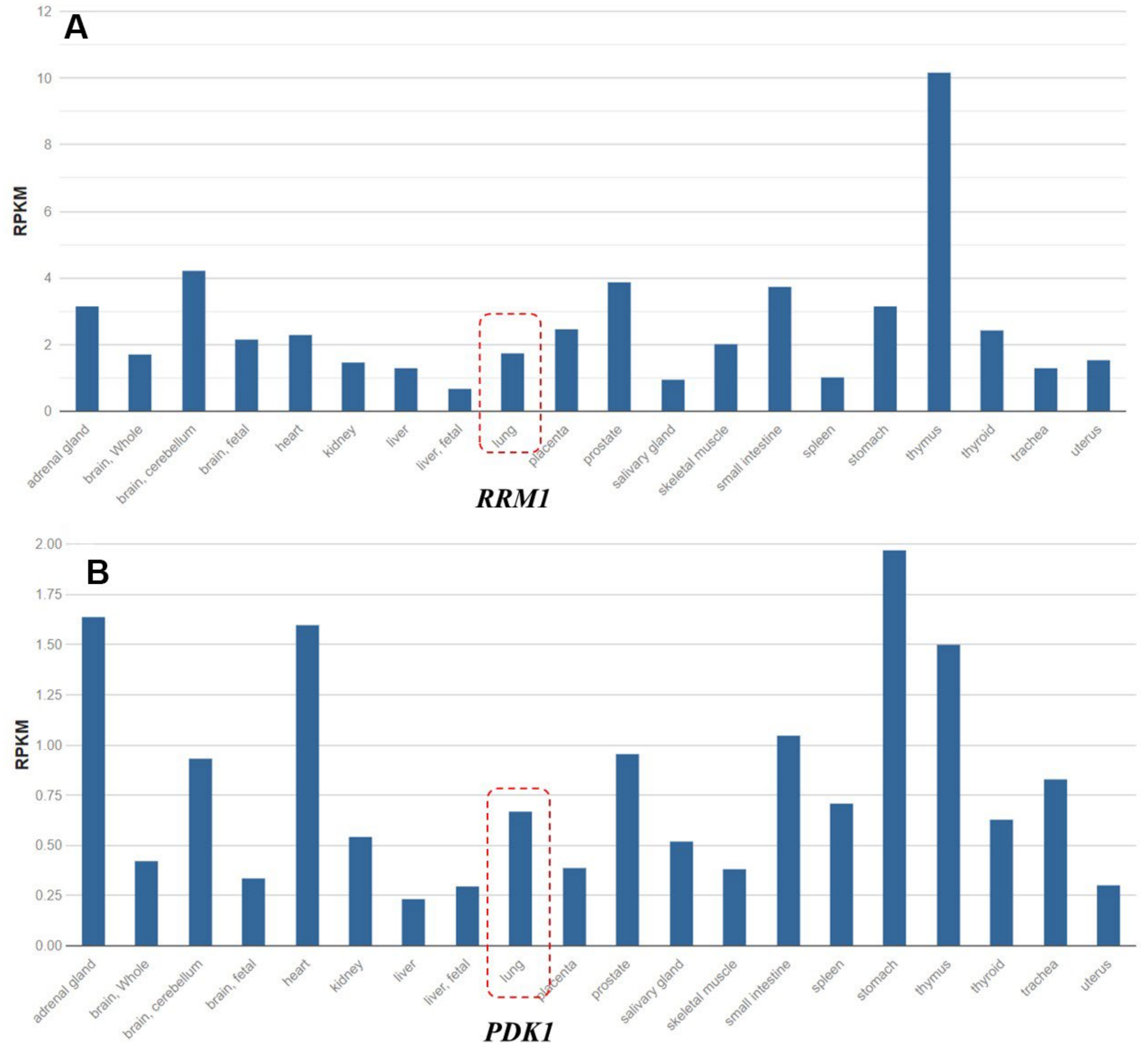

Supplementary Figure 7. Expression abundance of *RRM1* and *PDK1* based on RNA sequencing from 20 human tissues. The expression data were obtained from BioProject (Accession No. PRJNA280600). Expression values are shown in Reads Per Kilobase per Million mapped reads (RPKM).

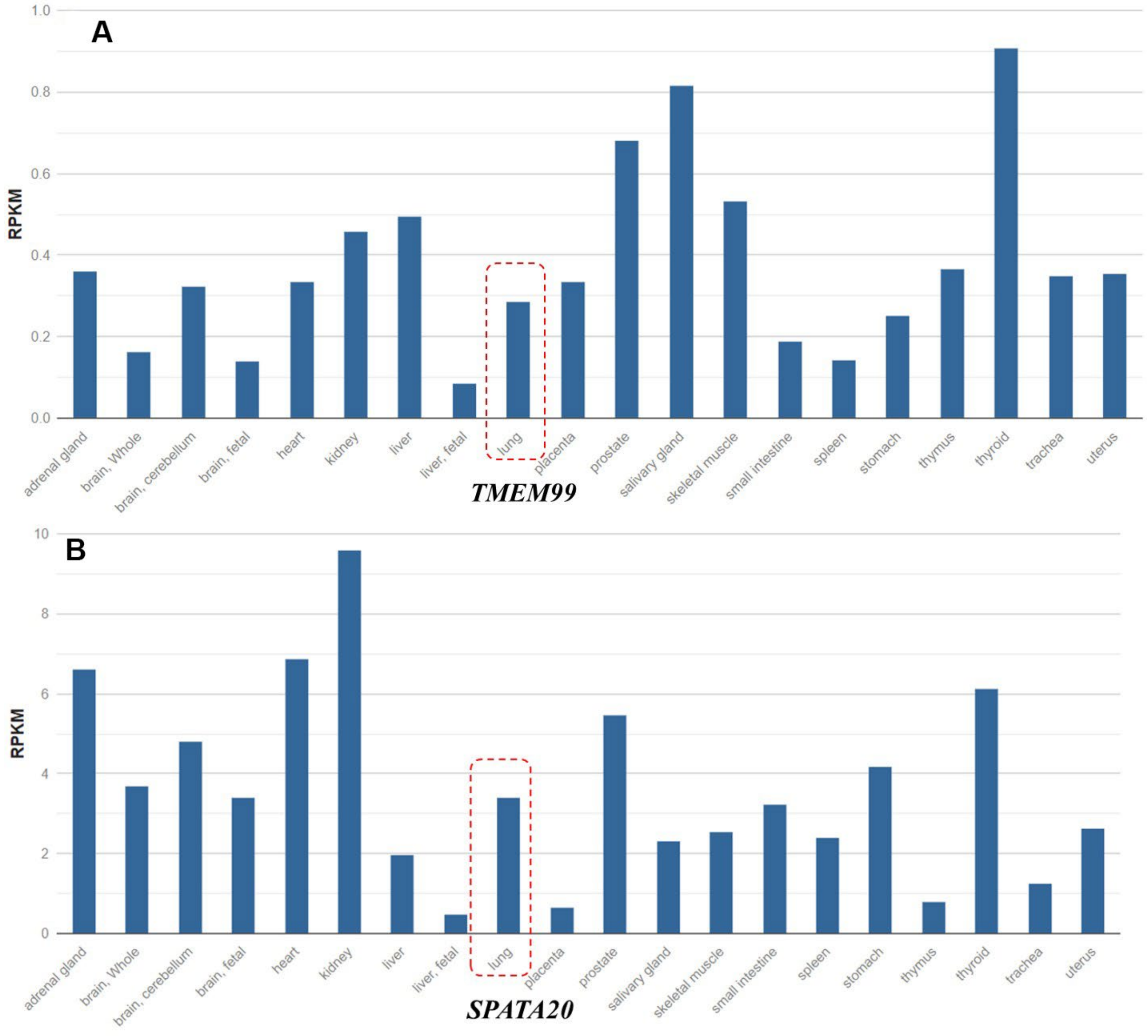

Supplementary Figure 8. Expression abundance of *TMEM99* and *SPATA20* based on RNA sequencing from 20 human tissues. The expression data were obtained from BioProject (Accession No. PRJNA280600). Expression values are shown in Reads Per Kilobase per Million mapped reads (RPKM).

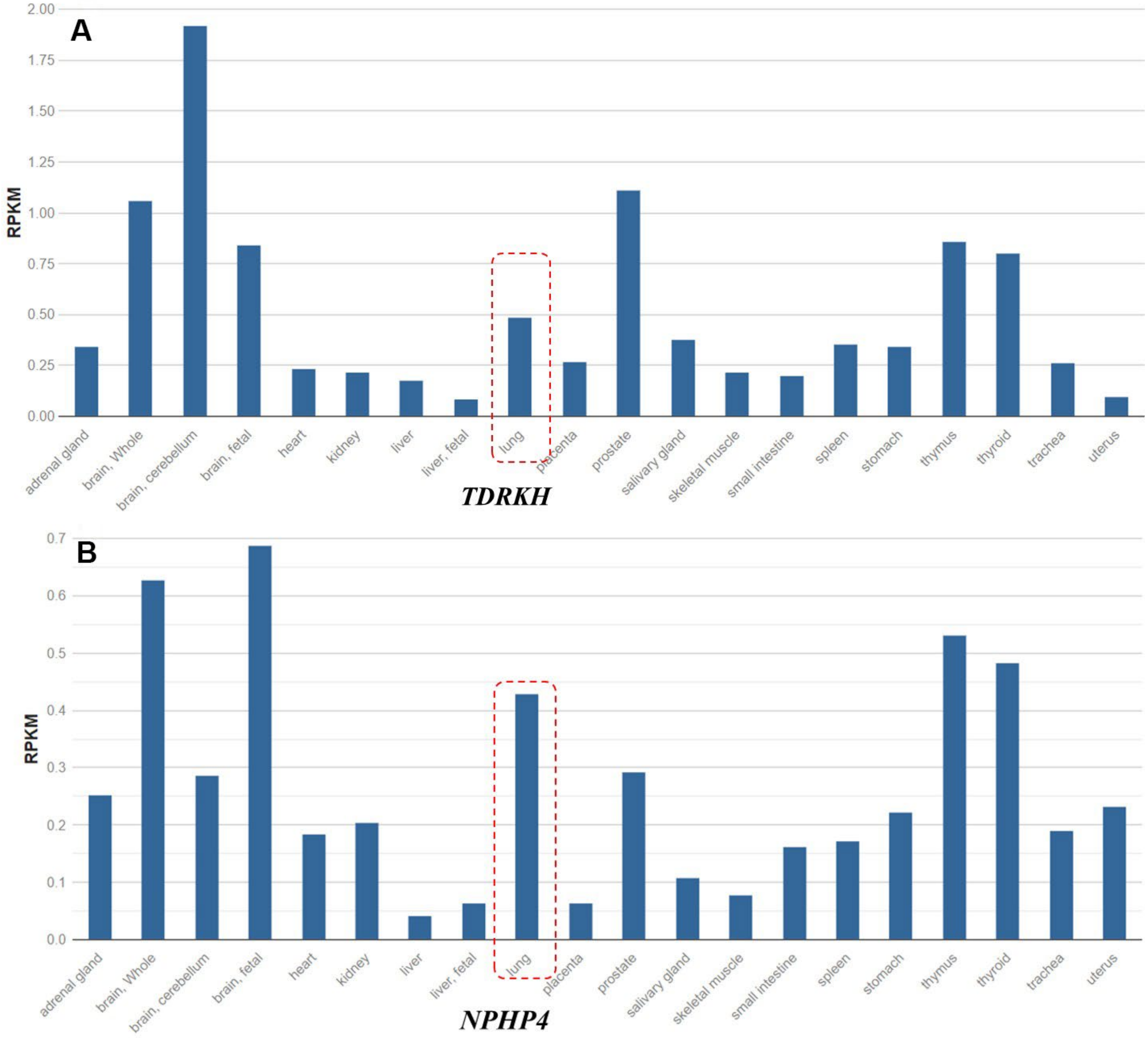

Supplementary Figure 9. Expression abundance of *TDRKH* and *NPHP4* based on RNA sequencing from 20 human tissues. The expression data were obtained from BioProject (Accession No. PRJNA280600). Expression values are shown in Reads Per Kilobase per Million mapped reads (RPKM).

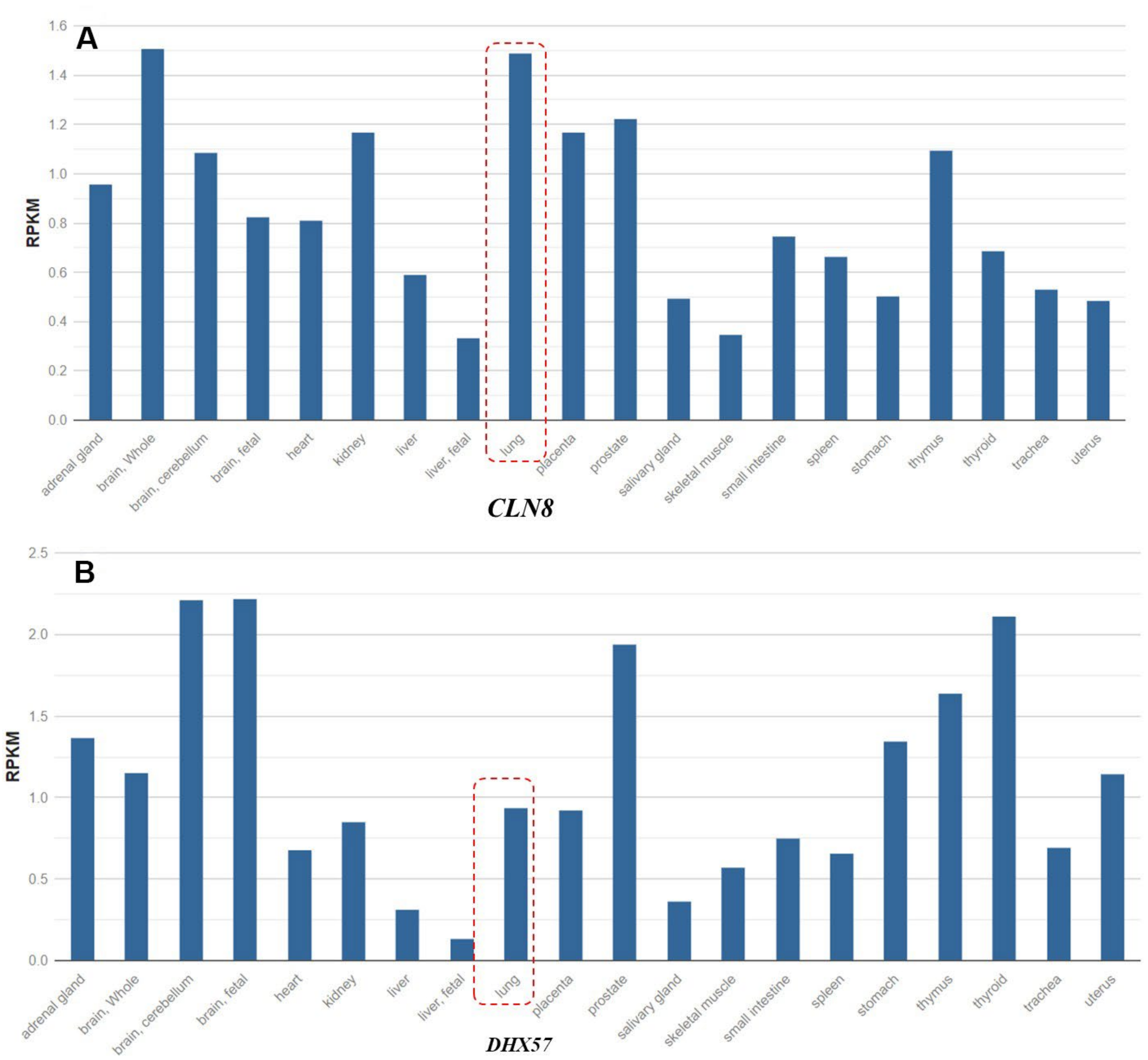

Supplementary Figure 10. Expression abundance of *CLN8* and *DHX57* based on RNA sequencing from 20 human tissues. The expression data were obtained from BioProject (Accession No. PRJNA280600). Expression values are shown in Reads Per Kilobase per Million mapped reads (RPKM).

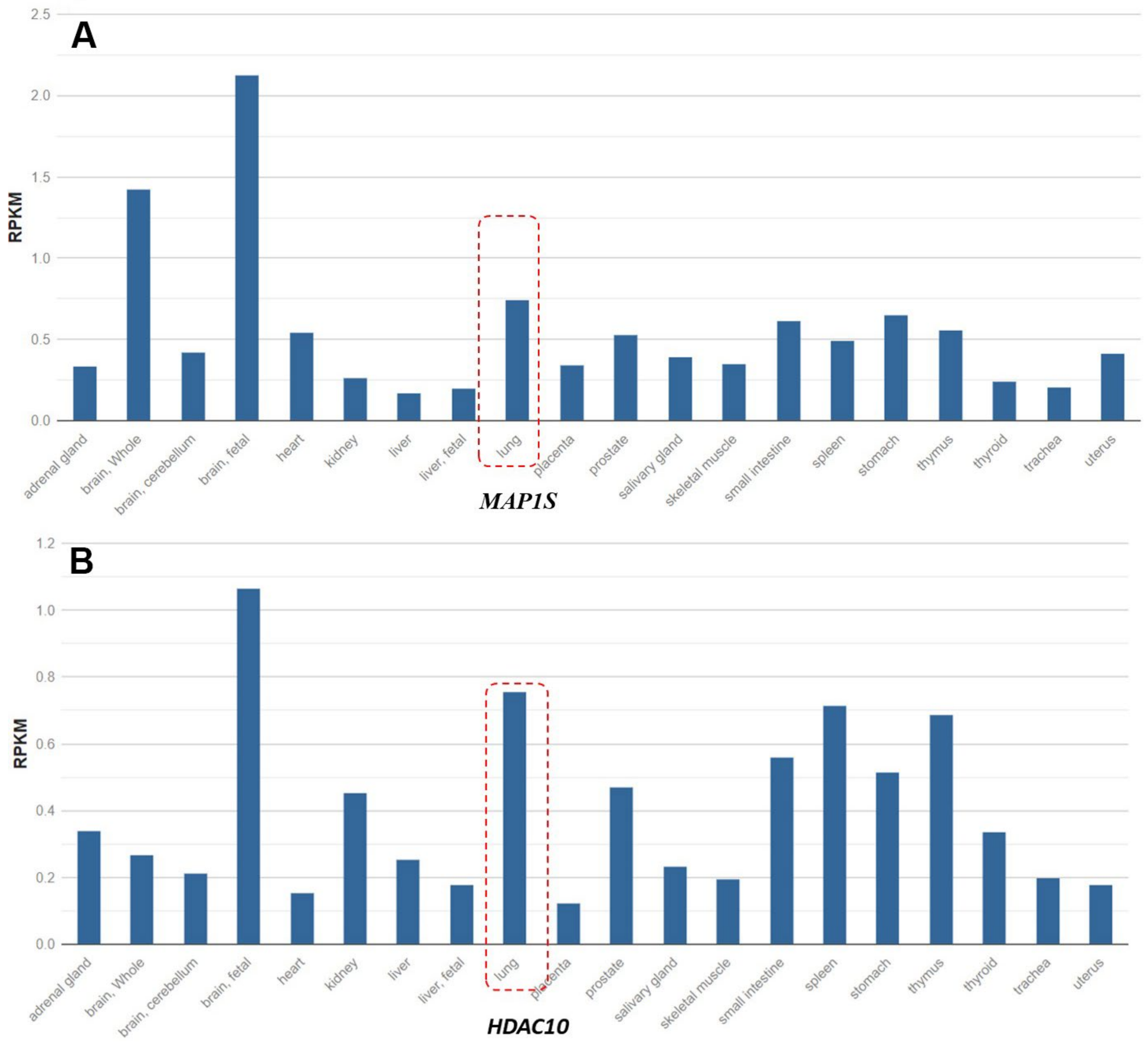

Supplementary Figure 11. Expression abundance of *MAP1S* and *HDAC10* based on RNA sequencing from 20 human tissues. The expression data were obtained from BioProject (Accession No. PRJNA280600). Expression values are shown in Reads Per Kilobase per Million mapped reads (RPKM).

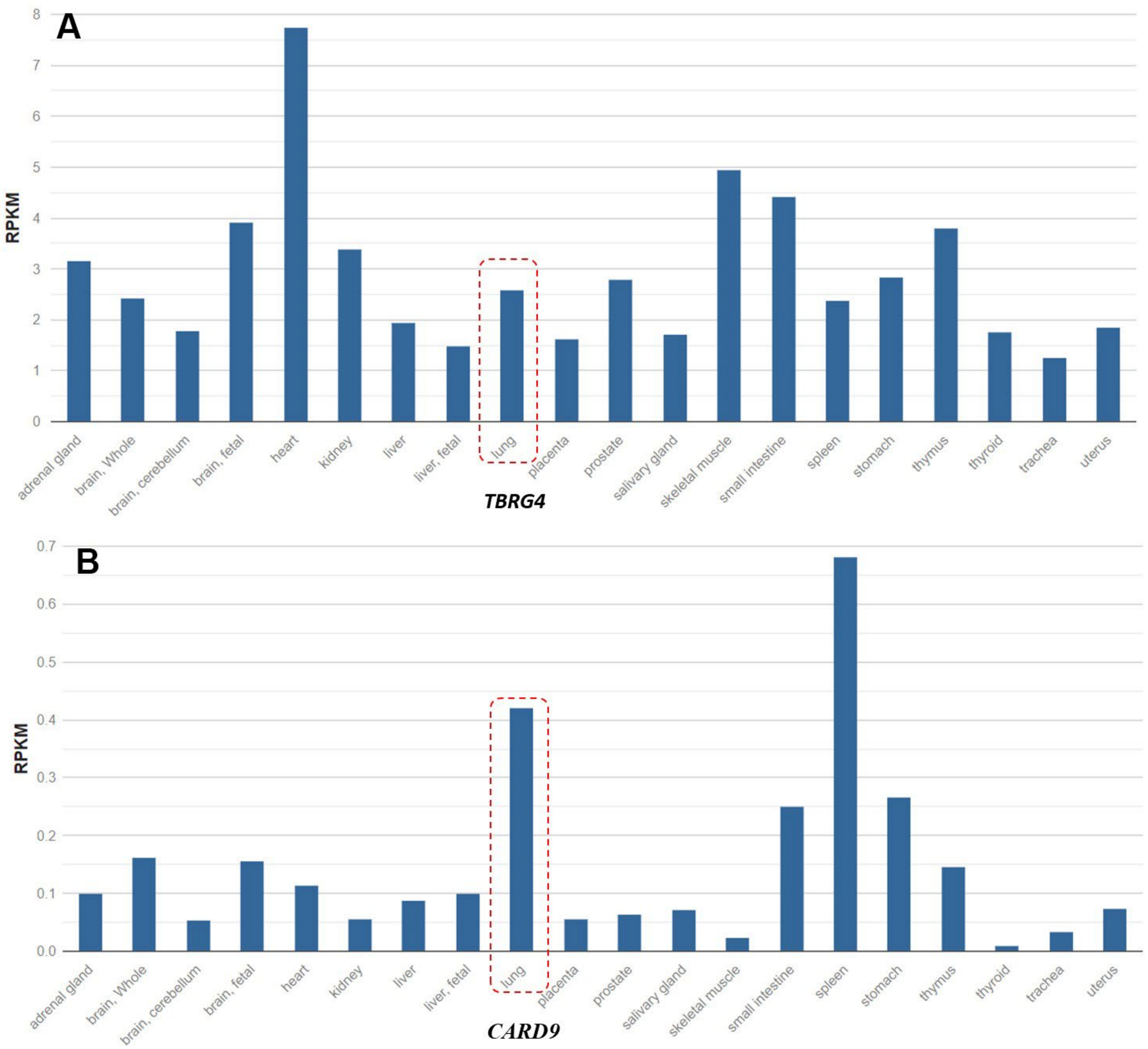

Supplementary Figure 12. Expression abundance of *TBRG4* and *CARD9* based on RNA sequencing from 20 human tissues. The expression data were obtained from BioProject (Accession No. PRJNA280600). Expression values are shown in Reads Per Kilobase per Million mapped reads (RPKM).

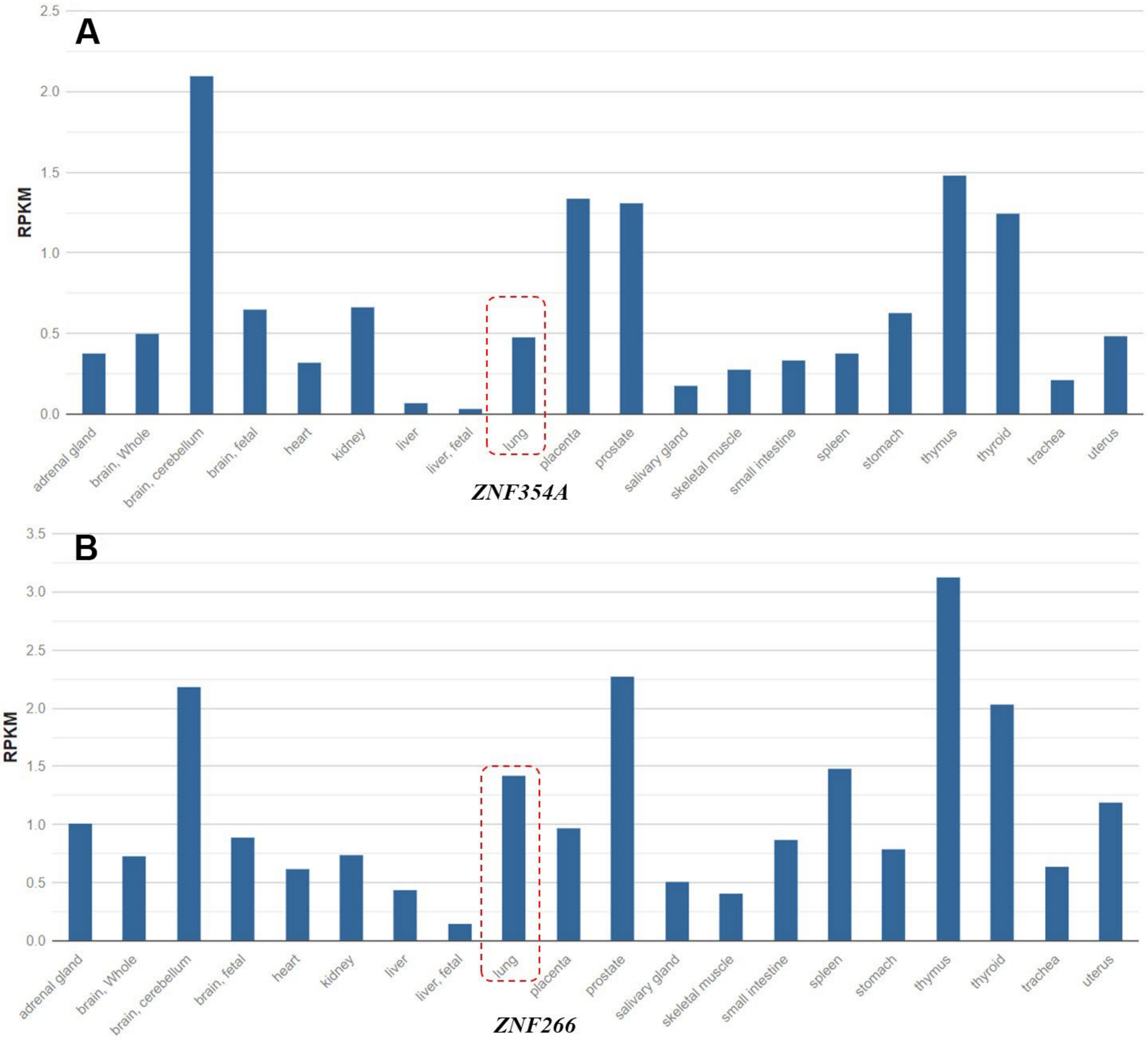

Supplementary Figure 13. Expression abundance of *ZNF354A* and *ZNF266* based on RNA sequencing from 20 human tissues. The expression data were obtained from BioProject (Accession No. PRJNA280600). Expression values are shown in Reads Per Kilobase per Million mapped reads (RPKM).

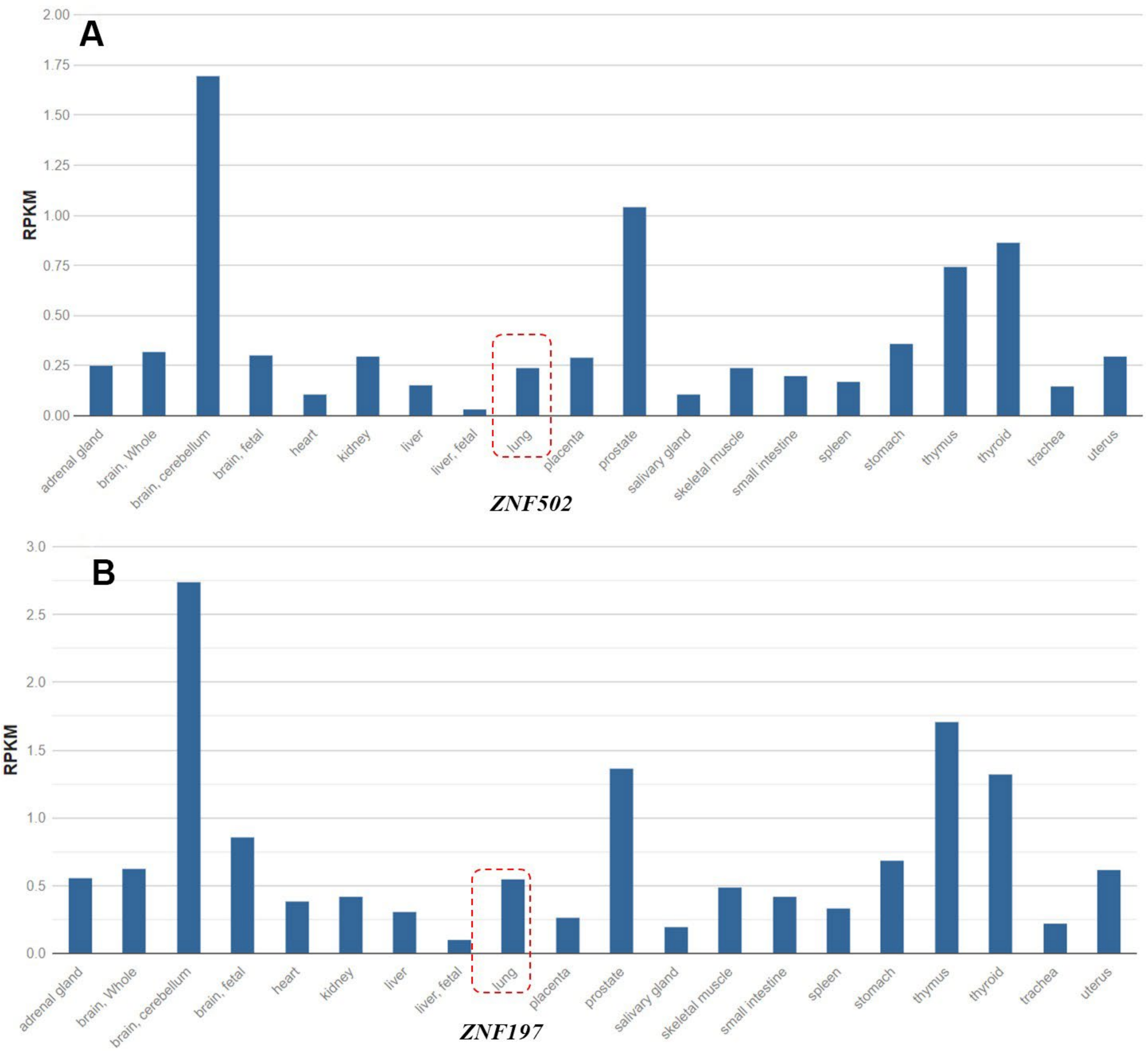

Supplementary Figure 14. Expression abundance of *ZNF502* and *ZNF197* based on RNA sequencing from 20 human tissues. The expression data were obtained from BioProject (Accession No. PRJNA280600). Expression values are shown in Reads Per Kilobase per Million mapped reads (RPKM).

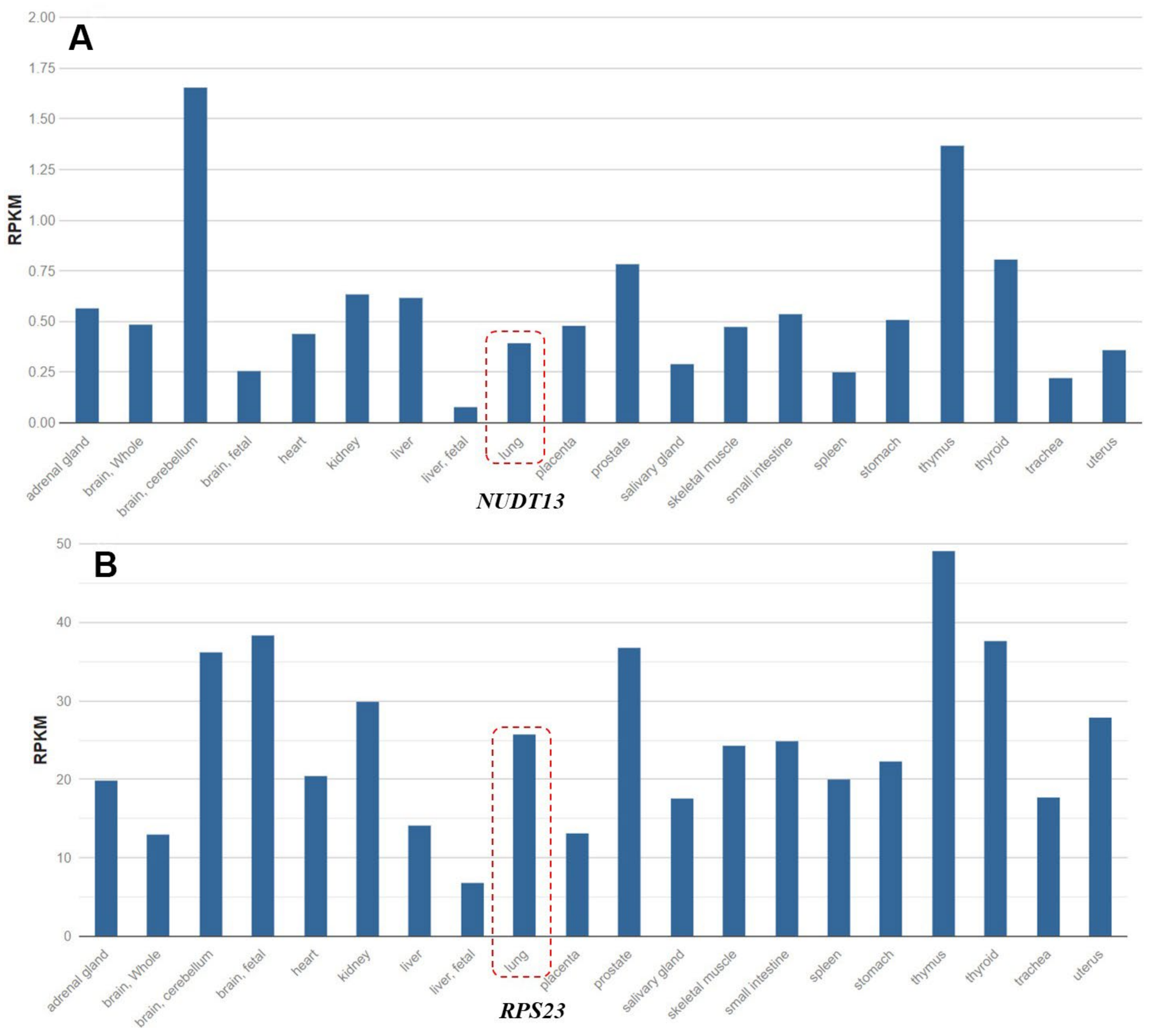

Supplementary Figure 15. Expression abundance of *NUDT13* and *RPS23* based on RNA sequencing from 20 human tissues. The expression data were obtained from BioProject (Accession No. PRJNA280600). Expression values are shown in Reads Per Kilobase per Million mapped reads (RPKM).

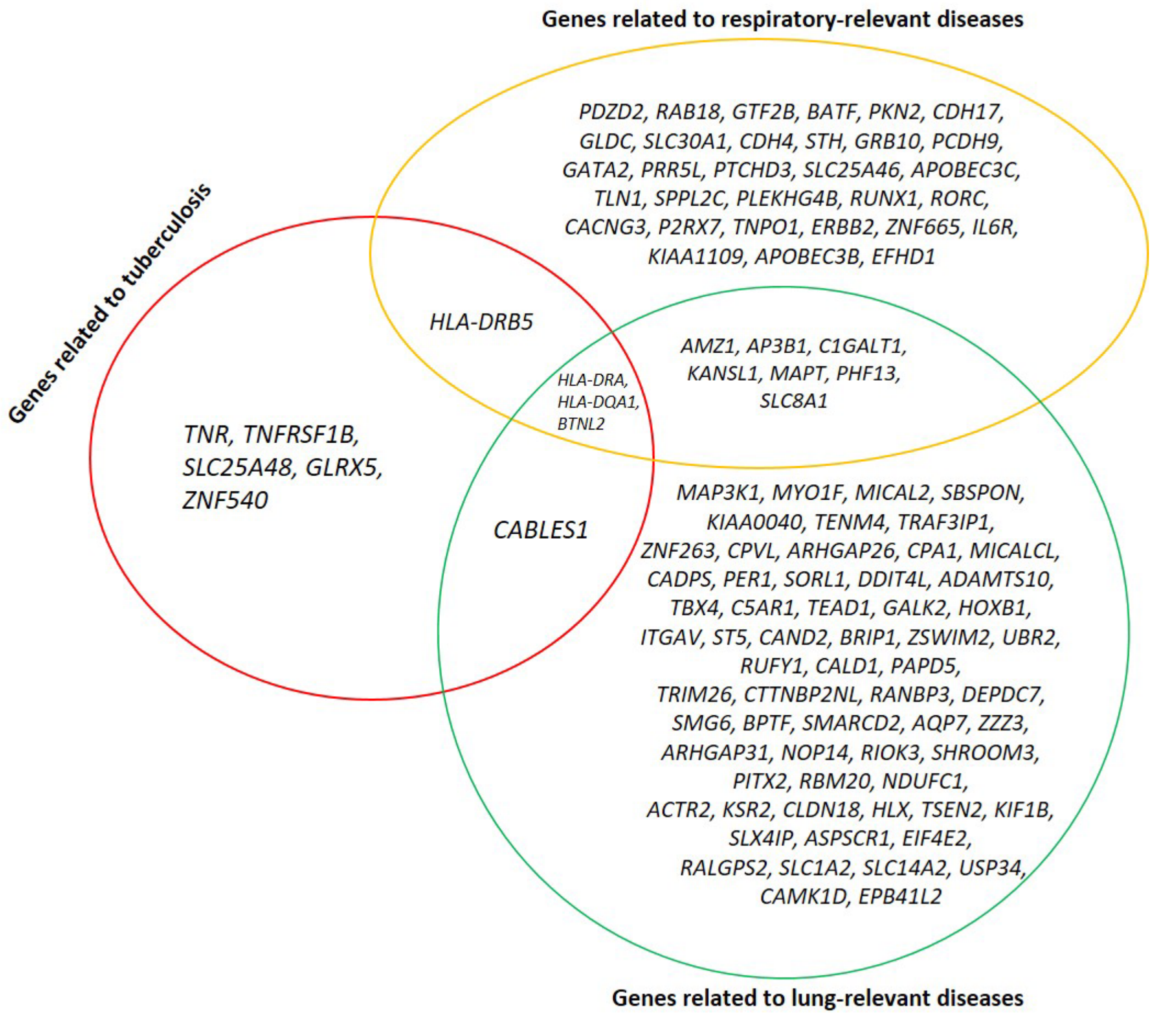

Supplementary Figure 16. Previous studies provides supportive evidence of these MAGMA-identified genes in the replication stage (based on Dataset #1).

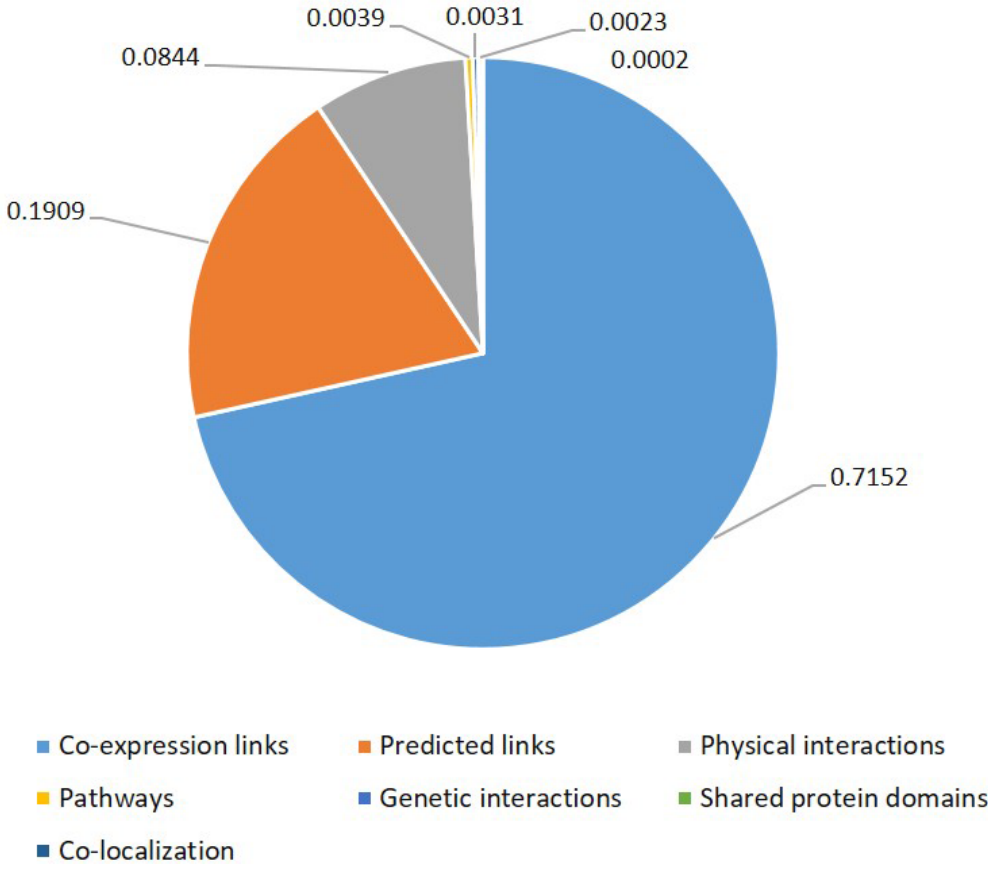

Supplementary Figure 17. The proportion of multiple layers of evidence in constructed GGI network using the GeneMANIA tool.

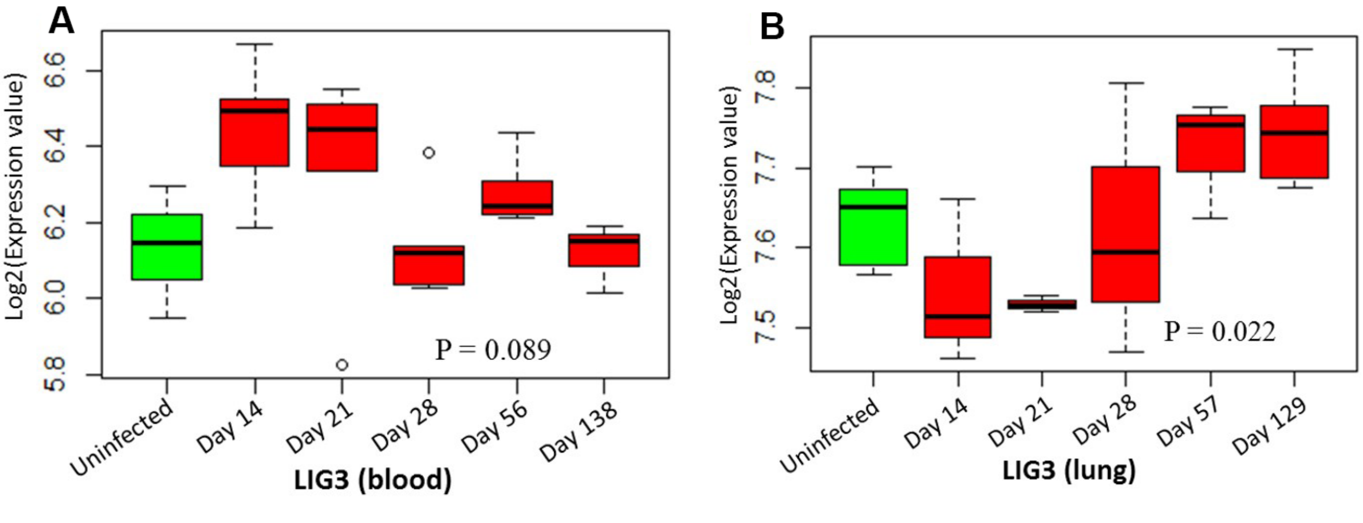

Supplementary Figure 18. Boxplots show the differential expression levels of tuberculosis-genes between uninfected mice and infected mice with 5 distinct time points based on two GSE1440943 (blood) and GSE1440944 (lung) datasets. (**A**) *LIG3* for blood; (**B**) *LIG3* for lung. P values were generated by Anova test.

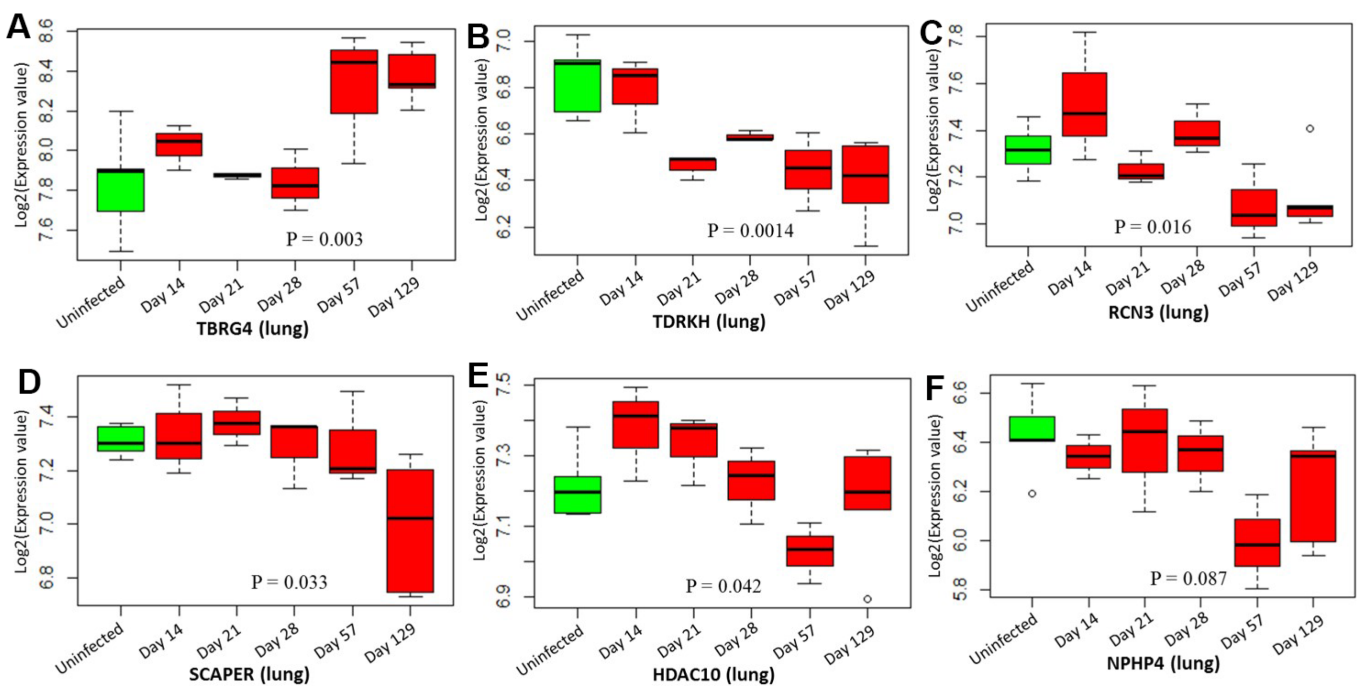

Supplementary Figure 19. Boxplots show the differential expression levels of tuberculosis-genes between uninfected mice and infected mice with 5 distinct time points based on the GSE1440944 (lung) dataset. (**A**) *TBRG4* for lung; (**B**) *TDRKH* for lung; (**C**) *RCN3* for lung; (**D**) *SCAPER* for lung; (**E**) *HDAC10* for lung; (**F**) *NPHP4* for lung. P values were generated by Anova test.

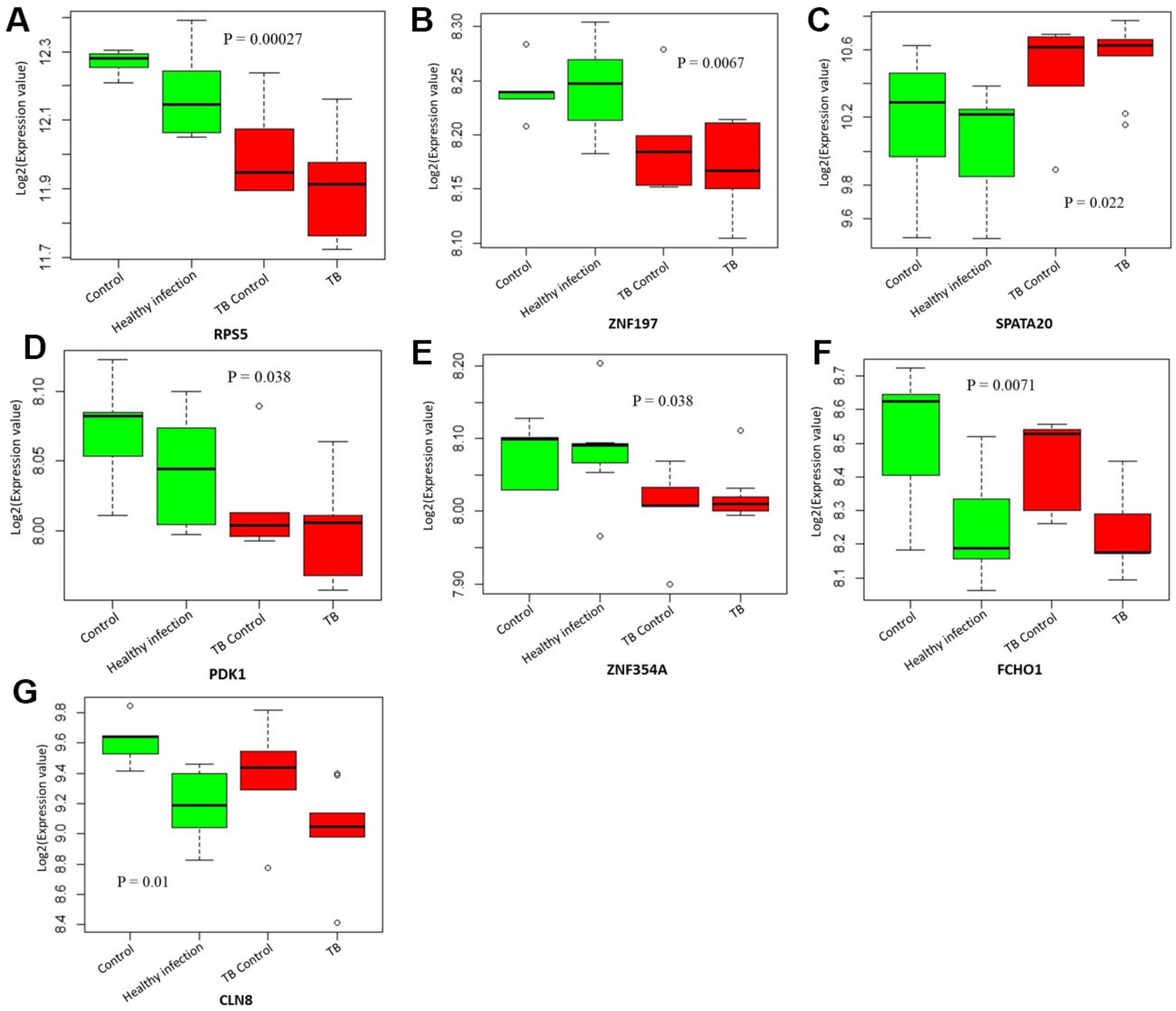

Supplementary Figure 20. Boxplots show the significantly differential expression levels of tuberculosis-genes in alveolar macrophages with four groups of TB infection, TB control, healthy infection, and healthy control based on the GSE139825 dataset. (**A**) *RPS5*; (**B**) *ZNF197*; (**C**) *SPATA20*; (**D**) *PDK1*; (**E**) *ZNF354A*; (**F**) *FCHO1*; (**G**) *CLN8*. P values were generated by Anova test.

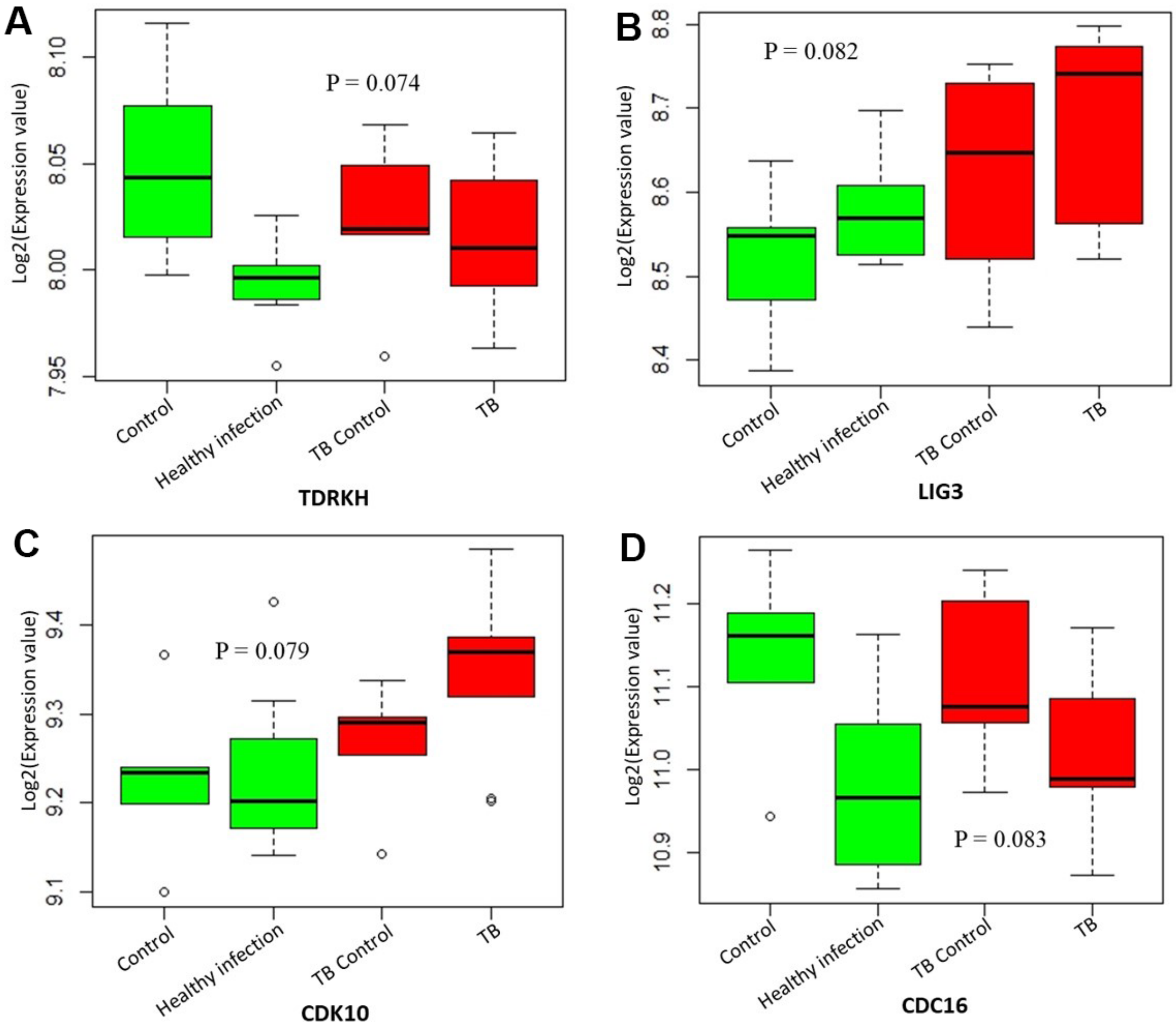

Supplementary Figure 21. Boxplots show the suggestively differential expression levels of tuberculosis-genes in alveolar macrophages with four groups of TB infection, TB control, healthy infection, and healthy control based on the GSE139825 dataset. (**A**) *RPS5*; (**B**) *ZNF197*; (**C**) *SPATA20*; (**D**) *PDK1*; (**E**) *ZNF354A*; (**F**) *FCHO1*; (**G**) *CLN8*. P values were generated by Anova test.
