# Supplementary material for: Integrative genomics analysis identifies promising SNPs and genes implicated in tuberculosis risk based on multiple omics datasets
Source: Aging (Albany NY). 2020 Oct 13;12(19):19173–220. doi: 10.18632/aging.103744 (PMC7732298; doi:10.18632/aging.103744)
Supplement: Supplementary Table 1 [file aging-12-103744-s002..docx]

**Supplementary Table 1**. **Sherlock Bayesian analysis identifies 694 genes as tuberculosis-associated risk genes (Gene set #1) from Dataset #3 in the discovery stage**

| **Gene name** | **LBF** | **Simulated P value** | **GWAS Catalog documented genes** |
| --- | --- | --- | --- |
| *SIPA1L1* | 6.95 | 1.26E-05 | Novel gene |
| *GSTA2* | 5.33 | 1.61E-04 | Novel gene |
| *TIGD6* | 4.81 | 3.02E-04 | Novel gene |
| *TSPYL4* | 4.62 | 4.22E-04 | Novel gene |
| *POLG2* | 4.53 | 4.68E-04 | Novel gene |
| *LIPF* | 4.50 | 4.81E-04 | Novel gene |
| *SS18* | 4.41 | 5.50E-04 | Novel gene |
| *GSTA1* | 4.34 | 6.08E-04 | Novel gene |
| *MAGOHB* | 4.28 | 6.51E-04 | Novel gene |
| *CD59* | 4.27 | 6.62E-04 | Novel gene |
| *ANXA1* | 4.20 | 7.33E-04 | Novel gene |
| *ITPKC* | 4.15 | 7.83E-04 | Novel gene |
| *ADRA2A* | 3.94 | 1.03E-03 | Novel gene |
| *PNRC1* | 3.84 | 1.18E-03 | Novel gene |
| *CCDC121* | 3.84 | 1.20E-03 | Novel gene |
| *CRELD1* | 3.82 | 1.22E-03 | Reported gene on lung-related diseases |
| *GZMB* | 3.82 | 1.22E-03 | Novel gene |
| *ALDH1A1* | 3.80 | 1.26E-03 | Novel gene |
| *MCM2* | 3.78 | 1.30E-03 | Novel gene |
| *CSF3R* | 3.75 | 1.34E-03 | Novel gene |
| *TMEM101* | 3.74 | 1.35E-03 | Novel gene |
| *PDCD2L* | 3.68 | 1.47E-03 | Novel gene |
| *PLD4* | 3.68 | 1.47E-03 | Novel gene |
| *DUX3* | 3.64 | 1.53E-03 | Novel gene |
| *TRAF3* | 3.59 | 1.65E-03 | Novel gene |
| *RUNX3* | 3.56 | 1.72E-03 | Reported gene on lung-related and respiratory-related diseases |
| *ZNF175* | 3.56 | 1.73E-03 | Novel gene |
| *ZNF358* | 3.53 | 1.77E-03 | Novel gene |
| *SLC35B4* | 3.53 | 1.77E-03 | Novel gene |
| *VPS29* | 3.50 | 1.84E-03 | Novel gene |
| *PLEKHA5* | 3.49 | 1.86E-03 | Novel gene |
| *TNNI3* | 3.43 | 2.01E-03 | Novel gene |
| *CHST2* | 3.39 | 2.10E-03 | Novel gene |
| *ANKRD20A3* | 3.37 | 2.17E-03 | Novel gene |
| *ANK1* | 3.34 | 2.24E-03 | Reported on respiratory-related diseases |
| *HDAC10* | 3.30 | 2.34E-03 | Novel gene |
| *HMBOX1* | 3.29 | 2.37E-03 | Novel gene |
| *THAP11* | 3.28 | 2.39E-03 | Novel gene |
| *ZC3H6* | 3.23 | 2.56E-03 | Novel gene |
| *TUBE1* | 3.23 | 2.56E-03 | Novel gene |
| *SEC62* | 3.18 | 2.71E-03 | Novel gene |
| *PARN* | 3.16 | 2.78E-03 | Novel gene |
| *SOX30* | 3.10 | 2.99E-03 | Novel gene |
| *NARFL* | 3.04 | 3.23E-03 | Novel gene |
| *KRT10* | 3.03 | 3.27E-03 | Novel gene |
| *GPSN2* | 3.02 | 3.34E-03 | Novel gene |
| *HLCS* | 2.94 | 3.71E-03 | Novel gene |
| *FAM20C* | 2.92 | 3.78E-03 | Novel gene |
| *ANAPC7* | 2.91 | 3.79E-03 | Novel gene |
| *PDK1* | 2.91 | 3.79E-03 | Novel gene |
| *IRF4* | 2.87 | 4.03E-03 | Reported gene on lung-related diseases |
| *HSPA4* | 2.85 | 4.13E-03 | Reported gene on lung-related and respiratory-related diseases |
| *SACM1L* | 2.83 | 4.28E-03 | Novel gene |
| *TFAP2E* | 2.82 | 4.33E-03 | Novel gene |
| *CCT6B* | 2.81 | 4.37E-03 | Novel gene |
| *IRF2BP1* | 2.79 | 4.46E-03 | Novel gene |
| *FAM91A2* | 2.79 | 4.50E-03 | Novel gene |
| *RHPN2* | 2.76 | 4.69E-03 | Novel gene |
| *TATDN3* | 2.75 | 4.73E-03 | Novel gene |
| *PARD3* | 2.73 | 4.81E-03 | Reported gene on lung-related diseases |
| *PGD* | 2.72 | 4.84E-03 | Novel gene |
| *ZNF74* | 2.71 | 4.94E-03 | Novel gene |
| *SCN3A* | 2.70 | 4.99E-03 | Novel gene |
| *OKL38* | 2.70 | 5.01E-03 | Novel gene |
| *CHMP1B* | 2.70 | 5.02E-03 | Novel gene |
| *LTB4R* | 2.69 | 5.09E-03 | Novel gene |
| *TMEM99* | 2.67 | 5.18E-03 | Novel gene |
| *FAM110B* | 2.63 | 5.49E-03 | Novel gene |
| *HSPA1L* | 2.61 | 5.63E-03 | Novel gene |
| *NKX6-3* | 2.60 | 5.67E-03 | Novel gene |
| *PDE4B* | 2.60 | 5.69E-03 | Novel gene |
| *FALZ* | 2.60 | 5.71E-03 | Novel gene |
| *SLC25A44* | 2.59 | 5.76E-03 | Novel gene |
| *COL18A1* | 2.58 | 5.78E-03 | Novel gene |
| *YY2* | 2.58 | 5.79E-03 | Novel gene |
| *KIR3DP1* | 2.56 | 5.89E-03 | Novel gene |
| *AKR1C4* | 2.54 | 6.04E-03 | Novel gene |
| *RWDD1* | 2.53 | 6.10E-03 | Novel gene |
| *WDR25* | 2.52 | 6.21E-03 | Novel gene |
| *CDC16* | 2.52 | 6.21E-03 | Novel gene |
| *SOX8* | 2.52 | 6.22E-03 | Novel gene |
| *GARNL4* | 2.51 | 6.28E-03 | Novel gene |
| *STX18* | 2.51 | 6.29E-03 | Novel gene |
| *KLF10* | 2.50 | 6.32E-03 | Novel gene |
| *PSMA5* | 2.50 | 6.38E-03 | Novel gene |
| *SPSB4* | 2.49 | 6.42E-03 | Novel gene |
| *OLR1* | 2.48 | 6.54E-03 | Novel gene |
| *SELE* | 2.46 | 6.72E-03 | Novel gene |
| *ABCC2* | 2.45 | 6.77E-03 | Novel gene |
| *RFP* | 2.45 | 6.80E-03 | Novel gene |
| *CCDC112* | 2.44 | 6.82E-03 | Novel gene |
| *BATF3* | 2.44 | 6.82E-03 | Novel gene |
| *KIF25* | 2.42 | 6.96E-03 | Novel gene |
| *MAMDC4* | 2.41 | 7.13E-03 | Novel gene |
| *ZNF474* | 2.40 | 7.22E-03 | Novel gene |
| *TRMT11* | 2.39 | 7.28E-03 | Novel gene |
| *CTSG* | 2.38 | 7.34E-03 | Novel gene |
| *LMTK3* | 2.38 | 7.35E-03 | Novel gene |
| *TFB2M* | 2.37 | 7.38E-03 | Novel gene |
| *DDX28* | 2.36 | 7.48E-03 | Novel gene |
| *RAD51L3* | 2.36 | 7.50E-03 | Novel gene |
| *DCST1* | 2.36 | 7.52E-03 | Novel gene |
| *SPATA20* | 2.33 | 7.80E-03 | Novel gene |
| *CMBL* | 2.32 | 7.84E-03 | Novel gene |
| *ITIH3* | 2.31 | 8.00E-03 | Novel gene |
| *TMEM145* | 2.30 | 8.01E-03 | Novel gene |
| *CYB5B* | 2.30 | 8.10E-03 | Novel gene |
| *RAB11B* | 2.29 | 8.17E-03 | Novel gene |
| *TDRKH* | 2.29 | 8.18E-03 | Novel gene |
| *DEFA3* | 2.29 | 8.20E-03 | Novel gene |
| *DHRS1* | 2.27 | 8.35E-03 | Novel gene |
| *PURA* | 2.27 | 8.37E-03 | Novel gene |
| *DCUN1D1* | 2.26 | 8.53E-03 | Novel gene |
| *OR52B6* | 2.25 | 8.61E-03 | Novel gene |
| *TBX18* | 2.25 | 8.64E-03 | Novel gene |
| *DHRS4L2* | 2.25 | 8.64E-03 | Novel gene |
| *ALG2* | 2.23 | 8.78E-03 | Novel gene |
| *FAM19A4* | 2.23 | 8.79E-03 | Novel gene |
| *HNRNPA0* | 2.23 | 8.80E-03 | Novel gene |
| *PCYT2* | 2.23 | 8.83E-03 | Novel gene |
| *TMEM205* | 2.22 | 8.89E-03 | Novel gene |
| *CMTM2* | 2.22 | 8.93E-03 | Novel gene |
| *IL17D* | 2.22 | 8.99E-03 | Novel gene |
| *SH2D1B* | 2.20 | 9.14E-03 | Novel gene |
| *S100A11* | 2.19 | 9.20E-03 | Reported on respiratory-related diseases |
| *ZFP37* | 2.18 | 9.40E-03 | Novel gene |
| *TMEM41A* | 2.16 | 9.65E-03 | Novel gene |
| *KREMEN1* | 2.15 | 9.67E-03 | Reported gene on lung-related diseases |
| *GLI1* | 2.15 | 9.68E-03 | Novel gene |
| *TEDDM1* | 2.15 | 9.76E-03 | Novel gene |
| *SIGLECP16* | 2.14 | 9.79E-03 | Novel gene |
| *PTGES2* | 2.13 | 9.94E-03 | Novel gene |
| *RASSF4* | 2.13 | 9.94E-03 | Novel gene |
| *LSP1* | 2.13 | 9.95E-03 | Novel gene |
| *ZBED5* | 2.13 | 9.96E-03 | Novel gene |
| *NAGLU* | 2.12 | 1.00E-02 | Novel gene |
| *TMEM202* | 2.12 | 1.01E-02 | Novel gene |
| *PAQR8* | 2.12 | 1.01E-02 | Novel gene |
| *TMEM191B* | 2.11 | 1.02E-02 | Novel gene |
| *TMEM59* | 2.11 | 1.03E-02 | Novel gene |
| *SEC31B* | 2.10 | 1.03E-02 | Novel gene |
| *IKZF3* | 2.10 | 1.03E-02 | Reported on respiratory-related diseases |
| *PVRIG* | 2.10 | 1.03E-02 | Novel gene |
| *TMEM47* | 2.10 | 1.04E-02 | Novel gene |
| *TFCP2* | 2.09 | 1.05E-02 | Novel gene |
| *RPL35* | 2.09 | 1.05E-02 | Novel gene |
| *NASP* | 2.09 | 1.05E-02 | Novel gene |
| *ZNF764* | 2.06 | 1.09E-02 | Novel gene |
| *SLC9A6* | 2.05 | 1.10E-02 | Novel gene |
| *RRN3* | 2.05 | 1.10E-02 | Novel gene |
| *SDC1* | 2.03 | 1.12E-02 | Novel gene |
| *NUDT11* | 2.03 | 1.12E-02 | Novel gene |
| *FZD10* | 2.03 | 1.13E-02 | Novel gene |
| *RAPGEF4* | 2.03 | 1.13E-02 | Novel gene |
| *S100A2* | 2.02 | 1.13E-02 | Novel gene |
| *ETFA* | 2.02 | 1.13E-02 | Novel gene |
| *TNFRSF12A* | 2.02 | 1.14E-02 | Novel gene |
| *NPHP4* | 2.01 | 1.15E-02 | Novel gene |
| *TRDMT1* | 2.01 | 1.15E-02 | Novel gene |
| *BRUNOL6* | 2.01 | 1.15E-02 | Novel gene |
| *ZIK1* | 2.01 | 1.16E-02 | Novel gene |
| *FAM22F* | 2.00 | 1.17E-02 | Novel gene |
| *IFNGR2* | 2.00 | 1.18E-02 | Novel gene |
| *ILDR1* | 1.99 | 1.18E-02 | Novel gene |
| *ACTL6B* | 1.99 | 1.19E-02 | Novel gene |
| *UBE2V1* | 1.98 | 1.19E-02 | Novel gene |
| *TH1L* | 1.96 | 1.23E-02 | Novel gene |
| *TGM7* | 1.95 | 1.24E-02 | Novel gene |
| *HIST1H4E* | 1.94 | 1.25E-02 | Novel gene |
| *HNRPH1* | 1.94 | 1.25E-02 | Novel gene |
| *IL10* | 1.94 | 1.25E-02 | Novel gene |
| *PEBP1* | 1.93 | 1.27E-02 | Novel gene |
| *TNNI2* | 1.92 | 1.27E-02 | Novel gene |
| *MALT1* | 1.92 | 1.28E-02 | Novel gene |
| *HDAC7A* | 1.91 | 1.29E-02 | Novel gene |
| *HPR* | 1.91 | 1.29E-02 | Novel gene |
| *PLGLA* | 1.90 | 1.32E-02 | Novel gene |
| *ZNF25* | 1.88 | 1.34E-02 | Novel gene |
| *LMAN1L* | 1.88 | 1.34E-02 | Novel gene |
| *RORA* | 1.88 | 1.35E-02 | Reported on respiratory-related diseases |
| *TWIST2* | 1.87 | 1.37E-02 | Reported on respiratory-related diseases |
| *CCRK* | 1.86 | 1.38E-02 | Novel gene |
| *UNC119* | 1.86 | 1.39E-02 | Novel gene |
| *SETD4* | 1.85 | 1.39E-02 | Novel gene |
| *S100PBP* | 1.84 | 1.41E-02 | Novel gene |
| *CNOT6* | 1.84 | 1.41E-02 | Novel gene |
| *NAV1* | 1.82 | 1.45E-02 | Novel gene |
| *SDCCAG3* | 1.82 | 1.45E-02 | Novel gene |
| *MRPS30* | 1.81 | 1.46E-02 | Novel gene |
| *RPAP2* | 1.81 | 1.46E-02 | Novel gene |
| *EDEM2* | 1.81 | 1.47E-02 | Novel gene |
| *CEP290* | 1.80 | 1.48E-02 | Novel gene |
| *P2RX4* | 1.80 | 1.49E-02 | Novel gene |
| *HIATL1* | 1.79 | 1.51E-02 | Novel gene |
| *DPF2* | 1.78 | 1.53E-02 | Novel gene |
| *CRLF1* | 1.77 | 1.55E-02 | Novel gene |
| *TMSB4X* | 1.77 | 1.55E-02 | Novel gene |
| *AP1M2* | 1.77 | 1.55E-02 | Novel gene |
| *ZRANB3* | 1.77 | 1.55E-02 | Novel gene |
| *SURF1* | 1.76 | 1.56E-02 | Novel gene |
| *PID1* | 1.76 | 1.56E-02 | Reported gene on lung-related diseases |
| *STBD1* | 1.76 | 1.57E-02 | Novel gene |
| *ADH6* | 1.75 | 1.57E-02 | Novel gene |
| *TECTA* | 1.75 | 1.58E-02 | Novel gene |
| *SETD3* | 1.75 | 1.58E-02 | Novel gene |
| *SEMA5A* | 1.74 | 1.60E-02 | Novel gene |
| *KCNK10* | 1.74 | 1.60E-02 | Novel gene |
| *DUSP18* | 1.73 | 1.62E-02 | Novel gene |
| *B3GALTL* | 1.72 | 1.63E-02 | Novel gene |
| *SNX30* | 1.72 | 1.64E-02 | Novel gene |
| *FAM53A* | 1.71 | 1.65E-02 | Novel gene |
| *FOXD2* | 1.71 | 1.66E-02 | Novel gene |
| *YEATS4* | 1.70 | 1.66E-02 | Novel gene |
| *TBRG4* | 1.70 | 1.67E-02 | Novel gene |
| *PLDN* | 1.70 | 1.68E-02 | Novel gene |
| *C1QB* | 1.70 | 1.68E-02 | Novel gene |
| *CAMK1D* | 1.70 | 1.68E-02 | Reported gene on lung-related diseases |
| *IFIT2* | 1.69 | 1.68E-02 | Novel gene |
| *ZNF641* | 1.69 | 1.69E-02 | Novel gene |
| *HIST2H4A* | 1.69 | 1.69E-02 | Novel gene |
| *CAMTA1* | 1.69 | 1.69E-02 | Novel gene |
| *DDX51* | 1.69 | 1.70E-02 | Novel gene |
| *ZNF721* | 1.69 | 1.70E-02 | Novel gene |
| *RRM2B* | 1.68 | 1.70E-02 | Reported on respiratory-related diseases |
| *SERGEF* | 1.68 | 1.70E-02 | Novel gene |
| *PRPS1L1* | 1.68 | 1.70E-02 | Novel gene |
| *FADS6* | 1.68 | 1.72E-02 | Novel gene |
| *CARD9* | 1.67 | 1.73E-02 | Reported gene on lung-related diseases |
| *RAET1E* | 1.67 | 1.73E-02 | Novel gene |
| *SLC4A8* | 1.67 | 1.73E-02 | Novel gene |
| *EPB41L2* | 1.67 | 1.73E-02 | Reported gene on lung-related diseases |
| *INPP5E* | 1.67 | 1.73E-02 | Novel gene |
| *KRTAP10-9* | 1.67 | 1.74E-02 | Novel gene |
| *TRY1* | 1.67 | 1.74E-02 | Novel gene |
| *ZNF354A* | 1.67 | 1.74E-02 | Novel gene |
| *AURKB* | 1.66 | 1.74E-02 | Novel gene |
| *SLC6A15* | 1.66 | 1.75E-02 | Novel gene |
| *BCAS4* | 1.66 | 1.75E-02 | Novel gene |
| *SLC33A1* | 1.65 | 1.77E-02 | Novel gene |
| *SPPL2A* | 1.64 | 1.79E-02 | Reported on respiratory-related diseases |
| *RPUSD4* | 1.64 | 1.79E-02 | Reported gene on lung-related diseases |
| *CCDC120* | 1.64 | 1.80E-02 | Novel gene |
| *ZBTB6* | 1.64 | 1.81E-02 | Novel gene |
| *CLRN1* | 1.64 | 1.81E-02 | Novel gene |
| *RARRES2* | 1.64 | 1.81E-02 | Novel gene |
| *TSGA10IP* | 1.63 | 1.83E-02 | Novel gene |
| *BAT2D1* | 1.63 | 1.83E-02 | Novel gene |
| *MYO7B* | 1.61 | 1.86E-02 | Novel gene |
| *DEPDC7* | 1.60 | 1.88E-02 | Reported gene on lung-related diseases |
| *NUCB1* | 1.60 | 1.88E-02 | Novel gene |
| *SCRN2* | 1.60 | 1.89E-02 | Novel gene |
| *TAF13* | 1.60 | 1.89E-02 | Novel gene |
| *IL18* | 1.59 | 1.90E-02 | Novel gene |
| *SNF1LK* | 1.59 | 1.90E-02 | Novel gene |
| *TMEM155* | 1.59 | 1.90E-02 | Novel gene |
| *IRS1* | 1.59 | 1.90E-02 | Novel gene |
| *NDNL2* | 1.59 | 1.91E-02 | Novel gene |
| *GPRASP2* | 1.59 | 1.92E-02 | Novel gene |
| *CEP152* | 1.58 | 1.93E-02 | Novel gene |
| *ZNF714* | 1.58 | 1.93E-02 | Novel gene |
| *PHYH* | 1.58 | 1.93E-02 | Novel gene |
| *APP* | 1.57 | 1.95E-02 | Novel gene |
| *BCYRN1* | 1.57 | 1.95E-02 | Novel gene |
| *ZNF786* | 1.57 | 1.95E-02 | Novel gene |
| *ZNF37A* | 1.57 | 1.96E-02 | Novel gene |
| *CD8A* | 1.56 | 1.97E-02 | Novel gene |
| *ALOX5AP* | 1.56 | 1.97E-02 | Novel gene |
| *PKD1L3* | 1.56 | 1.98E-02 | Reported gene on lung-related diseases |
| *VAMP4* | 1.56 | 1.98E-02 | Novel gene |
| *WTAP* | 1.55 | 1.99E-02 | Novel gene |
| *APCS* | 1.55 | 1.99E-02 | Novel gene |
| *AKT1* | 1.55 | 2.00E-02 | Novel gene |
| *NLRP8* | 1.55 | 2.01E-02 | Novel gene |
| *RCN3* | 1.54 | 2.01E-02 | Novel gene |
| *GAGE7* | 1.54 | 2.01E-02 | Novel gene |
| *NR2F2* | 1.54 | 2.03E-02 | Novel gene |
| *DDX11* | 1.54 | 2.03E-02 | Novel gene |
| *TBX22* | 1.53 | 2.04E-02 | Novel gene |
| *TRIM44* | 1.53 | 2.05E-02 | Novel gene |
| *SSB* | 1.52 | 2.07E-02 | Novel gene |
| *RNMT* | 1.52 | 2.08E-02 | Novel gene |
| *TBN* | 1.52 | 2.08E-02 | Novel gene |
| *DHODH* | 1.52 | 2.09E-02 | Novel gene |
| *WNT2B* | 1.51 | 2.10E-02 | Novel gene |
| *PLVAP* | 1.51 | 2.10E-02 | Novel gene |
| *CLN8* | 1.51 | 2.10E-02 | Novel gene |
| *TNF* | 1.51 | 2.10E-02 | Novel gene |
| *LTA* | 1.51 | 2.12E-02 | Novel gene |
| *TRK1* | 1.51 | 2.12E-02 | Novel gene |
| *RSPH1* | 1.50 | 2.12E-02 | Novel gene |
| *ALPP* | 1.50 | 2.13E-02 | Novel gene |
| *NXPH1* | 1.50 | 2.13E-02 | Novel gene |
| *NUP210* | 1.50 | 2.14E-02 | Novel gene |
| *POU5F1* | 1.50 | 2.14E-02 | Novel gene |
| *IL27* | 1.49 | 2.15E-02 | Reported gene on lung-related diseases |
| *BCAS3* | 1.49 | 2.16E-02 | Reported gene on lung-related and respiratory-related diseases |
| *NPL* | 1.49 | 2.16E-02 | Novel gene |
| *CD300C* | 1.48 | 2.17E-02 | Novel gene |
| *TRIM71* | 1.48 | 2.19E-02 | Reported gene on lung-related and respiratory-related diseases |
| *EID1* | 1.48 | 2.20E-02 | Novel gene |
| *LHX5* | 1.48 | 2.20E-02 | Novel gene |
| *NUP50* | 1.47 | 2.21E-02 | Novel gene |
| *RWDD4A* | 1.47 | 2.21E-02 | Novel gene |
| *PRR7* | 1.47 | 2.22E-02 | Novel gene |
| *KRT9* | 1.47 | 2.22E-02 | Novel gene |
| *VDAC1* | 1.47 | 2.22E-02 | Novel gene |
| *RALA* | 1.47 | 2.22E-02 | Novel gene |
| *FAM53B* | 1.46 | 2.24E-02 | Novel gene |
| *SNTB2* | 1.45 | 2.25E-02 | Novel gene |
| *PPP1R16B* | 1.45 | 2.25E-02 | Novel gene |
| *ZNF202* | 1.45 | 2.26E-02 | Novel gene |
| *TRIM31* | 1.45 | 2.28E-02 | Novel gene |
| *FAM108C1* | 1.44 | 2.31E-02 | Novel gene |
| *NTN1* | 1.43 | 2.31E-02 | Novel gene |
| *NUDT18* | 1.43 | 2.31E-02 | Novel gene |
| *GRINL1A* | 1.43 | 2.32E-02 | Novel gene |
| *IMPG2* | 1.43 | 2.32E-02 | Novel gene |
| *DAK* | 1.43 | 2.32E-02 | Novel gene |
| *AHCY* | 1.42 | 2.35E-02 | Novel gene |
| *PSMC5* | 1.42 | 2.35E-02 | Novel gene |
| *HNRNPA3* | 1.42 | 2.35E-02 | Novel gene |
| *CMKLR1* | 1.42 | 2.36E-02 | Novel gene |
| *NUDCD3* | 1.41 | 2.39E-02 | Novel gene |
| *GSTO2* | 1.41 | 2.39E-02 | Novel gene |
| *CHMP1A* | 1.41 | 2.39E-02 | Novel gene |
| *POLR2F* | 1.40 | 2.40E-02 | Novel gene |
| *NETO2* | 1.40 | 2.40E-02 | Novel gene |
| *LSAMP* | 1.40 | 2.41E-02 | Novel gene |
| *SERINC4* | 1.40 | 2.41E-02 | Novel gene |
| *DKFZP434L187* | 1.40 | 2.41E-02 | Novel gene |
| *RAB11FIP2* | 1.39 | 2.43E-02 | Reported on respiratory-related diseases |
| *CRTAM* | 1.39 | 2.43E-02 | Novel gene |
| *MYO5C* | 1.39 | 2.44E-02 | Novel gene |
| *SHF* | 1.39 | 2.45E-02 | Novel gene |
| *DMC1* | 1.38 | 2.46E-02 | Novel gene |
| *EMP1* | 1.38 | 2.47E-02 | Novel gene |
| *RBM6* | 1.38 | 2.48E-02 | Novel gene |
| *PNPT1* | 1.37 | 2.48E-02 | Novel gene |
| *MMP11* | 1.37 | 2.48E-02 | Novel gene |
| *SHARPIN* | 1.37 | 2.49E-02 | Reported on respiratory-related diseases |
| *AES* | 1.37 | 2.49E-02 | Novel gene |
| *FAM39DP* | 1.37 | 2.50E-02 | Novel gene |
| *XIST* | 1.37 | 2.50E-02 | Novel gene |
| *OR2AK2* | 1.37 | 2.51E-02 | Novel gene |
| *RAB3IP* | 1.36 | 2.52E-02 | Novel gene |
| *DNAJB2* | 1.36 | 2.52E-02 | Novel gene |
| *FMO5* | 1.36 | 2.53E-02 | Novel gene |
| *AOC2* | 1.36 | 2.53E-02 | Novel gene |
| *FAM40B* | 1.36 | 2.54E-02 | Novel gene |
| *FCF1* | 1.35 | 2.55E-02 | Reported gene on lung-related diseases |
| *TLR6* | 1.35 | 2.55E-02 | Reported on respiratory-related diseases |
| *CLDN19* | 1.35 | 2.56E-02 | Novel gene |
| *CRADD* | 1.35 | 2.57E-02 | Reported gene on lung-related diseases |
| *NOD2* | 1.35 | 2.57E-02 | Novel gene |
| *TBC1D10A* | 1.34 | 2.58E-02 | Novel gene |
| *FAM131B* | 1.34 | 2.59E-02 | Novel gene |
| *H1FNT* | 1.34 | 2.60E-02 | Novel gene |
| *ERAP1* | 1.33 | 2.62E-02 | Novel gene |
| *RARS* | 1.33 | 2.62E-02 | Novel gene |
| *ERO1L* | 1.32 | 2.65E-02 | Novel gene |
| *NDUFB2* | 1.32 | 2.65E-02 | Novel gene |
| *ERCC4* | 1.32 | 2.66E-02 | Reported on respiratory-related diseases |
| *UROC1* | 1.32 | 2.66E-02 | Novel gene |
| *RASGRP1* | 1.32 | 2.66E-02 | Novel gene |
| *HOPX* | 1.31 | 2.68E-02 | Novel gene |
| *EMX2* | 1.31 | 2.69E-02 | Novel gene |
| *NEK11* | 1.31 | 2.71E-02 | Novel gene |
| *SDCCAG3L* | 1.31 | 2.71E-02 | Novel gene |
| *F2R* | 1.30 | 2.71E-02 | Novel gene |
| *COX10* | 1.30 | 2.73E-02 | Novel gene |
| *CCPG1* | 1.30 | 2.73E-02 | Novel gene |
| *SMTNL1* | 1.30 | 2.74E-02 | Novel gene |
| *ZAN* | 1.29 | 2.75E-02 | Novel gene |
| *LRTM1* | 1.29 | 2.75E-02 | Novel gene |
| *DHX40* | 1.29 | 2.76E-02 | Novel gene |
| *ANXA2P3* | 1.29 | 2.76E-02 | Novel gene |
| *TFAM* | 1.29 | 2.76E-02 | Novel gene |
| *IFI44L* | 1.29 | 2.76E-02 | Novel gene |
| *USP36* | 1.29 | 2.77E-02 | Novel gene |
| *MAD2L2* | 1.28 | 2.79E-02 | Novel gene |
| *CCDC136* | 1.28 | 2.79E-02 | Novel gene |
| *PRKCZ* | 1.28 | 2.80E-02 | Novel gene |
| *BATF* | 1.28 | 2.81E-02 | Reported on respiratory-related diseases |
| *OAT* | 1.28 | 2.81E-02 | Novel gene |
| *SURF6* | 1.28 | 2.82E-02 | Novel gene |
| *DENND1A* | 1.27 | 2.82E-02 | Novel gene |
| *GCUD2* | 1.27 | 2.83E-02 | Novel gene |
| *NDST1* | 1.27 | 2.83E-02 | Reported gene on lung-related diseases |
| *TNFAIP8L3* | 1.27 | 2.84E-02 | Novel gene |
| *ST6GALNAC3* | 1.27 | 2.85E-02 | Novel gene |
| *FAM104B* | 1.25 | 2.90E-02 | Novel gene |
| *RNF183* | 1.25 | 2.90E-02 | Novel gene |
| *ADCYAP1* | 1.25 | 2.90E-02 | Novel gene |
| *WDR43* | 1.25 | 2.91E-02 | Novel gene |
| *DNAJB12* | 1.25 | 2.91E-02 | Novel gene |
| *MAP2K6* | 1.25 | 2.93E-02 | Novel gene |
| *FCHO1* | 1.25 | 2.93E-02 | Novel gene |
| *TMEM123* | 1.24 | 2.93E-02 | Novel gene |
| *MYST4* | 1.24 | 2.94E-02 | Novel gene |
| *HMBS* | 1.24 | 2.94E-02 | Novel gene |
| *ZNF683* | 1.24 | 2.95E-02 | Novel gene |
| *GPS2* | 1.23 | 2.97E-02 | Novel gene |
| *ALDH2* | 1.23 | 2.97E-02 | Novel gene |
| *SLC39A7* | 1.23 | 2.98E-02 | Novel gene |
| *TRAPPC1* | 1.23 | 2.99E-02 | Novel gene |
| *FNBP1L* | 1.23 | 2.99E-02 | Novel gene |
| *NPHP3* | 1.22 | 3.01E-02 | Novel gene |
| *DNAJC4* | 1.22 | 3.01E-02 | Novel gene |
| *NCF1B* | 1.22 | 3.02E-02 | Novel gene |
| *SF3A1* | 1.21 | 3.04E-02 | Novel gene |
| *CCM2* | 1.21 | 3.04E-02 | Novel gene |
| *DHX57* | 1.21 | 3.05E-02 | Novel gene |
| *CXXC4* | 1.21 | 3.05E-02 | Novel gene |
| *THRAP5* | 1.21 | 3.07E-02 | Novel gene |
| *CDK10* | 1.20 | 3.08E-02 | Novel gene |
| *ZNF75A* | 1.19 | 3.12E-02 | Novel gene |
| *NPEPPS* | 1.19 | 3.13E-02 | Novel gene |
| *MX1* | 1.19 | 3.13E-02 | Novel gene |
| *BBS9* | 1.19 | 3.16E-02 | Reported on respiratory-related diseases |
| *CETN2* | 1.18 | 3.17E-02 | Novel gene |
| *PIK3C3* | 1.18 | 3.17E-02 | Novel gene |
| *ENTPD6* | 1.18 | 3.19E-02 | Reported gene on lung-related diseases |
| *CANT1* | 1.18 | 3.20E-02 | Novel gene |
| *SLC28A3* | 1.17 | 3.20E-02 | Novel gene |
| *CD96* | 1.17 | 3.20E-02 | Reported on respiratory-related diseases |
| *GUCA1C* | 1.17 | 3.21E-02 | Novel gene |
| *BRAF* | 1.17 | 3.22E-02 | Reported gene on lung-related diseases |
| *TWISTNB* | 1.17 | 3.24E-02 | Novel gene |
| *NUDT13* | 1.16 | 3.27E-02 | Novel gene |
| *MOBKL2B* | 1.16 | 3.28E-02 | Novel gene |
| *NSF* | 1.15 | 3.30E-02 | Novel gene |
| *MCAT* | 1.15 | 3.32E-02 | Novel gene |
| *PLEKHF1* | 1.15 | 3.33E-02 | Novel gene |
| *DISC1* | 1.15 | 3.34E-02 | Novel gene |
| *NECAB3* | 1.15 | 3.34E-02 | Novel gene |
| *TAZ* | 1.14 | 3.34E-02 | Novel gene |
| *SERPINA1* | 1.14 | 3.35E-02 | Novel gene |
| *CA4* | 1.14 | 3.36E-02 | Novel gene |
| *GTF3C5* | 1.13 | 3.38E-02 | Novel gene |
| *OPTN* | 1.13 | 3.39E-02 | Novel gene |
| *RAB2A* | 1.13 | 3.41E-02 | Novel gene |
| *RFC3* | 1.12 | 3.43E-02 | Novel gene |
| *BUD13* | 1.12 | 3.44E-02 | Reported gene on lung-related diseases |
| *CHERP* | 1.12 | 3.44E-02 | Novel gene |
| *CCDC96* | 1.12 | 3.45E-02 | Novel gene |
| *DHRS13* | 1.12 | 3.45E-02 | Novel gene |
| *UPF1* | 1.11 | 3.47E-02 | Novel gene |
| *CPNE5* | 1.11 | 3.48E-02 | Novel gene |
| *FTH1* | 1.11 | 3.48E-02 | Novel gene |
| *DR1* | 1.11 | 3.50E-02 | Novel gene |
| *TNFRSF21* | 1.10 | 3.52E-02 | Novel gene |
| *TBC1D2B* | 1.10 | 3.52E-02 | Novel gene |
| *ADRA1B* | 1.10 | 3.52E-02 | Novel gene |
| *HDAC8* | 1.10 | 3.53E-02 | Novel gene |
| *QDPR* | 1.10 | 3.53E-02 | Novel gene |
| *FCRL1* | 1.10 | 3.54E-02 | Novel gene |
| *MMP23B* | 1.10 | 3.55E-02 | Novel gene |
| *SCARA5* | 1.10 | 3.55E-02 | Novel gene |
| *TRMT1* | 1.10 | 3.56E-02 | Novel gene |
| *HPS6* | 1.10 | 3.56E-02 | Novel gene |
| *ZNF345* | 1.09 | 3.57E-02 | Novel gene |
| *UQCRC2* | 1.09 | 3.57E-02 | Novel gene |
| *CHMP5* | 1.09 | 3.58E-02 | Novel gene |
| *KL* | 1.09 | 3.58E-02 | Reported on respiratory-related diseases |
| *C2CD2* | 1.09 | 3.59E-02 | Reported gene on tuberculosis |
| *CLK4* | 1.09 | 3.59E-02 | Novel gene |
| *AGTRAP* | 1.09 | 3.59E-02 | Novel gene |
| *FLVCR1* | 1.09 | 3.60E-02 | Novel gene |
| *SCAPER* | 1.09 | 3.60E-02 | Novel gene |
| *LAMP3* | 1.09 | 3.60E-02 | Novel gene |
| *SMARCD3* | 1.09 | 3.60E-02 | Novel gene |
| *MKL1* | 1.08 | 3.61E-02 | Novel gene |
| *ACOT1* | 1.08 | 3.62E-02 | Novel gene |
| *3-Mar* | 1.08 | 3.62E-02 | Novel gene |
| *SUMF1* | 1.08 | 3.62E-02 | Novel gene |
| *ANXA5* | 1.07 | 3.66E-02 | Reported on respiratory-related diseases |
| *ZNF266* | 1.07 | 3.66E-02 | Novel gene |
| *TIGA1* | 1.07 | 3.69E-02 | Novel gene |
| *ZNF669* | 1.07 | 3.70E-02 | Novel gene |
| *RPS5* | 1.07 | 3.71E-02 | Novel gene |
| *TMEM180* | 1.06 | 3.73E-02 | Novel gene |
| *RAB11FIP4* | 1.06 | 3.73E-02 | Novel gene |
| *PLA2G7* | 1.06 | 3.74E-02 | Novel gene |
| *TUBGCP4* | 1.06 | 3.75E-02 | Novel gene |
| *TMC2* | 1.06 | 3.76E-02 | Novel gene |
| *NPAL2* | 1.05 | 3.77E-02 | Novel gene |
| *IQCE* | 1.05 | 3.77E-02 | Novel gene |
| *PHKG2* | 1.05 | 3.77E-02 | Novel gene |
| *SPRY2* | 1.05 | 3.77E-02 | Novel gene |
| *ORMDL3* | 1.05 | 3.78E-02 | Reported on respiratory-related diseases |
| *UGT2B7* | 1.05 | 3.79E-02 | Novel gene |
| *UPF3A* | 1.05 | 3.79E-02 | Novel gene |
| *RNF122* | 1.05 | 3.79E-02 | Novel gene |
| *PUS1* | 1.05 | 3.80E-02 | Novel gene |
| *TIPARP* | 1.05 | 3.80E-02 | Reported on respiratory-related diseases |
| *WDR34* | 1.04 | 3.81E-02 | Novel gene |
| *RYR1* | 1.04 | 3.82E-02 | Reported on respiratory-related diseases |
| *PDGFB* | 1.04 | 3.82E-02 | Reported gene on lung-related diseases |
| *TMED5* | 1.04 | 3.84E-02 | Novel gene |
| *RNF40* | 1.04 | 3.85E-02 | Novel gene |
| *TIGD5* | 1.04 | 3.86E-02 | Novel gene |
| *MC1R* | 1.03 | 3.87E-02 | Novel gene |
| *ZNF818* | 1.03 | 3.88E-02 | Novel gene |
| *NCF1C* | 1.03 | 3.89E-02 | Novel gene |
| *LY6G6C* | 1.03 | 3.89E-02 | Novel gene |
| *DNAJC7* | 1.03 | 3.90E-02 | Novel gene |
| *RHOT2* | 1.03 | 3.91E-02 | Novel gene |
| *RPL32P3* | 1.03 | 3.91E-02 | Novel gene |
| *TACSTD1* | 1.02 | 3.92E-02 | Novel gene |
| *SPOCK2* | 1.02 | 3.93E-02 | Novel gene |
| *RBM8A* | 1.02 | 3.94E-02 | Novel gene |
| *TRIP13* | 1.02 | 3.94E-02 | Novel gene |
| *FAM89B* | 1.02 | 3.94E-02 | Novel gene |
| *FDX1* | 1.02 | 3.95E-02 | Reported gene on lung-related diseases |
| *LIG3* | 1.01 | 3.98E-02 | Novel gene |
| *SNORA65* | 1.01 | 3.99E-02 | Novel gene |
| *DCTD* | 1.01 | 3.99E-02 | Novel gene |
| *ZNF248* | 1.01 | 4.00E-02 | Novel gene |
| *TMEM169* | 1.00 | 4.02E-02 | Novel gene |
| *MAP1S* | 1.00 | 4.03E-02 | Novel gene |
| *RNF25* | 1.00 | 4.03E-02 | Novel gene |
| *MYLK2* | 1.00 | 4.03E-02 | Novel gene |
| *ARFGAP1* | 1.00 | 4.04E-02 | Novel gene |
| *WDR4* | 1.00 | 4.07E-02 | Novel gene |
| *ICA1* | 0.99 | 4.07E-02 | Novel gene |
| *ZNF10* | 0.99 | 4.08E-02 | Novel gene |
| *H2AFY* | 0.99 | 4.10E-02 | Novel gene |
| *BAT2* | 0.99 | 4.11E-02 | Novel gene |
| *OTUB1* | 0.99 | 4.12E-02 | Novel gene |
| *RUFY1* | 0.98 | 4.13E-02 | Reported gene on lung-related diseases |
| *ALDH8A1* | 0.98 | 4.14E-02 | Novel gene |
| *HS1BP3* | 0.98 | 4.14E-02 | Novel gene |
| *TNS4* | 0.98 | 4.16E-02 | Novel gene |
| *SELS* | 0.98 | 4.16E-02 | Novel gene |
| *PLXNA3* | 0.98 | 4.18E-02 | Novel gene |
| *ADAMTS19* | 0.98 | 4.18E-02 | Novel gene |
| *PRKCD* | 0.97 | 4.19E-02 | Novel gene |
| *HK3* | 0.97 | 4.19E-02 | Novel gene |
| *DTWD2* | 0.97 | 4.19E-02 | Novel gene |
| *ZNF502* | 0.97 | 4.23E-02 | Novel gene |
| *SGK* | 0.97 | 4.23E-02 | Novel gene |
| *ZNF557* | 0.96 | 4.24E-02 | Novel gene |
| *TMCO4* | 0.96 | 4.25E-02 | Novel gene |
| *ZSCAN18* | 0.96 | 4.25E-02 | Novel gene |
| *CNPY3* | 0.96 | 4.25E-02 | Novel gene |
| *FAU* | 0.96 | 4.25E-02 | Novel gene |
| *MAN1B1* | 0.96 | 4.25E-02 | Novel gene |
| *HNRPM* | 0.96 | 4.26E-02 | Novel gene |
| *IBSP* | 0.96 | 4.27E-02 | Novel gene |
| *VEGFB* | 0.96 | 4.29E-02 | Novel gene |
| *DTX2* | 0.96 | 4.29E-02 | Novel gene |
| *HP* | 0.95 | 4.31E-02 | Novel gene |
| *ZDHHC12* | 0.95 | 4.31E-02 | Novel gene |
| *GRB2* | 0.95 | 4.32E-02 | Novel gene |
| *ZNF197* | 0.95 | 4.32E-02 | Novel gene |
| *BRE* | 0.95 | 4.32E-02 | Novel gene |
| *SAMD10* | 0.95 | 4.33E-02 | Novel gene |
| *SLC23A1* | 0.95 | 4.33E-02 | Novel gene |
| *TUG1* | 0.95 | 4.34E-02 | Novel gene |
| *SCRN1* | 0.95 | 4.35E-02 | Novel gene |
| *USP42* | 0.95 | 4.36E-02 | Novel gene |
| *TNFRSF10A* | 0.95 | 4.36E-02 | Novel gene |
| *TULP3* | 0.94 | 4.36E-02 | Novel gene |
| *MRPS16* | 0.94 | 4.36E-02 | Novel gene |
| *C1QC* | 0.94 | 4.37E-02 | Novel gene |
| *VPREB3* | 0.94 | 4.37E-02 | Novel gene |
| *MRPL16* | 0.94 | 4.37E-02 | Novel gene |
| *EXT2* | 0.94 | 4.39E-02 | Novel gene |
| *HCK* | 0.94 | 4.40E-02 | Reported on respiratory-related diseases |
| *CCS* | 0.94 | 4.40E-02 | Novel gene |
| *GAP43* | 0.94 | 4.41E-02 | Novel gene |
| *SPG7* | 0.94 | 4.42E-02 | Novel gene |
| *CYCS* | 0.94 | 4.42E-02 | Novel gene |
| *SERPINF1* | 0.93 | 4.43E-02 | Novel gene |
| *GMPPA* | 0.93 | 4.43E-02 | Novel gene |
| *CHD1L* | 0.93 | 4.43E-02 | Novel gene |
| *SLC35A5* | 0.93 | 4.43E-02 | Novel gene |
| *PPA2* | 0.93 | 4.44E-02 | Novel gene |
| *WASF2* | 0.93 | 4.44E-02 | Novel gene |
| *CFDP1* | 0.93 | 4.44E-02 | Reported gene on lung-related and respiratory-related diseases |
| *NADK* | 0.93 | 4.45E-02 | Reported gene on lung-related diseases |
| *PUSL1* | 0.93 | 4.46E-02 | Novel gene |
| *HIST1H4K* | 0.93 | 4.47E-02 | Novel gene |
| *RG9MTD3* | 0.93 | 4.47E-02 | Novel gene |
| *TRIM72* | 0.93 | 4.47E-02 | Novel gene |
| *SNORD35A* | 0.93 | 4.48E-02 | Novel gene |
| *IL4I1* | 0.92 | 4.49E-02 | Novel gene |
| *ZNF660* | 0.92 | 4.50E-02 | Novel gene |
| *ZBTB32* | 0.92 | 4.50E-02 | Novel gene |
| *ZDHHC16* | 0.92 | 4.52E-02 | Novel gene |
| *IL32* | 0.92 | 4.53E-02 | Novel gene |
| *MEGF8* | 0.92 | 4.54E-02 | Novel gene |
| *RAB33A* | 0.92 | 4.54E-02 | Novel gene |
| *NSUN5* | 0.91 | 4.55E-02 | Novel gene |
| *RFC1* | 0.91 | 4.55E-02 | Novel gene |
| *ZNF559* | 0.91 | 4.56E-02 | Novel gene |
| *SPEF1* | 0.91 | 4.57E-02 | Novel gene |
| *CD276* | 0.91 | 4.59E-02 | Novel gene |
| *F8* | 0.91 | 4.60E-02 | Novel gene |
| *CALM2* | 0.90 | 4.63E-02 | Novel gene |
| *ENPP7* | 0.90 | 4.63E-02 | Novel gene |
| *CCDC18* | 0.90 | 4.63E-02 | Novel gene |
| *TRIM28* | 0.90 | 4.64E-02 | Novel gene |
| *ZNF766* | 0.90 | 4.64E-02 | Novel gene |
| *DRD3* | 0.90 | 4.64E-02 | Novel gene |
| *PRIM1* | 0.90 | 4.64E-02 | Novel gene |
| *ATP5A1* | 0.90 | 4.66E-02 | Novel gene |
| *URM1* | 0.89 | 4.69E-02 | Novel gene |
| *MEIS3P1* | 0.89 | 4.69E-02 | Novel gene |
| *CDK5RAP3* | 0.89 | 4.70E-02 | Reported gene on lung-related diseases |
| *DNM2* | 0.89 | 4.70E-02 | Novel gene |
| *YDJC* | 0.89 | 4.71E-02 | Novel gene |
| *OR52K2* | 0.89 | 4.72E-02 | Reported gene on lung-related diseases |
| *IFIT1* | 0.89 | 4.72E-02 | Novel gene |
| *GALNT4* | 0.88 | 4.73E-02 | Novel gene |
| *KNTC1* | 0.88 | 4.73E-02 | Novel gene |
| *ACO1* | 0.88 | 4.74E-02 | Reported on respiratory-related diseases |
| *EGR2* | 0.88 | 4.76E-02 | Novel gene |
| *SLC26A9* | 0.88 | 4.76E-02 | Reported gene on lung-related diseases |
| *MOSPD1* | 0.88 | 4.78E-02 | Novel gene |
| *HLA-DRB6* | 0.87 | 4.80E-02 | Reported gene on tuberculosis and respiratory-related diseases |
| *LYPD2* | 0.87 | 4.81E-02 | Novel gene |
| *NUDT17* | 0.87 | 4.81E-02 | Novel gene |
| *HUWE1* | 0.87 | 4.81E-02 | Novel gene |
| *FFAR1* | 0.87 | 4.81E-02 | Novel gene |
| *RRM1* | 0.87 | 4.82E-02 | Novel gene |
| *SLC29A1* | 0.87 | 4.82E-02 | Novel gene |
| *IFI44* | 0.87 | 4.82E-02 | Reported on respiratory-related diseases |
| *TMEM65* | 0.87 | 4.82E-02 | Novel gene |
| *SPTLC1* | 0.87 | 4.83E-02 | Novel gene |
| *ADRB2* | 0.87 | 4.84E-02 | Reported gene on lung-related and respiratory-related diseases |
| *AMOT* | 0.87 | 4.84E-02 | Novel gene |
| *FBXO32* | 0.87 | 4.84E-02 | Novel gene |
| *LST1* | 0.87 | 4.85E-02 | Reported gene on lung-related diseases |
| *MED22* | 0.87 | 4.85E-02 | Novel gene |
| *TM9SF4* | 0.86 | 4.86E-02 | Reported on respiratory-related diseases |
| *TOM1* | 0.86 | 4.86E-02 | Novel gene |
| *RHOBTB2* | 0.86 | 4.89E-02 | Novel gene |
| *TMEM129* | 0.86 | 4.89E-02 | Novel gene |
| *ADCK5* | 0.86 | 4.90E-02 | Novel gene |
| *TKT* | 0.86 | 4.90E-02 | Novel gene |
| *SQLE* | 0.86 | 4.90E-02 | Novel gene |
| *TGOLN2* | 0.86 | 4.91E-02 | Novel gene |
| *ZNF160* | 0.86 | 4.92E-02 | Novel gene |
| *NFXL1* | 0.86 | 4.92E-02 | Novel gene |
| *PLA2G2D* | 0.86 | 4.92E-02 | Novel gene |
| *TMEM161B* | 0.85 | 4.94E-02 | Novel gene |
| *TRABD* | 0.85 | 4.94E-02 | Novel gene |
| *NADSYN1* | 0.85 | 4.94E-02 | Novel gene |
| *ANAPC1* | 0.85 | 4.94E-02 | Reported gene on lung-related and respiratory-related diseases |
| *MAPK8IP3* | 0.85 | 4.94E-02 | Novel gene |
| *BMP3* | 0.85 | 4.96E-02 | Novel gene |
| *CRYM* | 0.85 | 4.96E-02 | Novel gene |
| *GTF3C1* | 0.85 | 4.97E-02 | Novel gene |
| *DLG5* | 0.85 | 4.98E-02 | Novel gene |
| *RPS23* | NA | 7.88E-07 | Novel gene |
| *XYLT1* | NA | 7.88E-07 | Novel gene |
| *XRCC6BP1* | NA | 7.88E-07 | Reported gene on lung-related diseases |
| *WDR41* | NA | 7.88E-07 | Reported gene on lung-related diseases |
| *VAV3* | NA | 7.88E-07 | Reported gene on lung-related and respiratory-related diseases |
| *VASH1* | NA | 7.88E-07 | Novel gene |
| *USMG5* | NA | 7.88E-07 | Novel gene |
| *TMEM176B* | NA | 7.88E-07 | Novel gene |
| *TMEM176A* | NA | 7.88E-07 | Reported on respiratory-related diseases |
| *TIMM10* | NA | 7.88E-07 | Novel gene |
| *TACSTD2* | NA | 7.88E-07 | Novel gene |
| *SMC6* | NA | 7.88E-07 | Novel gene |
| *RPS26L1* | NA | 7.88E-07 | Novel gene |
| *RPS26L* | NA | 7.88E-07 | Novel gene |
| *RPS26* | NA | 7.88E-07 | Reported on respiratory-related diseases |
| *HLA-DQB1* | NA | 7.88E-07 | Reported gene on tuberculosis, lung-related and respiratory-related diseases |
| *RIPK5* | NA | 7.88E-07 | Novel gene |
| *RAD51C* | NA | 7.88E-07 | Novel gene |
| *PTER* | NA | 7.88E-07 | Novel gene |
| *PEX6* | NA | 7.88E-07 | Novel gene |
| *NT5DC3* | NA | 7.88E-07 | Novel gene |
| *NT5C3L* | NA | 7.88E-07 | Novel gene |
| *MAPK8IP1* | NA | 7.88E-07 | Novel gene |
| *LRRC37A4* | NA | 7.88E-07 | Novel gene |
| *LPCAT2* | NA | 7.88E-07 | Reported gene on tuberculosis |
| *LILRA3* | NA | 7.88E-07 | Novel gene |
| *KCTD10* | NA | 7.88E-07 | Novel gene |
| *JUP* | NA | 7.88E-07 | Novel gene |
| *IRF5* | NA | 7.88E-07 | Novel gene |
| *ERAP2* | NA | 7.88E-07 | Novel gene |
| *ACTA2* | NA | 7.88E-07 | Reported gene on lung-related diseases |
| *CLEC4F* | NA | 7.88E-07 | Novel gene |
| *CLECL1* | NA | 7.88E-07 | Novel gene |
| *CHRM4* | NA | 7.88E-07 | Novel gene |
| *CHST13* | NA | 7.88E-07 | Novel gene |
| *CHURC1* | NA | 7.88E-07 | Novel gene |
| *GOLGB1* | NA | 7.88E-07 | Novel gene |
| *GSTT1* | NA | 7.88E-07 | Novel gene |
| *FAM118A* | NA | 7.88E-07 | Novel gene |

**Note:** Reported genes mean these genes have been documented to be associated with tuberculosis, lung-related and respiratory-related disease in the GWAS Catalog database; Novel genes mean these genes were not documented in the GWAS Catalog database. NA represents not applicable.
