# Supplementary material for: Integrative genomics analysis identifies promising SNPs and genes implicated in tuberculosis risk based on multiple omics datasets
Source: Aging (Albany NY). 2020 Oct 13;12(19):19173–220. doi: 10.18632/aging.103744 (PMC7732298; doi:10.18632/aging.103744)
Supplement: Supplementary Table 2 [file aging-12-103744-s003..docx]

**Supplementary Table 2.** **Significant pathways enriched by tuberculosis-associated genes (Gene set #1) identified from Sherlock Bayesian analysis from Dataset #3 in the discovery stage.**

| **Pathway ID** | **Database** | **Input number** | **Background number** | **P-Value** | **FDR** |
| --- | --- | --- | --- | --- | --- |
| R-HSA-1430728 | Reactome | 103 | 2075 | 2.38E-32 | 1.78E-28 |
| R-HSA-168256 | Reactome | 91 | 2096 | 1.28E-24 | 2.12E-21 |
| R-HSA-74160 | Reactome | 72 | 1448 | 9.94E-23 | 1.06E-19 |
| hsa01100 | KEGG PATHWAY | 70 | 1433 | 1.04E-21 | 7.75E-19 |
| R-HSA-392499 | Reactome | 79 | 2012 | 5.78E-19 | 3.94E-16 |
| R-HSA-73857 | Reactome | 62 | 1316 | 1.34E-18 | 8.33E-16 |
| R-HSA-212436 | Reactome | 57 | 1193 | 1.94E-17 | 7.63E-15 |
| R-HSA-597592 | Reactome | 61 | 1412 | 1.23E-16 | 4.60E-14 |
| R-HSA-1280215 | Reactome | 44 | 836 | 4.08E-15 | 9.86E-13 |
| R-HSA-162582 | Reactome | 81 | 2689 | 2.97E-13 | 5.70E-11 |
| R-HSA-5653656 | Reactome | 36 | 669 | 7.27E-13 | 1.36E-10 |
| R-HSA-1643685 | Reactome | 45 | 1049 | 1.78E-12 | 3.11E-10 |
| R-HSA-199991 | Reactome | 33 | 631 | 1.36E-11 | 2.17E-09 |
| R-HSA-449147 | Reactome | 31 | 619 | 1.57E-10 | 2.21E-08 |
| R-HSA-8953854 | Reactome | 32 | 667 | 2.21E-10 | 3.01E-08 |
| R-HSA-168249 | Reactome | 41 | 1043 | 2.21E-10 | 3.01E-08 |
| hsa05168 | KEGG PATHWAY | 26 | 492 | 1.64E-09 | 1.86E-07 |
| R-HSA-556833 | Reactome | 31 | 728 | 6.23E-09 | 6.05E-07 |
| R-HSA-1280218 | Reactome | 31 | 748 | 1.13E-08 | 1.09E-06 |
| R-HSA-76002 | Reactome | 18 | 260 | 1.15E-08 | 1.09E-06 |
| R-HSA-109582 | Reactome | 27 | 617 | 3.48E-08 | 2.86E-06 |
| R-HSA-3108232 | Reactome | 14 | 170 | 6.64E-08 | 5.14E-06 |
| R-HSA-2990846 | Reactome | 14 | 176 | 9.89E-08 | 7.26E-06 |
| hsa05418 | KEGG PATHWAY | 12 | 139 | 3.58E-07 | 2.29E-05 |
| R-HSA-1266738 | Reactome | 35 | 1082 | 4.40E-07 | 2.74E-05 |
| R-HSA-9006934 | Reactome | 21 | 458 | 5.58E-07 | 3.45E-05 |
| R-HSA-1640170 | Reactome | 25 | 629 | 6.14E-07 | 3.76E-05 |
| hsa05152 | KEGG PATHWAY | 13 | 179 | 7.41E-07 | 4.44E-05 |
| R-HSA-69278 | Reactome | 22 | 525 | 1.26E-06 | 7.04E-05 |
| hsa04625 | KEGG PATHWAY | 10 | 104 | 1.37E-06 | 7.59E-05 |
| hsa05200 | KEGG PATHWAY | 22 | 530 | 1.46E-06 | 8.04E-05 |
| hsa04015 | KEGG PATHWAY | 13 | 210 | 3.92E-06 | 1.98E-04 |
| R-HSA-71387 | Reactome | 15 | 288 | 5.38E-06 | 2.67E-04 |
| R-HSA-211859 | Reactome | 13 | 220 | 6.31E-06 | 2.97E-04 |
| R-HSA-1852241 | Reactome | 15 | 294 | 6.81E-06 | 3.15E-04 |
| R-HSA-114608 | Reactome | 10 | 128 | 7.68E-06 | 3.53E-04 |
| PWY-7210 | BioCyc | 5 | 19 | 8.92E-06 | 3.93E-04 |
| R-HSA-446203 | Reactome | 15 | 304 | 9.94E-06 | 4.35E-04 |
| R-HSA-68886 | Reactome | 17 | 384 | 1.01E-05 | 4.41E-04 |
| R-HSA-76005 | Reactome | 10 | 133 | 1.05E-05 | 4.47E-04 |
| hsa05322 | KEGG PATHWAY | 10 | 133 | 1.05E-05 | 4.47E-04 |
| hsa00240 | KEGG PATHWAY | 7 | 57 | 1.25E-05 | 5.16E-04 |
| hsa05143 | KEGG PATHWAY | 6 | 37 | 1.27E-05 | 5.22E-04 |
| hsa05225 | KEGG PATHWAY | 11 | 168 | 1.31E-05 | 5.37E-04 |
| P00006 | PANTHER | 9 | 108 | 1.35E-05 | 5.49E-04 |
| R-HSA-382551 | Reactome | 24 | 720 | 1.76E-05 | 7.01E-04 |
| hsa05034 | KEGG PATHWAY | 11 | 180 | 2.40E-05 | 9.22E-04 |
| R-HSA-5663205 | Reactome | 16 | 372 | 2.56E-05 | 9.68E-04 |
| R-HSA-196854 | Reactome | 11 | 186 | 3.19E-05 | 1.15E-03 |
| R-HSA-2262752 | Reactome | 17 | 422 | 3.20E-05 | 1.15E-03 |
| R-HSA-8953897 | Reactome | 19 | 516 | 3.65E-05 | 1.26E-03 |
| hsa04020 | KEGG PATHWAY | 11 | 193 | 4.39E-05 | 1.49E-03 |
| R-HSA-6798695 | Reactome | 18 | 478 | 4.40E-05 | 1.49E-03 |
| R-HSA-913531 | Reactome | 11 | 194 | 4.59E-05 | 1.55E-03 |
| R-HSA-157118 | Reactome | 12 | 231 | 4.71E-05 | 1.58E-03 |
| R-HSA-199992 | Reactome | 7 | 72 | 4.98E-05 | 1.66E-03 |
| hsa00982 | KEGG PATHWAY | 7 | 72 | 4.98E-05 | 1.66E-03 |
| hsa04217 | KEGG PATHWAY | 10 | 162 | 5.16E-05 | 1.71E-03 |
| hsa01524 | KEGG PATHWAY | 7 | 73 | 5.40E-05 | 1.78E-03 |
| hsa04650 | KEGG PATHWAY | 9 | 131 | 5.60E-05 | 1.84E-03 |
| R-HSA-449836 | Reactome | 13 | 275 | 5.81E-05 | 1.89E-03 |
| hsa04141 | KEGG PATHWAY | 10 | 165 | 5.96E-05 | 1.92E-03 |
| hsa04660 | KEGG PATHWAY | 8 | 103 | 6.46E-05 | 2.05E-03 |
| hsa05164 | KEGG PATHWAY | 10 | 167 | 6.56E-05 | 2.07E-03 |
| R-HSA-72203 | Reactome | 12 | 241 | 6.93E-05 | 2.18E-03 |
| hsa04144 | KEGG PATHWAY | 12 | 244 | 7.76E-05 | 2.43E-03 |
| R-HSA-194138 | Reactome | 8 | 106 | 7.80E-05 | 2.43E-03 |
| R-HSA-71291 | Reactome | 15 | 367 | 7.85E-05 | 2.44E-03 |
| R-HSA-5693538 | Reactome | 9 | 138 | 8.17E-05 | 2.51E-03 |
| R-HSA-983169 | Reactome | 15 | 370 | 8.56E-05 | 2.59E-03 |
| hsa00983 | KEGG PATHWAY | 7 | 79 | 8.58E-05 | 2.59E-03 |
| R-HSA-432722 | Reactome | 6 | 54 | 8.76E-05 | 2.60E-03 |
| R-HSA-6785807 | Reactome | 8 | 108 | 8.82E-05 | 2.60E-03 |
| R-HSA-69620 | Reactome | 13 | 289 | 9.36E-05 | 2.68E-03 |
| R-HSA-5663202 | Reactome | 15 | 374 | 9.59E-05 | 2.68E-03 |
| R-HSA-2151201 | Reactome | 6 | 55 | 9.61E-05 | 2.68E-03 |
| R-HSA-1483257 | Reactome | 11 | 212 | 9.75E-05 | 2.71E-03 |
| hsa00480 | KEGG PATHWAY | 6 | 56 | 1.05E-04 | 2.91E-03 |
| hsa04024 | KEGG PATHWAY | 11 | 214 | 1.05E-04 | 2.91E-03 |
| P04372 | PANTHER | 4 | 17 | 1.07E-04 | 2.93E-03 |
| hsa04060 | KEGG PATHWAY | 13 | 294 | 1.10E-04 | 3.00E-03 |
| P00018 | PANTHER | 8 | 114 | 1.25E-04 | 3.38E-03 |
| R-HSA-975957 | Reactome | 8 | 115 | 1.33E-04 | 3.55E-03 |
| R-HSA-927802 | Reactome | 8 | 115 | 1.33E-04 | 3.55E-03 |
| R-HSA-69239 | Reactome | 8 | 118 | 1.57E-04 | 4.11E-03 |
| R-HSA-162906 | Reactome | 11 | 227 | 1.72E-04 | 4.42E-03 |
| R-HSA-199977 | Reactome | 9 | 154 | 1.79E-04 | 4.50E-03 |
| R-HSA-168898 | Reactome | 9 | 154 | 1.79E-04 | 4.50E-03 |
| hsa05160 | KEGG PATHWAY | 9 | 155 | 1.87E-04 | 4.63E-03 |
| R-HSA-9007101 | Reactome | 8 | 123 | 2.05E-04 | 4.95E-03 |
| R-HSA-194315 | Reactome | 16 | 449 | 2.09E-04 | 5.04E-03 |
| R-HSA-4839726 | Reactome | 12 | 273 | 2.11E-04 | 5.04E-03 |
| R-HSA-3247509 | Reactome | 12 | 273 | 2.11E-04 | 5.04E-03 |
| R-HSA-3700989 | Reactome | 14 | 359 | 2.14E-04 | 5.10E-03 |
| P00005 | PANTHER | 9 | 158 | 2.15E-04 | 5.10E-03 |
| hsa05321 | KEGG PATHWAY | 6 | 65 | 2.24E-04 | 5.29E-03 |
| R-HSA-69242 | Reactome | 9 | 160 | 2.35E-04 | 5.36E-03 |
| R-HSA-1592230 | Reactome | 7 | 94 | 2.35E-04 | 5.36E-03 |
| R-HSA-69306 | Reactome | 8 | 126 | 2.39E-04 | 5.45E-03 |
| PWY-4061 | BioCyc | 4 | 22 | 2.56E-04 | 5.73E-03 |
| R-HSA-375280 | Reactome | 5 | 42 | 2.56E-04 | 5.73E-03 |
| R-HSA-6782315 | Reactome | 5 | 42 | 2.56E-04 | 5.73E-03 |
| R-HSA-204005 | Reactome | 6 | 68 | 2.81E-04 | 6.19E-03 |
| hsa04664 | KEGG PATHWAY | 6 | 68 | 2.81E-04 | 6.19E-03 |
| R-HSA-4420097 | Reactome | 7 | 97 | 2.81E-04 | 6.19E-03 |
| R-HSA-425397 | Reactome | 5 | 43 | 2.83E-04 | 6.19E-03 |
| hsa05205 | KEGG PATHWAY | 10 | 203 | 2.97E-04 | 6.43E-03 |
| R-HSA-5693532 | Reactome | 9 | 166 | 3.04E-04 | 6.51E-03 |
| R-HSA-195721 | Reactome | 13 | 329 | 3.15E-04 | 6.69E-03 |
| R-HSA-5607764 | Reactome | 7 | 99 | 3.16E-04 | 6.71E-03 |
| R-HSA-5693567 | Reactome | 8 | 132 | 3.22E-04 | 6.81E-03 |
| P04393 | PANTHER | 6 | 70 | 3.25E-04 | 6.81E-03 |
| R-HSA-73894 | Reactome | 13 | 331 | 3.33E-04 | 6.96E-03 |
| R-HSA-9010553 | Reactome | 9 | 169 | 3.44E-04 | 7.13E-03 |
| R-HSA-3108214 | Reactome | 6 | 71 | 3.49E-04 | 7.19E-03 |
| R-HSA-174178 | Reactome | 6 | 71 | 3.49E-04 | 7.19E-03 |
| R-HSA-390696 | Reactome | 3 | 9 | 3.56E-04 | 7.28E-03 |
| R-HSA-425407 | Reactome | 11 | 249 | 3.67E-04 | 7.49E-03 |
| P00021 | PANTHER | 7 | 103 | 3.96E-04 | 7.95E-03 |
| R-HSA-72306 | Reactome | 7 | 103 | 3.96E-04 | 7.95E-03 |
| hsa05142 | KEGG PATHWAY | 7 | 103 | 3.96E-04 | 7.95E-03 |
| hsa04080 | KEGG PATHWAY | 13 | 338 | 4.03E-04 | 8.05E-03 |
| hsa04010 | KEGG PATHWAY | 12 | 295 | 4.13E-04 | 8.15E-03 |
| R-HSA-446652 | Reactome | 8 | 138 | 4.27E-04 | 8.31E-03 |
| P06959 | PANTHER | 9 | 175 | 4.38E-04 | 8.47E-03 |
| R-HSA-211945 | Reactome | 7 | 105 | 4.42E-04 | 8.52E-03 |
| PWY-7211 | BioCyc | 4 | 26 | 4.51E-04 | 8.63E-03 |
| hsa05214 | KEGG PATHWAY | 6 | 75 | 4.58E-04 | 8.67E-03 |
| R-HSA-376176 | Reactome | 10 | 216 | 4.73E-04 | 8.93E-03 |
| R-HSA-187037 | Reactome | 6 | 76 | 4.90E-04 | 9.10E-03 |
| hsa00980 | KEGG PATHWAY | 6 | 76 | 4.90E-04 | 9.10E-03 |
| hsa05144 | KEGG PATHWAY | 5 | 49 | 4.92E-04 | 9.10E-03 |
| hsa04621 | KEGG PATHWAY | 9 | 178 | 4.92E-04 | 9.10E-03 |
| R-HSA-499943 | Reactome | 4 | 28 | 5.81E-04 | 1.03E-02 |
| hsa04610 | KEGG PATHWAY | 6 | 79 | 5.93E-04 | 1.05E-02 |
| R-HSA-72163 | Reactome | 9 | 183 | 5.95E-04 | 1.05E-02 |
| R-HSA-983168 | Reactome | 12 | 308 | 5.95E-04 | 1.05E-02 |
| hsa04668 | KEGG PATHWAY | 7 | 112 | 6.36E-04 | 1.11E-02 |
| R-HSA-422475 | Reactome | 17 | 549 | 6.36E-04 | 1.11E-02 |
| R-HSA-948021 | Reactome | 9 | 185 | 6.41E-04 | 1.12E-02 |
| R-HSA-5218920 | Reactome | 4 | 29 | 6.54E-04 | 1.13E-02 |
| hsa05145 | KEGG PATHWAY | 7 | 113 | 6.68E-04 | 1.15E-02 |
| R-HSA-500792 | Reactome | 15 | 454 | 6.92E-04 | 1.19E-02 |
| hsa05204 | KEGG PATHWAY | 6 | 82 | 7.13E-04 | 1.21E-02 |
| R-HSA-71384 | Reactome | 3 | 12 | 7.17E-04 | 1.21E-02 |
| R-HSA-381340 | Reactome | 6 | 83 | 7.57E-04 | 1.26E-02 |
| R-HSA-72172 | Reactome | 9 | 191 | 7.96E-04 | 1.31E-02 |
| R-HSA-168643 | Reactome | 5 | 55 | 8.01E-04 | 1.31E-02 |
| R-HSA-1638091 | Reactome | 5 | 55 | 8.01E-04 | 1.31E-02 |
| R-HSA-1483255 | Reactome | 6 | 84 | 8.03E-04 | 1.31E-02 |
| hsa04150 | KEGG PATHWAY | 8 | 153 | 8.13E-04 | 1.33E-02 |
| R-HSA-195258 | Reactome | 12 | 321 | 8.41E-04 | 1.37E-02 |
| R-HSA-174143 | Reactome | 6 | 85 | 8.51E-04 | 1.38E-02 |
| R-HSA-453276 | Reactome | 6 | 85 | 8.51E-04 | 1.38E-02 |
| R-HSA-373076 | Reactome | 12 | 322 | 8.63E-04 | 1.40E-02 |
| hsa04722 | KEGG PATHWAY | 7 | 119 | 8.91E-04 | 1.43E-02 |
| R-HSA-447115 | Reactome | 5 | 57 | 9.31E-04 | 1.49E-02 |
| R-HSA-196849 | Reactome | 7 | 120 | 9.33E-04 | 1.49E-02 |
| hsa05135 | KEGG PATHWAY | 7 | 121 | 9.77E-04 | 1.55E-02 |
| R-HSA-5694530 | Reactome | 4 | 33 | 1.02E-03 | 1.59E-02 |
| R-HSA-1592389 | Reactome | 4 | 33 | 1.02E-03 | 1.59E-02 |
| R-HSA-69052 | Reactome | 6 | 89 | 1.07E-03 | 1.65E-02 |
| hsa05235 | KEGG PATHWAY | 6 | 89 | 1.07E-03 | 1.65E-02 |
| R-HSA-1630316 | Reactome | 7 | 123 | 1.07E-03 | 1.65E-02 |
| hsa05203 | KEGG PATHWAY | 9 | 201 | 1.12E-03 | 1.73E-02 |
| R-HSA-73772 | Reactome | 6 | 90 | 1.12E-03 | 1.73E-02 |
| R-HSA-877300 | Reactome | 6 | 90 | 1.12E-03 | 1.73E-02 |
| hsa05161 | KEGG PATHWAY | 8 | 163 | 1.20E-03 | 1.83E-02 |
| R-HSA-392517 | Reactome | 3 | 15 | 1.25E-03 | 1.88E-02 |
| P00056 | PANTHER | 5 | 62 | 1.32E-03 | 1.98E-02 |
| R-HSA-174084 | Reactome | 5 | 62 | 1.32E-03 | 1.98E-02 |
| R-HSA-166016 | Reactome | 7 | 128 | 1.33E-03 | 1.98E-02 |
| R-HSA-198933 | Reactome | 7 | 128 | 1.33E-03 | 1.98E-02 |
| P00057 | PANTHER | 11 | 294 | 1.35E-03 | 2.00E-02 |
| R-HSA-168638 | Reactome | 4 | 36 | 1.37E-03 | 2.00E-02 |
| R-HSA-156590 | Reactome | 4 | 36 | 1.37E-03 | 2.00E-02 |
| R-HSA-69481 | Reactome | 8 | 167 | 1.39E-03 | 2.00E-02 |
| hsa04022 | KEGG PATHWAY | 8 | 167 | 1.39E-03 | 2.00E-02 |
| R-HSA-3214815 | Reactome | 6 | 94 | 1.39E-03 | 2.00E-02 |
| hsa04666 | KEGG PATHWAY | 6 | 94 | 1.39E-03 | 2.00E-02 |
| R-HSA-2454202 | Reactome | 7 | 130 | 1.45E-03 | 2.07E-02 |
| hsa00230 | KEGG PATHWAY | 7 | 130 | 1.45E-03 | 2.07E-02 |
| R-HSA-397014 | Reactome | 9 | 209 | 1.45E-03 | 2.07E-02 |
| R-HSA-9607240 | Reactome | 10 | 252 | 1.46E-03 | 2.08E-02 |
| R-HSA-15869 | Reactome | 6 | 95 | 1.46E-03 | 2.08E-02 |
| R-HSA-168273 | Reactome | 7 | 131 | 1.51E-03 | 2.13E-02 |
| hsa04270 | KEGG PATHWAY | 7 | 132 | 1.58E-03 | 2.21E-02 |
| hsa04068 | KEGG PATHWAY | 7 | 132 | 1.58E-03 | 2.21E-02 |
| R-HSA-166520 | Reactome | 6 | 97 | 1.62E-03 | 2.25E-02 |
| R-HSA-211000 | Reactome | 7 | 133 | 1.64E-03 | 2.28E-02 |
| P00011 | PANTHER | 4 | 38 | 1.64E-03 | 2.28E-02 |
| R-HSA-76009 | Reactome | 4 | 38 | 1.64E-03 | 2.28E-02 |
| R-HSA-372790 | Reactome | 27 | 1170 | 1.70E-03 | 2.34E-02 |
| R-HSA-174154 | Reactome | 5 | 66 | 1.71E-03 | 2.34E-02 |
| R-HSA-4551638 | Reactome | 5 | 66 | 1.71E-03 | 2.34E-02 |
| R-HSA-5654738 | Reactome | 5 | 66 | 1.71E-03 | 2.34E-02 |
| R-HSA-3371453 | Reactome | 5 | 66 | 1.71E-03 | 2.34E-02 |
| R-HSA-1428517 | Reactome | 8 | 173 | 1.72E-03 | 2.34E-02 |
| R-HSA-202131 | Reactome | 3 | 17 | 1.72E-03 | 2.34E-02 |
| R-HSA-5676594 | Reactome | 3 | 17 | 1.72E-03 | 2.34E-02 |
| hsa03040 | KEGG PATHWAY | 7 | 135 | 1.78E-03 | 2.40E-02 |
| hsa04914 | KEGG PATHWAY | 6 | 99 | 1.78E-03 | 2.40E-02 |
| R-HSA-6802952 | Reactome | 5 | 67 | 1.82E-03 | 2.44E-02 |
| R-HSA-909733 | Reactome | 5 | 67 | 1.82E-03 | 2.44E-02 |
| R-HSA-162599 | Reactome | 7 | 136 | 1.85E-03 | 2.47E-02 |
| R-HSA-5668541 | Reactome | 6 | 100 | 1.87E-03 | 2.48E-02 |
| hsa04933 | KEGG PATHWAY | 6 | 100 | 1.87E-03 | 2.48E-02 |
| hsa04910 | KEGG PATHWAY | 7 | 137 | 1.93E-03 | 2.52E-02 |
| hsa04371 | KEGG PATHWAY | 7 | 137 | 1.93E-03 | 2.52E-02 |
| hsa05150 | KEGG PATHWAY | 5 | 68 | 1.94E-03 | 2.53E-02 |
| R-HSA-74158 | Reactome | 4 | 40 | 1.95E-03 | 2.54E-02 |
| R-HSA-749476 | Reactome | 4 | 40 | 1.95E-03 | 2.54E-02 |
| R-HSA-445355 | Reactome | 4 | 40 | 1.95E-03 | 2.54E-02 |
| R-HSA-9020702 | Reactome | 6 | 101 | 1.96E-03 | 2.54E-02 |
| hsa05166 | KEGG PATHWAY | 9 | 219 | 1.97E-03 | 2.54E-02 |
| R-HSA-1660514 | Reactome | 3 | 18 | 1.99E-03 | 2.54E-02 |
| hsa04915 | KEGG PATHWAY | 7 | 138 | 2.00E-03 | 2.56E-02 |
| R-HSA-9006931 | Reactome | 10 | 264 | 2.02E-03 | 2.58E-02 |
| R-HSA-2173782 | Reactome | 4 | 41 | 2.13E-03 | 2.70E-02 |
| R-HSA-5578749 | Reactome | 6 | 103 | 2.15E-03 | 2.73E-02 |
| R-HSA-5621481 | Reactome | 7 | 140 | 2.17E-03 | 2.74E-02 |
| R-HSA-168255 | Reactome | 7 | 141 | 2.25E-03 | 2.83E-02 |
| hsa04620 | KEGG PATHWAY | 6 | 104 | 2.26E-03 | 2.83E-02 |
| PWY-7184 | BioCyc | 3 | 19 | 2.28E-03 | 2.83E-02 |
| R-HSA-5218921 | Reactome | 3 | 19 | 2.28E-03 | 2.83E-02 |
| R-HSA-176407 | Reactome | 3 | 19 | 2.28E-03 | 2.83E-02 |
| R-HSA-176412 | Reactome | 3 | 19 | 2.28E-03 | 2.83E-02 |
| R-HSA-69017 | Reactome | 5 | 71 | 2.31E-03 | 2.85E-02 |
| R-HSA-174184 | Reactome | 5 | 71 | 2.31E-03 | 2.85E-02 |
| R-HSA-3781865 | Reactome | 7 | 143 | 2.43E-03 | 2.97E-02 |
| R-HSA-179419 | Reactome | 5 | 72 | 2.45E-03 | 2.99E-02 |
| hsa04940 | KEGG PATHWAY | 4 | 43 | 2.50E-03 | 3.04E-02 |
| R-HSA-141405 | Reactome | 3 | 20 | 2.60E-03 | 3.16E-02 |
| R-HSA-141430 | Reactome | 3 | 20 | 2.60E-03 | 3.16E-02 |
| R-HSA-8856828 | Reactome | 7 | 145 | 2.61E-03 | 3.17E-02 |
| hsa05167 | KEGG PATHWAY | 8 | 186 | 2.64E-03 | 3.20E-02 |
| R-HSA-156580 | Reactome | 6 | 108 | 2.70E-03 | 3.24E-02 |
| R-HSA-176409 | Reactome | 5 | 74 | 2.74E-03 | 3.29E-02 |
| R-HSA-73854 | Reactome | 6 | 109 | 2.82E-03 | 3.35E-02 |
| hsa04014 | KEGG PATHWAY | 9 | 232 | 2.86E-03 | 3.35E-02 |
| R-HSA-176814 | Reactome | 5 | 75 | 2.90E-03 | 3.35E-02 |
| hsa04072 | KEGG PATHWAY | 7 | 148 | 2.91E-03 | 3.35E-02 |
| P02721 | PANTHER | 2 | 5 | 2.94E-03 | 3.35E-02 |
| R-HSA-187706 | Reactome | 2 | 5 | 2.94E-03 | 3.35E-02 |
| R-HSA-187024 | Reactome | 2 | 5 | 2.94E-03 | 3.35E-02 |
| R-HSA-70350 | Reactome | 2 | 5 | 2.94E-03 | 3.35E-02 |
| PWY-6569 | BioCyc | 3 | 21 | 2.94E-03 | 3.35E-02 |
| R-HSA-73864 | Reactome | 6 | 110 | 2.94E-03 | 3.35E-02 |
| R-HSA-2559582 | Reactome | 6 | 110 | 2.94E-03 | 3.35E-02 |
| hsa04062 | KEGG PATHWAY | 8 | 190 | 2.99E-03 | 3.40E-02 |
| R-HSA-162587 | Reactome | 7 | 149 | 3.02E-03 | 3.42E-02 |
| hsa05133 | KEGG PATHWAY | 5 | 76 | 3.06E-03 | 3.46E-02 |
| R-HSA-5696398 | Reactome | 6 | 111 | 3.07E-03 | 3.47E-02 |
| hsa04930 | KEGG PATHWAY | 4 | 46 | 3.13E-03 | 3.53E-02 |
| P00004 | PANTHER | 6 | 112 | 3.20E-03 | 3.58E-02 |
| R-HSA-190236 | Reactome | 5 | 77 | 3.22E-03 | 3.59E-02 |
| R-HSA-168254 | Reactome | 7 | 152 | 3.35E-03 | 3.69E-02 |
| hsa00565 | KEGG PATHWAY | 4 | 47 | 3.37E-03 | 3.69E-02 |
| P00053 | PANTHER | 5 | 78 | 3.40E-03 | 3.71E-02 |
| hsa03010 | KEGG PATHWAY | 7 | 153 | 3.47E-03 | 3.79E-02 |
| R-HSA-1989781 | Reactome | 6 | 114 | 3.48E-03 | 3.79E-02 |
| R-HSA-3214858 | Reactome | 5 | 79 | 3.57E-03 | 3.85E-02 |
| R-HSA-176408 | Reactome | 5 | 79 | 3.57E-03 | 3.85E-02 |
| R-HSA-400206 | Reactome | 6 | 115 | 3.62E-03 | 3.88E-02 |
| P00002 | PANTHER | 3 | 23 | 3.71E-03 | 3.94E-02 |
| R-HSA-174048 | Reactome | 3 | 23 | 3.71E-03 | 3.94E-02 |
| R-HSA-9609507 | Reactome | 7 | 156 | 3.84E-03 | 3.94E-02 |
| R-HSA-6811440 | Reactome | 4 | 49 | 3.87E-03 | 3.94E-02 |
| SER-GLYSYN-PWY | BioCyc | 2 | 6 | 3.89E-03 | 3.94E-02 |
| R-HSA-8964208 | Reactome | 2 | 6 | 3.89E-03 | 3.94E-02 |
| R-HSA-187015 | Reactome | 2 | 6 | 3.89E-03 | 3.94E-02 |
| R-HSA-173623 | Reactome | 2 | 6 | 3.89E-03 | 3.94E-02 |
| hsa04510 | KEGG PATHWAY | 8 | 199 | 3.91E-03 | 3.96E-02 |
| R-HSA-2408522 | Reactome | 6 | 117 | 3.93E-03 | 3.97E-02 |
| R-HSA-2871837 | Reactome | 5 | 81 | 3.95E-03 | 3.98E-02 |
| R-HSA-5617833 | Reactome | 8 | 200 | 4.03E-03 | 4.05E-02 |
| R-HSA-5668599 | Reactome | 3 | 24 | 4.14E-03 | 4.05E-02 |
| R-HSA-450302 | Reactome | 3 | 24 | 4.14E-03 | 4.05E-02 |
| hsa00534 | KEGG PATHWAY | 3 | 24 | 4.14E-03 | 4.05E-02 |
| R-HSA-1483249 | Reactome | 4 | 50 | 4.14E-03 | 4.05E-02 |
| R-HSA-1168372 | Reactome | 5 | 82 | 4.15E-03 | 4.05E-02 |
| R-HSA-157579 | Reactome | 5 | 82 | 4.15E-03 | 4.05E-02 |
| hsa04662 | KEGG PATHWAY | 5 | 82 | 4.15E-03 | 4.05E-02 |
| R-HSA-6811442 | Reactome | 8 | 202 | 4.26E-03 | 4.13E-02 |
| R-HSA-6802957 | Reactome | 5 | 83 | 4.36E-03 | 4.22E-02 |
| R-HSA-2029480 | Reactome | 5 | 83 | 4.36E-03 | 4.22E-02 |
| hsa05014 | KEGG PATHWAY | 4 | 51 | 4.43E-03 | 4.27E-02 |
| R-HSA-5696399 | Reactome | 5 | 84 | 4.58E-03 | 4.39E-02 |
| R-HSA-179409 | Reactome | 3 | 25 | 4.59E-03 | 4.39E-02 |
| hsa04630 | KEGG PATHWAY | 7 | 162 | 4.68E-03 | 4.47E-02 |
| R-HSA-163200 | Reactome | 6 | 123 | 4.95E-03 | 4.65E-02 |
| R-HSA-162909 | Reactome | 6 | 123 | 4.95E-03 | 4.65E-02 |
| R-HSA-5652084 | Reactome | 2 | 7 | 4.96E-03 | 4.65E-02 |
| R-HSA-444473 | Reactome | 2 | 7 | 4.96E-03 | 4.65E-02 |
| R-HSA-170984 | Reactome | 2 | 7 | 4.96E-03 | 4.65E-02 |
| R-HSA-450531 | Reactome | 5 | 86 | 5.03E-03 | 4.67E-02 |
| R-HSA-912446 | Reactome | 5 | 86 | 5.03E-03 | 4.67E-02 |
| R-HSA-3371556 | Reactome | 5 | 86 | 5.03E-03 | 4.67E-02 |
| hsa05210 | KEGG PATHWAY | 5 | 86 | 5.03E-03 | 4.67E-02 |
| R-HSA-3238698 | Reactome | 3 | 26 | 5.08E-03 | 4.68E-02 |
| R-HSA-6803529 | Reactome | 3 | 26 | 5.08E-03 | 4.68E-02 |
| R-HSA-354192 | Reactome | 3 | 26 | 5.08E-03 | 4.68E-02 |
| R-HSA-76066 | Reactome | 3 | 26 | 5.08E-03 | 4.68E-02 |
| R-HSA-9006921 | Reactome | 3 | 26 | 5.08E-03 | 4.68E-02 |
| R-HSA-381426 | Reactome | 6 | 125 | 5.33E-03 | 4.90E-02 |

**Note:** Proportion of risk genes: these identified risk genes (Input number) accounted for the proportion of all genes in each pathway (Background number) enriched by these genes. FDR values were calculated by using the method of Benjamini-Hochberg false discovery rate (FDR) correction.
