# Supplementary material for: Integrative genomics analysis identifies promising SNPs and genes implicated in tuberculosis risk based on multiple omics datasets
Source: Aging (Albany NY). 2020 Oct 13;12(19):19173–220. doi: 10.18632/aging.103744 (PMC7732298; doi:10.18632/aging.103744)
Supplement: Supplementary Table 3 [file aging-12-103744-s004..docx]

**Supplementary Table 3. Significant GO-terms enriched by tuberculosis-associated genes (Gene set #1) identified from Sherlock Bayesian analysis of Dataset #3 in the discovery stage**

| **GO-terms ID** | **Input number** | **Background number** | **P-Value** | **FDR** |
| --- | --- | --- | --- | --- |
| GO:0005622 | 99 | 2228 | 1.64E-27 | 4.09E-24 |
| GO:0110165 | 108 | 2864 | 1.70E-24 | 2.12E-21 |
| GO:0009987 | 103 | 2852 | 5.08E-22 | 4.22E-19 |
| GO:0043226 | 79 | 2086 | 4.22E-18 | 2.26E-15 |
| GO:0044237 | 77 | 2027 | 9.89E-18 | 4.45E-15 |
| GO:0043227 | 77 | 2030 | 1.07E-17 | 4.45E-15 |
| GO:0043229 | 70 | 1863 | 5.80E-16 | 1.97E-13 |
| GO:0005488 | 78 | 2247 | 7.04E-16 | 2.19E-13 |
| GO:0003824 | 53 | 1162 | 1.67E-15 | 4.81E-13 |
| GO:0005737 | 64 | 1641 | 2.16E-15 | 5.71E-13 |
| GO:0005515 | 65 | 1688 | 2.29E-15 | 5.71E-13 |
| GO:0043231 | 62 | 1606 | 9.45E-15 | 2.14E-12 |
| GO:1901363 | 42 | 813 | 3.16E-14 | 6.56E-12 |
| GO:0005634 | 51 | 1182 | 4.54E-14 | 8.95E-12 |
| GO:0016020 | 54 | 1443 | 1.73E-12 | 3.09E-10 |
| GO:0046872 | 35 | 657 | 1.99E-12 | 3.31E-10 |
| GO:0097659 | 31 | 612 | 1.20E-10 | 1.83E-08 |
| GO:0005576 | 34 | 736 | 1.55E-10 | 2.21E-08 |
| GO:0044238 | 65 | 2249 | 4.07E-10 | 5.35E-08 |
| GO:0019222 | 41 | 1070 | 4.56E-10 | 5.78E-08 |
| GO:0048519 | 42 | 1152 | 1.15E-09 | 1.42E-07 |
| GO:0031224 | 35 | 843 | 1.23E-09 | 1.46E-07 |
| GO:0071704 | 69 | 2548 | 1.58E-09 | 1.82E-07 |
| GO:0051173 | 25 | 469 | 2.86E-09 | 3.14E-07 |
| GO:0010467 | 40 | 1111 | 4.04E-09 | 4.28E-07 |
| GO:0016787 | 28 | 593 | 4.12E-09 | 4.28E-07 |
| GO:0140110 | 19 | 278 | 5.52E-09 | 5.59E-07 |
| GO:0043233 | 31 | 725 | 5.68E-09 | 5.60E-07 |
| GO:0070013 | 30 | 712 | 1.37E-08 | 1.27E-06 |
| GO:0032991 | 28 | 639 | 1.89E-08 | 1.71E-06 |
| GO:0043230 | 23 | 444 | 2.01E-08 | 1.77E-06 |
| GO:0043168 | 23 | 454 | 2.97E-08 | 2.48E-06 |
| GO:0043167 | 35 | 962 | 2.98E-08 | 2.48E-06 |
| GO:0031982 | 28 | 667 | 4.48E-08 | 3.60E-06 |
| GO:0071944 | 31 | 812 | 6.67E-08 | 5.14E-06 |
| GO:0008104 | 21 | 403 | 7.51E-08 | 5.68E-06 |
| GO:0043228 | 26 | 606 | 9.00E-08 | 6.67E-06 |
| GO:0031974 | 28 | 697 | 1.07E-07 | 7.68E-06 |
| GO:0080090 | 43 | 1427 | 1.41E-07 | 9.98E-06 |
| GO:0140352 | 13 | 156 | 1.71E-07 | 1.19E-05 |
| GO:0097367 | 19 | 351 | 1.79E-07 | 1.22E-05 |
| GO:0044249 | 50 | 1833 | 2.55E-07 | 1.70E-05 |
| GO:0008152 | 62 | 2527 | 3.29E-07 | 2.16E-05 |
| GO:0070062 | 21 | 445 | 3.57E-07 | 2.29E-05 |
| GO:0000139 | 11 | 115 | 4.28E-07 | 2.69E-05 |
| GO:1903506 | 23 | 549 | 7.30E-07 | 4.41E-05 |
| GO:0043169 | 25 | 641 | 8.52E-07 | 5.00E-05 |
| GO:0016021 | 30 | 871 | 8.61E-07 | 5.00E-05 |
| GO:0010628 | 17 | 317 | 8.87E-07 | 5.07E-05 |
| GO:0005654 | 21 | 474 | 9.44E-07 | 5.31E-05 |
| GO:0005829 | 22 | 531 | 1.51E-06 | 8.16E-05 |
| GO:0016818 | 14 | 227 | 1.75E-06 | 9.36E-05 |
| GO:0005615 | 23 | 596 | 2.74E-06 | 1.43E-04 |
| GO:0031981 | 23 | 603 | 3.30E-06 | 1.68E-04 |
| GO:0098662 | 11 | 150 | 4.80E-06 | 2.39E-04 |
| GO:0031410 | 15 | 289 | 5.60E-06 | 2.74E-04 |
| GO:0032555 | 15 | 292 | 6.30E-06 | 2.97E-04 |
| GO:0036477 | 10 | 125 | 6.32E-06 | 2.97E-04 |
| GO:0003700 | 13 | 221 | 6.61E-06 | 3.07E-04 |
| GO:0015031 | 18 | 418 | 8.04E-06 | 3.62E-04 |
| GO:0000166 | 17 | 377 | 8.07E-06 | 3.62E-04 |
| GO:0008144 | 15 | 300 | 8.56E-06 | 3.79E-04 |
| GO:0010604 | 20 | 513 | 1.05E-05 | 4.47E-04 |
| GO:1903561 | 20 | 514 | 1.08E-05 | 4.53E-04 |
| GO:0016740 | 19 | 474 | 1.20E-05 | 4.97E-04 |
| GO:0016817 | 12 | 206 | 1.63E-05 | 6.55E-04 |
| GO:1903508 | 13 | 244 | 1.79E-05 | 7.06E-04 |
| GO:1903047 | 9 | 115 | 2.15E-05 | 8.39E-04 |
| GO:0044271 | 40 | 1586 | 2.35E-05 | 9.06E-04 |
| GO:0003676 | 19 | 501 | 2.49E-05 | 9.44E-04 |
| GO:0048471 | 8 | 91 | 2.84E-05 | 1.06E-03 |
| GO:0097159 | 26 | 845 | 2.98E-05 | 1.10E-03 |
| GO:0044267 | 28 | 949 | 3.06E-05 | 1.12E-03 |
| GO:0016491 | 9 | 121 | 3.13E-05 | 1.14E-03 |
| GO:0036094 | 18 | 467 | 3.29E-05 | 1.17E-03 |
| GO:1901576 | 45 | 1904 | 3.40E-05 | 1.20E-03 |
| GO:0097708 | 13 | 261 | 3.49E-05 | 1.22E-03 |
| GO:0043170 | 57 | 2637 | 3.68E-05 | 1.26E-03 |
| GO:0012505 | 21 | 633 | 6.18E-05 | 1.98E-03 |
| GO:0031328 | 13 | 285 | 8.19E-05 | 2.51E-03 |
| GO:0031323 | 32 | 1229 | 8.63E-05 | 2.59E-03 |
| GO:0043005 | 10 | 173 | 8.66E-05 | 2.59E-03 |
| GO:1905114 | 8 | 108 | 8.82E-05 | 2.60E-03 |
| GO:0016462 | 12 | 248 | 8.98E-05 | 2.61E-03 |
| GO:0005215 | 11 | 210 | 9.00E-05 | 2.61E-03 |
| GO:0005694 | 9 | 140 | 9.06E-05 | 2.61E-03 |
| GO:0036211 | 25 | 857 | 9.52E-05 | 2.68E-03 |
| GO:0060070 | 6 | 55 | 9.61E-05 | 2.68E-03 |
| GO:0070161 | 7 | 82 | 1.07E-04 | 2.92E-03 |
| GO:0005794 | 11 | 216 | 1.14E-04 | 3.08E-03 |
| GO:0005886 | 22 | 726 | 1.47E-04 | 3.88E-03 |
| GO:0004674 | 8 | 117 | 1.49E-04 | 3.90E-03 |
| GO:0099080 | 10 | 188 | 1.65E-04 | 4.28E-03 |
| GO:0098772 | 13 | 307 | 1.66E-04 | 4.28E-03 |
| GO:0008324 | 8 | 120 | 1.75E-04 | 4.45E-03 |
| GO:0010256 | 6 | 62 | 1.76E-04 | 4.46E-03 |
| GO:0022890 | 8 | 121 | 1.85E-04 | 4.60E-03 |
| GO:0042277 | 5 | 39 | 1.87E-04 | 4.63E-03 |
| GO:0045184 | 14 | 356 | 1.97E-04 | 4.82E-03 |
| GO:0097747 | 3 | 7 | 1.98E-04 | 4.82E-03 |
| GO:0120036 | 10 | 193 | 2.02E-04 | 4.90E-03 |
| GO:0044085 | 16 | 452 | 2.25E-04 | 5.29E-03 |
| GO:2000377 | 5 | 41 | 2.31E-04 | 5.36E-03 |
| GO:0042995 | 12 | 276 | 2.32E-04 | 5.36E-03 |
| GO:0098588 | 12 | 276 | 2.32E-04 | 5.36E-03 |
| GO:1901564 | 42 | 1894 | 2.33E-04 | 5.36E-03 |
| GO:0005856 | 13 | 320 | 2.44E-04 | 5.52E-03 |
| GO:0045732 | 5 | 43 | 2.83E-04 | 6.19E-03 |
| GO:0150034 | 5 | 43 | 2.83E-04 | 6.19E-03 |
| GO:0005524 | 11 | 242 | 2.91E-04 | 6.34E-03 |
| GO:0005102 | 11 | 243 | 3.01E-04 | 6.48E-03 |
| GO:0098794 | 5 | 44 | 3.12E-04 | 6.65E-03 |
| GO:0019899 | 13 | 330 | 3.24E-04 | 6.81E-03 |
| GO:0006643 | 4 | 24 | 3.43E-04 | 7.13E-03 |
| GO:0098576 | 3 | 9 | 3.56E-04 | 7.28E-03 |
| GO:0016070 | 32 | 1342 | 3.91E-04 | 7.90E-03 |
| GO:0010243 | 9 | 173 | 4.04E-04 | 8.05E-03 |
| GO:0098589 | 5 | 47 | 4.13E-04 | 8.15E-03 |
| GO:1901135 | 11 | 253 | 4.17E-04 | 8.18E-03 |
| GO:0005815 | 7 | 104 | 4.19E-04 | 8.18E-03 |
| GO:0031325 | 19 | 630 | 4.29E-04 | 8.31E-03 |
| GO:0017137 | 4 | 26 | 4.51E-04 | 8.63E-03 |
| GO:0034062 | 3 | 10 | 4.59E-04 | 8.67E-03 |
| GO:0046982 | 6 | 76 | 4.90E-04 | 9.10E-03 |
| GO:0005126 | 5 | 49 | 4.92E-04 | 9.10E-03 |
| GO:0033036 | 15 | 440 | 5.09E-04 | 9.35E-03 |
| GO:1901360 | 49 | 2412 | 5.12E-04 | 9.36E-03 |
| GO:0005739 | 11 | 260 | 5.19E-04 | 9.43E-03 |
| GO:0009891 | 13 | 350 | 5.51E-04 | 9.97E-03 |
| GO:0031090 | 16 | 493 | 5.61E-04 | 1.01E-02 |
| GO:0031128 | 3 | 11 | 5.79E-04 | 1.03E-02 |
| GO:0030674 | 4 | 28 | 5.81E-04 | 1.03E-02 |
| GO:0003677 | 14 | 401 | 6.18E-04 | 1.08E-02 |
| GO:0044798 | 4 | 29 | 6.54E-04 | 1.13E-02 |
| GO:0005516 | 4 | 29 | 6.54E-04 | 1.13E-02 |
| GO:0017076 | 12 | 315 | 7.19E-04 | 1.21E-02 |
| GO:0010557 | 12 | 315 | 7.19E-04 | 1.21E-02 |
| GO:0006464 | 22 | 821 | 7.34E-04 | 1.23E-02 |
| GO:0000323 | 6 | 83 | 7.57E-04 | 1.26E-02 |
| GO:0042176 | 6 | 83 | 7.57E-04 | 1.26E-02 |
| GO:0045893 | 11 | 273 | 7.63E-04 | 1.26E-02 |
| GO:0003723 | 8 | 155 | 8.80E-04 | 1.42E-02 |
| GO:0071840 | 35 | 1594 | 8.92E-04 | 1.43E-02 |
| GO:0016791 | 5 | 58 | 1.00E-03 | 1.58E-02 |
| GO:0031226 | 11 | 283 | 1.01E-03 | 1.59E-02 |
| GO:0016829 | 4 | 33 | 1.02E-03 | 1.59E-02 |
| GO:0060322 | 7 | 122 | 1.02E-03 | 1.59E-02 |
| GO:0015075 | 8 | 163 | 1.20E-03 | 1.83E-02 |
| GO:0043292 | 4 | 35 | 1.24E-03 | 1.88E-02 |
| GO:0005200 | 3 | 15 | 1.25E-03 | 1.88E-02 |
| GO:0061097 | 3 | 15 | 1.25E-03 | 1.88E-02 |
| GO:0098590 | 7 | 128 | 1.33E-03 | 1.98E-02 |
| GO:1901265 | 13 | 388 | 1.36E-03 | 2.00E-02 |
| GO:0019438 | 37 | 1761 | 1.38E-03 | 2.00E-02 |
| GO:0140096 | 15 | 488 | 1.39E-03 | 2.00E-02 |
| GO:0015630 | 8 | 167 | 1.39E-03 | 2.00E-02 |
| GO:0065007 | 75 | 4289 | 1.39E-03 | 2.00E-02 |
| GO:0044853 | 3 | 16 | 1.47E-03 | 2.09E-02 |
| GO:0042623 | 4 | 37 | 1.50E-03 | 2.12E-02 |
| GO:0017111 | 9 | 212 | 1.59E-03 | 2.23E-02 |
| GO:0006996 | 33 | 1535 | 1.72E-03 | 2.34E-02 |
| GO:0008134 | 6 | 99 | 1.78E-03 | 2.40E-02 |
| GO:1903311 | 4 | 39 | 1.79E-03 | 2.41E-02 |
| GO:0009100 | 6 | 100 | 1.87E-03 | 2.48E-02 |
| GO:1901698 | 8 | 176 | 1.90E-03 | 2.51E-02 |
| GO:0019538 | 24 | 1003 | 1.91E-03 | 2.51E-02 |
| GO:0030554 | 10 | 262 | 1.92E-03 | 2.51E-02 |
| GO:0030054 | 8 | 177 | 1.97E-03 | 2.54E-02 |
| GO:0016363 | 3 | 18 | 1.99E-03 | 2.54E-02 |
| GO:0016925 | 4 | 41 | 2.13E-03 | 2.70E-02 |
| GO:0043933 | 13 | 410 | 2.17E-03 | 2.74E-02 |
| GO:0030546 | 5 | 70 | 2.18E-03 | 2.75E-02 |
| GO:0007005 | 9 | 224 | 2.28E-03 | 2.83E-02 |
| GO:0016772 | 10 | 269 | 2.30E-03 | 2.85E-02 |
| GO:0006886 | 8 | 183 | 2.40E-03 | 2.94E-02 |
| GO:0019439 | 8 | 183 | 2.40E-03 | 2.94E-02 |
| GO:0006352 | 4 | 44 | 2.70E-03 | 3.24E-02 |
| GO:0098813 | 4 | 44 | 2.70E-03 | 3.24E-02 |
| GO:0005783 | 8 | 188 | 2.81E-03 | 3.35E-02 |
| GO:0006807 | 53 | 2898 | 2.91E-03 | 3.35E-02 |
| GO:0071243 | 2 | 5 | 2.94E-03 | 3.35E-02 |
| GO:0048532 | 2 | 5 | 2.94E-03 | 3.35E-02 |
| GO:0016303 | 2 | 5 | 2.94E-03 | 3.35E-02 |
| GO:0061641 | 2 | 5 | 2.94E-03 | 3.35E-02 |
| GO:0005777 | 3 | 21 | 2.94E-03 | 3.35E-02 |
| GO:1903749 | 3 | 21 | 2.94E-03 | 3.35E-02 |
| GO:0016810 | 3 | 21 | 2.94E-03 | 3.35E-02 |
| GO:1903037 | 4 | 46 | 3.13E-03 | 3.53E-02 |
| GO:0009893 | 17 | 642 | 3.15E-03 | 3.54E-02 |
| GO:0034613 | 10 | 282 | 3.19E-03 | 3.57E-02 |
| GO:0018130 | 34 | 1664 | 3.24E-03 | 3.60E-02 |
| GO:0009628 | 8 | 193 | 3.28E-03 | 3.63E-02 |
| GO:0015293 | 3 | 22 | 3.31E-03 | 3.65E-02 |
| GO:0052126 | 3 | 22 | 3.31E-03 | 3.65E-02 |
| GO:0098857 | 4 | 47 | 3.37E-03 | 3.69E-02 |
| GO:0016310 | 20 | 819 | 3.52E-03 | 3.82E-02 |
| GO:1990904 | 5 | 79 | 3.57E-03 | 3.85E-02 |
| GO:0098657 | 5 | 79 | 3.57E-03 | 3.85E-02 |
| GO:0005085 | 4 | 48 | 3.61E-03 | 3.88E-02 |
| GO:0000423 | 3 | 23 | 3.71E-03 | 3.94E-02 |
| GO:1903900 | 3 | 23 | 3.71E-03 | 3.94E-02 |
| GO:0043902 | 5 | 80 | 3.76E-03 | 3.94E-02 |
| GO:0060255 | 35 | 1748 | 3.87E-03 | 3.94E-02 |
| GO:0050772 | 2 | 6 | 3.89E-03 | 3.94E-02 |
| GO:1905517 | 2 | 6 | 3.89E-03 | 3.94E-02 |
| GO:0031593 | 2 | 6 | 3.89E-03 | 3.94E-02 |
| GO:0080171 | 2 | 6 | 3.89E-03 | 3.94E-02 |
| GO:0032994 | 2 | 6 | 3.89E-03 | 3.94E-02 |
| GO:0032309 | 2 | 6 | 3.89E-03 | 3.94E-02 |
| GO:0005790 | 2 | 6 | 3.89E-03 | 3.94E-02 |
| GO:0008173 | 2 | 6 | 3.89E-03 | 3.94E-02 |
| GO:0051287 | 2 | 6 | 3.89E-03 | 3.94E-02 |
| GO:0019787 | 5 | 81 | 3.95E-03 | 3.98E-02 |
| GO:0051248 | 7 | 158 | 4.11E-03 | 4.05E-02 |
| GO:0000781 | 3 | 24 | 4.14E-03 | 4.05E-02 |
| GO:0031674 | 3 | 24 | 4.14E-03 | 4.05E-02 |
| GO:0005795 | 3 | 24 | 4.14E-03 | 4.05E-02 |
| GO:0016747 | 4 | 50 | 4.14E-03 | 4.05E-02 |
| GO:0045944 | 8 | 201 | 4.14E-03 | 4.05E-02 |
| GO:0043025 | 5 | 82 | 4.15E-03 | 4.05E-02 |
| GO:0009986 | 6 | 119 | 4.25E-03 | 4.13E-02 |
| GO:0042802 | 8 | 202 | 4.26E-03 | 4.13E-02 |
| GO:0098805 | 8 | 204 | 4.51E-03 | 4.34E-02 |
| GO:0033365 | 7 | 161 | 4.53E-03 | 4.35E-02 |
| GO:0070875 | 2 | 7 | 4.96E-03 | 4.65E-02 |
| GO:1901186 | 2 | 7 | 4.96E-03 | 4.65E-02 |
| GO:0042629 | 2 | 7 | 4.96E-03 | 4.65E-02 |
| GO:0018209 | 4 | 53 | 5.03E-03 | 4.67E-02 |
| GO:0030141 | 4 | 53 | 5.03E-03 | 4.67E-02 |
| GO:0120025 | 9 | 255 | 5.17E-03 | 4.75E-02 |
| GO:0060828 | 4 | 54 | 5.35E-03 | 4.90E-02 |

**Note:** Proportion of risk genes: these identified risk genes (Input number) accounted for the proportion of all genes in each pathway (Background number) enriched by these genes. FDR values were calculated by using the method of Benjamini-Hochberg false discovery rate (FDR) correction.
