# Supplementary material for: Integrative genomics analysis identifies promising SNPs and genes implicated in tuberculosis risk based on multiple omics datasets
Source: Aging (Albany NY). 2020 Oct 13;12(19):19173–220. doi: 10.18632/aging.103744 (PMC7732298; doi:10.18632/aging.103744)
Supplement: Supplementary Table 4 [file aging-12-103744-s005..docx]

**Supplementary Table 4. Significant KEGG and NHGRI GWAS Catalog disease enriched by tuberculosis-associated genes (Gene set #1) identified from Sherlock Bayesian analysis of Dataset #3 in the discovery stage**

| **Disease Terms** | **Database** | **Input number** | **Background number** | **P-Value** | **FDR** |
| --- | --- | --- | --- | --- | --- |
| Congenital disorders of metabolism | KEGG DISEASE | 39 | 695 | 2.26E-14 | 4.96E-12 |
| Obesity-related traits | NHGRI GWAS Catalog | 35 | 691 | 7.58E-12 | 1.23E-09 |
| Congenital malformations | KEGG DISEASE | 38 | 900 | 1.51E-10 | 2.21E-08 |
| Nervous system diseases | KEGG DISEASE | 33 | 859 | 2.19E-08 | 1.90E-06 |
| Inflammatory bowel disease | NHGRI GWAS Catalog | 16 | 227 | 5.90E-08 | 4.70E-06 |
| Neurodegenerative diseases | KEGG DISEASE | 17 | 337 | 1.96E-06 | 1.04E-04 |
| Ulcerative colitis | NHGRI GWAS Catalog | 11 | 138 | 2.26E-06 | 1.19E-04 |
| Other diseases | KEGG DISEASE | 13 | 204 | 2.91E-06 | 1.50E-04 |
| Crohn's disease | NHGRI GWAS Catalog | 13 | 220 | 6.31E-06 | 2.97E-04 |
| Congenital disorders of amino acid metabolism | KEGG DISEASE | 10 | 141 | 1.69E-05 | 6.77E-04 |
| Mental and behavioural disorders | KEGG DISEASE | 11 | 189 | 3.66E-05 | 1.26E-03 |
| Cardiovascular diseases | KEGG DISEASE | 15 | 342 | 3.67E-05 | 1.26E-03 |
| Mitochondrial diseases | KEGG DISEASE | 10 | 164 | 5.68E-05 | 1.86E-03 |
| Other congenital malformations | KEGG DISEASE | 15 | 357 | 5.84E-05 | 1.89E-03 |
| Immune system diseases | KEGG DISEASE | 13 | 278 | 6.45E-05 | 2.05E-03 |
| Celiac disease | NHGRI GWAS Catalog | 8 | 109 | 9.37E-05 | 2.68E-03 |
| Acne (severe) | NHGRI GWAS Catalog | 10 | 184 | 1.40E-04 | 3.73E-03 |
| Autosomal recessive mental retardation | KEGG DISEASE | 5 | 38 | 1.67E-04 | 4.30E-03 |
| Type 1 diabetes autoantibodies | NHGRI GWAS Catalog | 4 | 23 | 2.97E-04 | 6.43E-03 |
| Alzheimer's disease (cognitive decline) | NHGRI GWAS Catalog | 5 | 46 | 3.77E-04 | 7.66E-03 |
| Inflammatory bowel disease (IBD) | KEGG DISEASE | 3 | 10 | 4.59E-04 | 8.67E-03 |
| Asthma, susceptibility to | OMIM | 3 | 10 | 4.59E-04 | 8.67E-03 |
| Congenital disorders of cofactor/vitamin metabolism | KEGG DISEASE | 4 | 27 | 5.13E-04 | 9.36E-03 |
| Rheumatoid arthritis | NHGRI GWAS Catalog | 8 | 144 | 5.58E-04 | 1.01E-02 |
| Homeostasis model assessment of beta-cell function (interaction) | NHGRI GWAS Catalog | 3 | 12 | 7.17E-04 | 1.21E-02 |
| Schizophrenia | KEGG DISEASE | 3 | 13 | 8.74E-04 | 1.41E-02 |
| Allergies and autoimmune diseases | KEGG DISEASE | 6 | 93 | 1.32E-03 | 1.98E-02 |
| Height | NHGRI GWAS Catalog | 13 | 395 | 1.59E-03 | 2.22E-02 |
| Ankylosing spondylitis | NHGRI GWAS Catalog | 3 | 17 | 1.72E-03 | 2.34E-02 |
| Liver enzyme levels (gamma-glutamyl transferase) | NHGRI GWAS Catalog | 4 | 39 | 1.79E-03 | 2.41E-02 |
| Other immune system diseases | KEGG DISEASE | 4 | 45 | 2.91E-03 | 3.35E-02 |
| Leigh syndrome | OMIM | 2 | 5 | 2.94E-03 | 3.35E-02 |
| Freckles | NHGRI GWAS Catalog | 2 | 5 | 2.94E-03 | 3.35E-02 |
| Adverse response to chemotherapy (neutropenia/leucopenia) (cisplatin) | NHGRI GWAS Catalog | 2 | 5 | 2.94E-03 | 3.35E-02 |
| Fanconi anemia | KEGG DISEASE | 3 | 21 | 2.94E-03 | 3.35E-02 |
| Plasma omega-6 polyunsaturated fatty acid levels (dihomo-gamma-linolenic acid) | NHGRI GWAS Catalog | 3 | 22 | 3.31E-03 | 3.65E-02 |
| Congenital malformations of skin | KEGG DISEASE | 7 | 154 | 3.59E-03 | 3.86E-02 |
| Congenital malformations of the urinary system | KEGG DISEASE | 4 | 49 | 3.87E-03 | 3.94E-02 |
| Blood metabolite ratios | NHGRI GWAS Catalog | 4 | 49 | 3.87E-03 | 3.94E-02 |
| Progressive external ophthalmoplegia | KEGG DISEASE | 2 | 6 | 3.89E-03 | 3.94E-02 |
| Black vs. red hair color | NHGRI GWAS Catalog | 2 | 6 | 3.89E-03 | 3.94E-02 |
| Asthma (sex interaction) | NHGRI GWAS Catalog | 2 | 6 | 3.89E-03 | 3.94E-02 |
| Response to antipsychotic therapy (extrapyramidal side effects) | NHGRI GWAS Catalog | 2 | 6 | 3.89E-03 | 3.94E-02 |
| Congenital malformations of the musculoskeletal system | KEGG DISEASE | 8 | 201 | 4.14E-03 | 4.05E-02 |
| Eye disease | KEGG DISEASE | 9 | 249 | 4.46E-03 | 4.30E-02 |
| Plasma omega-6 polyunsaturated fatty acid levels (gamma-linolenic acid) | NHGRI GWAS Catalog | 3 | 25 | 4.59E-03 | 4.39E-02 |
| Senior-Loken syndrome | KEGG DISEASE | 2 | 7 | 4.96E-03 | 4.65E-02 |
| Stroke (ischemic) | NHGRI GWAS Catalog | 2 | 7 | 4.96E-03 | 4.65E-02 |
| Waist Circumference - Triglycerides (WC-TG) | NHGRI GWAS Catalog | 2 | 7 | 4.96E-03 | 4.65E-02 |
| Plasma omega-6 polyunsaturated fatty acid levels (linoleic acid) | NHGRI GWAS Catalog | 3 | 26 | 5.08E-03 | 4.68E-02 |

**Note:** Proportion of risk genes: these identified risk genes (Input number) accounted for the proportion of all genes in each pathway (Background number) enriched by these genes. FDR values were calculated by using the method of Benjamini-Hochberg false discovery rate (FDR) correction.
