# Supplementary material for: Integrative genomics analysis identifies promising SNPs and genes implicated in tuberculosis risk based on multiple omics datasets
Source: Aging (Albany NY). 2020 Oct 13;12(19):19173–220. doi: 10.18632/aging.103744 (PMC7732298; doi:10.18632/aging.103744)
Supplement: Supplementary Table 5 [file aging-12-103744-s006..docx]

**Supplementary Table 5**. **Sherlock Bayesian analysis identifies 311 (Gene set #2) genes as tuberculosis-associated risk genes from Dataset #4 in the replication stage**

| **Gene name** | **LBF** | **Simulated P value** | **GWAS Catalog documented genes** |
| --- | --- | --- | --- |
| *ENSG00000271828* | 5.66 | 1.11E-04 | Novel gene |
| *NSUN5P2* | 5.56 | 1.28E-04 | Novel gene |
| *CDPF1* | 5.52 | 1.42E-04 | Novel gene |
| *SETD9* | 5.48 | 1.66E-04 | Novel gene |
| *ANXA1* | 4.63 | 3.87E-04 | Novel gene |
| *ENSG00000254531* | 3.99 | 9.61E-04 | Novel gene |
| *RASSF4* | 3.96 | 9.96E-04 | Novel gene |
| *RASL11A* | 3.93 | 1.07E-03 | Novel gene |
| *SAMD12* | 3.87 | 1.13E-03 | Reported gene on respiratory-related diseases |
| *RAB44* | 3.71 | 1.35E-03 | Novel gene |
| *ALDH1A1* | 3.59 | 1.62E-03 | Novel gene |
| *MUCL1* | 3.58 | 1.64E-03 | Novel gene |
| *FAM216A* | 3.54 | 1.74E-03 | Novel gene |
| *MTG2* | 3.50 | 1.79E-03 | Novel gene |
| *PDK1* | 3.47 | 1.87E-03 | Novel gene |
| *LRRC37A* | 3.45 | 1.93E-03 | Novel gene |
| *PRPF19* | 3.41 | 2.04E-03 | Novel gene |
| *GPRC5C* | 3.35 | 2.22E-03 | Novel gene |
| *TCP11L2* | 3.20 | 2.68E-03 | Novel gene |
| *TMEM99* | 3.12 | 3.02E-03 | Novel gene |
| *ENSG00000262539* | 3.09 | 3.12E-03 | Novel gene |
| *KANSL1-AS1* | 3.07 | 3.19E-03 | Novel gene |
| *ENSG00000262500* | 3.05 | 3.26E-03 | Novel gene |
| *SLC8A1* | 3.02 | 3.37E-03 | Reported gene on lung-related and respiratory-related diseases |
| *KANSL1* | 3.02 | 3.37E-03 | Reported gene on lung-related and respiratory-related diseases |
| *BATF3* | 3.00 | 3.44E-03 | Novel gene |
| *SPATA20* | 2.98 | 3.51E-03 | Novel gene |
| *ASB16-AS1* | 2.90 | 3.86E-03 | Novel gene |
| *GPN3* | 2.85 | 4.16E-03 | Novel gene |
| *SLC25A46* | 2.84 | 4.20E-03 | Reported gene on respiratory-related diseases |
| *ZNF883* | 2.83 | 4.25E-03 | Novel gene |
| *MFNG* | 2.81 | 4.35E-03 | Novel gene |
| *ARL11* | 2.78 | 4.56E-03 | Novel gene |
| *CUBN* | 2.73 | 4.76E-03 | Novel gene |
| *GPATCH2L* | 2.72 | 4.80E-03 | Novel gene |
| *INPP5E* | 2.60 | 5.54E-03 | Novel gene |
| *ENSG00000223522* | 2.53 | 5.99E-03 | Novel gene |
| *FCGRT* | 2.52 | 6.05E-03 | Novel gene |
| *UPF3A* | 2.51 | 6.10E-03 | Novel gene |
| *ENSG00000235271* | 2.44 | 6.69E-03 | Novel gene |
| *MAP1S* | 2.44 | 6.70E-03 | Novel gene |
| *C1QA* | 2.44 | 6.72E-03 | Novel gene |
| *PKN3* | 2.41 | 6.88E-03 | Novel gene |
| *LRRC37A2* | 2.40 | 7.03E-03 | Novel gene |
| *RCN3* | 2.39 | 7.14E-03 | Novel gene |
| *PRDM4* | 2.38 | 7.23E-03 | Novel gene |
| *PROZ* | 2.32 | 7.78E-03 | Novel gene |
| *ENSG00000232837* | 2.31 | 7.80E-03 | Novel gene |
| *HOXB4* | 2.31 | 7.82E-03 | Novel gene |
| *USP47* | 2.28 | 8.07E-03 | Novel gene |
| *MORN1* | 2.28 | 8.09E-03 | Novel gene |
| *RITA1* | 2.28 | 8.12E-03 | Novel gene |
| *WAPAL* | 2.27 | 8.17E-03 | Novel gene |
| *ADCK2* | 2.26 | 8.29E-03 | Novel gene |
| *FAM86DP* | 2.25 | 8.46E-03 | Novel gene |
| *ENSG00000267480* | 2.25 | 8.46E-03 | Novel gene |
| *DHX57* | 2.24 | 8.48E-03 | Novel gene |
| *TDRKH* | 2.24 | 8.50E-03 | Novel gene |
| *CCDC147-AS1* | 2.22 | 8.78E-03 | Novel gene |
| *EPOR* | 2.21 | 8.91E-03 | Novel gene |
| *ENSG00000237721* | 2.19 | 9.10E-03 | Novel gene |
| *ENSG00000272373* | 2.17 | 9.28E-03 | Novel gene |
| *SPATA33* | 2.13 | 9.70E-03 | Novel gene |
| *IGLV8-61* | 2.13 | 9.72E-03 | Novel gene |
| *TMEM9B* | 2.12 | 9.82E-03 | Novel gene |
| *ENSG00000261000* | 2.12 | 9.89E-03 | Novel gene |
| *RNMT* | 2.11 | 1.00E-02 | Novel gene |
| *ENSG00000259293* | 2.05 | 1.07E-02 | Novel gene |
| *HOXB-AS1* | 2.05 | 1.07E-02 | Novel gene |
| *ENSG00000253982* | 2.03 | 1.10E-02 | Novel gene |
| *ZNF582-AS1* | 2.02 | 1.11E-02 | Novel gene |
| *STEAP3* | 2.01 | 1.12E-02 | Novel gene |
| *HIATL1* | 1.99 | 1.16E-02 | Novel gene |
| *CLN8* | 1.97 | 1.19E-02 | Novel gene |
| *ADAM10* | 1.96 | 1.21E-02 | Novel gene |
| *ENSG00000105520* | 1.93 | 1.25E-02 | Novel gene |
| *CES1* | 1.93 | 1.25E-02 | Novel gene |
| *CROCCP2* | 1.93 | 1.25E-02 | Novel gene |
| *ULK4* | 1.91 | 1.27E-02 | Reported gene on lung-related diseases |
| *ENSG00000226524* | 1.90 | 1.29E-02 | Novel gene |
| *TEC* | 1.90 | 1.30E-02 | Novel gene |
| *ENSG00000235888* | 1.89 | 1.31E-02 | Novel gene |
| *PCCB* | 1.86 | 1.36E-02 | Novel gene |
| *CPA5* | 1.85 | 1.38E-02 | Novel gene |
| *CDC16* | 1.85 | 1.38E-02 | Novel gene |
| *STIM1* | 1.85 | 1.38E-02 | Reported gene on lung-related diseases |
| *SMIM20* | 1.85 | 1.38E-02 | Novel gene |
| *HRH4* | 1.84 | 1.40E-02 | Novel gene |
| *BTBD9* | 1.83 | 1.42E-02 | Novel gene |
| *POM121C* | 1.83 | 1.42E-02 | Novel gene |
| *TMED5* | 1.81 | 1.45E-02 | Novel gene |
| *DND1P1* | 1.80 | 1.48E-02 | Novel gene |
| *CRHR1-IT1* | 1.79 | 1.49E-02 | Novel gene |
| *RHOG* | 1.79 | 1.49E-02 | Novel gene |
| *PMS2P3* | 1.77 | 1.54E-02 | Novel gene |
| *DPY19L3* | 1.77 | 1.55E-02 | Novel gene |
| *PHLDB3* | 1.77 | 1.55E-02 | Novel gene |
| *ENSG00000260841* | 1.75 | 1.57E-02 | Novel gene |
| *C6orf164* | 1.75 | 1.58E-02 | Novel gene |
| *ENSG00000271912* | 1.73 | 1.61E-02 | Novel gene |
| *SCAPER* | 1.73 | 1.62E-02 | Novel gene |
| *N4BP2* | 1.73 | 1.62E-02 | Novel gene |
| *ETFA* | 1.72 | 1.63E-02 | Novel gene |
| *ENSG00000267174* | 1.72 | 1.64E-02 | Novel gene |
| *FCHO1* | 1.72 | 1.64E-02 | Novel gene |
| *SNAPC4* | 1.72 | 1.64E-02 | Reported gene on lung-related diseases |
| *SEC16A* | 1.71 | 1.64E-02 | Novel gene |
| *ENSG00000263503* | 1.71 | 1.65E-02 | Novel gene |
| *FLVCR1-AS1* | 1.71 | 1.66E-02 | Novel gene |
| *RPS23* | 1.71 | 1.66E-02 | Novel gene |
| *TNNT3* | 1.70 | 1.67E-02 | Novel gene |
| *ZNRF1* | 1.70 | 1.68E-02 | Novel gene |
| *AP3B1* | 1.70 | 1.68E-02 | Reported gene on lung-related and respiratory-related diseases |
| *KIAA1143* | 1.70 | 1.68E-02 | Novel gene |
| *EYA3* | 1.69 | 1.69E-02 | Novel gene |
| *CMBL* | 1.69 | 1.70E-02 | Novel gene |
| *ENSG00000231050* | 1.68 | 1.71E-02 | Novel gene |
| *ENSG00000233360* | 1.67 | 1.72E-02 | Novel gene |
| *PFKP* | 1.67 | 1.73E-02 | Novel gene |
| *CLINT1* | 1.67 | 1.73E-02 | Novel gene |
| *LRRC37A4P* | 1.67 | 1.73E-02 | Novel gene |
| *CARD9* | 1.67 | 1.74E-02 | Reported gene on lung-related diseases |
| *CCDC122* | 1.65 | 1.77E-02 | Novel gene |
| *TKT* | 1.63 | 1.82E-02 | Novel gene |
| *ISL2* | 1.63 | 1.84E-02 | Novel gene |
| *ATP6AP1L* | 1.61 | 1.87E-02 | Novel gene |
| *UBE2D1* | 1.60 | 1.90E-02 | Novel gene |
| *ENSG00000226328* | 1.60 | 1.91E-02 | Novel gene |
| *SLC35A1* | 1.59 | 1.94E-02 | Novel gene |
| *KNOP1* | 1.59 | 1.94E-02 | Novel gene |
| *SESTD1* | 1.58 | 1.96E-02 | Novel gene |
| *KRT23* | 1.57 | 1.98E-02 | Novel gene |
| *ENSG00000262879* | 1.57 | 1.98E-02 | Novel gene |
| *ZNF502* | 1.57 | 1.99E-02 | Novel gene |
| *CAND2* | 1.55 | 2.03E-02 | Reported gene on lung-related diseases |
| *RRM1* | 1.54 | 2.04E-02 | Novel gene |
| *FLVCR1* | 1.54 | 2.05E-02 | Novel gene |
| *SLC48A1* | 1.53 | 2.07E-02 | Reported gene on respiratory-related diseases |
| *ENSG00000261253* | 1.53 | 2.07E-02 | Novel gene |
| *SDCCAG3* | 1.53 | 2.08E-02 | Novel gene |
| *LINC00944* | 1.52 | 2.10E-02 | Novel gene |
| *ENSG00000269690* | 1.51 | 2.13E-02 | Novel gene |
| *NPHP3* | 1.51 | 2.13E-02 | Novel gene |
| *NEK11* | 1.51 | 2.13E-02 | Novel gene |
| *TLR10* | 1.51 | 2.14E-02 | Reported gene on respiratory-related diseases |
| *GJA4* | 1.51 | 2.14E-02 | Novel gene |
| *ENSG00000230825* | 1.51 | 2.15E-02 | Novel gene |
| *PLEKHF2* | 1.50 | 2.17E-02 | Reported gene on respiratory-related diseases |
| *SPATA2L* | 1.50 | 2.19E-02 | Novel gene |
| *TBC1D2B* | 1.49 | 2.20E-02 | Novel gene |
| *HOXB3* | 1.48 | 2.24E-02 | Novel gene |
| *NIPAL2* | 1.47 | 2.28E-02 | Novel gene |
| *ACTBP8* | 1.46 | 2.29E-02 | Novel gene |
| *CDRT15P1* | 1.46 | 2.30E-02 | Reported gene on respiratory-related diseases |
| *NSUN4* | 1.46 | 2.31E-02 | Novel gene |
| *RAD21* | 1.45 | 2.32E-02 | Novel gene |
| *LIPC* | 1.45 | 2.34E-02 | Novel gene |
| *RBPMS2* | 1.44 | 2.36E-02 | Novel gene |
| *ENSG00000234426* | 1.44 | 2.36E-02 | Novel gene |
| *IGLV4-60* | 1.43 | 2.41E-02 | Novel gene |
| *MIF4GD* | 1.42 | 2.44E-02 | Novel gene |
| *ACCS* | 1.42 | 2.44E-02 | Novel gene |
| *ADAP1* | 1.41 | 2.44E-02 | Novel gene |
| *NTAN1* | 1.41 | 2.46E-02 | Novel gene |
| *ATXN3* | 1.41 | 2.46E-02 | Reported gene on lung-related diseases |
| *ENSG00000170092* | 1.41 | 2.46E-02 | Novel gene |
| *MRPL41* | 1.39 | 2.52E-02 | Novel gene |
| *HDAC10* | 1.39 | 2.53E-02 | Novel gene |
| *PLEKHG5* | 1.39 | 2.53E-02 | Novel gene |
| *QSOX2* | 1.38 | 2.56E-02 | Reported gene on lung-related diseases |
| *EIF4B* | 1.38 | 2.57E-02 | Novel gene |
| *ZNF197* | 1.37 | 2.57E-02 | Novel gene |
| *C6orf163* | 1.37 | 2.59E-02 | Novel gene |
| *ETS2* | 1.37 | 2.59E-02 | Novel gene |
| *DGUOK* | 1.36 | 2.62E-02 | Novel gene |
| *CTSG* | 1.36 | 2.64E-02 | Novel gene |
| *UEVLD* | 1.35 | 2.67E-02 | Novel gene |
| *SAYSD1* | 1.35 | 2.67E-02 | Novel gene |
| *IL6R* | 1.34 | 2.71E-02 | Reported gene on respiratory-related diseases |
| *SERPINA1* | 1.33 | 2.74E-02 | Novel gene |
| *SUPT5H* | 1.32 | 2.77E-02 | Novel gene |
| *CLTB* | 1.32 | 2.77E-02 | Novel gene |
| *KDELR2* | 1.31 | 2.80E-02 | Novel gene |
| *ENSG00000259865* | 1.30 | 2.82E-02 | Novel gene |
| *FOXRED1* | 1.30 | 2.83E-02 | Novel gene |
| *PIGCP1* | 1.30 | 2.84E-02 | Novel gene |
| *LIMD1* | 1.30 | 2.84E-02 | Novel gene |
| *SLC15A3* | 1.30 | 2.85E-02 | Novel gene |
| *SETD3* | 1.29 | 2.88E-02 | Novel gene |
| *CABLES2* | 1.28 | 2.90E-02 | Novel gene |
| *PHF13* | 1.28 | 2.91E-02 | Reported gene on lung-related and respiratory-related diseases |
| *NEK6* | 1.27 | 2.95E-02 | Reported gene on respiratory-related diseases |
| *ENSG00000269051* | 1.27 | 2.96E-02 | Novel gene |
| *NPHP4* | 1.26 | 2.98E-02 | Novel gene |
| *VWDE* | 1.26 | 3.00E-02 | Novel gene |
| *HBS1L* | 1.26 | 3.00E-02 | Novel gene |
| *ABCC3* | 1.25 | 3.03E-02 | Novel gene |
| *ENSG00000262370* | 1.25 | 3.05E-02 | Novel gene |
| *ENSG00000269896* | 1.25 | 3.05E-02 | Novel gene |
| *SPRYD4* | 1.24 | 3.09E-02 | Novel gene |
| *ZNF699* | 1.24 | 3.09E-02 | Novel gene |
| *ZNF266* | 1.24 | 3.09E-02 | Novel gene |
| *GTF2F1* | 1.23 | 3.11E-02 | Novel gene |
| *SH3RF3* | 1.23 | 3.12E-02 | Novel gene |
| *ASB16* | 1.23 | 3.13E-02 | Novel gene |
| *GRM2* | 1.23 | 3.13E-02 | Novel gene |
| *ENSG00000197813* | 1.21 | 3.18E-02 | Novel gene |
| *SCN5A* | 1.21 | 3.19E-02 | Novel gene |
| *FAM153B* | 1.21 | 3.19E-02 | Novel gene |
| *CASS4* | 1.21 | 3.20E-02 | Novel gene |
| *SLC6A16* | 1.19 | 3.27E-02 | Novel gene |
| *COQ2* | 1.19 | 3.29E-02 | Novel gene |
| *GLRX5* | 1.18 | 3.30E-02 | Reported gene on tuberculosis |
| *ENSG00000223745* | 1.18 | 3.33E-02 | Novel gene |
| *SCAMP1* | 1.17 | 3.35E-02 | Novel gene |
| *CBR3* | 1.17 | 3.36E-02 | Novel gene |
| *ALDH8A1* | 1.17 | 3.38E-02 | Novel gene |
| *NOL9* | 1.16 | 3.40E-02 | Novel gene |
| *CCDC163P* | 1.15 | 3.44E-02 | Novel gene |
| *KLHDC7B* | 1.15 | 3.44E-02 | Novel gene |
| *OAS1* | 1.15 | 3.45E-02 | Novel gene |
| *ENSG00000259514* | 1.15 | 3.45E-02 | Novel gene |
| *DPYD* | 1.15 | 3.46E-02 | Novel gene |
| *YTHDC2* | 1.14 | 3.48E-02 | Novel gene |
| *EXT2* | 1.14 | 3.49E-02 | Novel gene |
| *NDUFA9* | 1.14 | 3.49E-02 | Novel gene |
| *ENSG00000272666* | 1.14 | 3.50E-02 | Novel gene |
| *FEZ2* | 1.14 | 3.50E-02 | Novel gene |
| *TRMT10B* | 1.13 | 3.54E-02 | Novel gene |
| *NUDT13* | 1.12 | 3.57E-02 | Novel gene |
| *TTC18* | 1.11 | 3.62E-02 | Novel gene |
| *STK32C* | 1.11 | 3.62E-02 | Novel gene |
| *MIR3150A* | 1.11 | 3.63E-02 | Novel gene |
| *TBRG4* | 1.10 | 3.66E-02 | Novel gene |
| *AES* | 1.10 | 3.68E-02 | Novel gene |
| *STAG3L1* | 1.09 | 3.70E-02 | Novel gene |
| *CDK10* | 1.09 | 3.70E-02 | Novel gene |
| *ZZEF1* | 1.09 | 3.70E-02 | Reported gene on lung-related diseases |
| *LAMA5* | 1.09 | 3.71E-02 | Novel gene |
| *LIG3* | 1.09 | 3.73E-02 | Novel gene |
| *ENSG00000273302* | 1.08 | 3.74E-02 | Novel gene |
| *ENSG00000183748* | 1.08 | 3.75E-02 | Novel gene |
| *ASGR2* | 1.08 | 3.77E-02 | Novel gene |
| *TRIP4* | 1.08 | 3.78E-02 | Novel gene |
| *SLC35A5* | 1.08 | 3.79E-02 | Novel gene |
| *GLI4* | 1.07 | 3.84E-02 | Novel gene |
| *CORIN* | 1.06 | 3.87E-02 | Novel gene |
| *TPGS2* | 1.06 | 3.89E-02 | Novel gene |
| *ENSG00000260302* | 1.05 | 3.90E-02 | Novel gene |
| *FGL2* | 1.04 | 3.99E-02 | Novel gene |
| *MX1* | 1.04 | 4.00E-02 | Novel gene |
| *ENSG00000169203* | 1.03 | 4.01E-02 | Novel gene |
| *TRIM9* | 1.03 | 4.03E-02 | Novel gene |
| *EXO5* | 1.03 | 4.04E-02 | Novel gene |
| *NDST1* | 1.02 | 4.10E-02 | Reported gene on lung-related diseases |
| *CLK4* | 1.01 | 4.13E-02 | Novel gene |
| *ZNF354A* | 1.01 | 4.14E-02 | Novel gene |
| *ANXA5* | 1.01 | 4.16E-02 | Reported gene on respiratory-related diseases |
| *RPH3A* | 1.01 | 4.16E-02 | Novel gene |
| *WRNIP1* | 1.01 | 4.17E-02 | Novel gene |
| *TMEM161B-AS1* | 1.00 | 4.20E-02 | Novel gene |
| *FAM69B* | 1.00 | 4.21E-02 | Novel gene |
| *ZC3H7A* | 1.00 | 4.22E-02 | Novel gene |
| *SLC9A9* | 1.00 | 4.22E-02 | Reported gene on lung-related diseases |
| *FAM149B1* | 1.00 | 4.23E-02 | Novel gene |
| *HOXB2* | 0.99 | 4.24E-02 | Novel gene |
| *CCNK* | 0.99 | 4.24E-02 | Novel gene |
| *ANKRD27* | 0.99 | 4.24E-02 | Novel gene |
| *ENSG00000231494* | 0.99 | 4.24E-02 | Novel gene |
| *RFFL* | 0.99 | 4.26E-02 | Novel gene |
| *CYP21A2* | 0.99 | 4.28E-02 | Novel gene |
| *RBMS2* | 0.98 | 4.32E-02 | Novel gene |
| *MAU2* | 0.98 | 4.35E-02 | Novel gene |
| *ENSG00000259959* | 0.97 | 4.37E-02 | Novel gene |
| *RPS5* | 0.97 | 4.41E-02 | Novel gene |
| *CRYL1* | 0.97 | 4.41E-02 | Novel gene |
| *LINC00926* | 0.96 | 4.42E-02 | Novel gene |
| *RPIA* | 0.96 | 4.47E-02 | Novel gene |
| *TRIM73* | 0.95 | 4.47E-02 | Novel gene |
| *SMAD5* | 0.95 | 4.50E-02 | Novel gene |
| *F2RL1* | 0.95 | 4.52E-02 | Novel gene |
| *SLC39A10* | 0.95 | 4.52E-02 | Novel gene |
| *KIAA1191* | 0.95 | 4.52E-02 | Novel gene |
| *AXIN2* | 0.94 | 4.54E-02 | Novel gene |
| *XRCC1* | 0.94 | 4.55E-02 | Novel gene |
| *PARP16* | 0.94 | 4.56E-02 | Novel gene |
| *ENSG00000263004* | 0.94 | 4.57E-02 | Novel gene |
| *PIK3C3* | 0.94 | 4.59E-02 | Novel gene |
| *CD101* | 0.94 | 4.59E-02 | Novel gene |
| *TFPT* | 0.93 | 4.61E-02 | Novel gene |
| *FAM26F* | 0.93 | 4.61E-02 | Novel gene |
| *RPL7P18* | 0.93 | 4.66E-02 | Novel gene |
| *PI4KAP2* | 0.93 | 4.66E-02 | Novel gene |
| *CTSC* | 0.92 | 4.67E-02 | Novel gene |
| *SH3BP1* | 0.92 | 4.69E-02 | Novel gene |
| *SLC22A31* | 0.92 | 4.69E-02 | Novel gene |
| *HEMK1* | 0.92 | 4.71E-02 | Novel gene |
| *ST6GALNAC6* | 0.92 | 4.71E-02 | Novel gene |
| *DCLRE1C* | 0.91 | 4.73E-02 | Novel gene |
| *ENSG00000270820* | 0.91 | 4.73E-02 | Novel gene |
| *CSNK1G1* | 0.91 | 4.76E-02 | Novel gene |
| *LINC00310* | 0.90 | 4.81E-02 | Novel gene |
| *PDCD5* | 0.89 | 4.87E-02 | Novel gene |
| *ENSG00000272821* | 0.89 | 4.90E-02 | Novel gene |
| *CHMP1A* | 0.88 | 4.94E-02 | Novel gene |
| *ENSG00000272761* | 0.88 | 4.96E-02 | Novel gene |
| *DDX58* | 0.88 | 4.96E-02 | Novel gene |
| *IL10* | 0.87 | 4.98E-02 | Novel gene |
| *FRAT2* | 0.87 | 4.98E-02 | Novel gene |
| *CCDC146* | 0.87 | 5.00E-02 | Novel gene |
| *GALK2* | 0.87 | 5.03E-02 | Reported gene on lung-related diseases |
