# Supplementary material for: Integrative genomics analysis identifies promising SNPs and genes implicated in tuberculosis risk based on multiple omics datasets
Source: Aging (Albany NY). 2020 Oct 13;12(19):19173–220. doi: 10.18632/aging.103744 (PMC7732298; doi:10.18632/aging.103744)
Supplement: Supplementary Table 6 [file aging-12-103744-s007..docx]

**Supplementary Table 6**. **Sherlock Bayesian analysis identifies 405 genes as tuberculosis-associated risk genes (Gene set #3) from Dataset #5 in the replication stage**

| **Gene name** | **LBF** | **Simulated P value** | **GWAS Catalog documented genes** |
| --- | --- | --- | --- |
| *PKDREJ* | 5.90 | 9.66E-05 | Novel gene |
| *TTC38* | 5.49 | 1.40E-04 | Novel gene |
| *ZNF233* | 5.36 | 1.66E-04 | Novel gene |
| *GSTA2* | 5.36 | 1.66E-04 | Novel gene |
| *ENSG00000225940* | 5.25 | 1.85E-04 | Novel gene |
| *SETD9* | 5.05 | 2.36E-04 | Novel gene |
| *GSTA1* | 4.93 | 2.66E-04 | Novel gene |
| *BDP1* | 4.88 | 2.90E-04 | Novel gene |
| *BCL7B* | 4.54 | 4.46E-04 | Novel gene |
| *ENSG00000254531* | 4.20 | 7.09E-04 | Novel gene |
| *ENSG00000253704* | 4.13 | 8.00E-04 | Novel gene |
| *CACNA1G-AS1* | 3.77 | 1.24E-03 | Novel gene |
| *FAM216A* | 3.64 | 1.45E-03 | Novel gene |
| *ATP6V1B1* | 3.44 | 1.83E-03 | Novel gene |
| *LRRC37A* | 3.44 | 1.83E-03 | Novel gene |
| *RIPK3* | 3.43 | 1.87E-03 | Novel gene |
| *ENSG00000226328* | 3.42 | 1.88E-03 | Novel gene |
| *PLEKHM1* | 3.41 | 1.94E-03 | Novel gene |
| *ITGB1* | 3.37 | 2.07E-03 | Novel gene |
| *PRPF19* | 3.36 | 2.09E-03 | Novel gene |
| *MAPT* | 3.29 | 2.30E-03 | Reported gene on lung-related and respiratory-related diseases |
| *VILL* | 3.29 | 2.31E-03 | Novel gene |
| *ENSG00000228549* | 3.22 | 2.49E-03 | Novel gene |
| *ENSG00000262539* | 3.19 | 2.59E-03 | Novel gene |
| *ENSG00000262500* | 3.17 | 2.65E-03 | Novel gene |
| *SBSPON* | 3.13 | 2.77E-03 | Reported gene on lung-related diseases |
| *ENSG00000214870* | 3.13 | 2.78E-03 | Novel gene |
| *KDM2B* | 3.10 | 2.87E-03 | Novel gene |
| *GJB3* | 3.10 | 2.87E-03 | Novel gene |
| *STYX* | 2.97 | 3.34E-03 | Novel gene |
| *TMEM132A* | 2.94 | 3.46E-03 | Novel gene |
| *KANSL1-AS1* | 2.93 | 3.48E-03 | Novel gene |
| *PTCHD2* | 2.91 | 3.59E-03 | Novel gene |
| *CUBN* | 2.90 | 3.60E-03 | Novel gene |
| *ASB16-AS1* | 2.90 | 3.63E-03 | Novel gene |
| *PDCD5* | 2.83 | 3.97E-03 | Novel gene |
| *ECI1* | 2.83 | 3.99E-03 | Novel gene |
| *SPATA20* | 2.80 | 4.13E-03 | Novel gene |
| *ABCC3* | 2.79 | 4.15E-03 | Novel gene |
| *CTSZ* | 2.79 | 4.19E-03 | Novel gene |
| *PTER* | 2.77 | 4.25E-03 | Novel gene |
| *ESRRB* | 2.75 | 4.37E-03 | Reported gene on tuberculosis |
| *FAM215B* | 2.74 | 4.46E-03 | Novel gene |
| *BMS1P3* | 2.69 | 4.79E-03 | Novel gene |
| *SLC4A8* | 2.68 | 4.81E-03 | Novel gene |
| *WNT3* | 2.68 | 4.86E-03 | Reported gene on lung-related diseases |
| *UQCC2* | 2.63 | 5.12E-03 | Reported gene on respiratory-related diseases |
| *ENSG00000150076* | 2.62 | 5.17E-03 | Novel gene |
| *TM4SF1* | 2.61 | 5.25E-03 | Novel gene |
| *SH3BGRL2* | 2.60 | 5.33E-03 | Novel gene |
| *KANSL1* | 2.58 | 5.51E-03 | Reported gene on lung-related and respiratory-related diseases |
| *ARL17A* | 2.57 | 5.57E-03 | Novel gene |
| *ENSG00000267150* | 2.56 | 5.69E-03 | Novel gene |
| *KRT23* | 2.53 | 5.91E-03 | Novel gene |
| *PCDHGC3* | 2.51 | 6.03E-03 | Novel gene |
| *ENSG00000270659* | 2.48 | 6.27E-03 | Novel gene |
| *ZNF192P1* | 2.47 | 6.33E-03 | Novel gene |
| *ARHGAP40* | 2.45 | 6.49E-03 | Novel gene |
| *ENSG00000272690* | 2.43 | 6.67E-03 | Novel gene |
| *TRDMT1* | 2.40 | 7.00E-03 | Novel gene |
| *LSM14B* | 2.35 | 7.44E-03 | Novel gene |
| *FCGRT* | 2.33 | 7.56E-03 | Novel gene |
| *PROZ* | 2.31 | 7.82E-03 | Novel gene |
| *RITA1* | 2.31 | 7.82E-03 | Novel gene |
| *ENSG00000225603* | 2.30 | 7.94E-03 | Novel gene |
| *OTUB1* | 2.29 | 8.06E-03 | Novel gene |
| *ENSG00000258454* | 2.27 | 8.26E-03 | Novel gene |
| *CPNE4* | 2.27 | 8.26E-03 | Novel gene |
| *MAP1S* | 2.25 | 8.39E-03 | Novel gene |
| *GNAI1* | 2.21 | 8.74E-03 | Reported gene on respiratory-related diseases |
| *LRRC37A2* | 2.21 | 8.74E-03 | Novel gene |
| *NBPF13P* | 2.21 | 8.76E-03 | Reported gene on lung-related diseases |
| *ZNF667-AS1* | 2.16 | 9.26E-03 | Novel gene |
| *CCT6B* | 2.16 | 9.36E-03 | Novel gene |
| *RNF168* | 2.10 | 9.92E-03 | Novel gene |
| *ENSG00000272373* | 2.10 | 9.96E-03 | Novel gene |
| *ENSG00000261000* | 2.10 | 1.00E-02 | Novel gene |
| *TDRKH* | 2.09 | 1.01E-02 | Novel gene |
| *ENSG00000234028* | 2.09 | 1.02E-02 | Novel gene |
| *ENSG00000226180* | 2.09 | 1.02E-02 | Novel gene |
| *STAB2* | 2.05 | 1.06E-02 | Novel gene |
| *ACTR3C* | 2.04 | 1.08E-02 | Novel gene |
| *FHOD3* | 2.01 | 1.12E-02 | Novel gene |
| *PMPCA* | 2.01 | 1.12E-02 | Novel gene |
| *CLN8* | 2.01 | 1.13E-02 | Novel gene |
| *APOL4* | 2.01 | 1.13E-02 | Novel gene |
| *HOXA-AS2* | 2.00 | 1.14E-02 | Novel gene |
| *TCP11L1* | 1.99 | 1.15E-02 | Novel gene |
| *GCNT1P1* | 1.98 | 1.17E-02 | Novel gene |
| *HRH4* | 1.98 | 1.17E-02 | Novel gene |
| *RFFL* | 1.97 | 1.18E-02 | Novel gene |
| *TRMT10B* | 1.97 | 1.19E-02 | Novel gene |
| *PDP1* | 1.96 | 1.20E-02 | Novel gene |
| *CDC16* | 1.95 | 1.20E-02 | Novel gene |
| *FKBP7* | 1.94 | 1.21E-02 | Novel gene |
| *NELFCD* | 1.94 | 1.21E-02 | Novel gene |
| *CCL17* | 1.94 | 1.23E-02 | Novel gene |
| *GK3P* | 1.92 | 1.24E-02 | Novel gene |
| *ULK4* | 1.92 | 1.25E-02 | Reported gene on lung-related diseases |
| *ENSG00000225231* | 1.90 | 1.28E-02 | Novel gene |
| *ENSG00000131484* | 1.90 | 1.28E-02 | Novel gene |
| *SLC26A6* | 1.89 | 1.30E-02 | Novel gene |
| *PRPH2* | 1.89 | 1.30E-02 | Novel gene |
| *ENSG00000267174* | 1.89 | 1.30E-02 | Novel gene |
| *FCHO1* | 1.88 | 1.31E-02 | Novel gene |
| *KIF25* | 1.88 | 1.32E-02 | Novel gene |
| *ZNF519* | 1.87 | 1.33E-02 | Novel gene |
| *CELSR1* | 1.86 | 1.35E-02 | Novel gene |
| *SEC16A* | 1.86 | 1.36E-02 | Novel gene |
| *ARHGEF33* | 1.86 | 1.36E-02 | Novel gene |
| *ZNF582-AS1* | 1.85 | 1.37E-02 | Novel gene |
| *HAPLN1* | 1.84 | 1.39E-02 | Reported gene on lung-related diseases |
| *RCN3* | 1.83 | 1.40E-02 | Novel gene |
| *COLEC10* | 1.83 | 1.40E-02 | Reported gene on respiratory-related diseases |
| *KLK11* | 1.83 | 1.40E-02 | Novel gene |
| *TFAM* | 1.82 | 1.42E-02 | Novel gene |
| *SHC3* | 1.81 | 1.43E-02 | Novel gene |
| *TATDN3* | 1.81 | 1.43E-02 | Novel gene |
| *SS18L1* | 1.81 | 1.44E-02 | Novel gene |
| *RYR3* | 1.80 | 1.45E-02 | Novel gene |
| *ENDOD1* | 1.80 | 1.45E-02 | Novel gene |
| *RPS11* | 1.80 | 1.46E-02 | Novel gene |
| *PMS2P3* | 1.79 | 1.46E-02 | Novel gene |
| *TMEM150C* | 1.79 | 1.47E-02 | Novel gene |
| *FLVCR1-AS1* | 1.79 | 1.47E-02 | Novel gene |
| *ENSG00000244137* | 1.79 | 1.47E-02 | Novel gene |
| *ENSG00000263503* | 1.79 | 1.47E-02 | Novel gene |
| *KIAA1143* | 1.79 | 1.47E-02 | Novel gene |
| *DHX57* | 1.78 | 1.48E-02 | Novel gene |
| *FAM86DP* | 1.78 | 1.49E-02 | Novel gene |
| *MCOLN1* | 1.77 | 1.50E-02 | Novel gene |
| *ENSG00000253982* | 1.77 | 1.51E-02 | Novel gene |
| *ENSG00000235271* | 1.73 | 1.58E-02 | Novel gene |
| *CAND2* | 1.73 | 1.59E-02 | Reported gene on lung-related diseases |
| *FAM117A* | 1.72 | 1.60E-02 | Novel gene |
| *CRHR1-IT1* | 1.72 | 1.60E-02 | Novel gene |
| *MIF4GD* | 1.72 | 1.60E-02 | Novel gene |
| *LRRC37A4P* | 1.72 | 1.61E-02 | Novel gene |
| *RBPMS2* | 1.71 | 1.62E-02 | Novel gene |
| *KIAA0040* | 1.70 | 1.64E-02 | Reported gene on lung-related diseases |
| *ENSG00000260193* | 1.70 | 1.65E-02 | Novel gene |
| *C8orf12* | 1.69 | 1.66E-02 | Novel gene |
| *LIG3* | 1.69 | 1.66E-02 | Novel gene |
| *ENSG00000231050* | 1.68 | 1.68E-02 | Novel gene |
| *ENSG00000269896* | 1.68 | 1.69E-02 | Novel gene |
| *ARHGEF40* | 1.68 | 1.69E-02 | Novel gene |
| *TIGD5* | 1.67 | 1.70E-02 | Novel gene |
| *SPRYD4* | 1.67 | 1.70E-02 | Novel gene |
| *UQCRH* | 1.67 | 1.72E-02 | Novel gene |
| *ENSG00000259422* | 1.66 | 1.73E-02 | Novel gene |
| *FAR2* | 1.66 | 1.73E-02 | Novel gene |
| *C1orf43* | 1.66 | 1.73E-02 | Novel gene |
| *GABRB3* | 1.66 | 1.74E-02 | Reported gene on respiratory-related diseases |
| *DND1P1* | 1.65 | 1.75E-02 | Novel gene |
| *AP1M2* | 1.64 | 1.76E-02 | Novel gene |
| *POLR1D* | 1.64 | 1.77E-02 | Novel gene |
| *C4BPA* | 1.64 | 1.79E-02 | Novel gene |
| *LRRK2* | 1.64 | 1.79E-02 | Novel gene |
| *LINC00944* | 1.63 | 1.80E-02 | Novel gene |
| *ZNF197* | 1.63 | 1.81E-02 | Novel gene |
| *SLC25A10* | 1.62 | 1.82E-02 | Novel gene |
| *FSIP2* | 1.62 | 1.82E-02 | Novel gene |
| *ZNF248* | 1.62 | 1.82E-02 | Novel gene |
| *TMX1* | 1.62 | 1.83E-02 | Novel gene |
| *GPRC5B* | 1.62 | 1.83E-02 | Novel gene |
| *ENSG00000242611* | 1.61 | 1.84E-02 | Novel gene |
| *GJB5* | 1.61 | 1.85E-02 | Novel gene |
| *TMEM99* | 1.61 | 1.86E-02 | Novel gene |
| *KNOP1* | 1.60 | 1.87E-02 | Novel gene |
| *KIAA0195* | 1.60 | 1.87E-02 | Novel gene |
| *SMIM8* | 1.58 | 1.91E-02 | Novel gene |
| *SPATA2L* | 1.58 | 1.93E-02 | Novel gene |
| *CLPP* | 1.57 | 1.94E-02 | Novel gene |
| *LINC01165* | 1.57 | 1.95E-02 | Novel gene |
| *SCAPER* | 1.57 | 1.95E-02 | Novel gene |
| *ZNF518A* | 1.57 | 1.95E-02 | Novel gene |
| *TRABD* | 1.56 | 1.97E-02 | Novel gene |
| *NUP50* | 1.55 | 1.99E-02 | Novel gene |
| *ENSG00000272983* | 1.54 | 2.02E-02 | Novel gene |
| *ATP6AP1L* | 1.54 | 2.02E-02 | Novel gene |
| *CCDC122* | 1.54 | 2.03E-02 | Novel gene |
| *TREM2* | 1.54 | 2.03E-02 | Novel gene |
| *HIATL1* | 1.53 | 2.05E-02 | Novel gene |
| *ENSG00000266995* | 1.53 | 2.06E-02 | Novel gene |
| *IGLV4-60* | 1.52 | 2.07E-02 | Novel gene |
| *ENSG00000259847* | 1.51 | 2.12E-02 | Novel gene |
| *ZNF25* | 1.50 | 2.12E-02 | Novel gene |
| *AMZ1* | 1.50 | 2.13E-02 | Reported gene on lung-related and respiratory-related diseases |
| *RP9* | 1.50 | 2.15E-02 | Novel gene |
| *OAF* | 1.49 | 2.15E-02 | Novel gene |
| *ZNF502* | 1.48 | 2.18E-02 | Novel gene |
| *RAD21* | 1.48 | 2.18E-02 | Novel gene |
| *NADK* | 1.48 | 2.19E-02 | Reported gene on lung-related diseases |
| *OPTN* | 1.48 | 2.19E-02 | Novel gene |
| *GALM* | 1.47 | 2.21E-02 | Novel gene |
| *RPS23* | 1.47 | 2.22E-02 | Novel gene |
| *ENSG00000259877* | 1.47 | 2.23E-02 | Novel gene |
| *ANKLE1* | 1.45 | 2.27E-02 | Novel gene |
| *ZNF497* | 1.44 | 2.31E-02 | Novel gene |
| *ENSG00000259344* | 1.44 | 2.33E-02 | Novel gene |
| *HDAC10* | 1.43 | 2.36E-02 | Novel gene |
| *FEZ2* | 1.43 | 2.36E-02 | Novel gene |
| *HEATR3* | 1.42 | 2.38E-02 | Novel gene |
| *ACTBP8* | 1.42 | 2.39E-02 | Novel gene |
| *SRP14-AS1* | 1.40 | 2.44E-02 | Novel gene |
| *BRK1* | 1.40 | 2.44E-02 | Novel gene |
| *FBXL17* | 1.40 | 2.44E-02 | Reported gene on lung-related diseases |
| *CABLES2* | 1.40 | 2.45E-02 | Novel gene |
| *ENSG00000247317* | 1.39 | 2.47E-02 | Novel gene |
| *SESTD1* | 1.39 | 2.47E-02 | Novel gene |
| *ENSG00000258702* | 1.39 | 2.49E-02 | Novel gene |
| *FAM114A1* | 1.39 | 2.49E-02 | Reported gene on respiratory-related diseases |
| *ENSG00000259775* | 1.38 | 2.50E-02 | Novel gene |
| *MEST* | 1.38 | 2.50E-02 | Novel gene |
| *ENSG00000260302* | 1.38 | 2.52E-02 | Novel gene |
| *ENSG00000224713* | 1.37 | 2.53E-02 | Novel gene |
| *PDK1* | 1.37 | 2.54E-02 | Novel gene |
| *C16orf46* | 1.37 | 2.54E-02 | Novel gene |
| *RBM11* | 1.37 | 2.55E-02 | Novel gene |
| *CRELD1* | 1.35 | 2.61E-02 | Reported gene on lung-related diseases |
| *FAM69B* | 1.34 | 2.64E-02 | Novel gene |
| *ADAD2* | 1.34 | 2.64E-02 | Novel gene |
| *ENSG00000267733* | 1.34 | 2.65E-02 | Novel gene |
| *FAM153B* | 1.34 | 2.65E-02 | Novel gene |
| *RRM1* | 1.33 | 2.67E-02 | Novel gene |
| *ENSG00000223522* | 1.33 | 2.68E-02 | Novel gene |
| *EIF4B* | 1.32 | 2.72E-02 | Novel gene |
| *PARP8* | 1.32 | 2.73E-02 | Novel gene |
| *ENSG00000254333* | 1.32 | 2.73E-02 | Novel gene |
| *NFXL1* | 1.31 | 2.74E-02 | Novel gene |
| *SWAP70* | 1.31 | 2.74E-02 | Novel gene |
| *PM20D2* | 1.31 | 2.75E-02 | Novel gene |
| *CYP2G1P* | 1.31 | 2.75E-02 | Novel gene |
| *LYRM2* | 1.31 | 2.76E-02 | Novel gene |
| *EPB41L3* | 1.31 | 2.76E-02 | Novel gene |
| *CROCCP2* | 1.30 | 2.78E-02 | Novel gene |
| *ANKRD62* | 1.30 | 2.81E-02 | Novel gene |
| *RCN2* | 1.29 | 2.81E-02 | Novel gene |
| *RPL7P18* | 1.29 | 2.83E-02 | Novel gene |
| *ARHGEF39* | 1.29 | 2.84E-02 | Novel gene |
| *PCBP3* | 1.28 | 2.85E-02 | Novel gene |
| *HBS1L* | 1.28 | 2.86E-02 | Novel gene |
| *ZNF329* | 1.28 | 2.86E-02 | Novel gene |
| *EBF3* | 1.28 | 2.88E-02 | Novel gene |
| *ZNF138* | 1.28 | 2.88E-02 | Novel gene |
| *DR1* | 1.27 | 2.89E-02 | Novel gene |
| *MNX1* | 1.27 | 2.90E-02 | Novel gene |
| *ENSG00000183657* | 1.27 | 2.90E-02 | Novel gene |
| *LYPD2* | 1.27 | 2.90E-02 | Novel gene |
| *CEP152* | 1.27 | 2.91E-02 | Novel gene |
| *CDRT15P1* | 1.26 | 2.92E-02 | Reported gene on respiratory-related diseases |
| *SURF1* | 1.26 | 2.93E-02 | Novel gene |
| *NDUFA11* | 1.26 | 2.94E-02 | Novel gene |
| *ETHE1* | 1.26 | 2.94E-02 | Novel gene |
| *ENSG00000271912* | 1.25 | 2.96E-02 | Novel gene |
| *FNDC3B* | 1.25 | 2.97E-02 | Reported gene on lung-related diseases |
| *SNCG* | 1.24 | 2.99E-02 | Novel gene |
| *ENSG00000272335* | 1.24 | 3.02E-02 | Novel gene |
| *ENSG00000223745* | 1.23 | 3.04E-02 | Novel gene |
| *ZNF837* | 1.23 | 3.05E-02 | Novel gene |
| *ENSG00000262879* | 1.22 | 3.07E-02 | Novel gene |
| *PARD6B* | 1.22 | 3.08E-02 | Novel gene |
| *FAM85A* | 1.22 | 3.10E-02 | Novel gene |
| *GRM2* | 1.22 | 3.10E-02 | Novel gene |
| *STIM1* | 1.21 | 3.13E-02 | Reported gene on lung-related diseases |
| *BTBD9* | 1.20 | 3.16E-02 | Novel gene |
| *UBL3* | 1.20 | 3.18E-02 | Novel gene |
| *AGTRAP* | 1.20 | 3.18E-02 | Novel gene |
| *MAPK12* | 1.18 | 3.25E-02 | Novel gene |
| *CFH* | 1.18 | 3.25E-02 | Reported gene on lung-related diseases |
| *ZNF175* | 1.18 | 3.25E-02 | Novel gene |
| *GPR63* | 1.18 | 3.25E-02 | Novel gene |
| *PIF1* | 1.18 | 3.25E-02 | Novel gene |
| *MORN1* | 1.17 | 3.29E-02 | Novel gene |
| *RDH13* | 1.17 | 3.30E-02 | Novel gene |
| *FBXO41* | 1.16 | 3.32E-02 | Novel gene |
| *ENSG00000263004* | 1.16 | 3.32E-02 | Novel gene |
| *AK5* | 1.16 | 3.34E-02 | Reported gene on lung-related diseases |
| *CDK10* | 1.16 | 3.35E-02 | Novel gene |
| *POLR3G* | 1.15 | 3.36E-02 | Novel gene |
| *LINC00648* | 1.15 | 3.37E-02 | Novel gene |
| *ENSG00000248596* | 1.15 | 3.37E-02 | Novel gene |
| *HOXB2* | 1.14 | 3.40E-02 | Novel gene |
| *ADAMTS7P3* | 1.14 | 3.42E-02 | Novel gene |
| *ENSG00000272420* | 1.14 | 3.44E-02 | Novel gene |
| *GTF2F1* | 1.13 | 3.46E-02 | Novel gene |
| *AP3B1* | 1.13 | 3.47E-02 | Reported gene on lung-related and respiratory-related diseases |
| *FRAS1* | 1.13 | 3.47E-02 | Reported gene on lung-related diseases |
| *LINC01146* | 1.12 | 3.53E-02 | Novel gene |
| *ZNF320* | 1.11 | 3.55E-02 | Novel gene |
| *KRT85* | 1.10 | 3.59E-02 | Novel gene |
| *MRFAP1* | 1.10 | 3.60E-02 | Novel gene |
| *ADCK2* | 1.09 | 3.66E-02 | Novel gene |
| *GLI4* | 1.09 | 3.66E-02 | Novel gene |
| *PLCD1* | 1.09 | 3.67E-02 | Novel gene |
| *TRIP4* | 1.08 | 3.67E-02 | Novel gene |
| *NPHP4* | 1.08 | 3.69E-02 | Novel gene |
| *YTHDC2* | 1.08 | 3.69E-02 | Novel gene |
| *NMNAT3* | 1.08 | 3.73E-02 | Novel gene |
| *FAM181A* | 1.08 | 3.73E-02 | Novel gene |
| *TM4SF18* | 1.07 | 3.73E-02 | Novel gene |
| *ENSG00000260259* | 1.07 | 3.74E-02 | Novel gene |
| *ZNF354A* | 1.07 | 3.75E-02 | Novel gene |
| *POM121C* | 1.07 | 3.75E-02 | Novel gene |
| *TTC18* | 1.07 | 3.75E-02 | Novel gene |
| *MED22* | 1.07 | 3.76E-02 | Novel gene |
| *LAMA5* | 1.07 | 3.76E-02 | Novel gene |
| *ARL10* | 1.07 | 3.77E-02 | Novel gene |
| *SENP5* | 1.07 | 3.78E-02 | Novel gene |
| *NUDT13* | 1.07 | 3.78E-02 | Novel gene |
| *GLRX5* | 1.06 | 3.78E-02 | Reported gene on tuberculosis |
| *ENSG00000235994* | 1.06 | 3.80E-02 | Novel gene |
| *LRRK1* | 1.06 | 3.82E-02 | Novel gene |
| *ACCS* | 1.06 | 3.82E-02 | Novel gene |
| *CCDC163P* | 1.05 | 3.84E-02 | Novel gene |
| *RPH3A* | 1.05 | 3.85E-02 | Novel gene |
| *ENGASE* | 1.05 | 3.86E-02 | Novel gene |
| *LIMD1* | 1.05 | 3.86E-02 | Novel gene |
| *CARD9* | 1.05 | 3.86E-02 | Reported gene on lung-related diseases |
| *KIF25-AS1* | 1.05 | 3.87E-02 | Novel gene |
| *HSD17B2* | 1.05 | 3.87E-02 | Reported gene on lung-related diseases |
| *LIPC* | 1.05 | 3.87E-02 | Novel gene |
| *ENSG00000224014* | 1.04 | 3.89E-02 | Novel gene |
| *LYNX1* | 1.04 | 3.92E-02 | Novel gene |
| *ZNF266* | 1.03 | 3.94E-02 | Novel gene |
| *ENSG00000258738* | 1.03 | 3.95E-02 | Novel gene |
| *VWDE* | 1.03 | 3.96E-02 | Novel gene |
| *AGR3* | 1.03 | 3.96E-02 | Reported gene on lung-related diseases |
| *SKINTL* | 1.03 | 3.96E-02 | Novel gene |
| *FAM149B1* | 1.03 | 3.99E-02 | Novel gene |
| *SRSF8* | 1.02 | 4.00E-02 | Novel gene |
| *HAX1* | 1.02 | 4.02E-02 | Novel gene |
| *GLIS1* | 1.02 | 4.02E-02 | Novel gene |
| *ENSG00000266289* | 1.02 | 4.04E-02 | Novel gene |
| *ZNF112* | 1.02 | 4.05E-02 | Novel gene |
| *PHLDB3* | 1.01 | 4.06E-02 | Novel gene |
| *DCAF16* | 1.01 | 4.06E-02 | Novel gene |
| *ENSG00000250328* | 1.01 | 4.08E-02 | Novel gene |
| *FAM83D* | 1.01 | 4.08E-02 | Novel gene |
| *GATA2* | 1.01 | 4.09E-02 | Reported gene on respiratory-related diseases |
| *KIF1B* | 1.01 | 4.09E-02 | Reported gene on lung-related diseases |
| *ENSG00000273253* | 1.00 | 4.12E-02 | Novel gene |
| *MRPL41* | 1.00 | 4.12E-02 | Novel gene |
| *MRPL9* | 1.00 | 4.13E-02 | Novel gene |
| *IGLV8-61* | 1.00 | 4.13E-02 | Novel gene |
| *USP24* | 1.00 | 4.15E-02 | Reported gene on respiratory-related diseases |
| *ENSG00000250075* | 0.99 | 4.17E-02 | Novel gene |
| *SUCO* | 0.99 | 4.17E-02 | Novel gene |
| *ENSG00000261054* | 0.99 | 4.17E-02 | Novel gene |
| *LRPAP1* | 0.99 | 4.19E-02 | Reported gene on tuberculosis |
| *ENSG00000273148* | 0.99 | 4.20E-02 | Novel gene |
| *UBE2F* | 0.98 | 4.23E-02 | Novel gene |
| *SRCRB4D* | 0.97 | 4.30E-02 | Novel gene |
| *CCDC107* | 0.97 | 4.31E-02 | Novel gene |
| *USP37* | 0.97 | 4.32E-02 | Novel gene |
| *SRD5A1* | 0.97 | 4.32E-02 | Novel gene |
| *FAM173B* | 0.97 | 4.34E-02 | Novel gene |
| *AIF1L* | 0.96 | 4.36E-02 | Novel gene |
| *SLC39A12* | 0.96 | 4.36E-02 | Novel gene |
| *TMEM232* | 0.96 | 4.38E-02 | Reported gene on respiratory-related diseases |
| *CDRT15* | 0.96 | 4.38E-02 | Novel gene |
| *EIF3L* | 0.96 | 4.39E-02 | Novel gene |
| *ENSG00000273456* | 0.96 | 4.39E-02 | Novel gene |
| *TPMT* | 0.96 | 4.39E-02 | Novel gene |
| *TRIM73* | 0.96 | 4.40E-02 | Novel gene |
| *ZNF761* | 0.96 | 4.40E-02 | Novel gene |
| *BRI3* | 0.95 | 4.44E-02 | Novel gene |
| *TMEM18* | 0.95 | 4.45E-02 | Reported gene on lung-related diseases |
| *GALNT3* | 0.95 | 4.45E-02 | Novel gene |
| *EMC3-AS1* | 0.95 | 4.45E-02 | Novel gene |
| *CD37* | 0.95 | 4.45E-02 | Novel gene |
| *DRG2* | 0.95 | 4.46E-02 | Novel gene |
| *MCC* | 0.94 | 4.47E-02 | Reported gene on lung-related diseases |
| *MTG2* | 0.94 | 4.47E-02 | Novel gene |
| *SMAD5* | 0.94 | 4.47E-02 | Novel gene |
| *ENSG00000255050* | 0.94 | 4.48E-02 | Novel gene |
| *MCUR1* | 0.94 | 4.49E-02 | Novel gene |
| *TBRG4* | 0.93 | 4.53E-02 | Novel gene |
| *EEF1D* | 0.93 | 4.54E-02 | Novel gene |
| *ENSG00000226261* | 0.93 | 4.56E-02 | Novel gene |
| *ENSG00000236849* | 0.93 | 4.57E-02 | Novel gene |
| *ENSG00000205047* | 0.93 | 4.57E-02 | Novel gene |
| *PI4KA* | 0.93 | 4.57E-02 | Novel gene |
| *TFPT* | 0.92 | 4.62E-02 | Novel gene |
| *ETFDH* | 0.92 | 4.63E-02 | Novel gene |
| *RPS5* | 0.91 | 4.65E-02 | Novel gene |
| *ENSG00000183748* | 0.91 | 4.69E-02 | Novel gene |
| *COA6* | 0.91 | 4.70E-02 | Novel gene |
| *C1GALT1* | 0.90 | 4.74E-02 | Reported gene on lung-related and respiratory-related diseases |
| *ENSG00000273151* | 0.90 | 4.75E-02 | Novel gene |
| *CBR3* | 0.90 | 4.77E-02 | Novel gene |
| *ZNF33B* | 0.90 | 4.77E-02 | Novel gene |
| *F10* | 0.89 | 4.80E-02 | Novel gene |
| *LYPD3* | 0.89 | 4.81E-02 | Novel gene |
| *NIPAL2* | 0.88 | 4.85E-02 | Novel gene |
| *ICA1* | 0.88 | 4.87E-02 | Novel gene |
| *C17orf89* | 0.88 | 4.88E-02 | Novel gene |
| *GNB1L* | 0.88 | 4.89E-02 | Reported gene on lung-related diseases |
| *ANKRD9* | 0.88 | 4.90E-02 | Novel gene |
| *C18orf63* | 0.88 | 4.90E-02 | Novel gene |
| *FCHO2* | 0.87 | 4.95E-02 | Novel gene |
| *LEKR1* | 0.87 | 4.96E-02 | Reported gene on respiratory-related diseases |
| *ENSG00000254427* | 0.87 | 4.97E-02 | Novel gene |
| *SUN1* | 0.86 | 4.98E-02 | Novel gene |
| *BRIP1* | 0.86 | 4.99E-02 | Reported gene on lung-related diseases |

**Note:** Reported genes mean these genes have been documented to be associated with tuberculosis, lung-related and respiratory-related disease in the GWAS Catalog database; Novel genes mean these genes were not documented in the GWAS Catalog database.
