# Supplementary material for: Integrative genomics analysis identifies promising SNPs and genes implicated in tuberculosis risk based on multiple omics datasets
Source: Aging (Albany NY). 2020 Oct 13;12(19):19173–220. doi: 10.18632/aging.103744 (PMC7732298; doi:10.18632/aging.103744)
Supplement: Supplementary Tables 7, 8, 9, 10, 11, 12 and 13 [file aging-12-103744-s008..pdf]

SUPPLEMENTARY TABLES

Supplementary Table 7. Significant pathways enriched by tuberculosis-associated genes (Gene set #2) identified from Sherlock Bayesian analysis of dataset #4 in the replication stage.

Pathway ID	Database	Input number	Background number	P-Value	FDR
R-HSA-1430728	Reactome	28	2075	4.09E-07	1.19E-04
R-HSA-168256	Reactome	28	2096	4.98E-07	1.33E-04
R-HSA-1643685	Reactome	19	1049	5.30E-07	1.33E-04
R-HSA-392499	Reactome	26	2012	2.37E-06	3.72E-04
hsa01100	KEGG PATHWAY	21	1433	3.61E-06	5.05E-04
R-HSA-162582	Reactome	30	2689	6.93E-06	8.72E-04
R-HSA-597592	Reactome	20	1412	1.01E-05	1.09E-03
R-HSA-2470946	Reactome	3	10	2.53E-05	2.39E-03
R-HSA-68884	Reactome	3	14	5.94E-05	4.87E-03
R-HSA-71387	Reactome	8	288	6.82E-05	5.47E-03
R-HSA-168249	Reactome	15	1043	1.12E-04	6.97E-03
R-HSA-211945	Reactome	5	105	1.51E-04	8.92E-03
R-HSA-199991	Reactome	11	631	1.89E-04	1.10E-02
R-HSA-8953854	Reactome	11	667	3.00E-04	1.66E-02
R-HSA-5653656	Reactome	11	669	3.07E-04	1.68E-02
R-HSA-77075	Reactome	3	27	3.39E-04	1.80E-02
R-HSA-167160	Reactome	3	27	3.39E-04	1.80E-02
R-HSA-72086	Reactome	3	29	4.12E-04	1.90E-02
R-HSA-74160	Reactome	17	1448	4.17E-04	1.90E-02
R-HSA-73857	Reactome	16	1316	4.24E-04	1.90E-02
R-HSA-167172	Reactome	4	73	4.27E-04	1.90E-02
R-HSA-6807505	Reactome	4	74	4.49E-04	1.96E-02
hsa00030	KEGG PATHWAY	3	30	4.51E-04	1.96E-02
R-HSA-397014	Reactome	6	209	4.70E-04	1.97E-02
R-HSA-382551	Reactome	11	720	5.61E-04	2.19E-02
R-HSA-446203	Reactome	7	304	5.81E-04	2.22E-02
R-HSA-5649702	Reactome	2	7	7.19E-04	2.42E-02
R-HSA-167287	Reactome	3	36	7.41E-04	2.45E-02
R-HSA-167290	Reactome	3	36	7.41E-04	2.45E-02
R-HSA-8983711	Reactome	2	9	1.09E-03	3.27E-02
R-HSA-15869	Reactome	4	95	1.10E-03	3.27E-02
R-HSA-5362517	Reactome	3	43	1.20E-03	3.55E-02
R-HSA-5685939	Reactome	2	10	1.31E-03	3.73E-02
R-HSA-212436	Reactome	14	1193	1.33E-03	3.79E-02
R-HSA-5668914	Reactome	4	103	1.47E-03	4.06E-02
R-HSA-2468052	Reactome	2	11	1.54E-03	4.24E-02
R-HSA-167152	Reactome	3	48	1.62E-03	4.37E-02
R-HSA-6785807	Reactome	4	108	1.73E-03	4.64E-02
R-HSA-6798695	Reactome	8	478	1.80E-03	4.72E-02
R-HSA-5696398	Reactome	4	111	1.91E-03	4.90E-02

Note: Proportion of risk genes: these identified risk genes (Input number) accounted for the proportion of all genes in each pathway (Background number) enriched by these genes. FDR values were calculated by using the method of Benjamini-Hochberg false discovery rate (FDR) correction.

Supplementary Table 8. Significant GO-terms enriched by tuberculosis-associated genes (Gene set #2) identified from Sherlock Bayesian analysis of dataset #4 in the replication stage.

GO-terms ID	Input number	Background number	P-Value	FDR
GO:0043229	33	1863	4.63E-11	8.74E-08
GO:0005488	31	2247	5.75E-08	4.34E-05
GO:0097159	18	845	1.07E-07	5.78E-05
GO:0032555	11	292	1.69E-07	7.08E-05
GO:0009987	34	2852	3.69E-07	1.16E-04
GO:0005737	24	1641	7.34E-07	1.63E-04
GO:0043227	27	2030	8.76E-07	1.74E-04
GO:0005622	28	2228	1.61E-06	2.89E-04
GO:0043168	12	454	1.76E-06	2.89E-04
GO:0019222	18	1070	2.92E-06	4.24E-04
GO:0097708	9	261	4.54E-06	5.91E-04
GO:0005829	12	531	8.30E-06	9.79E-04
GO:0003824	18	1162	8.82E-06	9.79E-04
GO:0032553	9	292	1.08E-05	1.11E-03
GO:0031982	13	667	1.62E-05	1.57E-03
GO:0043226	24	2086	3.77E-05	3.39E-03
GO:0005515	21	1688	3.96E-05	3.39E-03
GO:0010468	15	1012	8.07E-05	6.08E-03
GO:0005654	10	474	8.21E-05	6.08E-03
GO:1901265	9	388	9.28E-05	6.61E-03
GO:0003723	6	155	9.91E-05	6.80E-03
GO:0008152	26	2527	1.09E-04	6.97E-03
GO:0000794	3	18	1.15E-04	6.97E-03
GO:1901363	13	813	1.16E-04	6.97E-03
GO:0030054	6	177	1.99E-04	1.12E-02
GO:0036094	9	467	3.55E-04	1.83E-02
GO:0051173	9	469	3.66E-04	1.83E-02
GO:1903708	3	28	3.74E-04	1.83E-02
GO:0005634	15	1182	4.14E-04	1.90E-02
GO:0006266	2	5	4.22E-04	1.90E-02
GO:0044237	21	2027	4.64E-04	1.97E-02
GO:0046914	6	215	5.43E-04	2.19E-02
GO:1990904	4	79	5.68E-04	2.19E-02
GO:0060089	6	217	5.69E-04	2.19E-02
GO:0043902	4	80	5.94E-04	2.22E-02
GO:0036211	12	857	6.76E-04	2.42E-02
GO:0048037	4	84	7.08E-04	2.42E-02
GO:0016818	6	227	7.16E-04	2.42E-02
GO:0110165	26	2864	7.18E-04	2.42E-02
GO:0042629	2	7	7.19E-04	2.42E-02
GO:0001649	3	38	8.59E-04	2.79E-02
GO:0070942	2	8	8.95E-04	2.84E-02
GO:0031331	4	92	9.80E-04	3.06E-02
GO:0016486	2	9	1.09E-03	3.27E-02
GO:0009262	2	9	1.09E-03	3.27E-02
GO:2000108	2	10	1.31E-03	3.73E-02
GO:0010976	3	48	1.62E-03	4.37E-02

GO:0019320	2	12	1.79E-03	4.72E-02
GO:0016787	9	593	1.83E-03	4.74E-02
GO:0000166	7	377	1.95E-03	4.95E-02

Note: Proportion of risk genes: these identified risk genes (Input number) accounted for the proportion of all genes in each pathway (Background number) enriched by these genes. FDR values were calculated by using the method of Benjamini-Hochberg false discovery rate (FDR) correction.

Supplementary Table 9. Significant KEGG and NHGRI GWAS catalog disease enriched by tuberculosis-associated genes (Gene set #2) identified from Sherlock Bayesian analysis of dataset #4 in the replication stage.

Disease terms	Database	Input number	Background number	P-Value	FDR
QT interval	NHGRI GWAS Catalog	6	37	4.28E-08	4.34E-05
Obesity-related traits	NHGRI GWAS Catalog	16	691	1.89E-07	7.11E-05
Ulcerative colitis	NHGRI GWAS Catalog	6	138	5.35E-05	4.48E-03
Congenital disorders of metabolism	KEGG DISEASE	12	695	1.06E-04	6.97E-03
Hematological and biochemical traits	NHGRI GWAS Catalog	3	31	4.93E-04	2.05E-02
Skin and soft tissue diseases	KEGG DISEASE	4	103	1.47E-03	4.06E-02
Skin diseases	KEGG DISEASE	4	103	1.47E-03	4.06E-02

Note: Proportion of risk genes: these identified risk genes (Input number) accounted for the proportion of all genes in each pathway (Background number) enriched by these genes. FDR values were calculated by using the method of Benjamini-Hochberg false discovery rate (FDR) correction.

Supplementary Table 10. Significant pathways enriched by tuberculosis-associated genes (Gene set #3) identified from Sherlock Bayesian analysis of dataset #5 in the replication stage.

Pathway ID	Database	Input number	Background number	P-Value	FDR
R-HSA-1430728	Reactome	35	2075	2.43E-08	3.16E-05
R-HSA-392499	Reactome	33	2012	1.18E-07	5.19E-05
R-HSA-74160	Reactome	26	1448	5.42E-07	1.24E-04
R-HSA-212436	Reactome	22	1193	2.73E-06	4.24E-04
R-HSA-597592	Reactome	23	1412	1.17E-05	1.34E-03
R-HSA-73857	Reactome	22	1316	1.23E-05	1.37E-03
R-HSA-72649	Reactome	5	58	3.22E-05	3.21E-03
R-HSA-72702	Reactome	5	58	3.22E-05	3.21E-03
R-HSA-72662	Reactome	5	59	3.48E-05	3.32E-03
hsa05168	KEGG PATHWAY	12	492	4.15E-05	3.76E-03
R-HSA-72695	Reactome	4	51	2.88E-04	1.67E-02
R-HSA-72766	Reactome	8	291	3.67E-04	2.07E-02
R-HSA-9006934	Reactome	10	458	4.22E-04	2.28E-02
R-HSA-156827	Reactome	5	111	5.74E-04	2.79E-02
R-HSA-72706	Reactome	5	112	5.97E-04	2.87E-02
R-HSA-5653656	Reactome	12	669	6.51E-04	3.09E-02
R-HSA-499943	Reactome	3	28	7.46E-04	3.34E-02
R-HSA-72737	Reactome	5	119	7.76E-04	3.39E-02
R-HSA-72613	Reactome	5	119	7.76E-04	3.39E-02
R-HSA-1614517	Reactome	2	6	8.98E-04	3.55E-02
R-HSA-196807	Reactome	3	31	9.81E-04	3.76E-02
R-HSA-168273	Reactome	5	131	1.17E-03	3.98E-02
hsa05133	KEGG PATHWAY	4	76	1.19E-03	3.98E-02
R-HSA-382551	Reactome	12	720	1.21E-03	3.98E-02
R-HSA-199991	Reactome	11	631	1.35E-03	4.22E-02
hsa00983	KEGG PATHWAY	4	79	1.37E-03	4.22E-02
R-HSA-1643685	Reactome	15	1049	1.41E-03	4.22E-02
R-HSA-168255	Reactome	5	141	1.61E-03	4.56E-02
R-HSA-159763	Reactome	2	9	1.74E-03	4.78E-02

Note: Proportion of risk genes: these identified risk genes (Input number) accounted for the proportion of all genes in each pathway (Background number) enriched by these genes. FDR values were calculated by using the method of Benjamini-Hochberg false discovery rate (FDR) correction.

Supplementary Table 11. Significant GO-terms enriched by tuberculosis-associated genes (Gene set #3) identified from Sherlock Bayesian analysis of dataset #5 in the replication stage.

GO-terms ID	Input number	Background number	P-Value	FDR
GO:0005515	32	1688	7.25E-09	1.41E-05
GO:0110165	42	2864	4.32E-08	3.36E-05
GO:0019222	23	1070	1.23E-07	5.19E-05
GO:0043227	33	2030	1.44E-07	5.19E-05
GO:0005488	35	2247	1.60E-07	5.19E-05
GO:0005622	34	2228	3.86E-07	1.07E-04
GO:0000166	13	377	5.26E-07	1.24E-04
GO:0043229	30	1863	6.65E-07	1.36E-04
GO:0043231	27	1606	1.12E-06	2.07E-04
GO:0043167	20	962	1.36E-06	2.31E-04
GO:0043233	16	725	7.63E-06	9.91E-04
GO:0030659	6	75	7.69E-06	9.91E-04
GO:1901363	17	813	7.90E-06	9.91E-04
GO:0046872	15	657	9.96E-06	1.17E-03
GO:0005509	7	137	2.15E-05	2.26E-03
GO:0005576	15	736	3.58E-05	3.32E-03
GO:0003723	7	155	4.56E-05	3.95E-03
GO:0005737	23	1641	1.11E-04	8.80E-03
GO:0010468	17	1012	1.13E-04	8.80E-03
GO:0032991	13	639	1.20E-04	8.80E-03
GO:1990904	5	79	1.28E-04	9.07E-03
GO:0005856	9	320	1.35E-04	9.23E-03
GO:0005739	8	260	1.76E-04	1.14E-02
GO:0031982	13	667	1.81E-04	1.14E-02
GO:0031090	11	493	1.84E-04	1.14E-02
GO:0008152	30	2527	1.91E-04	1.14E-02
GO:0032553	8	292	3.75E-04	2.07E-02
GO:0031967	6	165	4.89E-04	2.54E-02
GO:1901265	9	388	5.34E-04	2.70E-02
GO:0005654	10	474	5.47E-04	2.70E-02
GO:0009295	2	5	6.76E-04	3.10E-02
GO:0005635	4	65	6.85E-04	3.10E-02
GO:0031975	6	183	8.26E-04	3.47E-02
GO:0016787	11	593	8.30E-04	3.47E-02
GO:0044237	24	2027	8.66E-04	3.51E-02
GO:0016020	19	1443	9.02E-04	3.55E-02
GO:0043228	11	606	9.85E-04	3.76E-02
GO:0071704	28	2548	1.01E-03	3.78E-02
GO:0032549	4	74	1.09E-03	3.95E-02
GO:0016229	2	7	1.15E-03	3.96E-02
GO:0070129	2	7	1.15E-03	3.96E-02
GO:0005310	2	7	1.15E-03	3.96E-02
GO:0097367	8	351	1.20E-03	3.98E-02
GO:0033036	9	440	1.26E-03	4.10E-02
GO:0005886	12	726	1.29E-03	4.12E-02
GO:0065003	8	358	1.36E-03	4.22E-02
GO:0034707	2	8	1.43E-03	4.22E-02

GO:0003777	2	8	1.43E-03	4.22E-02
GO:0043025	4	82	1.56E-03	4.49E-02
GO:0005215	6	210	1.63E-03	4.58E-02
GO:0035091	3	38	1.70E-03	4.68E-02

Note: Proportion of risk genes: these identified risk genes (Input number) accounted for the proportion of all genes in each pathway (Background number) enriched by these genes. FDR values were calculated by using the method of Benjamini-Hochberg false discovery rate (FDR) correction.

Supplementary Table 12. Significant KEGG and NHGRI GWAS catalog disease enriched by tuberculosis-associated genes (Gene set #3) identified from Sherlock Bayesian analysis of dataset #5 in the replication stage.

Disease terms	Database	Input number	Background number	P-Value	FDR
Parkinson's disease	NHGRI GWAS Catalog	6	56	1.60E-06	2.59E-04
Hematological and biochemical traits	NHGRI GWAS Catalog	4	31	4.83E-05	4.09E-03
Hematologic diseases	KEGG DISEASE	7	181	1.16E-04	8.80E-03
Congenital disorders of metabolism	KEGG DISEASE	13	695	2.66E-04	1.57E-02
Mean platelet volume	NHGRI GWAS Catalog	4	55	3.77E-04	2.07E-02
Metabolite levels	NHGRI GWAS Catalog	5	107	4.89E-04	2.54E-02
Obesity-related traits	NHGRI GWAS Catalog	12	691	8.56E-04	3.51E-02
Cardiovascular diseases	KEGG DISEASE	8	342	1.02E-03	3.79E-02
Serum total protein level	NHGRI GWAS Catalog	2	8	1.43E-03	4.22E-02
Triglycerides	NHGRI GWAS Catalog	4	81	1.49E-03	4.37E-02
QT interval	NHGRI GWAS Catalog	3	37	1.58E-03	4.52E-02
Bone mineral density	NHGRI GWAS Catalog	4	85	1.77E-03	4.81E-02
Nervous system diseases	KEGG DISEASE	13	859	1.78E-03	4.81E-02

Note: Proportion of risk genes: these identified risk genes (Input number) accounted for the proportion of all genes in each pathway (Background number) enriched by these genes. FDR values were calculated by using the method of Benjamini-Hochberg false discovery rate (FDR) correction.

Supplementary Table 13. 2 common diseases enriched by tuberculosis-associated genes across 3 gene sets identified from Sherlock analysis.

Disease terms	Database	Gene set #1		Gene set #2		Gene set #3	
		Proportion of risk genes	FDR	Proportion of risk genes	Corrected P-Value	Proportion of risk genes	Corrected P-Value
Congenital disorders of metabolism	KEGG DISEASE	5.61%	4.96E-12	1.73%	6.97E-03	1.87%	1.57E-02
Obesity-related traits	NHGRI GWAS Catalog	5.07%	1.23E-09	2.32%	7.11E-05	1.74%	3.51E-02

Note: Proportion of risk genes: these identified risk genes (Input number) accounted for the proportion of all genes in each pathway (Background number) enriched by these genes. FDR values were calculated by using the method of Benjamini-Hochberg false discovery rate (FDR) correction.
