# Supplementary material for: Integrative genomics analysis identifies promising SNPs and genes implicated in tuberculosis risk based on multiple omics datasets
Source: Aging (Albany NY). 2020 Oct 13;12(19):19173–220. doi: 10.18632/aging.103744 (PMC7732298; doi:10.18632/aging.103744)
Supplement: Supplementary Table 14 [file aging-12-103744-s009..docx]

**Supplementary Table 14**. **MAGMA gene-based analysis identifies 1,017 genes as tuberculosis-associated risk genes (Gene set #4) from Dataset #1 in the replication stage**

| **Gene** | **CHR** | **START** | **STOP** | **NSNPS** | **ZSTAT** | **MAGMA-based P value** | **GWAS Catalog documented genes** |
| --- | --- | --- | --- | --- | --- | --- | --- |
| *TRMU* | 22 | 46711298 | 46773237 | 258 | 4.99 | 3.04E-07 | Novel gene |
| *GTSE1* | 22 | 46672638 | 46749596 | 302 | 4.74 | 1.08E-06 | Novel gene |
| *RHBDL2* | 1 | 39330614 | 39427456 | 367 | 4.20 | 1.32E-05 | Novel gene |
| *TTC38* | 22 | 46643861 | 46709905 | 276 | 4.12 | 1.92E-05 | Novel gene |
| *BDP1* | 5 | 70731442 | 70883649 | 472 | 3.87 | 5.42E-05 | Novel gene |
| *GSTA1* | 6 | 52636178 | 52688664 | 159 | 3.85 | 5.94E-05 | Novel gene |
| *CDPF1* | 22 | 46619910 | 46666193 | 204 | 3.84 | 6.11E-05 | Novel gene |
| *MAP3K1* | 5 | 56090900 | 56211979 | 488 | 3.84 | 6.26E-05 | Reported gene on lung-related diseases |
| *KCNMA1* | 10 | 78609359 | 79417577 | 2998 | 3.81 | 6.85E-05 | Novel gene |
| *PRDX6* | 1 | 173426486 | 173477946 | 180 | 3.79 | 7.46E-05 | Novel gene |
| *MCCC2* | 5 | 70863115 | 70974533 | 362 | 3.77 | 8.25E-05 | Novel gene |
| *SYT2* | 1 | 202539724 | 202699551 | 456 | 3.72 | 9.88E-05 | Novel gene |
| *CELSR1* | 22 | 46736731 | 46953067 | 1155 | 3.72 | 9.95E-05 | Novel gene |
| *ZNF235* | 19 | 44770501 | 44829606 | 196 | 3.70 | 1.08E-04 | Novel gene |
| *UBTF* | 17 | 42262401 | 42318994 | 123 | 3.68 | 1.18E-04 | Novel gene |
| *PKDREJ* | 22 | 46631560 | 46679277 | 196 | 3.65 | 1.32E-04 | Novel gene |
| *SMIM21* | 18 | 73101827 | 73159589 | 218 | 3.64 | 1.37E-04 | Novel gene |
| *CSHL1* | 17 | 61966965 | 62008688 | 131 | 3.62 | 1.50E-04 | Novel gene |
| *ZNF831* | 20 | 57715895 | 57854168 | 478 | 3.59 | 1.68E-04 | Novel gene |
| *LIMD2* | 17 | 61753249 | 61798527 | 105 | 3.54 | 1.99E-04 | Novel gene |
| *MYO1F* | 19 | 8565674 | 8662331 | 370 | 3.52 | 2.13E-04 | Reported gene on lung-related diseases |
| *TMC1* | 9 | 75116717 | 75471267 | 1215 | 3.52 | 2.17E-04 | Novel gene |
| *GSTA2* | 6 | 52594885 | 52648361 | 171 | 3.51 | 2.22E-04 | Novel gene |
| *SLC4A1* | 17 | 42305758 | 42371654 | 174 | 3.49 | 2.38E-04 | Novel gene |
| *ZNF233* | 19 | 44744033 | 44799470 | 167 | 3.47 | 2.57E-04 | Novel gene |
| *SOX1* | 13 | 112701913 | 112746020 | 104 | 3.44 | 2.95E-04 | Novel gene |
| *ZNF75A* | 16 | 3335406 | 3392935 | 163 | 3.42 | 3.16E-04 | Novel gene |
| *PROP1* | 5 | 177399236 | 177443243 | 135 | 3.41 | 3.30E-04 | Novel gene |
| *SLC15A3* | 11 | 60684555 | 60739257 | 219 | 3.40 | 3.37E-04 | Novel gene |
| *CACNA1G* | 17 | 48618429 | 48724835 | 381 | 3.40 | 3.42E-04 | Novel gene |
| *MICAL2* | 11 | 12112138 | 12305335 | 924 | 3.38 | 3.58E-04 | Reported gene on lung-related diseases |
| *LCA5* | 6 | 80174708 | 80267147 | 270 | 3.35 | 4.03E-04 | Novel gene |
| *SBSPON* | 8 | 73956778 | 74025507 | 274 | 3.34 | 4.25E-04 | Reported gene on lung-related diseases |
| *SPATA20* | 17 | 48604450 | 48653213 | 201 | 3.32 | 4.55E-04 | Novel gene |
| *SDHAF3* | 7 | 96725905 | 96831075 | 274 | 3.30 | 4.88E-04 | Novel gene |
| *AHCYL1* | 1 | 110507387 | 110586364 | 186 | 3.29 | 4.99E-04 | Novel gene |
| *STRIP1* | 1 | 110554199 | 110617263 | 158 | 3.28 | 5.13E-04 | Novel gene |
| *PTER* | 10 | 16458942 | 16575744 | 483 | 3.27 | 5.29E-04 | Novel gene |
| *KIAA0040* | 1 | 175106123 | 175182229 | 299 | 3.27 | 5.47E-04 | Reported gene on lung-related diseases |
| *ATF5* | 19 | 50411959 | 50457193 | 180 | 3.27 | 5.47E-04 | Novel gene |
| *C14orf159* | 14 | 91506677 | 91711976 | 739 | 3.26 | 5.65E-04 | Novel gene |
| *BTBD8* | 1 | 92525862 | 92633397 | 343 | 3.24 | 5.95E-04 | Novel gene |
| *TP53RK* | 20 | 45293004 | 45338301 | 120 | 3.22 | 6.45E-04 | Novel gene |
| *PSMC6* | 14 | 53153894 | 53214716 | 162 | 3.21 | 6.60E-04 | Novel gene |
| *MYCBP* | 1 | 39308162 | 39359050 | 181 | 3.21 | 6.72E-04 | Novel gene |
| *PDGFRB* | 5 | 149473402 | 149555447 | 294 | 3.20 | 6.93E-04 | Novel gene |
| *NNAT* | 20 | 36129607 | 36172092 | 80 | 3.20 | 6.94E-04 | Novel gene |
| *HSPH1* | 13 | 31690762 | 31757246 | 227 | 3.19 | 7.05E-04 | Novel gene |
| *LSM14B* | 20 | 60677517 | 60730434 | 243 | 3.18 | 7.32E-04 | Novel gene |
| *TRIM15* | 6 | 30110968 | 30160473 | 478 | 3.17 | 7.50E-04 | Novel gene |
| *TENM4* | 11 | 78344328 | 79172014 | 3239 | 3.17 | 7.51E-04 | Reported gene on lung-related diseases |
| *NFATC4* | 14 | 24814885 | 24868810 | 169 | 3.16 | 7.87E-04 | Novel gene |
| *STYX* | 14 | 53176883 | 53261707 | 196 | 3.16 | 7.90E-04 | Novel gene |
| *BLCAP* | 20 | 36125819 | 36176333 | 94 | 3.15 | 8.20E-04 | Novel gene |
| *CSH1* | 17 | 61952268 | 61994027 | 163 | 3.14 | 8.32E-04 | Novel gene |
| *ZNF112* | 19 | 44810706 | 44880856 | 326 | 3.13 | 8.65E-04 | Novel gene |
| *TMEM132A* | 11 | 60671913 | 60724631 | 215 | 3.13 | 8.81E-04 | Novel gene |
| *GJA9* | 1 | 39319739 | 39367298 | 160 | 3.12 | 9.02E-04 | Novel gene |
| *ZNF197* | 3 | 44646511 | 44709963 | 125 | 3.10 | 9.53E-04 | Novel gene |
| *SLC13A3* | 20 | 45166462 | 45333124 | 480 | 3.09 | 1.00E-03 | Novel gene |
| *RSPO2* | 8 | 108891544 | 109115913 | 806 | 3.09 | 1.01E-03 | Novel gene |
| *GNPNAT1* | 14 | 53221911 | 53278386 | 114 | 3.08 | 1.02E-03 | Novel gene |
| *PDZD2* | 5 | 31619345 | 32131038 | 2105 | 3.07 | 1.08E-03 | Reported gene on respiratory-related diseases |
| *PPARA* | 22 | 46526458 | 46659653 | 344 | 3.07 | 1.08E-03 | Novel gene |
| *PSMA7* | 20 | 60691783 | 60738514 | 235 | 3.06 | 1.10E-03 | Novel gene |
| *TIGD7* | 16 | 3328808 | 3375439 | 120 | 3.04 | 1.17E-03 | Novel gene |
| *PLEKHM1* | 17 | 43493266 | 43588146 | 190 | 3.04 | 1.18E-03 | Novel gene |
| *SPATA45* | 1 | 212983483 | 213040991 | 323 | 3.04 | 1.20E-03 | Novel gene |
| *F10* | 13 | 113757113 | 113823843 | 166 | 3.03 | 1.21E-03 | Novel gene |
| *SH3BGRL2* | 6 | 80321000 | 80433369 | 402 | 3.03 | 1.24E-03 | Novel gene |
| *GLP1R* | 6 | 38996557 | 39076657 | 388 | 3.02 | 1.24E-03 | Novel gene |
| *ZC3HC1* | 7 | 129638126 | 129711233 | 247 | 3.02 | 1.24E-03 | Novel gene |
| *ZNF479* | 7 | 57167321 | 57227571 | 185 | 3.02 | 1.26E-03 | Novel gene |
| *EPN3* | 17 | 48590043 | 48641111 | 233 | 3.01 | 1.29E-03 | Novel gene |
| *RIPK3* | 14 | 24785227 | 24829242 | 190 | 3.01 | 1.31E-03 | Novel gene |
| *NSL1* | 1 | 212879495 | 212985139 | 555 | 3.00 | 1.35E-03 | Novel gene |
| *TMEM99* | 17 | 38955344 | 39012526 | 202 | 3.00 | 1.36E-03 | Novel gene |
| *KRT28* | 17 | 38928448 | 38976211 | 185 | 3.00 | 1.36E-03 | Novel gene |
| *LOC101927216* | 13 | 80596827 | 80644465 | 199 | 2.99 | 1.38E-03 | Novel gene |
| *TRAF3IP1* | 2 | 239209185 | 239329541 | 448 | 2.99 | 1.38E-03 | Reported gene on lung-related diseases |
| *KIAA1429* | 8 | 95480005 | 95585746 | 473 | 2.99 | 1.39E-03 | Novel gene |
| *ZNF263* | 16 | 3313380 | 3371401 | 152 | 2.99 | 1.40E-03 | Reported gene on lung-related diseases |
| *MED21* | 12 | 27155455 | 27203606 | 228 | 2.98 | 1.45E-03 | Novel gene |
| *NUP62* | 19 | 50390082 | 50452988 | 249 | 2.95 | 1.56E-03 | Novel gene |
| *PRRC2C* | 1 | 171434652 | 171582650 | 506 | 2.95 | 1.57E-03 | Novel gene |
| *NDUFB2* | 7 | 140376481 | 140426446 | 102 | 2.95 | 1.59E-03 | Novel gene |
| *TSGA13* | 7 | 130333486 | 130392268 | 187 | 2.94 | 1.66E-03 | Novel gene |
| *C11orf16* | 11 | 8921623 | 8974553 | 275 | 2.93 | 1.71E-03 | Novel gene |
| *UBE2U* | 1 | 64649135 | 64752969 | 384 | 2.93 | 1.72E-03 | Novel gene |
| *HRH3* | 20 | 60770017 | 60815323 | 232 | 2.92 | 1.72E-03 | Novel gene |
| *OR2AE1* | 7 | 99453685 | 99494656 | 164 | 2.92 | 1.77E-03 | Novel gene |
| *PYDC2* | 3 | 191158952 | 191199245 | 192 | 2.91 | 1.80E-03 | Novel gene |
| *STX1A* | 7 | 73093535 | 73154032 | 151 | 2.91 | 1.81E-03 | Novel gene |
| *TMEM109* | 11 | 60661351 | 60710915 | 193 | 2.91 | 1.82E-03 | Novel gene |
| *TRAK1* | 3 | 42035294 | 42287382 | 953 | 2.90 | 1.87E-03 | Novel gene |
| *CEP41* | 7 | 130013612 | 130101051 | 246 | 2.90 | 1.87E-03 | Novel gene |
| *ACTL9* | 19 | 8787751 | 8829172 | 113 | 2.90 | 1.87E-03 | Novel gene |
| *AKIP1* | 11 | 8912701 | 8961626 | 252 | 2.90 | 1.88E-03 | Novel gene |
| *CPVL* | 7 | 29015247 | 29245213 | 951 | 2.90 | 1.89E-03 | Reported gene on lung-related diseases |
| *ITGA6* | 2 | 173271954 | 173391181 | 610 | 2.88 | 1.97E-03 | Novel gene |
| *GH2* | 17 | 61937572 | 61980039 | 178 | 2.87 | 2.03E-03 | Novel gene |
| *ARHGAP27* | 17 | 43451268 | 43530282 | 225 | 2.87 | 2.08E-03 | Novel gene |
| *RRAGC* | 1 | 39283869 | 39345495 | 223 | 2.86 | 2.10E-03 | Novel gene |
| *ADGRF5* | 6 | 46800242 | 46942681 | 721 | 2.86 | 2.12E-03 | Novel gene |
| *ATP2C1* | 3 | 130549327 | 130755556 | 649 | 2.85 | 2.17E-03 | Novel gene |
| *KRT10* | 17 | 38954369 | 38998863 | 158 | 2.84 | 2.24E-03 | Novel gene |
| *ZNF35* | 3 | 44670108 | 44722283 | 97 | 2.84 | 2.27E-03 | Novel gene |
| *NUP210* | 3 | 13337730 | 13481819 | 586 | 2.82 | 2.38E-03 | Novel gene |
| *MOXD1* | 6 | 132597194 | 132742664 | 430 | 2.82 | 2.40E-03 | Novel gene |
| *RUNDC3A* | 17 | 42365786 | 42416039 | 150 | 2.81 | 2.48E-03 | Novel gene |
| *IL4I1* | 19 | 50372911 | 50452796 | 292 | 2.81 | 2.49E-03 | Novel gene |
| *PDE6D* | 2 | 232577147 | 232665974 | 241 | 2.80 | 2.54E-03 | Novel gene |
| *ALX3* | 1 | 110582997 | 110633322 | 154 | 2.80 | 2.58E-03 | Novel gene |
| *RAB18* | 10 | 27773103 | 27851166 | 301 | 2.79 | 2.60E-03 | Reported gene on respiratory-related diseases |
| *ARHGAP26* | 5 | 142129881 | 142628572 | 1493 | 2.79 | 2.60E-03 | Reported gene on lung-related diseases |
| *CPA1* | 7 | 130000290 | 130047949 | 163 | 2.79 | 2.60E-03 | Reported gene on lung-related diseases |
| *SLC9C2* | 1 | 173449604 | 173592233 | 461 | 2.79 | 2.67E-03 | Novel gene |
| *GSTA5* | 6 | 52676540 | 52730893 | 174 | 2.78 | 2.69E-03 | Novel gene |
| *COPS7B* | 2 | 232626388 | 232693958 | 160 | 2.78 | 2.70E-03 | Novel gene |
| *MICALCL* | 11 | 12288447 | 12403687 | 582 | 2.78 | 2.73E-03 | Reported gene on lung-related diseases |
| *CELSR3* | 3 | 48653896 | 48720348 | 143 | 2.77 | 2.77E-03 | Novel gene |
| *ASCL3* | 11 | 8939119 | 8984580 | 239 | 2.77 | 2.79E-03 | Novel gene |
| *CRIP2* | 14 | 105919275 | 105966507 | 82 | 2.76 | 2.87E-03 | Novel gene |
| *GTF2B* | 1 | 89298321 | 89377301 | 367 | 2.76 | 2.88E-03 | Reported gene on respiratory-related diseases |
| *PRKAB2* | 1 | 146606685 | 146664168 | 251 | 2.75 | 2.97E-03 | Novel gene |
| *AGBL3* | 7 | 134651259 | 134840530 | 687 | 2.75 | 2.98E-03 | Novel gene |
| *NEUROG2* | 4 | 113414672 | 113457328 | 156 | 2.75 | 3.00E-03 | Novel gene |
| *ZNF175* | 19 | 52054531 | 52112991 | 306 | 2.73 | 3.15E-03 | Novel gene |
| *TATDN3* | 1 | 212945170 | 213010167 | 338 | 2.73 | 3.16E-03 | Novel gene |
| *CADPS* | 3 | 62364021 | 62881064 | 2102 | 2.73 | 3.18E-03 | Reported gene on lung-related diseases |
| *TRIM10* | 6 | 30099722 | 30148711 | 555 | 2.72 | 3.29E-03 | Novel gene |
| *KIF26B* | 1 | 245298287 | 245886428 | 2479 | 2.72 | 3.31E-03 | Novel gene |
| *LRRC7* | 1 | 70013230 | 70609171 | 2047 | 2.71 | 3.32E-03 | Novel gene |
| *IL12A* | 3 | 159686623 | 159733806 | 233 | 2.71 | 3.33E-03 | Novel gene |
| *SV2B* | 15 | 91622996 | 91864539 | 993 | 2.71 | 3.37E-03 | Novel gene |
| *SLC26A6* | 3 | 48643156 | 48692926 | 108 | 2.71 | 3.41E-03 | Novel gene |
| *FCGRT* | 19 | 49995536 | 50049685 | 213 | 2.70 | 3.46E-03 | Novel gene |
| *ZNF845* | 19 | 53817002 | 53878122 | 354 | 2.70 | 3.48E-03 | Novel gene |
| *SLC2A10* | 20 | 45318279 | 45384986 | 262 | 2.69 | 3.53E-03 | Reported gene on lung-related and respiratory-related diseases |
| *CDKN1A* | 6 | 36624237 | 36675116 | 293 | 2.69 | 3.57E-03 | Novel gene |
| *KRT12* | 17 | 38997430 | 39043462 | 172 | 2.69 | 3.58E-03 | Novel gene |
| *LHX5* | 12 | 113880694 | 113929877 | 101 | 2.69 | 3.59E-03 | Novel gene |
| *STRADA* | 17 | 61760192 | 61839330 | 212 | 2.69 | 3.59E-03 | Novel gene |
| *SMPDL3A* | 6 | 123089601 | 123150865 | 218 | 2.68 | 3.64E-03 | Novel gene |
| *HTR1A* | 5 | 63235875 | 63278119 | 119 | 2.68 | 3.65E-03 | Novel gene |
| *TRIM40* | 6 | 30083885 | 30136512 | 668 | 2.68 | 3.67E-03 | Novel gene |
| *TMEM107* | 17 | 8056296 | 8099723 | 125 | 2.68 | 3.71E-03 | Novel gene |
| *TMEM89* | 3 | 48638275 | 48679189 | 97 | 2.68 | 3.71E-03 | Novel gene |
| *WBSCR22* | 7 | 73077898 | 73132551 | 168 | 2.68 | 3.71E-03 | Novel gene |
| *C12orf71* | 12 | 27213990 | 27255455 | 259 | 2.67 | 3.77E-03 | Novel gene |
| *TH* | 11 | 2165159 | 2213107 | 242 | 2.66 | 3.87E-03 | Novel gene |
| *ZNF660* | 3 | 44606456 | 44661186 | 136 | 2.66 | 3.92E-03 | Novel gene |
| *KLF14* | 7 | 130397382 | 130438860 | 148 | 2.66 | 3.96E-03 | Novel gene |
| *LAMTOR3* | 4 | 100779495 | 100835703 | 128 | 2.65 | 3.98E-03 | Novel gene |
| *PYY* | 17 | 42010101 | 42101837 | 312 | 2.65 | 4.02E-03 | Novel gene |
| *B3GNT4* | 12 | 122668228 | 122712677 | 136 | 2.65 | 4.07E-03 | Novel gene |
| *BATF* | 14 | 75968784 | 76033335 | 202 | 2.65 | 4.08E-03 | Reported gene on respiratory-related diseases |
| *PRDM4* | 12 | 108106643 | 108175049 | 254 | 2.64 | 4.09E-03 | Novel gene |
| *ARID5A* | 2 | 97182464 | 97238375 | 113 | 2.64 | 4.12E-03 | Novel gene |
| *FLVCR1* | 1 | 213011597 | 213092705 | 472 | 2.64 | 4.19E-03 | Novel gene |
| *GABRB1* | 4 | 47013295 | 47452801 | 1468 | 2.63 | 4.23E-03 | Novel gene |
| *PKN2* | 1 | 89129876 | 89321938 | 831 | 2.63 | 4.24E-03 | Reported gene on respiratory-related diseases |
| *LOC101928592* | 3 | 44704574 | 44746283 | 90 | 2.63 | 4.26E-03 | Novel gene |
| *ANAPC7* | 12 | 110790705 | 110861535 | 242 | 2.63 | 4.28E-03 | Novel gene |
| *C5AR2* | 19 | 47815404 | 47865272 | 182 | 2.63 | 4.30E-03 | Novel gene |
| *RASSF4* | 10 | 45435183 | 45511337 | 317 | 2.63 | 4.33E-03 | Novel gene |
| *CDH17* | 8 | 95119394 | 95249531 | 638 | 2.62 | 4.33E-03 | Reported gene on respiratory-related diseases |
| *VPS29* | 12 | 110909330 | 110959916 | 155 | 2.62 | 4.37E-03 | Novel gene |
| *BTBD9* | 6 | 38116227 | 38628202 | 2175 | 2.62 | 4.38E-03 | Novel gene |
| *KRT27* | 17 | 38913060 | 38958786 | 181 | 2.62 | 4.39E-03 | Novel gene |
| *SUCO* | 1 | 172481494 | 172600973 | 303 | 2.62 | 4.40E-03 | Novel gene |
| *RCN3* | 19 | 50010875 | 50066891 | 211 | 2.62 | 4.41E-03 | Novel gene |
| *EGR3* | 8 | 22525172 | 22570815 | 112 | 2.62 | 4.42E-03 | Novel gene |
| *NXPE4* | 11 | 114421313 | 114486484 | 229 | 2.62 | 4.42E-03 | Novel gene |
| *KLHL20* | 1 | 173664063 | 173775960 | 289 | 2.62 | 4.43E-03 | Novel gene |
| *SEMA3C* | 7 | 80351854 | 80571695 | 618 | 2.61 | 4.48E-03 | Novel gene |
| *ARHGEF10L* | 1 | 17826693 | 18044370 | 564 | 2.61 | 4.48E-03 | Novel gene |
| *SETD9* | 5 | 56185087 | 56241359 | 220 | 2.61 | 4.48E-03 | Novel gene |
| *KIAA1107* | 1 | 92612609 | 92670280 | 179 | 2.60 | 4.64E-03 | Novel gene |
| *GPN3* | 12 | 110870291 | 110926667 | 188 | 2.60 | 4.66E-03 | Novel gene |
| *FAM167A* | 8 | 11258972 | 11344276 | 498 | 2.60 | 4.70E-03 | Novel gene |
| *CD4* | 12 | 6878638 | 6949976 | 96 | 2.60 | 4.71E-03 | Novel gene |
| *SLC25A39* | 17 | 42376993 | 42422217 | 118 | 2.59 | 4.77E-03 | Novel gene |
| *ZNF667* | 19 | 56930693 | 57008770 | 257 | 2.58 | 4.90E-03 | Novel gene |
| *IMPACT* | 18 | 21986609 | 22053499 | 277 | 2.58 | 4.95E-03 | Novel gene |
| *TNR* | 1 | 175271935 | 175732752 | 1811 | 2.58 | 4.99E-03 | Reported gene on tuberculosis |
| *TRIOBP* | 22 | 38072995 | 38192563 | 431 | 2.57 | 5.04E-03 | Novel gene |
| *PAQR8* | 6 | 52206926 | 52292575 | 396 | 2.57 | 5.05E-03 | Novel gene |
| *MS4A2* | 11 | 59835734 | 59885940 | 211 | 2.57 | 5.06E-03 | Novel gene |
| *PER1* | 17 | 8023788 | 8079723 | 189 | 2.57 | 5.11E-03 | Reported gene on lung-related diseases |
| *AURKB* | 17 | 8088049 | 8133944 | 148 | 2.57 | 5.13E-03 | Novel gene |
| *FUT5* | 19 | 5845837 | 5890551 | 211 | 2.57 | 5.14E-03 | Novel gene |
| *ADCK2* | 7 | 140352730 | 140415745 | 153 | 2.57 | 5.16E-03 | Novel gene |
| *GCLM* | 1 | 94332590 | 94395154 | 260 | 2.57 | 5.16E-03 | Novel gene |
| *SERPIND1* | 22 | 21108383 | 21162008 | 202 | 2.56 | 5.17E-03 | Novel gene |
| *NKX1-2* | 10 | 126115998 | 126158550 | 121 | 2.56 | 5.19E-03 | Novel gene |
| *ZNF559* | 19 | 9414448 | 9481838 | 224 | 2.56 | 5.22E-03 | Novel gene |
| *SORL1* | 11 | 121302912 | 121524471 | 490 | 2.56 | 5.30E-03 | Reported gene on lung-related diseases |
| *ADIRF* | 10 | 88708188 | 88750666 | 183 | 2.55 | 5.32E-03 | Novel gene |
| *VAMP2* | 17 | 8042465 | 8086293 | 127 | 2.55 | 5.38E-03 | Novel gene |
| *ZKSCAN7* | 3 | 44576667 | 44644975 | 205 | 2.55 | 5.42E-03 | Novel gene |
| *ACOT11* | 1 | 54987930 | 55120417 | 595 | 2.55 | 5.43E-03 | Novel gene |
| *CDC16* | 13 | 114980320 | 115058150 | 304 | 2.55 | 5.45E-03 | Novel gene |
| *RAB32* | 6 | 146844828 | 146896086 | 110 | 2.54 | 5.54E-03 | Novel gene |
| *PRPF19* | 11 | 60638019 | 60694061 | 163 | 2.54 | 5.54E-03 | Novel gene |
| *PDK1* | 2 | 173400101 | 173510351 | 467 | 2.54 | 5.61E-03 | Novel gene |
| *GRN* | 17 | 42402491 | 42450474 | 97 | 2.54 | 5.62E-03 | Novel gene |
| *SS18L1* | 20 | 60698816 | 60777566 | 491 | 2.53 | 5.68E-03 | Novel gene |
| *C17orf59* | 17 | 8071651 | 8113564 | 125 | 2.53 | 5.73E-03 | Novel gene |
| *TRIM77* | 11 | 89423467 | 89471040 | 140 | 2.52 | 5.80E-03 | Novel gene |
| *PTPN5* | 11 | 18729475 | 18834268 | 363 | 2.52 | 5.83E-03 | Novel gene |
| *AES* | 19 | 3032908 | 3082964 | 132 | 2.52 | 5.84E-03 | Novel gene |
| *ZNF699* | 19 | 9384246 | 9440390 | 174 | 2.52 | 5.85E-03 | Novel gene |
| *DDIT4L* | 4 | 101086267 | 101131939 | 273 | 2.51 | 6.02E-03 | Reported gene on lung-related diseases |
| *EMCN* | 4 | 101296498 | 101459250 | 614 | 2.51 | 6.02E-03 | Novel gene |
| *KDM2B* | 12 | 121846899 | 122039110 | 426 | 2.51 | 6.02E-03 | Novel gene |
| *ADAMTS10* | 19 | 8625124 | 8695588 | 166 | 2.51 | 6.03E-03 | Reported gene on lung-related diseases |
| *GLDC* | 9 | 6512464 | 6665692 | 817 | 2.51 | 6.05E-03 | Reported gene on respiratory-related diseases |
| *GGA1* | 22 | 37984481 | 38049571 | 286 | 2.51 | 6.07E-03 | Novel gene |
| *C2CD4D* | 1 | 151790339 | 151833033 | 147 | 2.51 | 6.07E-03 | Novel gene |
| *AGAP11* | 10 | 88708239 | 88789960 | 283 | 2.51 | 6.09E-03 | Novel gene |
| *WDR87* | 19 | 38355213 | 38417317 | 168 | 2.51 | 6.11E-03 | Novel gene |
| *LSP1* | 11 | 1854200 | 1933493 | 317 | 2.50 | 6.17E-03 | Novel gene |
| *DNAJC30* | 7 | 73075248 | 73117781 | 155 | 2.50 | 6.17E-03 | Novel gene |
| *TRIM31* | 6 | 30050674 | 30100867 | 565 | 2.50 | 6.18E-03 | Novel gene |
| *DUSP12* | 1 | 161699558 | 161746952 | 184 | 2.50 | 6.24E-03 | Novel gene |
| *TBX4* | 17 | 59509134 | 59582471 | 141 | 2.50 | 6.26E-03 | Reported gene on lung-related diseases |
| *MKL2* | 16 | 14145178 | 14380633 | 710 | 2.49 | 6.37E-03 | Novel gene |
| *CYP3A43* | 7 | 99405636 | 99483727 | 243 | 2.49 | 6.39E-03 | Novel gene |
| *FOLH1B* | 11 | 89350675 | 89451886 | 425 | 2.49 | 6.48E-03 | Novel gene |
| *TPCN1* | 12 | 113639260 | 113756390 | 412 | 2.48 | 6.64E-03 | Novel gene |
| *GIF* | 11 | 59576746 | 59632974 | 156 | 2.47 | 6.71E-03 | Novel gene |
| *ABCA5* | 17 | 67220576 | 67343323 | 324 | 2.47 | 6.78E-03 | Novel gene |
| *ZNF559-ZNF177* | 19 | 9414902 | 9513293 | 321 | 2.47 | 6.81E-03 | Novel gene |
| *CA9* | 9 | 35653915 | 35701156 | 175 | 2.46 | 6.96E-03 | Novel gene |
| *ZNF414* | 19 | 8555462 | 8599048 | 185 | 2.46 | 7.03E-03 | Novel gene |
| *FABP7* | 6 | 123080646 | 123125219 | 157 | 2.46 | 7.04E-03 | Novel gene |
| *MFNG* | 22 | 37845101 | 37902499 | 133 | 2.45 | 7.05E-03 | Novel gene |
| *SNCG* | 10 | 88697504 | 88743017 | 173 | 2.45 | 7.06E-03 | Novel gene |
| *MIER3* | 5 | 56195429 | 56267957 | 319 | 2.45 | 7.23E-03 | Novel gene |
| *KIAA0195* | 17 | 73432664 | 73516533 | 331 | 2.44 | 7.30E-03 | Novel gene |
| *IQCD* | 12 | 113613246 | 113679086 | 223 | 2.44 | 7.32E-03 | Novel gene |
| *HTR7* | 10 | 92480575 | 92637671 | 653 | 2.44 | 7.35E-03 | Novel gene |
| *FMO5* | 1 | 146635884 | 146717390 | 418 | 2.44 | 7.41E-03 | Novel gene |
| *F7* | 13 | 113740102 | 113794995 | 191 | 2.44 | 7.42E-03 | Novel gene |
| *FER1L5* | 2 | 97288574 | 97390624 | 171 | 2.44 | 7.43E-03 | Novel gene |
| *RPS11* | 19 | 49979622 | 50022969 | 162 | 2.43 | 7.45E-03 | Novel gene |
| *LGALS2* | 22 | 37946253 | 37996024 | 196 | 2.43 | 7.46E-03 | Novel gene |
| *KLHL26* | 19 | 18727835 | 18801308 | 274 | 2.43 | 7.46E-03 | Novel gene |
| *MYOC* | 1 | 171584557 | 171641773 | 290 | 2.43 | 7.47E-03 | Novel gene |
| *ZNF583* | 19 | 56895383 | 56956400 | 159 | 2.43 | 7.50E-03 | Novel gene |
| *GLO1* | 6 | 38623701 | 38690952 | 294 | 2.43 | 7.52E-03 | Novel gene |
| *C5AR1* | 19 | 47793104 | 47845327 | 192 | 2.43 | 7.62E-03 | Reported gene on lung-related diseases |
| *DAPP1* | 4 | 100717957 | 100811347 | 333 | 2.42 | 7.69E-03 | Novel gene |
| *EID1* | 15 | 49150290 | 49192380 | 116 | 2.42 | 7.71E-03 | Novel gene |
| *RITA1* | 12 | 113603342 | 113650169 | 166 | 2.42 | 7.71E-03 | Novel gene |
| *MAP3K3* | 17 | 61679404 | 61793670 | 224 | 2.42 | 7.74E-03 | Novel gene |
| *CHD3* | 17 | 7768096 | 7836075 | 190 | 2.42 | 7.76E-03 | Novel gene |
| *FBP2* | 9 | 97301002 | 97376075 | 436 | 2.42 | 7.86E-03 | Novel gene |
| *ATMIN* | 16 | 81049458 | 81100955 | 270 | 2.41 | 7.87E-03 | Novel gene |
| *ZP1* | 11 | 60615015 | 60663164 | 168 | 2.41 | 7.93E-03 | Novel gene |
| *ID3* | 1 | 23864421 | 23906285 | 97 | 2.41 | 8.00E-03 | Novel gene |
| *HEY2* | 6 | 126046211 | 126102415 | 139 | 2.40 | 8.15E-03 | Novel gene |
| *ASTE1* | 3 | 130712721 | 130765698 | 163 | 2.40 | 8.21E-03 | Novel gene |
| *HBS1L* | 6 | 135261516 | 135396036 | 452 | 2.40 | 8.28E-03 | Novel gene |
| *LYSMD3* | 5 | 89791443 | 89845401 | 186 | 2.39 | 8.35E-03 | Novel gene |
| *ANAPC16* | 10 | 73955758 | 74015618 | 221 | 2.39 | 8.39E-03 | Novel gene |
| *TPM2* | 9 | 35661990 | 35710583 | 151 | 2.39 | 8.47E-03 | Novel gene |
| *TEAD1* | 11 | 12675969 | 12986284 | 754 | 2.39 | 8.47E-03 | Reported gene on lung-related diseases |
| *CROCC* | 1 | 17228445 | 17319474 | 196 | 2.38 | 8.54E-03 | Novel gene |
| *COMMD9* | 11 | 36273842 | 36330999 | 261 | 2.38 | 8.55E-03 | Novel gene |
| *CSH2* | 17 | 61929372 | 61971089 | 188 | 2.38 | 8.61E-03 | Novel gene |
| *CRH* | 8 | 67068612 | 67110846 | 149 | 2.38 | 8.62E-03 | Novel gene |
| *ZNF793* | 19 | 37977323 | 38054239 | 204 | 2.38 | 8.65E-03 | Novel gene |
| *MTG2* | 20 | 60738081 | 60798624 | 383 | 2.38 | 8.69E-03 | Novel gene |
| *KIF2B* | 17 | 51880239 | 51922573 | 221 | 2.38 | 8.71E-03 | Novel gene |
| *FAR2* | 12 | 29281936 | 29508549 | 1020 | 2.38 | 8.72E-03 | Novel gene |
| *TRIM55* | 8 | 67019131 | 67107720 | 326 | 2.38 | 8.75E-03 | Novel gene |
| *IKBKE* | 1 | 206623586 | 206690223 | 244 | 2.38 | 8.75E-03 | Novel gene |
| *GALK2* | 15 | 49427974 | 49679955 | 820 | 2.38 | 8.75E-03 | Reported gene on lung-related diseases |
| *GGTLC1* | 20 | 23945687 | 23989475 | 174 | 2.37 | 8.80E-03 | Novel gene |
| *SLC30A1* | 1 | 211728381 | 211772099 | 159 | 2.37 | 8.88E-03 | Reported gene on respiratory-related diseases |
| *ZNF74* | 22 | 20728405 | 20782753 | 222 | 2.37 | 8.91E-03 | Novel gene |
| *USH2A* | 1 | 215776236 | 216616738 | 2953 | 2.37 | 8.97E-03 | Novel gene |
| *MUC16* | 19 | 8939516 | 9112018 | 769 | 2.37 | 9.00E-03 | Novel gene |
| *HLA-DRA* | 6 | 32387619 | 32432823 | 772 | 2.36 | 9.17E-03 | Reported gene on tuberculosis, lung-related and respiratory-related diseases |
| *C1orf146* | 1 | 92663573 | 92731367 | 198 | 2.36 | 9.18E-03 | Novel gene |
| *HVCN1* | 12 | 111066491 | 111150276 | 243 | 2.36 | 9.21E-03 | Novel gene |
| *DNTTIP2* | 1 | 94315014 | 94365474 | 212 | 2.36 | 9.26E-03 | Novel gene |
| *GDPD4* | 11 | 76907603 | 77032699 | 561 | 2.35 | 9.27E-03 | Novel gene |
| *OLFM2* | 19 | 9944394 | 10067070 | 409 | 2.35 | 9.42E-03 | Novel gene |
| *ZNF560* | 19 | 9557031 | 9629279 | 242 | 2.35 | 9.48E-03 | Novel gene |
| *CDC42EP1* | 22 | 37936471 | 37985412 | 178 | 2.35 | 9.48E-03 | Novel gene |
| *DTWD2* | 5 | 118152569 | 118344240 | 686 | 2.34 | 9.58E-03 | Novel gene |
| *ARHGEF39* | 9 | 35638872 | 35695863 | 232 | 2.34 | 9.61E-03 | Novel gene |
| *ALDH1B1* | 9 | 38372661 | 38418662 | 247 | 2.34 | 9.62E-03 | Novel gene |
| *ZFP1* | 16 | 75162392 | 75226132 | 290 | 2.34 | 9.70E-03 | Novel gene |
| *TIAF1* | 17 | 27380539 | 27422627 | 127 | 2.34 | 9.71E-03 | Novel gene |
| *KNOP1* | 16 | 19697673 | 19749726 | 208 | 2.34 | 9.74E-03 | Novel gene |
| *TRIL* | 7 | 28972974 | 29018029 | 203 | 2.33 | 9.88E-03 | Novel gene |
| *CDH4* | 20 | 59807482 | 60535673 | 3561 | 2.33 | 9.94E-03 | Reported gene on respiratory-related diseases |
| *HOXB1* | 17 | 46586807 | 46628272 | 150 | 2.32 | 1.01E-02 | Reported gene on lung-related diseases |
| *ARPC3* | 12 | 110852695 | 110908222 | 177 | 2.32 | 1.01E-02 | Novel gene |
| *DDX54* | 12 | 113574978 | 113643284 | 231 | 2.32 | 1.02E-02 | Novel gene |
| *TEX22* | 14 | 105844920 | 105900196 | 28 | 2.32 | 1.02E-02 | Novel gene |
| *OAT* | 10 | 126065872 | 126127545 | 233 | 2.32 | 1.03E-02 | Novel gene |
| *FAM162B* | 6 | 117053360 | 117106886 | 144 | 2.32 | 1.03E-02 | Novel gene |
| *CNTN5* | 11 | 98871706 | 100249616 | 7006 | 2.31 | 1.04E-02 | Reported gene on lung-related and respiratory-related diseases |
| *NOL12* | 22 | 38057680 | 38109718 | 214 | 2.31 | 1.04E-02 | Novel gene |
| *F2RL1* | 5 | 76094833 | 76151140 | 174 | 2.31 | 1.04E-02 | Novel gene |
| *IGSF9B* | 11 | 133758520 | 133846649 | 399 | 2.31 | 1.05E-02 | Novel gene |
| *ITPRIP* | 10 | 106049454 | 106118251 | 214 | 2.31 | 1.06E-02 | Novel gene |
| *KIAA0226L* | 13 | 46896136 | 46984177 | 420 | 2.31 | 1.06E-02 | Novel gene |
| *SELV* | 19 | 39985753 | 40031326 | 185 | 2.30 | 1.07E-02 | Novel gene |
| *BMP7* | 20 | 55723809 | 55861707 | 578 | 2.30 | 1.07E-02 | Novel gene |
| *ATXN7L3* | 17 | 42249173 | 42295747 | 105 | 2.30 | 1.08E-02 | Novel gene |
| *MMRN2* | 10 | 88675297 | 88737425 | 217 | 2.30 | 1.08E-02 | Novel gene |
| *ZNF266* | 19 | 9503102 | 9566254 | 262 | 2.29 | 1.09E-02 | Novel gene |
| *PCNA* | 20 | 5075599 | 5127268 | 117 | 2.29 | 1.09E-02 | Novel gene |
| *RAB44* | 6 | 36645628 | 36720960 | 415 | 2.29 | 1.10E-02 | Novel gene |
| *TMEM9B* | 11 | 8948840 | 9006553 | 275 | 2.28 | 1.12E-02 | Novel gene |
| *BATF3* | 1 | 212839759 | 212893327 | 258 | 2.28 | 1.13E-02 | Novel gene |
| *ITGAV* | 2 | 187434058 | 187565629 | 298 | 2.28 | 1.13E-02 | Reported gene on lung-related diseases |
| *FOXN3* | 14 | 89602516 | 90105506 | 1527 | 2.28 | 1.13E-02 | Novel gene |
| *FAM216A* | 12 | 110886232 | 110948192 | 194 | 2.28 | 1.14E-02 | Novel gene |
| *ST5* | 11 | 8694899 | 8952498 | 1012 | 2.28 | 1.14E-02 | Reported gene on lung-related diseases |
| *TDRKH* | 1 | 151722741 | 151783052 | 190 | 2.27 | 1.17E-02 | Novel gene |
| *MORN2* | 2 | 39083103 | 39129850 | 159 | 2.27 | 1.17E-02 | Novel gene |
| *FAM151A* | 1 | 55054850 | 55109200 | 249 | 2.27 | 1.18E-02 | Novel gene |
| *LILRB4* | 19 | 55154271 | 55201810 | 213 | 2.26 | 1.18E-02 | Novel gene |
| *DHX57* | 2 | 39004871 | 39123021 | 362 | 2.26 | 1.19E-02 | Novel gene |
| *MMP15* | 16 | 58039282 | 58100805 | 211 | 2.26 | 1.19E-02 | Reported gene on lung-related and respiratory-related diseases |
| *KANSL1* | 17 | 44087282 | 44322740 | 745 | 2.26 | 1.19E-02 | Reported gene on lung-related and respiratory-related diseases |
| *C10orf10* | 10 | 45451709 | 45494330 | 176 | 2.26 | 1.20E-02 | Novel gene |
| *PPP1CC* | 12 | 111137613 | 111200783 | 200 | 2.26 | 1.20E-02 | Novel gene |
| *MTA1* | 14 | 105866186 | 105957066 | 97 | 2.26 | 1.20E-02 | Novel gene |
| *SLC6A17* | 1 | 110673132 | 110764824 | 201 | 2.26 | 1.20E-02 | Novel gene |
| *TMEM244* | 6 | 130132389 | 130202416 | 409 | 2.25 | 1.22E-02 | Novel gene |
| *CRYGA* | 2 | 209005464 | 209048366 | 241 | 2.25 | 1.23E-02 | Novel gene |
| *ZNF502* | 3 | 44734135 | 44785323 | 167 | 2.25 | 1.23E-02 | Novel gene |
| *LRRC41* | 1 | 46724072 | 46789038 | 110 | 2.25 | 1.23E-02 | Novel gene |
| *TNN* | 1 | 175016994 | 175137202 | 492 | 2.25 | 1.23E-02 | Novel gene |
| *CYP11B1* | 8 | 143933773 | 143981236 | 188 | 2.24 | 1.24E-02 | Novel gene |
| *DEFB104A* | 8 | 7673993 | 7718764 | 3 | 2.24 | 1.24E-02 | Novel gene |
| *SPAG11A* | 8 | 7685209 | 7741319 | 3 | 2.24 | 1.24E-02 | Novel gene |
| *NCKIPSD* | 3 | 48680419 | 48743366 | 126 | 2.24 | 1.25E-02 | Novel gene |
| *DEFB110* | 6 | 49956851 | 50009694 | 168 | 2.24 | 1.25E-02 | Novel gene |
| *C6orf165* | 6 | 88097690 | 88194191 | 497 | 2.24 | 1.26E-02 | Novel gene |
| *FEZ2* | 2 | 36759397 | 36845333 | 358 | 2.24 | 1.26E-02 | Novel gene |
| *PYCR1* | 17 | 79870260 | 79915203 | 87 | 2.24 | 1.27E-02 | Novel gene |
| *TSSK1B* | 5 | 112748251 | 112790728 | 223 | 2.24 | 1.27E-02 | Novel gene |
| *KANSL3* | 2 | 97238892 | 97324116 | 147 | 2.23 | 1.27E-02 | Novel gene |
| *HSPA13* | 21 | 15723436 | 15775509 | 244 | 2.23 | 1.27E-02 | Novel gene |
| *CAND2* | 3 | 12818171 | 12896313 | 336 | 2.23 | 1.28E-02 | Reported gene on lung-related diseases |
| *OAZ3* | 1 | 151715445 | 151763806 | 150 | 2.23 | 1.28E-02 | Novel gene |
| *SFMBT1* | 3 | 52913221 | 53100089 | 570 | 2.23 | 1.28E-02 | Novel gene |
| *ZNF33B* | 10 | 43064532 | 43154016 | 358 | 2.23 | 1.28E-02 | Novel gene |
| *STH* | 17 | 44056616 | 44097060 | 241 | 2.23 | 1.29E-02 | Reported gene on respiratory-related diseases |
| *ZNF852* | 3 | 44519924 | 44572222 | 110 | 2.23 | 1.29E-02 | Novel gene |
| *COPG2* | 7 | 130126079 | 130373598 | 431 | 2.23 | 1.29E-02 | Novel gene |
| *CD82* | 11 | 44567141 | 44661339 | 389 | 2.23 | 1.29E-02 | Novel gene |
| *RNF34* | 12 | 121817886 | 121882155 | 198 | 2.23 | 1.29E-02 | Novel gene |
| *ZNF735* | 7 | 63647581 | 63700668 | 315 | 2.23 | 1.30E-02 | Novel gene |
| *MAP1S* | 19 | 17810303 | 17865324 | 238 | 2.23 | 1.30E-02 | Novel gene |
| *DEFB112* | 6 | 49991288 | 50036364 | 168 | 2.22 | 1.31E-02 | Novel gene |
| *BRIP1* | 17 | 59736547 | 59960920 | 477 | 2.22 | 1.32E-02 | Reported gene on lung-related diseases |
| *CEP152* | 15 | 49010135 | 49124092 | 258 | 2.22 | 1.32E-02 | Novel gene |
| *LACC1* | 13 | 44433420 | 44488068 | 254 | 2.22 | 1.32E-02 | Novel gene |
| *CCT6B* | 17 | 33234878 | 33327968 | 206 | 2.22 | 1.32E-02 | Novel gene |
| *SLC22A23* | 6 | 3249207 | 3476954 | 1032 | 2.22 | 1.33E-02 | Novel gene |
| *PAK7* | 20 | 9498037 | 9839687 | 1185 | 2.22 | 1.33E-02 | Novel gene |
| *SLC6A1* | 3 | 11014420 | 11100935 | 296 | 2.22 | 1.33E-02 | Novel gene |
| *HNRNPH1* | 5 | 179021179 | 179070722 | 244 | 2.21 | 1.34E-02 | Novel gene |
| *TM7SF3* | 12 | 27104503 | 27187360 | 277 | 2.21 | 1.36E-02 | Novel gene |
| *ZSWIM2* | 2 | 187672207 | 187733897 | 241 | 2.21 | 1.36E-02 | Reported gene on lung-related diseases |
| *ARHGAP29* | 1 | 94614463 | 94733251 | 321 | 2.21 | 1.36E-02 | Novel gene |
| *TMEM177* | 2 | 120416743 | 120459694 | 202 | 2.21 | 1.36E-02 | Novel gene |
| *SCGB3A2* | 5 | 147238274 | 147281756 | 194 | 2.21 | 1.37E-02 | Novel gene |
| *PPM1A* | 14 | 60692470 | 60785805 | 230 | 2.21 | 1.37E-02 | Novel gene |
| *NOSIP* | 19 | 50038725 | 50103845 | 246 | 2.21 | 1.37E-02 | Novel gene |
| *UBR2* | 6 | 42511748 | 42681243 | 374 | 2.20 | 1.37E-02 | Reported gene on lung-related diseases |
| *MAPT* | 17 | 43951702 | 44125700 | 951 | 2.20 | 1.38E-02 | Reported gene on lung-related and respiratory-related diseases |
| *SCAPER* | 15 | 76620526 | 77217796 | 1402 | 2.20 | 1.38E-02 | Novel gene |
| *ENKUR* | 10 | 25250908 | 25371208 | 493 | 2.20 | 1.38E-02 | Novel gene |
| *CUL4A* | 13 | 113842507 | 113939392 | 309 | 2.20 | 1.39E-02 | Novel gene |
| *CYP11B2* | 8 | 143971975 | 144019259 | 196 | 2.20 | 1.39E-02 | Novel gene |
| *GBP7* | 1 | 89577434 | 89661723 | 291 | 2.20 | 1.40E-02 | Novel gene |
| *HES7* | 17 | 8003908 | 8047410 | 163 | 2.20 | 1.40E-02 | Novel gene |
| *ZNF806* | 2 | 133044717 | 133096320 | 1 | 2.20 | 1.40E-02 | Novel gene |
| *EHF* | 11 | 34622588 | 34704835 | 246 | 2.20 | 1.40E-02 | Reported gene on lung-related and respiratory-related diseases |
| *C16orf46* | 16 | 81067102 | 81130872 | 422 | 2.19 | 1.41E-02 | Novel gene |
| *RUFY1* | 5 | 178957562 | 179057027 | 478 | 2.19 | 1.42E-02 | Reported gene on lung-related diseases |
| *KCNAB2* | 1 | 6032358 | 6181253 | 541 | 2.19 | 1.42E-02 | Novel gene |
| *RFFL* | 17 | 33316131 | 33436348 | 260 | 2.19 | 1.43E-02 | Novel gene |
| *RHOG* | 11 | 3828208 | 3882213 | 162 | 2.19 | 1.44E-02 | Novel gene |
| *PWP1* | 12 | 108059590 | 108126257 | 225 | 2.18 | 1.45E-02 | Novel gene |
| *PTPRB* | 12 | 70890630 | 71051220 | 711 | 2.18 | 1.45E-02 | Novel gene |
| *TCP11L2* | 12 | 106676570 | 106761365 | 288 | 2.18 | 1.46E-02 | Novel gene |
| *CLN8* | 8 | 1683944 | 1754736 | 316 | 2.18 | 1.46E-02 | Novel gene |
| *PI4KA* | 22 | 21041979 | 21233100 | 602 | 2.18 | 1.46E-02 | Novel gene |
| *C2orf80* | 2 | 209010071 | 209074969 | 337 | 2.18 | 1.47E-02 | Novel gene |
| *KCNC1* | 11 | 17737495 | 17824602 | 257 | 2.18 | 1.48E-02 | Novel gene |
| *DNAJB14* | 4 | 100797405 | 100887883 | 214 | 2.18 | 1.48E-02 | Novel gene |
| *MAFG* | 17 | 79856145 | 79905587 | 68 | 2.17 | 1.48E-02 | Novel gene |
| *TM4SF1* | 3 | 149066805 | 149115647 | 168 | 2.17 | 1.49E-02 | Novel gene |
| *POLR3G* | 5 | 89749876 | 89830474 | 235 | 2.17 | 1.49E-02 | Novel gene |
| *TSTA3* | 8 | 144674788 | 144720358 | 184 | 2.17 | 1.50E-02 | Novel gene |
| *CALD1* | 7 | 134444164 | 134675480 | 816 | 2.17 | 1.50E-02 | Reported gene on lung-related diseases |
| *ZNF384* | 12 | 6755643 | 6818738 | 129 | 2.17 | 1.50E-02 | Novel gene |
| *SNAP29* | 22 | 21193292 | 21265502 | 249 | 2.17 | 1.51E-02 | Novel gene |
| *LIPG* | 18 | 47067069 | 47139278 | 253 | 2.17 | 1.51E-02 | Novel gene |
| *GRB10* | 7 | 50637760 | 50881159 | 906 | 2.17 | 1.52E-02 | Reported gene on respiratory-related diseases |
| *USP33* | 1 | 78141672 | 78245564 | 279 | 2.16 | 1.53E-02 | Novel gene |
| *TCP11L1* | 11 | 33040963 | 33115109 | 354 | 2.16 | 1.53E-02 | Novel gene |
| *PPP1R16B* | 20 | 37414348 | 37571667 | 529 | 2.16 | 1.53E-02 | Novel gene |
| *CST2* | 20 | 23784404 | 23827312 | 339 | 2.16 | 1.53E-02 | Novel gene |
| *GJB3* | 1 | 35226790 | 35271967 | 227 | 2.16 | 1.54E-02 | Novel gene |
| *PAPD5* | 16 | 50166829 | 50289221 | 314 | 2.16 | 1.54E-02 | Reported gene on lung-related diseases |
| *USP41* | 22 | 20684868 | 20751544 | 63 | 2.16 | 1.54E-02 | Novel gene |
| *CYP8B1* | 3 | 42893684 | 42937633 | 164 | 2.16 | 1.54E-02 | Novel gene |
| *NDNL2* | 15 | 29540353 | 29582020 | 140 | 2.16 | 1.55E-02 | Novel gene |
| *PCDH9* | 13 | 66856966 | 67824468 | 2630 | 2.15 | 1.56E-02 | Reported gene on respiratory-related diseases |
| *APOBEC3D* | 22 | 39397118 | 39455686 | 183 | 2.15 | 1.57E-02 | Novel gene |
| *FGF12* | 3 | 191837033 | 192465388 | 2124 | 2.15 | 1.58E-02 | Novel gene |
| *TRIM26* | 6 | 30132232 | 30201271 | 558 | 2.15 | 1.59E-02 | Reported gene on lung-related diseases |
| *EAF2* | 3 | 121534030 | 121625373 | 276 | 2.15 | 1.59E-02 | Novel gene |
| *ZNF177* | 19 | 9453696 | 9513293 | 182 | 2.15 | 1.60E-02 | Novel gene |
| *TPTE2* | 13 | 19977017 | 20155714 | 777 | 2.14 | 1.60E-02 | Novel gene |
| *IMPA2* | 18 | 11961427 | 12050885 | 486 | 2.14 | 1.60E-02 | Novel gene |
| *KIRREL3* | 11 | 126273388 | 126890766 | 2582 | 2.14 | 1.61E-02 | Novel gene |
| *MFSD10* | 4 | 2912288 | 2956586 | 280 | 2.14 | 1.61E-02 | Novel gene |
| *CSF1R* | 5 | 149412854 | 149512935 | 406 | 2.14 | 1.61E-02 | Novel gene |
| *KAZALD1* | 10 | 102800999 | 102847888 | 135 | 2.14 | 1.61E-02 | Novel gene |
| *CCDC108* | 2 | 219847568 | 219926273 | 168 | 2.14 | 1.62E-02 | Novel gene |
| *ST6GALNAC3* | 1 | 76520389 | 77120311 | 2185 | 2.14 | 1.62E-02 | Novel gene |
| *PPY* | 17 | 41998172 | 42039833 | 153 | 2.14 | 1.62E-02 | Novel gene |
| *GATA2* | 3 | 128178265 | 128232030 | 222 | 2.14 | 1.63E-02 | Reported gene on respiratory-related diseases |
| *LINGO4* | 1 | 151752765 | 151797882 | 128 | 2.14 | 1.63E-02 | Novel gene |
| *NOL9* | 1 | 6561407 | 6634658 | 325 | 2.14 | 1.63E-02 | Novel gene |
| *CTTNBP2NL* | 1 | 112918800 | 113023786 | 251 | 2.14 | 1.64E-02 | Reported gene on lung-related diseases |
| *SH3BP1* | 22 | 38010661 | 38072119 | 275 | 2.14 | 1.64E-02 | Novel gene |
| *SPATA33* | 16 | 89704152 | 89756866 | 329 | 2.13 | 1.65E-02 | Novel gene |
| *RPL13A* | 19 | 49970811 | 50015565 | 161 | 2.13 | 1.65E-02 | Novel gene |
| *CD79B* | 17 | 61986098 | 62029710 | 152 | 2.13 | 1.65E-02 | Novel gene |
| *FAM153B* | 5 | 175470712 | 175561801 | 46 | 2.13 | 1.65E-02 | Novel gene |
| *ESRP1* | 8 | 95633364 | 95739694 | 470 | 2.13 | 1.67E-02 | Novel gene |
| *RANBP3* | 19 | 5896150 | 5998320 | 316 | 2.13 | 1.67E-02 | Reported gene on lung-related diseases |
| *WFIKKN2* | 17 | 48892011 | 48939709 | 183 | 2.12 | 1.68E-02 | Novel gene |
| *PTS* | 11 | 112077088 | 112124696 | 169 | 2.12 | 1.68E-02 | Novel gene |
| *SETSIP* | 1 | 92520093 | 92560968 | 102 | 2.12 | 1.68E-02 | Novel gene |
| *ICE1* | 5 | 5402786 | 5510347 | 301 | 2.12 | 1.69E-02 | Novel gene |
| *LYPD2* | 8 | 143811628 | 143853952 | 157 | 2.12 | 1.69E-02 | Novel gene |
| *MRPS30* | 5 | 44789027 | 44835616 | 108 | 2.12 | 1.70E-02 | Novel gene |
| *PIP4K2C* | 12 | 57964942 | 58017211 | 114 | 2.12 | 1.71E-02 | Novel gene |
| *PLEKHF1* | 19 | 30136327 | 30186384 | 182 | 2.11 | 1.72E-02 | Novel gene |
| *EYS* | 6 | 64409876 | 66437118 | 8387 | 2.11 | 1.73E-02 | Novel gene |
| *C1GALT1* | 7 | 7202178 | 7308282 | 706 | 2.11 | 1.73E-02 | Reported gene on lung-related and respiratory-related diseases |
| *NIPAL1* | 4 | 47998791 | 48059084 | 233 | 2.11 | 1.73E-02 | Novel gene |
| *CENPL* | 1 | 173748688 | 173814325 | 151 | 2.11 | 1.73E-02 | Novel gene |
| *PEX2* | 8 | 77872494 | 77933280 | 185 | 2.11 | 1.74E-02 | Novel gene |
| *SNCAIP* | 5 | 121627455 | 121819794 | 309 | 2.11 | 1.75E-02 | Novel gene |
| *SRR* | 17 | 2187244 | 2248553 | 204 | 2.11 | 1.75E-02 | Novel gene |
| *LRRC43* | 12 | 122632266 | 122708018 | 266 | 2.11 | 1.75E-02 | Novel gene |
| *DEPDC7* | 11 | 33017410 | 33075128 | 258 | 2.11 | 1.75E-02 | Reported gene on lung-related diseases |
| *PRDM1* | 6 | 106514195 | 106577814 | 167 | 2.11 | 1.76E-02 | Novel gene |
| *H2AFZ* | 4 | 100849242 | 100891512 | 112 | 2.11 | 1.76E-02 | Novel gene |
| *FAM46A* | 6 | 82346829 | 82482607 | 394 | 2.11 | 1.76E-02 | Novel gene |
| *KIF23* | 15 | 69686585 | 69760766 | 181 | 2.10 | 1.77E-02 | Novel gene |
| *NOTUM* | 17 | 79890383 | 79939057 | 128 | 2.10 | 1.77E-02 | Novel gene |
| *CHMP1A* | 16 | 89690839 | 89744193 | 308 | 2.10 | 1.77E-02 | Novel gene |
| *CDS2* | 20 | 5087407 | 5198533 | 287 | 2.10 | 1.78E-02 | Novel gene |
| *COPS2* | 15 | 49397471 | 49467854 | 220 | 2.10 | 1.78E-02 | Reported gene on lung-related and respiratory-related diseases |
| *PAK1* | 11 | 77013060 | 77205108 | 698 | 2.10 | 1.78E-02 | Novel gene |
| *SLC4A8* | 12 | 51765101 | 51929547 | 402 | 2.10 | 1.79E-02 | Novel gene |
| *YTHDC2* | 5 | 112829391 | 112950984 | 611 | 2.10 | 1.80E-02 | Novel gene |
| *C9orf69* | 9 | 138986427 | 139030731 | 160 | 2.10 | 1.80E-02 | Novel gene |
| *TOB1* | 17 | 48919584 | 48965339 | 123 | 2.10 | 1.80E-02 | Novel gene |
| *STC1* | 8 | 23679434 | 23732320 | 212 | 2.09 | 1.81E-02 | Novel gene |
| *PRR5L* | 11 | 36297725 | 36506754 | 957 | 2.09 | 1.81E-02 | Reported gene on respiratory-related diseases |
| *LRRC37A* | 17 | 44296744 | 44435160 | 51 | 2.09 | 1.82E-02 | Novel gene |
| *MFAP2* | 1 | 17280997 | 17328081 | 151 | 2.09 | 1.82E-02 | Reported gene on lung-related and respiratory-related diseases |
| *PDXP* | 22 | 38034737 | 38082939 | 219 | 2.09 | 1.83E-02 | Novel gene |
| *DIABLO* | 12 | 122672209 | 122732081 | 186 | 2.09 | 1.84E-02 | Novel gene |
| *OR10V1* | 11 | 59460389 | 59501318 | 175 | 2.09 | 1.84E-02 | Novel gene |
| *PIWIL1* | 12 | 130802433 | 130876877 | 348 | 2.09 | 1.85E-02 | Novel gene |
| *ADRA1A* | 8 | 26585667 | 26747003 | 859 | 2.09 | 1.85E-02 | Novel gene |
| *NR2F1* | 5 | 92899043 | 92950319 | 105 | 2.08 | 1.86E-02 | Novel gene |
| *EMILIN2* | 18 | 2826786 | 2934090 | 337 | 2.08 | 1.87E-02 | Novel gene |
| *GRB2* | 17 | 73294157 | 73421790 | 350 | 2.08 | 1.87E-02 | Novel gene |
| *KLHL21* | 1 | 6630784 | 6682958 | 162 | 2.08 | 1.87E-02 | Novel gene |
| *TMEM242* | 6 | 157690054 | 157765253 | 203 | 2.08 | 1.87E-02 | Novel gene |
| *TUBA1B* | 12 | 49501565 | 49545304 | 112 | 2.08 | 1.87E-02 | Novel gene |
| *SCUBE1* | 22 | 43579229 | 43759394 | 891 | 2.08 | 1.89E-02 | Novel gene |
| *AIF1L* | 9 | 133951863 | 134018539 | 269 | 2.07 | 1.90E-02 | Novel gene |
| *RAD9B* | 12 | 110920005 | 110993952 | 241 | 2.07 | 1.91E-02 | Novel gene |
| *SETD3* | 14 | 99844083 | 99967228 | 197 | 2.07 | 1.91E-02 | Novel gene |
| *CARD10* | 22 | 37866400 | 37935543 | 252 | 2.07 | 1.91E-02 | Novel gene |
| *GCSH* | 16 | 81095552 | 81149980 | 413 | 2.07 | 1.91E-02 | Novel gene |
| *LMBR1L* | 12 | 49470334 | 49524683 | 124 | 2.07 | 1.91E-02 | Novel gene |
| *SIGLEC5* | 19 | 52094756 | 52153727 | 255 | 2.07 | 1.92E-02 | Novel gene |
| *CMKLR1* | 12 | 108661821 | 108753094 | 336 | 2.07 | 1.92E-02 | Novel gene |
| *TNNI2* | 11 | 1840233 | 1882910 | 154 | 2.07 | 1.92E-02 | Novel gene |
| *IHH* | 2 | 219899142 | 219945238 | 159 | 2.07 | 1.93E-02 | Novel gene |
| *THEM5* | 1 | 151798220 | 151846173 | 164 | 2.07 | 1.93E-02 | Novel gene |
| *CRHR1* | 17 | 43677710 | 43933194 | 1243 | 2.07 | 1.94E-02 | Reported gene on lung-related and respiratory-related diseases |
| *MICA* | 6 | 31347561 | 31403090 | 908 | 2.06 | 1.95E-02 | Reported gene on lung-related and respiratory-related diseases |
| *SSUH2* | 3 | 8641086 | 8806721 | 675 | 2.06 | 1.96E-02 | Novel gene |
| *HMX3* | 10 | 124875567 | 124917247 | 117 | 2.06 | 1.97E-02 | Novel gene |
| *ORC5* | 7 | 103746788 | 103868495 | 697 | 2.06 | 1.97E-02 | Novel gene |
| *MRPL9* | 1 | 151712119 | 151756040 | 128 | 2.06 | 1.98E-02 | Novel gene |
| *HSPA14* | 10 | 14860159 | 14933740 | 176 | 2.06 | 1.98E-02 | Novel gene |
| *TNFRSF1B* | 1 | 12207044 | 12289279 | 248 | 2.06 | 1.98E-02 | Reported gene on tuberculosis |
| *AKAP1* | 17 | 55142553 | 55218710 | 335 | 2.05 | 2.00E-02 | Novel gene |
| *TRIQK* | 8 | 93875758 | 93998372 | 295 | 2.05 | 2.00E-02 | Novel gene |
| *LAMP1* | 13 | 113931436 | 113997746 | 112 | 2.05 | 2.00E-02 | Novel gene |
| *LRRC38* | 1 | 13781445 | 13860242 | 360 | 2.05 | 2.00E-02 | Novel gene |
| *SMG6* | 17 | 1943133 | 2227069 | 988 | 2.05 | 2.01E-02 | Reported gene on lung-related diseases |
| *NPHP4* | 1 | 5902870 | 6072618 | 813 | 2.05 | 2.01E-02 | Novel gene |
| *MCU* | 10 | 74431889 | 74667452 | 491 | 2.05 | 2.02E-02 | Novel gene |
| *DENR* | 12 | 123217371 | 123275953 | 180 | 2.05 | 2.02E-02 | Novel gene |
| *ASGR2* | 17 | 6984641 | 7038292 | 294 | 2.05 | 2.03E-02 | Novel gene |
| *RPS21* | 20 | 60942121 | 60983576 | 262 | 2.05 | 2.03E-02 | Novel gene |
| *SFMBT2* | 10 | 7180586 | 7473448 | 1269 | 2.05 | 2.04E-02 | Novel gene |
| *HECW2* | 2 | 197043971 | 197478416 | 1580 | 2.04 | 2.05E-02 | Novel gene |
| *TRIM4* | 7 | 99468030 | 99537223 | 242 | 2.04 | 2.05E-02 | Novel gene |
| *TAS2R1* | 5 | 9609109 | 9650463 | 121 | 2.04 | 2.06E-02 | Novel gene |
| *RPLP1* | 15 | 69725159 | 69767884 | 110 | 2.04 | 2.07E-02 | Novel gene |
| *TMEM200A* | 6 | 130667426 | 130784569 | 470 | 2.04 | 2.07E-02 | Novel gene |
| *PTCHD3* | 10 | 27667117 | 27723297 | 366 | 2.04 | 2.07E-02 | Reported gene on respiratory-related diseases |
| *PROZ* | 13 | 113792968 | 113846698 | 161 | 2.04 | 2.09E-02 | Novel gene |
| *ARL17B* | 17 | 44331550 | 44459416 | 38 | 2.03 | 2.10E-02 | Novel gene |
| *KRT82* | 12 | 52767735 | 52820176 | 329 | 2.03 | 2.10E-02 | Novel gene |
| *MYO7A* | 11 | 76819302 | 76946286 | 552 | 2.03 | 2.11E-02 | Novel gene |
| *NANOS1* | 10 | 120769228 | 120813854 | 108 | 2.03 | 2.11E-02 | Novel gene |
| *PRPF40B* | 12 | 49997197 | 50058452 | 143 | 2.03 | 2.12E-02 | Novel gene |
| *CDC27* | 17 | 45175310 | 45286665 | 288 | 2.03 | 2.12E-02 | Novel gene |
| *UCHL1* | 4 | 41238898 | 41290446 | 208 | 2.03 | 2.13E-02 | Novel gene |
| *MYADML2* | 17 | 79877521 | 79925109 | 113 | 2.03 | 2.13E-02 | Novel gene |
| *SLC5A4* | 22 | 32594463 | 32671318 | 401 | 2.03 | 2.13E-02 | Novel gene |
| *NAGS* | 17 | 42062032 | 42106436 | 140 | 2.02 | 2.15E-02 | Novel gene |
| *BCAR3* | 1 | 94007343 | 94332706 | 1209 | 2.02 | 2.15E-02 | Novel gene |
| *RAD51D* | 17 | 33406811 | 33466888 | 138 | 2.02 | 2.15E-02 | Novel gene |
| *FAM174B* | 15 | 93140678 | 93219031 | 335 | 2.02 | 2.16E-02 | Novel gene |
| *GRB14* | 2 | 165329323 | 165498360 | 418 | 2.02 | 2.16E-02 | Novel gene |
| *CCDC47* | 17 | 61802610 | 61871088 | 202 | 2.02 | 2.18E-02 | Novel gene |
| *ANKRD23* | 2 | 97468599 | 97529758 | 119 | 2.02 | 2.18E-02 | Novel gene |
| *FANCD2OS* | 3 | 10103004 | 10169975 | 232 | 2.02 | 2.18E-02 | Novel gene |
| *C5orf60* | 5 | 179048545 | 179092083 | 170 | 2.02 | 2.18E-02 | Novel gene |
| *SLC25A46* | 5 | 110053837 | 110120857 | 216 | 2.01 | 2.20E-02 | Reported gene on respiratory-related diseases |
| *PEG3* | 19 | 57265730 | 57372121 | 215 | 2.01 | 2.20E-02 | Novel gene |
| *ZIM2* | 19 | 57265915 | 57372097 | 215 | 2.01 | 2.20E-02 | Novel gene |
| *LOC101929163* | 6 | 32351234 | 32393967 | 772 | 2.01 | 2.21E-02 | Novel gene |
| *NR5A1* | 9 | 127223515 | 127289769 | 138 | 2.01 | 2.22E-02 | Novel gene |
| *CCDC107* | 9 | 35638279 | 35681500 | 187 | 2.01 | 2.22E-02 | Novel gene |
| *DLL3* | 19 | 39969557 | 40019121 | 196 | 2.01 | 2.22E-02 | Novel gene |
| *CABLES1* | 18 | 20694528 | 20860434 | 400 | 2.01 | 2.23E-02 | Reported gene on tuberculosis, lung-related diseases |
| *APOBEC3C* | 22 | 39390265 | 39434843 | 147 | 2.01 | 2.23E-02 | Reported gene on respiratory-related diseases |
| *MACROD2* | 20 | 13956146 | 16053842 | 7869 | 2.01 | 2.23E-02 | Novel gene |
| *MAML3* | 4 | 140617545 | 141095233 | 1372 | 2.01 | 2.24E-02 | Novel gene |
| *ULK2* | 17 | 19654143 | 19791239 | 329 | 2.00 | 2.25E-02 | Novel gene |
| *LGALS1* | 22 | 38051613 | 38095809 | 180 | 2.00 | 2.25E-02 | Novel gene |
| *EIF3A* | 10 | 120774541 | 120860959 | 215 | 2.00 | 2.27E-02 | Novel gene |
| *BPTF* | 17 | 65801644 | 66000494 | 587 | 2.00 | 2.27E-02 | Reported gene on lung-related diseases |
| *GNAT1* | 3 | 50209043 | 50255129 | 96 | 2.00 | 2.28E-02 | Novel gene |
| *LOC100287294* | 7 | 56971848 | 57032060 | 16 | 2.00 | 2.29E-02 | Novel gene |
| *MS4A3* | 11 | 59804101 | 59858588 | 195 | 2.00 | 2.29E-02 | Novel gene |
| *INS* | 11 | 2161009 | 2202439 | 189 | 2.00 | 2.29E-02 | Novel gene |
| *OC90* | 8 | 133016467 | 133091627 | 389 | 2.00 | 2.30E-02 | Novel gene |
| *INPP5E* | 9 | 139303067 | 139354305 | 235 | 2.00 | 2.30E-02 | Novel gene |
| *LOC101929355* | 13 | 114941877 | 115008549 | 284 | 1.99 | 2.31E-02 | Novel gene |
| *ALPK1* | 4 | 113198480 | 113383774 | 669 | 1.99 | 2.31E-02 | Novel gene |
| *TIGD5* | 8 | 144660074 | 144702485 | 225 | 1.99 | 2.31E-02 | Novel gene |
| *PTMA* | 2 | 232553235 | 232598251 | 171 | 1.99 | 2.32E-02 | Novel gene |
| *FMNL3* | 12 | 50011724 | 50121197 | 315 | 1.99 | 2.32E-02 | Novel gene |
| *SPACA3* | 17 | 31298882 | 31344895 | 206 | 1.99 | 2.32E-02 | Novel gene |
| *MOV10L1* | 22 | 50508435 | 50620119 | 674 | 1.99 | 2.35E-02 | Novel gene |
| *MCUR1* | 6 | 13766781 | 13834794 | 193 | 1.99 | 2.35E-02 | Novel gene |
| *C1QL3* | 10 | 16535742 | 16584004 | 189 | 1.99 | 2.35E-02 | Novel gene |
| *DGUOK* | 2 | 74133953 | 74206088 | 244 | 1.99 | 2.35E-02 | Novel gene |
| *MRPL16* | 11 | 59553608 | 59598345 | 147 | 1.99 | 2.35E-02 | Novel gene |
| *EEF1D* | 8 | 144641867 | 144699845 | 317 | 1.99 | 2.35E-02 | Novel gene |
| *UCN3* | 10 | 5386976 | 5436169 | 225 | 1.99 | 2.36E-02 | Novel gene |
| *ZNF532* | 18 | 56509832 | 56673709 | 612 | 1.99 | 2.36E-02 | Novel gene |
| *NXPE2* | 11 | 114529200 | 114599357 | 281 | 1.98 | 2.36E-02 | Novel gene |
| *GUCY2D* | 17 | 7885988 | 7943658 | 189 | 1.98 | 2.36E-02 | Novel gene |
| *BCAS2* | 1 | 115090178 | 115144265 | 174 | 1.98 | 2.36E-02 | Novel gene |
| *EFNB3* | 17 | 7588520 | 7634693 | 168 | 1.98 | 2.37E-02 | Novel gene |
| *GH1* | 17 | 61974560 | 62016206 | 134 | 1.98 | 2.38E-02 | Novel gene |
| *TLN1* | 9 | 35677334 | 35752392 | 206 | 1.98 | 2.38E-02 | Reported gene on respiratory-related diseases |
| *PARD6B* | 20 | 49327923 | 49393333 | 207 | 1.98 | 2.40E-02 | Novel gene |
| *GJA4* | 1 | 35238559 | 35281351 | 202 | 1.98 | 2.40E-02 | Novel gene |
| *C6orf163* | 6 | 88034571 | 88095381 | 307 | 1.98 | 2.41E-02 | Novel gene |
| *TAL2* | 9 | 108398562 | 108445393 | 159 | 1.98 | 2.41E-02 | Novel gene |
| *TRPM3* | 9 | 73123979 | 74081782 | 3816 | 1.97 | 2.43E-02 | Novel gene |
| *ZNF501* | 3 | 44751098 | 44798575 | 158 | 1.97 | 2.44E-02 | Novel gene |
| *SMARCD2* | 17 | 61889441 | 61940699 | 180 | 1.97 | 2.44E-02 | Reported gene on lung-related diseases |
| *AQP7* | 9 | 33364948 | 33422643 | 168 | 1.97 | 2.45E-02 | Reported gene on lung-related diseases |
| *ZZZ3* | 1 | 78008160 | 78169119 | 432 | 1.97 | 2.46E-02 | Reported gene on lung-related diseases |
| *ATP13A5* | 3 | 192972831 | 193116514 | 624 | 1.97 | 2.47E-02 | Novel gene |
| *ARHGAP31* | 3 | 118993220 | 119158323 | 739 | 1.97 | 2.47E-02 | Reported gene on lung-related diseases |
| *PLEKHG5* | 1 | 6506152 | 6600121 | 329 | 1.96 | 2.48E-02 | Novel gene |
| *LOC100130880* | 7 | 137618094 | 137662712 | 118 | 1.96 | 2.48E-02 | Novel gene |
| *ZNF426* | 19 | 9618667 | 9669638 | 257 | 1.96 | 2.49E-02 | Novel gene |
| *NLE1* | 17 | 33438368 | 33489322 | 131 | 1.96 | 2.50E-02 | Novel gene |
| *ERO1L* | 14 | 53088605 | 53182419 | 229 | 1.96 | 2.51E-02 | Novel gene |
| *SPPL2C* | 17 | 43902256 | 43944438 | 292 | 1.96 | 2.51E-02 | Reported gene on respiratory-related diseases |
| *LVRN* | 5 | 115278151 | 115383303 | 665 | 1.96 | 2.52E-02 | Novel gene |
| *ITM2C* | 2 | 231709546 | 231763963 | 245 | 1.96 | 2.52E-02 | Novel gene |
| *CYP7B1* | 8 | 65488529 | 65731348 | 639 | 1.95 | 2.53E-02 | Novel gene |
| *CSRP2BP* | 20 | 18102868 | 18189031 | 336 | 1.95 | 2.54E-02 | Novel gene |
| *RPS16* | 19 | 39903847 | 39946660 | 171 | 1.95 | 2.55E-02 | Novel gene |
| *MT1X* | 16 | 56696382 | 56738108 | 270 | 1.95 | 2.56E-02 | Novel gene |
| *LOC101929519* | 9 | 96413303 | 96455032 | 246 | 1.95 | 2.56E-02 | Novel gene |
| *KIAA1143* | 3 | 44770236 | 44823173 | 119 | 1.95 | 2.56E-02 | Novel gene |
| *PLEKHG4B* | 5 | 120373 | 210087 | 428 | 1.95 | 2.57E-02 | Reported gene on respiratory-related diseases |
| *FTSJ3* | 17 | 61876793 | 61925031 | 149 | 1.95 | 2.57E-02 | Novel gene |
| *DTX3* | 12 | 57978110 | 58023585 | 108 | 1.95 | 2.57E-02 | Novel gene |
| *MICU1* | 10 | 74107084 | 74405949 | 918 | 1.95 | 2.58E-02 | Novel gene |
| *KCNK18* | 10 | 118937000 | 118989810 | 161 | 1.95 | 2.58E-02 | Novel gene |
| *SLURP1* | 8 | 143802362 | 143843829 | 136 | 1.95 | 2.59E-02 | Novel gene |
| *FAM98B* | 15 | 38726328 | 38797063 | 299 | 1.94 | 2.60E-02 | Novel gene |
| *ZNF121* | 19 | 9656292 | 9715209 | 253 | 1.94 | 2.60E-02 | Novel gene |
| *ARHGEF25* | 12 | 57983963 | 58031028 | 113 | 1.94 | 2.61E-02 | Novel gene |
| *CDK10* | 16 | 89733076 | 89782772 | 258 | 1.94 | 2.62E-02 | Novel gene |
| *XKR5* | 8 | 6646038 | 6713166 | 336 | 1.94 | 2.63E-02 | Novel gene |
| *IP6K2* | 3 | 48705436 | 48774711 | 158 | 1.94 | 2.64E-02 | Novel gene |
| *SUV39H2* | 10 | 14900782 | 14966314 | 160 | 1.94 | 2.64E-02 | Novel gene |
| *RLN2* | 9 | 5279864 | 5324611 | 111 | 1.94 | 2.65E-02 | Novel gene |
| *ASIC2* | 17 | 31320105 | 32503825 | 4628 | 1.93 | 2.66E-02 | Novel gene |
| *PYCRL* | 8 | 144666083 | 144711784 | 201 | 1.93 | 2.66E-02 | Novel gene |
| *CPSF1* | 8 | 145598446 | 145654733 | 135 | 1.93 | 2.67E-02 | Novel gene |
| *SEC16A* | 9 | 139314548 | 139399297 | 375 | 1.93 | 2.67E-02 | Novel gene |
| *TCTN1* | 12 | 111031832 | 111106935 | 204 | 1.93 | 2.69E-02 | Novel gene |
| *FAM171A2* | 17 | 42411101 | 42461235 | 101 | 1.92 | 2.71E-02 | Novel gene |
| *NACC2* | 9 | 138878383 | 139007131 | 450 | 1.92 | 2.71E-02 | Novel gene |
| *RUNX1* | 21 | 36140098 | 36441595 | 776 | 1.92 | 2.73E-02 | Reported gene on respiratory-related diseases |
| *ZNF354A* | 5 | 178118522 | 178177703 | 212 | 1.92 | 2.74E-02 | Novel gene |
| *ZFP36L2* | 2 | 43429541 | 43473745 | 126 | 1.92 | 2.74E-02 | Novel gene |
| *C1orf195* | 1 | 15470692 | 15518120 | 268 | 1.92 | 2.74E-02 | Novel gene |
| *SYNE3* | 14 | 95863831 | 96003000 | 745 | 1.92 | 2.74E-02 | Novel gene |
| *COL11A1* | 1 | 103322023 | 103594052 | 1313 | 1.92 | 2.75E-02 | Novel gene |
| *LYNX1* | 8 | 143825756 | 143879640 | 252 | 1.92 | 2.75E-02 | Novel gene |
| *NDUFA11* | 19 | 5871287 | 5924024 | 273 | 1.92 | 2.75E-02 | Novel gene |
| *VPS37D* | 7 | 73062174 | 73106440 | 181 | 1.92 | 2.76E-02 | Novel gene |
| *CLEC10A* | 17 | 6957856 | 7003626 | 239 | 1.92 | 2.76E-02 | Novel gene |
| *NDUFB8* | 10 | 102263497 | 102309640 | 157 | 1.92 | 2.76E-02 | Novel gene |
| *CNNM3* | 2 | 97460950 | 97521121 | 118 | 1.92 | 2.77E-02 | Novel gene |
| *HLA-DQA1* | 6 | 32585169 | 32632152 | 633 | 1.91 | 2.79E-02 | Reported gene on tuberculosis, lung-related and respiratory-related diseases |
| *PET117* | 20 | 18098499 | 18189016 | 346 | 1.91 | 2.79E-02 | Novel gene |
| *NDUFA5* | 7 | 123161083 | 123217958 | 168 | 1.91 | 2.79E-02 | Novel gene |
| *GALC* | 14 | 88284164 | 88480009 | 1108 | 1.91 | 2.82E-02 | Novel gene |
| *CLEC16A* | 16 | 11018345 | 11296046 | 1173 | 1.91 | 2.82E-02 | Reported gene on lung-related and respiratory-related diseases |
| *RPAP2* | 1 | 92744522 | 92873732 | 445 | 1.91 | 2.82E-02 | Novel gene |
| *GOLPH3* | 5 | 32104817 | 32194425 | 278 | 1.91 | 2.83E-02 | Novel gene |
| *FOXJ1* | 17 | 74112414 | 74157380 | 142 | 1.91 | 2.83E-02 | Novel gene |
| *DDX39A* | 19 | 14499610 | 14550195 | 103 | 1.91 | 2.83E-02 | Novel gene |
| *MYO18A* | 17 | 27380528 | 27527432 | 416 | 1.91 | 2.84E-02 | Novel gene |
| *OR10A3* | 11 | 7940123 | 7981067 | 200 | 1.90 | 2.85E-02 | Novel gene |
| *CCNB1IP1* | 14 | 20759527 | 20821471 | 245 | 1.90 | 2.85E-02 | Novel gene |
| *DDX42* | 17 | 61831549 | 61916677 | 255 | 1.90 | 2.86E-02 | Novel gene |
| *UQCRH* | 1 | 46749324 | 46802448 | 89 | 1.90 | 2.86E-02 | Novel gene |
| *ADCY4* | 14 | 24767555 | 24824277 | 210 | 1.90 | 2.86E-02 | Novel gene |
| *CNTROB* | 17 | 7815442 | 7873237 | 213 | 1.90 | 2.86E-02 | Novel gene |
| *USP13* | 3 | 179350933 | 179527189 | 616 | 1.90 | 2.87E-02 | Novel gene |
| *SLIT3* | 5 | 168068738 | 168748133 | 2763 | 1.90 | 2.88E-02 | Novel gene |
| *PNPLA8* | 7 | 108090866 | 108188605 | 339 | 1.90 | 2.88E-02 | Novel gene |
| *TAS1R1* | 1 | 6594987 | 6659817 | 274 | 1.90 | 2.88E-02 | Novel gene |
| *NOP14* | 4 | 2919663 | 2985233 | 335 | 1.90 | 2.88E-02 | Reported gene on lung-related diseases |
| *OR2L2* | 1 | 248181474 | 248222607 | 74 | 1.90 | 2.89E-02 | Novel gene |
| *VAX2* | 2 | 71107720 | 71180576 | 279 | 1.90 | 2.90E-02 | Novel gene |
| *ZNF888* | 19 | 53389168 | 53446930 | 246 | 1.89 | 2.91E-02 | Novel gene |
| *LIG3* | 17 | 33287517 | 33352088 | 132 | 1.89 | 2.91E-02 | Novel gene |
| *SENP6* | 6 | 76291596 | 76447997 | 573 | 1.89 | 2.91E-02 | Novel gene |
| *PHLDB3* | 19 | 43959255 | 44029073 | 237 | 1.89 | 2.91E-02 | Novel gene |
| *ADD1* | 4 | 2825454 | 2951803 | 623 | 1.89 | 2.92E-02 | Novel gene |
| *TRIM29* | 11 | 119961994 | 120076237 | 620 | 1.89 | 2.92E-02 | Novel gene |
| *DENND2C* | 1 | 115107155 | 115232732 | 418 | 1.89 | 2.92E-02 | Novel gene |
| *BRK1* | 3 | 10137333 | 10188874 | 224 | 1.89 | 2.92E-02 | Novel gene |
| *DHH* | 12 | 49463204 | 49508602 | 95 | 1.89 | 2.92E-02 | Novel gene |
| *LOC100130357* | 6 | 13259527 | 13317314 | 317 | 1.89 | 2.93E-02 | Novel gene |
| *PSMC5* | 17 | 61884770 | 61929387 | 142 | 1.89 | 2.93E-02 | Novel gene |
| *SAMD11* | 1 | 839993 | 899961 | 286 | 1.89 | 2.94E-02 | Novel gene |
| *PNPT1* | 2 | 55841198 | 55941045 | 483 | 1.89 | 2.94E-02 | Novel gene |
| *KDM4B* | 19 | 4949123 | 5173609 | 904 | 1.89 | 2.94E-02 | Novel gene |
| *ANKRD39* | 2 | 97493722 | 97543756 | 88 | 1.89 | 2.95E-02 | Novel gene |
| *CASP4* | 11 | 104793594 | 104859325 | 238 | 1.89 | 2.95E-02 | Novel gene |
| *EID2B* | 19 | 40001630 | 40043494 | 181 | 1.89 | 2.96E-02 | Novel gene |
| *ZNF287* | 17 | 16433626 | 16492520 | 142 | 1.89 | 2.96E-02 | Novel gene |
| *OR10A6* | 11 | 7929265 | 7970209 | 207 | 1.89 | 2.96E-02 | Novel gene |
| *FNDC8* | 17 | 33428598 | 33477751 | 118 | 1.89 | 2.96E-02 | Novel gene |
| *TFAM* | 10 | 60124903 | 60178990 | 188 | 1.89 | 2.97E-02 | Novel gene |
| *WRAP53* | 17 | 7569389 | 7626820 | 223 | 1.88 | 2.97E-02 | Novel gene |
| *TAF1A* | 1 | 222710990 | 222783438 | 256 | 1.88 | 2.98E-02 | Novel gene |
| *RIOK3* | 18 | 21012787 | 21083104 | 222 | 1.88 | 2.98E-02 | Reported gene on lung-related diseases |
| *OAZ2* | 15 | 64959773 | 65015462 | 149 | 1.88 | 2.99E-02 | Novel gene |
| *ZNF775* | 7 | 150056406 | 150115719 | 181 | 1.88 | 2.99E-02 | Novel gene |
| *MYLPF* | 16 | 30366123 | 30409310 | 86 | 1.88 | 2.99E-02 | Novel gene |
| *SHROOM3* | 4 | 77336253 | 77724406 | 1319 | 1.88 | 3.00E-02 | Reported gene on lung-related diseases |
| *LDLRAD4* | 18 | 13198729 | 13672753 | 1637 | 1.88 | 3.00E-02 | Novel gene |
| *ATP6V1E2* | 2 | 46718986 | 46789551 | 287 | 1.88 | 3.01E-02 | Novel gene |
| *CKAP2L* | 2 | 113475444 | 113542254 | 193 | 1.88 | 3.01E-02 | Novel gene |
| *SRSF8* | 11 | 94780041 | 94824387 | 171 | 1.88 | 3.01E-02 | Novel gene |
| *AKR7A3* | 1 | 19589057 | 19635280 | 194 | 1.88 | 3.02E-02 | Novel gene |
| *TMEM74* | 8 | 109771165 | 109819770 | 136 | 1.88 | 3.02E-02 | Novel gene |
| *SIMC1* | 5 | 175645370 | 175792992 | 321 | 1.88 | 3.02E-02 | Novel gene |
| *DLL1* | 6 | 170571288 | 170619815 | 189 | 1.88 | 3.02E-02 | Novel gene |
| *AMPD2* | 1 | 110142435 | 110194677 | 146 | 1.88 | 3.02E-02 | Novel gene |
| *THNSL1* | 10 | 25285508 | 25335593 | 207 | 1.88 | 3.03E-02 | Novel gene |
| *PTCHD2* | 1 | 11519295 | 11617640 | 317 | 1.87 | 3.05E-02 | Novel gene |
| *KRT26* | 17 | 38902490 | 38948411 | 172 | 1.87 | 3.06E-02 | Novel gene |
| *FANCD2* | 3 | 10048113 | 10163614 | 346 | 1.87 | 3.06E-02 | Novel gene |
| *PRKAR2A* | 3 | 48764017 | 48905270 | 227 | 1.87 | 3.08E-02 | Novel gene |
| *RORC* | 1 | 151758547 | 151824348 | 201 | 1.87 | 3.08E-02 | Reported gene on respiratory-related diseases |
| *C3orf33* | 3 | 155460401 | 155544076 | 350 | 1.87 | 3.08E-02 | Novel gene |
| *ING2* | 4 | 184406220 | 184453422 | 179 | 1.87 | 3.09E-02 | Novel gene |
| *SDPR* | 2 | 192679031 | 192732006 | 128 | 1.87 | 3.10E-02 | Novel gene |
| *ZNF445* | 3 | 44461262 | 44539162 | 182 | 1.87 | 3.10E-02 | Novel gene |
| *CST5* | 20 | 23836572 | 23880380 | 222 | 1.86 | 3.12E-02 | Novel gene |
| *TM4SF18* | 3 | 149016285 | 149071548 | 181 | 1.86 | 3.13E-02 | Novel gene |
| *PITX2* | 4 | 111518579 | 111583279 | 118 | 1.86 | 3.14E-02 | Reported gene on lung-related diseases |
| *NEURL3* | 2 | 97143383 | 97193846 | 66 | 1.86 | 3.14E-02 | Novel gene |
| *BTC* | 4 | 75651448 | 75739882 | 329 | 1.86 | 3.17E-02 | Reported gene on lung-related and respiratory-related diseases |
| *NUP50* | 22 | 45539726 | 45603892 | 303 | 1.86 | 3.17E-02 | Novel gene |
| *TMBIM6* | 12 | 50115293 | 50178717 | 257 | 1.86 | 3.18E-02 | Novel gene |
| *GOLGB1* | 3 | 121362046 | 121488662 | 302 | 1.86 | 3.18E-02 | Novel gene |
| *TBXA2R* | 19 | 3574504 | 3626831 | 203 | 1.86 | 3.18E-02 | Novel gene |
| *RRP8* | 11 | 6601144 | 6644884 | 157 | 1.85 | 3.21E-02 | Novel gene |
| *MLC1* | 22 | 50477820 | 50544358 | 382 | 1.85 | 3.22E-02 | Novel gene |
| *DUSP13* | 10 | 76834190 | 76888976 | 197 | 1.85 | 3.24E-02 | Novel gene |
| *KIF25* | 6 | 168398553 | 168465769 | 378 | 1.84 | 3.26E-02 | Novel gene |
| *ERGIC2* | 12 | 29473579 | 29554143 | 254 | 1.84 | 3.27E-02 | Novel gene |
| *RBM20* | 10 | 112384155 | 112619227 | 942 | 1.84 | 3.28E-02 | Reported gene on lung-related diseases |
| *SIT1* | 9 | 35629295 | 35670947 | 187 | 1.84 | 3.29E-02 | Novel gene |
| *CTAGE6* | 7 | 143432182 | 143474843 | 13 | 1.84 | 3.30E-02 | Novel gene |
| *GNAI3* | 1 | 110071186 | 110158465 | 250 | 1.84 | 3.30E-02 | Novel gene |
| *ZNF248* | 10 | 38045454 | 38167012 | 600 | 1.84 | 3.32E-02 | Novel gene |
| *ANAPC1* | 2 | 112505214 | 112661765 | 305 | 1.84 | 3.32E-02 | Reported gene on lung-related and respiratory-related diseases |
| *GUCY1A3* | 4 | 156567862 | 156678214 | 436 | 1.84 | 3.32E-02 | Novel gene |
| *RASL11A* | 13 | 27824464 | 27867828 | 168 | 1.83 | 3.34E-02 | Novel gene |
| *CACNG3* | 16 | 24246874 | 24393737 | 599 | 1.83 | 3.34E-02 | Reported gene on respiratory-related diseases |
| *DOPEY2* | 21 | 37509080 | 37686572 | 858 | 1.83 | 3.36E-02 | Novel gene |
| *ACSF3* | 16 | 89140217 | 89242171 | 705 | 1.83 | 3.37E-02 | Novel gene |
| *MT1G* | 16 | 56680653 | 56721977 | 297 | 1.83 | 3.38E-02 | Novel gene |
| *NRCAM* | 7 | 107768071 | 108116841 | 1461 | 1.83 | 3.39E-02 | Novel gene |
| *GLG1* | 16 | 74461325 | 74661042 | 882 | 1.83 | 3.39E-02 | Novel gene |
| *SLC18A2* | 10 | 118980584 | 119058941 | 210 | 1.83 | 3.39E-02 | Novel gene |
| *ZNF48* | 16 | 30369633 | 30431429 | 107 | 1.83 | 3.40E-02 | Novel gene |
| *DKKL1* | 19 | 49846466 | 49898373 | 226 | 1.83 | 3.40E-02 | Novel gene |
| *VWA2* | 10 | 115979013 | 116074259 | 310 | 1.82 | 3.40E-02 | Novel gene |
| *MNX1* | 7 | 156777547 | 156823347 | 198 | 1.82 | 3.40E-02 | Novel gene |
| *OR51B4* | 11 | 5302244 | 5343176 | 217 | 1.82 | 3.40E-02 | Novel gene |
| *TTR* | 18 | 29151730 | 29198987 | 150 | 1.82 | 3.41E-02 | Novel gene |
| *IGSF22* | 11 | 18705852 | 18767777 | 234 | 1.82 | 3.41E-02 | Novel gene |
| *TESPA1* | 12 | 55322087 | 55398965 | 473 | 1.82 | 3.43E-02 | Novel gene |
| *MGAT3* | 22 | 39823235 | 39908199 | 195 | 1.82 | 3.45E-02 | Novel gene |
| *P2RX7* | 12 | 121550622 | 121644439 | 498 | 1.82 | 3.45E-02 | Reported gene on respiratory-related diseases |
| *CNNM4* | 2 | 97406639 | 97497628 | 187 | 1.82 | 3.47E-02 | Novel gene |
| *CABLES2* | 20 | 60943686 | 61002339 | 330 | 1.82 | 3.47E-02 | Novel gene |
| *CD44* | 11 | 35140417 | 35273949 | 586 | 1.82 | 3.47E-02 | Novel gene |
| *SIAH3* | 13 | 46334405 | 46445846 | 468 | 1.81 | 3.48E-02 | Novel gene |
| *VSTM5* | 11 | 93533735 | 93603668 | 393 | 1.81 | 3.49E-02 | Novel gene |
| *NOP58* | 2 | 203110515 | 203188384 | 213 | 1.81 | 3.49E-02 | Novel gene |
| *KCNB1* | 20 | 47968505 | 48119181 | 607 | 1.81 | 3.50E-02 | Novel gene |
| *TRDMT1* | 10 | 17159828 | 17264093 | 403 | 1.81 | 3.51E-02 | Novel gene |
| *ABCC3* | 17 | 48692205 | 48789063 | 297 | 1.81 | 3.51E-02 | Novel gene |
| *NDUFC1* | 4 | 140191071 | 140243705 | 145 | 1.81 | 3.51E-02 | Reported gene on lung-related diseases |
| *ZNF484* | 9 | 95587313 | 95660320 | 268 | 1.81 | 3.51E-02 | Novel gene |
| *PTPRF* | 1 | 43971708 | 44109343 | 529 | 1.81 | 3.52E-02 | Novel gene |
| *GPR150* | 5 | 94935980 | 94977284 | 113 | 1.81 | 3.52E-02 | Novel gene |
| *LOC101929530* | 3 | 11892358 | 11963081 | 370 | 1.81 | 3.53E-02 | Novel gene |
| *FCHO1* | 19 | 17838527 | 17919377 | 328 | 1.81 | 3.53E-02 | Novel gene |
| *FAM186B* | 12 | 49956658 | 50019433 | 169 | 1.81 | 3.55E-02 | Novel gene |
| *ACTR2* | 2 | 65434829 | 65518387 | 312 | 1.81 | 3.55E-02 | Reported gene on lung-related diseases |
| *HHIP* | 4 | 145547148 | 145682542 | 344 | 1.80 | 3.56E-02 | Reported gene on lung-related and respiratory-related diseases |
| *KSR2* | 12 | 117870817 | 118426399 | 1993 | 1.80 | 3.56E-02 | Reported gene on lung-related diseases |
| *ZNF397* | 18 | 32800994 | 32858397 | 172 | 1.80 | 3.56E-02 | Novel gene |
| *TP53* | 17 | 7551720 | 7610868 | 212 | 1.80 | 3.58E-02 | Novel gene |
| *GSG1L* | 16 | 27778850 | 28094830 | 1174 | 1.80 | 3.59E-02 | Novel gene |
| *CFAP70* | 10 | 74993516 | 75139425 | 507 | 1.80 | 3.60E-02 | Novel gene |
| *IQCK* | 16 | 19707778 | 19889087 | 658 | 1.80 | 3.60E-02 | Novel gene |
| *OPTN* | 10 | 13121425 | 13200291 | 242 | 1.79 | 3.64E-02 | Novel gene |
| *CLDN18* | 3 | 137697658 | 137772494 | 188 | 1.79 | 3.64E-02 | Reported gene on lung-related diseases |
| *PFKP* | 10 | 3089667 | 3198997 | 801 | 1.79 | 3.64E-02 | Novel gene |
| *TMPRSS11E* | 4 | 69293167 | 69383322 | 312 | 1.79 | 3.65E-02 | Novel gene |
| *NFIL3* | 9 | 94151327 | 94206908 | 175 | 1.79 | 3.65E-02 | Novel gene |
| *SSC4D* | 7 | 75998646 | 76059012 | 299 | 1.79 | 3.65E-02 | Novel gene |
| *ARRDC4* | 15 | 98483933 | 98537068 | 274 | 1.79 | 3.66E-02 | Novel gene |
| *ZNF778* | 16 | 89264111 | 89322402 | 409 | 1.79 | 3.66E-02 | Novel gene |
| *42979* | 16 | 30369454 | 30414171 | 87 | 1.79 | 3.66E-02 | Novel gene |
| *GALNT6* | 12 | 51725654 | 51805739 | 223 | 1.79 | 3.67E-02 | Novel gene |
| *SUPT5H* | 19 | 39916186 | 39987310 | 295 | 1.79 | 3.67E-02 | Novel gene |
| *AFAP1L2* | 10 | 116021248 | 116184515 | 514 | 1.79 | 3.67E-02 | Novel gene |
| *SPOPL* | 2 | 139239350 | 139350805 | 235 | 1.79 | 3.68E-02 | Novel gene |
| *GPATCH2L* | 14 | 76598255 | 76691239 | 408 | 1.79 | 3.68E-02 | Novel gene |
| *SCN4B* | 11 | 117984092 | 118043630 | 293 | 1.79 | 3.69E-02 | Novel gene |
| *RRP12* | 10 | 99096458 | 99181127 | 388 | 1.79 | 3.69E-02 | Novel gene |
| *VTA1* | 6 | 142448372 | 142562085 | 301 | 1.79 | 3.70E-02 | Reported gene on lung-related and respiratory-related diseases |
| *PCID2* | 13 | 113811853 | 113883335 | 279 | 1.79 | 3.70E-02 | Novel gene |
| *POU4F2* | 4 | 147540045 | 147583623 | 163 | 1.79 | 3.70E-02 | Novel gene |
| *SEMA3F* | 3 | 50172562 | 50246508 | 181 | 1.79 | 3.70E-02 | Novel gene |
| *ETHE1* | 19 | 43990871 | 44051408 | 203 | 1.79 | 3.71E-02 | Novel gene |
| *PM20D2* | 6 | 89830047 | 89895288 | 269 | 1.79 | 3.71E-02 | Novel gene |
| *GLMN* | 1 | 92691955 | 92784566 | 269 | 1.79 | 3.71E-02 | Novel gene |
| *DPEP1* | 16 | 89659716 | 89727216 | 381 | 1.78 | 3.72E-02 | Novel gene |
| *HLX* | 1 | 221032743 | 221078401 | 167 | 1.78 | 3.75E-02 | Reported gene on lung-related diseases |
| *CPA5* | 7 | 129964630 | 130028571 | 277 | 1.78 | 3.75E-02 | Novel gene |
| *KDM6B* | 17 | 7717521 | 7778118 | 169 | 1.78 | 3.75E-02 | Novel gene |
| *TSEN2* | 3 | 12497748 | 12600672 | 455 | 1.78 | 3.76E-02 | Reported gene on lung-related diseases |
| *RCN2* | 15 | 77203962 | 77262601 | 112 | 1.78 | 3.76E-02 | Novel gene |
| *BTNL2* | 6 | 32342513 | 32394900 | 896 | 1.78 | 3.76E-02 | Reported gene on tuberculosis, lung-related and respiratory-related diseases |
| *SIM1* | 6 | 100812891 | 100932805 | 382 | 1.78 | 3.76E-02 | Novel gene |
| *SLC35D2* | 9 | 99055719 | 99165992 | 454 | 1.78 | 3.77E-02 | Novel gene |
| *PANX2* | 22 | 50589160 | 50638724 | 257 | 1.78 | 3.77E-02 | Novel gene |
| *SLC25A48* | 5 | 135150365 | 135244326 | 429 | 1.78 | 3.77E-02 | Reported gene on tuberculosis |
| *ACCS* | 11 | 44067729 | 44125791 | 273 | 1.78 | 3.78E-02 | Novel gene |
| *CLCN2* | 3 | 184043973 | 184099439 | 184 | 1.78 | 3.78E-02 | Novel gene |
| *PXMP4* | 20 | 32270550 | 32328136 | 127 | 1.78 | 3.78E-02 | Novel gene |
| *ZNF609* | 15 | 64771619 | 64998267 | 677 | 1.78 | 3.79E-02 | Novel gene |
| *TRAM2* | 6 | 52342200 | 52461862 | 562 | 1.78 | 3.79E-02 | Novel gene |
| *TRIP4* | 15 | 64660003 | 64767502 | 294 | 1.78 | 3.79E-02 | Novel gene |
| *SHC4* | 15 | 49095934 | 49275641 | 519 | 1.77 | 3.80E-02 | Novel gene |
| *FGF8* | 10 | 103509887 | 103560126 | 86 | 1.77 | 3.81E-02 | Novel gene |
| *TRAM1L1* | 4 | 117984710 | 118026736 | 144 | 1.77 | 3.81E-02 | Novel gene |
| *C3orf67* | 3 | 58700199 | 59055804 | 643 | 1.77 | 3.82E-02 | Novel gene |
| *RAF1* | 3 | 12605100 | 12725700 | 416 | 1.77 | 3.83E-02 | Novel gene |
| *FIGNL2* | 12 | 52191676 | 52262364 | 203 | 1.77 | 3.84E-02 | Novel gene |
| *NPM3* | 10 | 103521082 | 103563170 | 85 | 1.77 | 3.84E-02 | Novel gene |
| *MN1* | 22 | 28124265 | 28217486 | 226 | 1.77 | 3.85E-02 | Reported gene on lung-related and respiratory-related diseases |
| *IL20RA* | 6 | 137301108 | 137386317 | 331 | 1.77 | 3.86E-02 | Novel gene |
| *TNPO1* | 5 | 72092418 | 72230215 | 275 | 1.77 | 3.87E-02 | Reported gene on respiratory-related diseases |
| *CASKIN2* | 17 | 73476341 | 73531664 | 245 | 1.77 | 3.87E-02 | Novel gene |
| *HLA-DRB5* | 6 | 32465151 | 32518006 | 46 | 1.77 | 3.87E-02 | Reported gene on tuberculosis and respiratory-related diseases |
| *KIF1B* | 1 | 10250606 | 10461664 | 829 | 1.76 | 3.88E-02 | Reported gene on lung-related diseases |
| *CCDC122* | 13 | 44390488 | 44473826 | 364 | 1.76 | 3.88E-02 | Novel gene |
| *CATSPER3* | 5 | 134283596 | 134367397 | 231 | 1.76 | 3.91E-02 | Novel gene |
| *SMAD5* | 5 | 135448536 | 135538422 | 230 | 1.76 | 3.91E-02 | Novel gene |
| *PMPCA* | 9 | 139285110 | 139338213 | 265 | 1.76 | 3.92E-02 | Novel gene |
| *ERBB2* | 17 | 37824167 | 37904915 | 193 | 1.76 | 3.92E-02 | Reported gene on respiratory-related diseases |
| *NDN* | 15 | 23910554 | 23952450 | 111 | 1.76 | 3.94E-02 | Novel gene |
| *MYCBPAP* | 17 | 48565745 | 48628863 | 272 | 1.76 | 3.95E-02 | Novel gene |
| *GPRC5C* | 17 | 72407480 | 72467797 | 251 | 1.76 | 3.95E-02 | Novel gene |
| *GPD2* | 2 | 157271965 | 157462915 | 569 | 1.76 | 3.95E-02 | Novel gene |
| *LOXL2* | 8 | 23134410 | 23281722 | 958 | 1.76 | 3.95E-02 | Novel gene |
| *DMAP1* | 1 | 44659104 | 44706351 | 105 | 1.76 | 3.96E-02 | Novel gene |
| *MEST* | 7 | 130106016 | 130166138 | 169 | 1.76 | 3.96E-02 | Novel gene |
| *ZNF570* | 19 | 37938463 | 37996242 | 146 | 1.75 | 3.96E-02 | Novel gene |
| *LNX2* | 13 | 28100048 | 28214720 | 455 | 1.75 | 3.98E-02 | Novel gene |
| *TIGD1* | 2 | 233392779 | 233435226 | 155 | 1.75 | 3.99E-02 | Novel gene |
| *MED9* | 17 | 17360300 | 17416534 | 160 | 1.75 | 4.00E-02 | Novel gene |
| *SMC3* | 10 | 112307449 | 112384392 | 354 | 1.75 | 4.02E-02 | Novel gene |
| *MXD1* | 2 | 70122173 | 70190077 | 201 | 1.75 | 4.02E-02 | Novel gene |
| *LAG3* | 12 | 6861670 | 6907621 | 133 | 1.75 | 4.02E-02 | Novel gene |
| *USP47* | 11 | 11842970 | 12000872 | 530 | 1.75 | 4.03E-02 | Novel gene |
| *SNRPA* | 19 | 41236725 | 41291297 | 195 | 1.75 | 4.03E-02 | Novel gene |
| *GLRX5* | 14 | 95981323 | 96031055 | 272 | 1.75 | 4.03E-02 | Reported gene on tuberculosis |
| *HTR5A* | 7 | 154842034 | 154899102 | 385 | 1.75 | 4.03E-02 | Novel gene |
| *SPRED1* | 15 | 38524925 | 38669450 | 531 | 1.75 | 4.03E-02 | Novel gene |
| *RBPMS2* | 15 | 65012093 | 65087770 | 260 | 1.75 | 4.03E-02 | Novel gene |
| *MS4A14* | 11 | 60143487 | 60205229 | 222 | 1.75 | 4.05E-02 | Novel gene |
| *EPCAM* | 2 | 47576287 | 47634167 | 264 | 1.74 | 4.05E-02 | Novel gene |
| *LARGE* | 22 | 33648509 | 34336464 | 2988 | 1.74 | 4.06E-02 | Novel gene |
| *RIN3* | 14 | 92960125 | 93175339 | 851 | 1.74 | 4.06E-02 | Reported gene on lung-related and respiratory-related diseases |
| *MT1F* | 16 | 56671855 | 56713215 | 312 | 1.74 | 4.06E-02 | Novel gene |
| *OLFML2A* | 9 | 127519435 | 127597161 | 200 | 1.74 | 4.07E-02 | Novel gene |
| *LY6D* | 8 | 143846298 | 143888008 | 245 | 1.74 | 4.09E-02 | Novel gene |
| *ZNF554* | 19 | 2799872 | 2856733 | 168 | 1.74 | 4.09E-02 | Novel gene |
| *TMUB2* | 17 | 42244300 | 42289099 | 105 | 1.74 | 4.10E-02 | Novel gene |
| *CR2* | 1 | 207607645 | 207683240 | 272 | 1.74 | 4.10E-02 | Novel gene |
| *C10orf76* | 10 | 103585356 | 103835932 | 534 | 1.74 | 4.11E-02 | Novel gene |
| *WASL* | 7 | 123301981 | 123409125 | 402 | 1.74 | 4.12E-02 | Novel gene |
| *TMEM59L* | 19 | 18703682 | 18751849 | 185 | 1.74 | 4.12E-02 | Novel gene |
| *ZP3* | 7 | 76006841 | 76091388 | 308 | 1.74 | 4.13E-02 | Novel gene |
| *SLX4IP* | 20 | 10395951 | 10624551 | 796 | 1.74 | 4.13E-02 | Reported gene on lung-related diseases |
| *EIF4B* | 12 | 53379942 | 53455993 | 247 | 1.73 | 4.14E-02 | Novel gene |
| *ZNF582* | 19 | 56866595 | 56924970 | 177 | 1.73 | 4.15E-02 | Novel gene |
| *OR7D2* | 19 | 9276270 | 9319493 | 139 | 1.73 | 4.16E-02 | Novel gene |
| *PAAF1* | 11 | 73567744 | 73658781 | 381 | 1.73 | 4.17E-02 | Novel gene |
| *SCRN1* | 7 | 29939719 | 30049905 | 349 | 1.73 | 4.18E-02 | Novel gene |
| *UGT1A7* | 2 | 234570584 | 234701945 | 616 | 1.73 | 4.19E-02 | Novel gene |
| *GBP2* | 1 | 89551815 | 89611842 | 240 | 1.73 | 4.19E-02 | Novel gene |
| *BET1L* | 11 | 182924 | 227422 | 139 | 1.73 | 4.19E-02 | Novel gene |
| *ASPSCR1* | 17 | 79915426 | 79995282 | 199 | 1.73 | 4.20E-02 | Reported gene on lung-related diseases |
| *SRP54* | 14 | 35431901 | 35518773 | 373 | 1.73 | 4.20E-02 | Novel gene |
| *POP5* | 12 | 120996848 | 121039201 | 155 | 1.73 | 4.21E-02 | Novel gene |
| *RNMT* | 18 | 13706659 | 13784555 | 252 | 1.73 | 4.21E-02 | Novel gene |
| *ZNF486* | 19 | 20258020 | 20331299 | 217 | 1.73 | 4.21E-02 | Novel gene |
| *UGT1A9* | 2 | 234560544 | 234701951 | 649 | 1.73 | 4.21E-02 | Novel gene |
| *TRABD* | 22 | 50604344 | 50658027 | 269 | 1.72 | 4.23E-02 | Novel gene |
| *DNAJC9* | 10 | 74982582 | 75027025 | 146 | 1.72 | 4.25E-02 | Novel gene |
| *PDS5A* | 4 | 39804483 | 39999576 | 672 | 1.72 | 4.26E-02 | Novel gene |
| *GPR85* | 7 | 112700468 | 112747833 | 168 | 1.72 | 4.28E-02 | Novel gene |
| *APOBEC3F* | 22 | 39416609 | 39471977 | 137 | 1.72 | 4.28E-02 | Novel gene |
| *EIF4E2* | 2 | 233394762 | 233468354 | 226 | 1.72 | 4.29E-02 | Reported gene on lung-related diseases |
| *GIPC3* | 19 | 3565557 | 3613539 | 142 | 1.72 | 4.30E-02 | Novel gene |
| *TERT* | 5 | 1233282 | 1315178 | 263 | 1.72 | 4.31E-02 | Reported gene on lung-related and respiratory-related diseases |
| *ZNF665* | 19 | 53646552 | 53716619 | 243 | 1.72 | 4.31E-02 | Reported gene on respiratory-related diseases |
| *MRPS16* | 10 | 74988601 | 75032451 | 137 | 1.72 | 4.31E-02 | Novel gene |
| *PLG* | 6 | 161103225 | 161195086 | 387 | 1.72 | 4.32E-02 | Novel gene |
| *ALK* | 2 | 29395640 | 30164477 | 3517 | 1.71 | 4.32E-02 | Novel gene |
| *CMTR1* | 6 | 37380907 | 37470637 | 239 | 1.71 | 4.33E-02 | Novel gene |
| *GCAT* | 22 | 38183906 | 38233183 | 165 | 1.71 | 4.33E-02 | Novel gene |
| *RASD1* | 17 | 17377751 | 17419709 | 115 | 1.71 | 4.34E-02 | Novel gene |
| *RAB11FIP5* | 2 | 73280510 | 73360146 | 213 | 1.71 | 4.35E-02 | Novel gene |
| *MAP3K8* | 10 | 30702950 | 30770762 | 175 | 1.71 | 4.36E-02 | Novel gene |
| *CDH12* | 5 | 21730870 | 22873731 | 3642 | 1.71 | 4.36E-02 | Novel gene |
| *LOC101928764* | 13 | 21840557 | 21891219 | 239 | 1.71 | 4.37E-02 | Novel gene |
| *VPS28* | 8 | 145628984 | 145673946 | 149 | 1.71 | 4.38E-02 | Novel gene |
| *FLJ20712* | 7 | 33745567 | 33788068 | 132 | 1.71 | 4.38E-02 | Novel gene |
| *RALGPS2* | 1 | 178674300 | 178910975 | 684 | 1.71 | 4.38E-02 | Reported gene on lung-related diseases |
| *UGT1A6* | 2 | 234580321 | 234701951 | 575 | 1.71 | 4.38E-02 | Novel gene |
| *FGL2* | 7 | 76802688 | 76849150 | 118 | 1.71 | 4.39E-02 | Novel gene |
| *KLHDC7A* | 1 | 18787424 | 18832540 | 269 | 1.71 | 4.39E-02 | Novel gene |
| *RNF144B* | 6 | 18257602 | 18489105 | 1190 | 1.71 | 4.40E-02 | Novel gene |
| *TSPAN16* | 19 | 11386798 | 11457672 | 270 | 1.70 | 4.42E-02 | Novel gene |
| *CRIP1* | 14 | 105932892 | 105975128 | 86 | 1.70 | 4.43E-02 | Novel gene |
| *RUNX3* | 1 | 25206002 | 25311648 | 341 | 1.70 | 4.43E-02 | Reported gene on lung-related and respiratory-related diseases |
| *AKIRIN2* | 6 | 88364578 | 88431985 | 252 | 1.70 | 4.43E-02 | Novel gene |
| *SAMD8* | 10 | 76851275 | 76961881 | 299 | 1.70 | 4.45E-02 | Novel gene |
| *NXPH2* | 2 | 139406727 | 139557811 | 481 | 1.70 | 4.47E-02 | Novel gene |
| *SCGB1C1* | 11 | 168101 | 214575 | 80 | 1.70 | 4.48E-02 | Novel gene |
| *NSMAF* | 8 | 59476063 | 59592404 | 332 | 1.70 | 4.48E-02 | Novel gene |
| *SLC1A2* | 11 | 35252752 | 35461610 | 822 | 1.70 | 4.48E-02 | Reported gene on lung-related diseases |
| *IL6R* | 1 | 154357669 | 154461926 | 295 | 1.70 | 4.49E-02 | Reported gene on respiratory-related diseases |
| *ZNF569* | 19 | 37881778 | 37980167 | 236 | 1.70 | 4.49E-02 | Novel gene |
| *RAD54B* | 8 | 95364188 | 95507343 | 521 | 1.70 | 4.49E-02 | Novel gene |
| *MKRN2OS* | 3 | 12561280 | 12606963 | 168 | 1.70 | 4.49E-02 | Novel gene |
| *HIF1AN* | 10 | 102275641 | 102333681 | 183 | 1.70 | 4.49E-02 | Novel gene |
| *KIAA1109* | 4 | 123053488 | 123303914 | 699 | 1.70 | 4.50E-02 | Reported gene on respiratory-related diseases |
| *DHX34* | 19 | 47832538 | 47905961 | 288 | 1.70 | 4.50E-02 | Novel gene |
| *DCLRE1C* | 10 | 14928870 | 15016106 | 325 | 1.69 | 4.51E-02 | Novel gene |
| *APOBEC3B* | 22 | 39358352 | 39408784 | 85 | 1.69 | 4.51E-02 | Reported gene on respiratory-related diseases |
| *PARVA* | 11 | 12377988 | 12576903 | 860 | 1.69 | 4.53E-02 | Reported gene on lung-related and respiratory-related diseases |
| *EID2* | 19 | 40009446 | 40050838 | 184 | 1.69 | 4.53E-02 | Novel gene |
| *DCTD* | 4 | 183791244 | 183858630 | 358 | 1.69 | 4.54E-02 | Novel gene |
| *NUDT8* | 11 | 67375409 | 67417408 | 169 | 1.69 | 4.55E-02 | Novel gene |
| *SLC14A2* | 18 | 42772947 | 43283072 | 2097 | 1.69 | 4.56E-02 | Reported gene on lung-related diseases |
| *CDH15* | 16 | 89218163 | 89281900 | 407 | 1.69 | 4.57E-02 | Novel gene |
| *EFHD1* | 2 | 233450767 | 233567491 | 352 | 1.69 | 4.57E-02 | Reported gene on respiratory-related diseases |
| *TSR1* | 17 | 2205972 | 2260678 | 180 | 1.69 | 4.57E-02 | Novel gene |
| *TBC1D10B* | 16 | 30348422 | 30401522 | 108 | 1.69 | 4.58E-02 | Novel gene |
| *CYB5A* | 18 | 71900527 | 71979251 | 462 | 1.69 | 4.58E-02 | Novel gene |
| *CHRNG* | 2 | 233384424 | 233431038 | 180 | 1.69 | 4.58E-02 | Novel gene |
| *ATP6V1B1* | 2 | 71142998 | 71212561 | 254 | 1.69 | 4.59E-02 | Novel gene |
| *TEAD2* | 19 | 49823852 | 49885714 | 216 | 1.69 | 4.59E-02 | Novel gene |
| *PIANP* | 12 | 6782957 | 6830009 | 115 | 1.69 | 4.59E-02 | Novel gene |
| *RNF157* | 17 | 74118534 | 74256448 | 419 | 1.69 | 4.60E-02 | Novel gene |
| *GRM2* | 3 | 51721081 | 51772629 | 108 | 1.68 | 4.61E-02 | Novel gene |
| *CRISPLD1* | 8 | 75876708 | 75966793 | 371 | 1.68 | 4.62E-02 | Novel gene |
| *KDM5B* | 1 | 202676532 | 202797549 | 378 | 1.68 | 4.62E-02 | Novel gene |
| *KLHDC4* | 16 | 87710239 | 87819603 | 648 | 1.68 | 4.63E-02 | Novel gene |
| *EYA3* | 1 | 28276855 | 28435173 | 553 | 1.68 | 4.63E-02 | Novel gene |
| *SULT4A1* | 22 | 44200387 | 44278378 | 326 | 1.68 | 4.63E-02 | Novel gene |
| *DZANK1* | 20 | 18344011 | 18467995 | 498 | 1.68 | 4.63E-02 | Novel gene |
| *TIMM50* | 19 | 39951052 | 40001528 | 207 | 1.68 | 4.63E-02 | Novel gene |
| *RBM11* | 21 | 15568466 | 15620693 | 397 | 1.68 | 4.64E-02 | Novel gene |
| *TRMT44* | 4 | 8422532 | 8498282 | 366 | 1.68 | 4.65E-02 | Novel gene |
| *ALOXE3* | 17 | 7979218 | 8042365 | 229 | 1.68 | 4.65E-02 | Novel gene |
| *FKBP14* | 7 | 30030199 | 30086417 | 123 | 1.68 | 4.66E-02 | Novel gene |
| *TRAPPC1* | 17 | 7813663 | 7855317 | 157 | 1.68 | 4.67E-02 | Novel gene |
| *LRP10* | 14 | 23320822 | 23370789 | 113 | 1.68 | 4.67E-02 | Novel gene |
| *NEK11* | 3 | 130725694 | 131089309 | 1311 | 1.68 | 4.68E-02 | Novel gene |
| *SMEK2* | 2 | 55754428 | 55864860 | 459 | 1.68 | 4.68E-02 | Novel gene |
| *FOCAD* | 9 | 20638309 | 21015954 | 1379 | 1.68 | 4.68E-02 | Novel gene |
| *TGFBR3* | 1 | 92125900 | 92391559 | 1056 | 1.67 | 4.70E-02 | Reported gene on lung-related and respiratory-related diseases |
| *UGT2A1* | 4 | 70434135 | 70538967 | 450 | 1.67 | 4.70E-02 | Novel gene |
| *KLF12* | 13 | 74240149 | 74728400 | 1693 | 1.67 | 4.71E-02 | Novel gene |
| *CTHRC1* | 8 | 104363743 | 104415233 | 234 | 1.67 | 4.71E-02 | Novel gene |
| *USP34* | 2 | 61394590 | 61717868 | 1020 | 1.67 | 4.71E-02 | Reported gene on lung-related diseases |
| *CRIM1* | 2 | 36563370 | 36798278 | 807 | 1.67 | 4.73E-02 | Reported gene on lung-related and respiratory-related diseases |
| *ZNF540* | 19 | 38022273 | 38125080 | 262 | 1.67 | 4.74E-02 | Reported gene on tuberculosis |
| *EVX2* | 2 | 176924835 | 176968690 | 141 | 1.67 | 4.74E-02 | Novel gene |
| *CCDC42B* | 12 | 113567543 | 113617081 | 165 | 1.67 | 4.75E-02 | Novel gene |
| *UHRF1* | 19 | 4889510 | 4982165 | 346 | 1.67 | 4.75E-02 | Novel gene |
| *SLC8A1* | 2 | 40319286 | 40759575 | 1947 | 1.67 | 4.76E-02 | Reported gene on lung-related and respiratory-related diseases |
| *ZBTB48* | 1 | 6620051 | 6669340 | 177 | 1.67 | 4.77E-02 | Novel gene |
| *SWAP70* | 11 | 9665624 | 9794508 | 559 | 1.67 | 4.77E-02 | Novel gene |
| *KIAA1191* | 5 | 175753065 | 175808809 | 159 | 1.67 | 4.78E-02 | Novel gene |
| *DAPL1* | 2 | 159631829 | 159692506 | 246 | 1.67 | 4.78E-02 | Novel gene |
| *TCF7* | 5 | 133430402 | 133503920 | 256 | 1.67 | 4.78E-02 | Novel gene |
| *MKRN2* | 3 | 12578513 | 12645212 | 222 | 1.67 | 4.79E-02 | Novel gene |
| *TUBGCP5* | 15 | 22813395 | 22893891 | 240 | 1.66 | 4.80E-02 | Novel gene |
| *FAM149B1* | 10 | 74907877 | 75022435 | 349 | 1.66 | 4.80E-02 | Novel gene |
| *PSKH2* | 8 | 87040691 | 87101851 | 256 | 1.66 | 4.81E-02 | Novel gene |
| *BTF3* | 5 | 72774250 | 72821448 | 185 | 1.66 | 4.81E-02 | Novel gene |
| *PTGDR2* | 11 | 60598398 | 60643444 | 219 | 1.66 | 4.81E-02 | Novel gene |
| *CAMK1D* | 10 | 12371583 | 12891735 | 2550 | 1.66 | 4.82E-02 | Reported gene on lung-related diseases |
| *CACTIN* | 19 | 3590626 | 3646813 | 224 | 1.66 | 4.83E-02 | Novel gene |
| *RFESD* | 5 | 94962471 | 95012849 | 161 | 1.66 | 4.83E-02 | Novel gene |
| *NAPRT* | 8 | 144636955 | 144680881 | 232 | 1.66 | 4.84E-02 | Novel gene |
| *KRT20* | 17 | 39012141 | 39061495 | 170 | 1.66 | 4.84E-02 | Novel gene |
| *ZNF639* | 3 | 179021551 | 179073325 | 179 | 1.66 | 4.84E-02 | Novel gene |
| *UGT2A2* | 4 | 70434135 | 70525360 | 412 | 1.66 | 4.85E-02 | Novel gene |
| *CDX2* | 13 | 28516205 | 28563505 | 156 | 1.66 | 4.85E-02 | Novel gene |
| *ILK* | 11 | 6604938 | 6652105 | 173 | 1.66 | 4.86E-02 | Novel gene |
| *GLTSCR1* | 19 | 48091453 | 48226534 | 534 | 1.66 | 4.86E-02 | Novel gene |
| *EPB41L2* | 6 | 131140487 | 131404462 | 1070 | 1.66 | 4.86E-02 | Reported gene on lung-related diseases |
| *N4BP2* | 4 | 40038524 | 40179872 | 429 | 1.66 | 4.87E-02 | Novel gene |
| *TMTC1* | 12 | 29633746 | 29957692 | 1581 | 1.66 | 4.87E-02 | Novel gene |
| *C9orf163* | 9 | 139357947 | 139400519 | 180 | 1.66 | 4.88E-02 | Novel gene |
| *ASB1* | 2 | 239315382 | 239380891 | 283 | 1.66 | 4.88E-02 | Reported gene on lung-related and respiratory-related diseases |
| *CTSC* | 11 | 88006760 | 88090941 | 320 | 1.66 | 4.90E-02 | Novel gene |
| *PCP4L1* | 1 | 161208517 | 161275240 | 299 | 1.65 | 4.90E-02 | Novel gene |
| *DNMT3L* | 21 | 45646222 | 45702099 | 283 | 1.65 | 4.91E-02 | Novel gene |
| *TTC21B* | 2 | 166709872 | 166830348 | 460 | 1.65 | 4.91E-02 | Novel gene |
| *RNPS1* | 16 | 2283100 | 2338413 | 133 | 1.65 | 4.93E-02 | Novel gene |
| *VN1R5* | 1 | 247399374 | 247440447 | 148 | 1.65 | 4.93E-02 | Novel gene |
| *PAQR5* | 15 | 69571294 | 69719976 | 611 | 1.65 | 4.94E-02 | Novel gene |
| *ATP6AP1L* | 5 | 81581166 | 81634147 | 160 | 1.65 | 4.95E-02 | Novel gene |
| *NTSR1* | 20 | 61320189 | 61414123 | 503 | 1.65 | 4.98E-02 | Novel gene |
| *NAA38* | 17 | 7740003 | 7808608 | 185 | 1.65 | 4.98E-02 | Novel gene |
| *HOXB5* | 17 | 46648619 | 46691103 | 133 | 1.65 | 4.98E-02 | Novel gene |
| *TRIAP1* | 12 | 120861764 | 120904215 | 155 | 1.65 | 4.98E-02 | Novel gene |
| *MRTO4* | 1 | 19558075 | 19606622 | 251 | 1.65 | 4.99E-02 | Novel gene |
| *VRK1* | 14 | 97243684 | 97367951 | 436 | 1.65 | 4.99E-02 | Novel gene |
| *ARHGEF40* | 14 | 21518419 | 21578407 | 161 | 1.65 | 5.00E-02 | Novel gene |
| *SPATA2L* | 16 | 89742765 | 89788131 | 231 | 1.65 | 5.00E-02 | Novel gene |
| *FIBCD1* | 9 | 133757825 | 133834455 | 336 | 1.65 | 5.00E-02 | Novel gene |

**Note:** Reported genes mean these genes have been documented to be associated with tuberculosis, lung-related and respiratory-related disease in the GWAS Catalog database; Novel genes mean these genes were not documented in the GWAS Catalog database.
