# Supplementary material for: Integrative genomics analysis identifies promising SNPs and genes implicated in tuberculosis risk based on multiple omics datasets
Source: Aging (Albany NY). 2020 Oct 13;12(19):19173–220. doi: 10.18632/aging.103744 (PMC7732298; doi:10.18632/aging.103744)
Supplement: Supplementary Tables 15, 16 and 17 [file aging-12-103744-s010..pdf]

SUPPLEMENTARY TABLES

Supplementary Table 15. 21 pathways enriched by using MAGMA gene-set analysis based on the KEGG pathway resource.

Pathway Name	Gene Number	Beta	MAGMA-based P values	KOBAS-based P values (genes from the discovery stage)
Pyruvate metabolism	38	0.35	4.88E-03	5.70E-02
Acute myeloid leukemia	53	0.28	5.67E-03	1.03E-02
Toxoplasmosis	121	0.20	6.47E-03	6.68E-04
Type II diabetes mellitus	44	0.30	9.75E-03	3.13E-03
Neurotrophin signaling pathway	121	0.17	9.91E-03	8.91E-04
RIG-I-like receptor signaling pathway	62	0.27	1.09E-02	5.87E-02
B cell receptor signaling pathway	70	0.20	1.55E-02	4.15E-03
Adipocytokine signaling pathway	64	0.22	1.69E-02	5.68E-02
Natural killer cell mediated cytotoxicity	128	0.16	1.81E-02	5.60E-05
VEGF signaling pathway	73	0.20	2.11E-02	1.32E-03
Insulin signaling pathway	127	0.14	2.46E-02	1.93E-03
Toll-like receptor signaling pathway	95	0.18	2.55E-02	2.26E-03
Jak-STAT signaling pathway	142	0.14	2.69E-02	4.68E-03
Drug metabolism - cytochrome P450	71	0.23	2.70E-02	4.98E-05
mTOR signaling pathway	45	0.21	3.08E-02	8.13E-04
Prostate cancer	85	0.16	3.42E-02	3.44E-02
Pancreatic cancer	66	0.16	4.16E-02	6.88E-02
Hepatitis C	128	0.12	4.62E-02	1.87E-04
Metabolism of xenobiotics by cytochrome P450	71	0.20	4.74E-02	4.90E-04

Supplementary Table 16. The proportion of multiple layers of evidence in constructed GGI network using the GeneMANIA tool.

ID	Evidence of interactions	Proportions
1	Co-expression links	71.52%
2	Predicted links	19.09%
3	Physical interactions	8.44%
4	Pathways	0.39%
5	Genetic interactions	0.31%
6	Shared protein domains	0.23%
7	Co-localization	0.02%

Supplementary Table 17. Differential expression analysis of 26 candidate genes between infected cells and uninfected cells based on the GSE133803 data.

Gene	GSM3927531 (infected cells)	GSM3927532 (infected cells)	GSM3927533 (infected cells)	GSM3927534 (uninfected cells)	GSM3927535 (uninfected cells)	GSM3927536 (uninfected cells)	P values (t-test)
RPS23	9.91	9.69	9.47	12.03	12.05	12.02	5.45E-05
RPS5	11.52	11.14	11.08	13.08	13.16	13.02	2.11E-04
CLN8	6.74	6.85	6.77	7.78	7.78	7.76	8.18E-06
SPATA20	10.15	9.77	9.63	10.97	11.02	10.95	1.98E-03
CDC16	11.36	11.11	11.05	12.33	12.45	12.22	5.63E-04
TMEM99	9.40	9.42	9.09	10.31	10.37	10.11	2.00E-03
LIG3	7.90	7.65	7.67	8.51	8.49	8.35	1.86E-03
RRM1	10.49	10.24	10.00	11.13	11.16	11.12	3.24E-03
SCAPER	8.10	8.04	7.96	8.44	8.41	8.45	8.84E-04
ZNF266	6.78	6.76	6.74	6.77	7.17	7.24	0.110
RCN3	11.07	10.79	10.91	11.37	11.51	11.31	8.73E-03
CARD9	7.13	7.39	7.09	7.55	7.44	7.43	5.48E-02
TBRG4	8.66	8.24	8.44	8.74	8.77	8.63	0.111
ZNF502	7.62	7.67	7.30	7.84	7.75	7.68	0.143
ZNF197	6.96	7.05	6.91	7.06	7.25	7.18	5.40E-02
NUDT13	7.06	6.77	6.96	7.03	7.27	6.96	0.281
HDAC10	7.14	6.78	6.88	7.05	7.06	6.98	0.424
TDRKH	6.91	6.69	6.77	6.64	6.77	6.89	0.833
PDK1	7.24	7.13	7.04	7.13	7.26	6.95	0.825
CDK10	6.77	6.74	6.59	6.68	6.59	6.68	0.494
DHX57	6.70	6.81	6.69	6.64	6.80	6.59	0.475
NPHP4	8.00	7.78	7.86	7.87	7.82	7.62	0.318
ZNF354A	7.29	7.26	7.49	7.24	7.34	7.09	0.293
FCHO1	6.77	6.90	6.65	6.42	6.75	6.67	0.267
MAP1S	10.71	10.32	10.30	9.81	9.88	9.71	1.01E-02
HIATL1	10.41	10.42	10.37	8.24	8.31	8.24	1.83E-07

Note: The P values were calculated by using the Student's t test.
