# Supplementary material for: Integrative genomics analysis identifies promising SNPs and genes implicated in tuberculosis risk based on multiple omics datasets
Source: Aging (Albany NY). 2020 Oct 13;12(19):19173–220. doi: 10.18632/aging.103744 (PMC7732298; doi:10.18632/aging.103744)
Supplement: Supplementary Table 20 [file aging-12-103744-s013..docx]

**Supplementary Table 20. Multiple top-ranked eSNPs identified in 26 candidate genes implicated in tuberculosis risk.**

| **Gene name** | **SNP ID** | **Chromosome** | **Position** | **Proximity** | **eQTL P values** | **GWAS P values** | **LBF** | **Gene resources** |
| --- | --- | --- | --- | --- | --- | --- | --- | --- |
| *RPS23* | rs6867982 | 5 | 81666080 | cis | 2.46E-98 | 8.92E-04 | NA | Gene set #1 |
| *RPS23* | rs6870033 | 5 | 81661404 | cis | 9.84E-09 | 3.46E-04 | 1.71 | Gene set #2 |
| *RPS23* | rs6870033 | 5 | 81661404 | cis | 7.44E-16 | 3.46E-04 | 1.47 | Gene set #3 |
| *RPS5* | rs734379 | 19 | 63590994 | cis | 4.11E-11 | 2.40E-02 | 1.08 | Gene set #1 |
| *RPS5* | rs12609654 | 19 | 63560292 | cis | 1.60E-07 | 1.16E-02 | 0.97 | Gene set #2 |
| *RPS5* | rs7252333 | 19 | 63592559 | cis | 4.86E-10 | 2.26E-02 | 0.91 | Gene set #3 |
| *CLN8* | rs6558527 | 8 | 1690318 | cis | 9.88E-07 | 4.93E-03 | 1.48 | Gene set #1 |
| *CLN8* | rs6558520 | 8 | 1684980 | cis | 3.87E-07 | 2.22E-03 | 1.97 | Gene set #2 |
| *CLN8* | rs6558520 | 8 | 1684980 | cis | 3.17E-10 | 2.22E-03 | 2.01 | Gene set #3 |
| *SPATA20* | rs9913430 | 17 | 45983469 | cis | 2.19E-121 | 1.11E-04 | 2.55 | Gene set #1 |
| *SPATA20* | rs9913430 | 17 | 45983469 | cis | 4.76E-36 | 1.11E-04 | 2.98 | Gene set #2 |
| *SPATA20* | rs9913430 | 17 | 45983469 | cis | 8.78E-31 | 1.11E-04 | 2.80 | Gene set #3 |
| *CDC16* | rs948182 | 11 | 82423879 | trans | 4.13E-06 | 2.01E-02 | 0.34 | Gene set #1 |
| *CDC16* | rs7987202 | 13 | 113986324 | cis | 4.70E-13 | 2.53E-03 | 2.18 | Gene set #1 |
| *CDC16* | rs9590408 | 13 | 114037568 | cis | 3.79E-49 | 2.02E-03 | 1.85 | Gene set #2 |
| *CDC16* | rs9590408 | 13 | 114037568 | cis | 5.60E-43 | 2.02E-03 | 1.95 | Gene set #3 |
| *TMEM99* | rs16966294 | 17 | 36205020 | cis | 6.82E-08 | 1.07E-03 | 2.92 | Gene set #1 |
| *TMEM99* | rs6056100 | 20 | 8705812 | trans | 6.57E-06 | 3.59E-02 | 0.11 | Gene set #1 |
| *TMEM99* | rs7222207 | 17 | 36208699 | cis | 9.81E-06 | 1.13E-03 | 3.12 | Gene set #2 |
| *TMEM99* | rs7211017 | 17 | 36211479 | cis | 2.79E-11 | 4.43E-03 | 1.61 | Gene set #3 |
| *LIG3* | rs234819 | 17 | 30236979 | cis | 1.27E-23 | 6.53E-03 | 1.14 | Gene set #1 |
| *LIG3* | rs4796030 | 17 | 30354263 | cis | 9.69E-11 | 6.75E-03 | 1.09 | Gene set #2 |
| *LIG3* | rs170923 | 17 | 30200207 | cis | 2.20E-09 | 4.09E-03 | 1.69 | Gene set #3 |
| *RRM1* | rs957252 | 9 | 26049028 | trans | 3.71E-06 | 5.47E-02 | 0.14 | Gene set #1 |
| *RRM1* | rs4910582 | 11 | 3845959 | cis | 1.04E-06 | 4.36E-03 | 0.92 | Gene set #1 |
| *RRM1* | rs7126870 | 11 | 3846635 | cis | 3.77E-07 | 8.88E-04 | 1.54 | Gene set #2 |
| *RRM1* | rs881411 | 11 | 4167103 | cis | 1.41E-06 | 2.74E-03 | 1.33 | Gene set #3 |
| *SCAPER* | rs437131 | 15 | 74568106 | cis | 5.83E-73 | 1.44E-03 | 1.44 | Gene set #1 |
| *SCAPER* | rs2461872 | 15 | 74564669 | cis | 7.02E-23 | 1.41E-03 | 1.73 | Gene set #2 |
| *SCAPER* | rs2461872 | 15 | 74564669 | cis | 5.84E-26 | 1.41E-03 | 1.57 | Gene set #3 |
| *ZNF266* | rs10415281 | 19 | 9460034 | cis | 1.11E-54 | 4.38E-03 | 1.07 | Gene set #1 |
| *ZNF266* | rs2161285 | 19 | 9484107 | cis | 1.29E-35 | 3.21E-03 | 1.24 | Gene set #2 |
| *ZNF266* | rs2161285 | 19 | 9484107 | cis | 3.78E-34 | 3.21E-03 | 1.03 | Gene set #3 |
| *RCN3* | rs2946863 | 19 | 54704857 | cis | 3.26E-07 | 6.42E-03 | 1.60 | Gene set #1 |
| *RCN3* | rs2878342 | 19 | 54709536 | cis | 2.70E-12 | 3.82E-03 | 2.39 | Gene set #2 |
| *RCN3* | rs3810194 | 19 | 54719852 | cis | 6.65E-06 | 1.43E-02 | 1.83 | Gene set #3 |
| *CARD9* | rs3812584 | 9 | 138432540 | cis | 1.06E-57 | 2.40E-03 | 1.89 | Gene set #1 |
| *CARD9* | rs10870165 | 9 | 138436422 | cis | 8.46E-18 | 2.11E-03 | 1.67 | Gene set #2 |
| *CARD9* | rs3812584 | 9 | 138432540 | cis | 4.90E-06 | 2.40E-03 | 1.05 | Gene set #3 |
| *TBRG4* | rs1476618 | 7 | 45013356 | cis | 9.00E-07 | 4.11E-02 | 0.66 | Gene set #1 |
| *TBRG4* | rs1058348 | 10 | 11342351 | trans | 2.35E-08 | 2.78E-02 | 1.22 | Gene set #1 |
| *TBRG4* | rs10264842 | 7 | 45053907 | cis | 2.24E-07 | 2.22E-02 | 1.10 | Gene set #2 |
| *TBRG4* | rs10264842 | 7 | 45053907 | cis | 3.51E-08 | 2.22E-02 | 0.93 | Gene set #3 |
| *ZNF502* | rs11709024 | 3 | 44702557 | cis | 6.18E-09 | 6.73E-03 | 1.14 | Gene set #1 |
| *ZNF502* | rs11709024 | 3 | 44702557 | cis | 7.94E-09 | 6.73E-03 | 1.57 | Gene set #2 |
| *ZNF502* | rs1506659 | 3 | 44887447 | cis | 1.54E-11 | 1.90E-03 | 1.48 | Gene set #3 |
| *ZNF197* | rs11709024 | 3 | 44702557 | cis | 3.62E-17 | 6.73E-03 | 1.19 | Gene set #1 |
| *ZNF197* | rs395136 | 19 | 37798582 | trans | 7.94E-06 | 3.98E-02 | 0.20 | Gene set #1 |
| *ZNF197* | rs11709024 | 3 | 44702557 | cis | 1.50E-06 | 6.73E-03 | 1.37 | Gene set #2 |
| *ZNF197* | rs1506659 | 3 | 44887447 | cis | 8.37E-08 | 1.90E-03 | 1.63 | Gene set #3 |
| *NUDT13* | rs12243451 | 10 | 74492672 | cis | 1.66E-10 | 2.79E-02 | 1.08 | Gene set #1 |
| *NUDT13* | rs8045050 | 16 | 76179102 | trans | 5.07E-06 | 1.14E-02 | 0.90 | Gene set #1 |
| *NUDT13* | rs12248308 | 10 | 74461628 | cis | 1.06E-09 | 2.34E-02 | 1.12 | Gene set #2 |
| *NUDT13* | rs12248308 | 10 | 74461628 | cis | 4.90E-36 | 2.34E-02 | 1.07 | Gene set #3 |
| *HDAC10* | rs2351482 | 5 | 153818101 | trans | 1.55E-06 | 9.55E-03 | 1.16 | Gene set #1 |
| *HDAC10* | rs10505751 | 12 | 10100978 | trans | 3.32E-06 | 5.67E-03 | 1.20 | Gene set #1 |
| *HDAC10* | rs11664741 | 18 | 45388463 | trans | 4.32E-06 | 3.31E-02 | 0.24 | Gene set #1 |
| *HDAC10* | rs7290465 | 22 | 49021885 | cis | 1.58E-19 | 1.03E-02 | 1.60 | Gene set #1 |
| *HDAC10* | rs1129880 | 22 | 49036424 | cis | 1.92E-19 | 6.55E-03 | 1.39 | Gene set #2 |
| *HDAC10* | rs1129880 | 22 | 49036424 | cis | 1.79E-20 | 6.55E-03 | 1.43 | Gene set #3 |
| *TDRKH* | rs3828057 | 1 | 150046801 | cis | 4.58E-09 | 7.65E-04 | 2.12 | Gene set #1 |
| *TDRKH* | rs289159 | 15 | 60382612 | trans | 9.60E-06 | 1.25E-02 | 0.29 | Gene set #1 |
| *TDRKH* | rs3828057 | 1 | 150046801 | cis | 8.80E-08 | 7.65E-04 | 2.24 | Gene set #2 |
| *TDRKH* | rs3828057 | 1 | 150046801 | cis | 8.17E-09 | 7.65E-04 | 2.09 | Gene set #3 |
| *PDK1* | rs17104533 | 1 | 48819733 | trans | 4.59E-06 | 7.48E-03 | 1.06 | Gene set #1 |
| *PDK1* | rs10917835 | 1 | 159983917 | trans | 3.71E-07 | 7.08E-03 | 2.35 | Gene set #1 |
| *PDK1* | rs6928974 | 6 | 97334413 | trans | 1.72E-07 | 1.07E-02 | 1.37 | Gene set #1 |
| *PDK1* | rs13398175 | 2 | 173087067 | cis | 3.52E-14 | 1.07E-04 | 3.47 | Gene set #2 |
| *PDK1* | rs3769321 | 2 | 173140600 | cis | 2.80E-06 | 2.01E-02 | 1.37 | Gene set #3 |
| *CDK10* | rs2115401 | 16 | 88268110 | cis | 1.43E-13 | 3.21E-03 | 1.28 | Gene set #1 |
| *CDK10* | rs164745 | 16 | 88237165 | cis | 4.07E-07 | 1.54E-03 | 1.09 | Gene set #2 |
| *CDK10* | rs164745 | 16 | 88237165 | cis | 7.41E-17 | 1.54E-03 | 1.16 | Gene set #3 |
| *NPHP4* | rs12079601 | 1 | 5865334 | cis | 1.52E-10 | 1.12E-03 | 1.39 | Gene set #1 |
| *NPHP4* | rs1058348 | 10 | 11342351 | trans | 2.86E-07 | 2.78E-02 | 1.01 | Gene set #1 |
| *NPHP4* | rs2152922 | 1 | 5923652 | cis | 4.08E-06 | 2.29E-03 | 1.26 | Gene set #2 |
| *NPHP4* | rs12079601 | 1 | 5865334 | cis | 1.12E-13 | 1.12E-03 | 1.08 | Gene set #3 |
| *ZNF354A* | rs17451397 | 5 | 107591099 | trans | 2.21E-06 | 3.00E-02 | 0.53 | Gene set #1 |
| *ZNF354A* | rs4419608 | 5 | 178029655 | cis | 1.20E-08 | 1.84E-02 | 1.32 | Gene set #1 |
| *ZNF354A* | rs11749438 | 5 | 178063851 | cis | 6.34E-15 | 1.32E-02 | 1.01 | Gene set #2 |
| *ZNF354A* | rs11749438 | 5 | 178063851 | cis | 5.34E-10 | 1.32E-02 | 1.07 | Gene set #3 |
| *FCHO1* | rs1058348 | 10 | 11342351 | trans | 3.24E-07 | 2.78E-02 | 0.99 | Gene set #1 |
| *FCHO1* | rs4280376 | 19 | 17722209 | cis | 1.95E-10 | 5.86E-02 | 0.59 | Gene set #1 |
| *FCHO1* | rs4808683 | 19 | 17723925 | cis | 9.98E-15 | 3.39E-03 | 1.72 | Gene set #2 |
| *FCHO1* | rs8107550 | 19 | 17715015 | cis | 2.85E-06 | 4.40E-03 | 1.88 | Gene set #3 |
| *MAP1S* | rs1058348 | 10 | 11342351 | trans | 2.00E-07 | 2.78E-02 | 1.07 | Gene set #1 |
| *MAP1S* | rs8107550 | 19 | 17715015 | cis | 4.49E-07 | 4.40E-03 | 2.44 | Gene set #2 |
| *MAP1S* | rs4808683 | 19 | 17723925 | cis | 2.40E-09 | 3.39E-03 | 2.25 | Gene set #3 |
| *HIATL1* | rs3118766 | 9 | 96065119 | cis | 3.24E-06 | 7.32E-04 | 2.04 | Gene set #1 |
| *HIATL1* | rs3118766 | 9 | 96065119 | cis | 5.45E-07 | 7.32E-04 | 1.99 | Gene set #2 |
| *HIATL1* | rs3118766 | 9 | 96065119 | cis | 3.05E-06 | 7.32E-04 | 1.53 | Gene set #3 |
